# Enrichment and Analysis of Intact Phosphoproteins in Arabidopsis Seedlings

**DOI:** 10.1371/journal.pone.0130763

**Published:** 2015-07-09

**Authors:** Uma K. Aryal, Andrew R. S. Ross, Joan E. Krochko

**Affiliations:** National Research Council of Canada, Saskatoon, SK, S7N 0W9, Canada; Wuhan Botanical Garden, Chinese Academy of Sciences, CHINA

## Abstract

Protein phosphorylation regulates diverse cellular functions and plays a key role in the early development of plants. To complement and expand upon previous investigations of protein phosphorylation in *Arabidopsis* seedlings we used an alternative approach that combines protein extraction under non-denaturing conditions with immobilized metal-ion affinity chromatography (IMAC) enrichment of intact phosphoproteins in Rubisco-depleted extracts, followed by identification using two-dimensional gel electrophoresis (2-DE) and liquid chromatography-tandem mass spectrometry (LC-MS/MS). In-gel trypsin digestion and analysis of selected gel spots identified 144 phosphorylated peptides and residues, of which only18 phosphopeptides and 8 phosphosites were found in the PhosPhAt 4.0 and P^3^DB *Arabidopsis thaliana* phosphorylation site databases. More than half of the 82 identified phosphoproteins were involved in carbohydrate metabolism, photosynthesis/respiration or oxidative stress response mechanisms. Enrichment of intact phosphoproteins prior to 2-DE and LC-MS/MS appears to enhance detection of phosphorylated threonine and tyrosine residues compared with methods that utilize peptide-level enrichment, suggesting that the two approaches are somewhat complementary in terms of phosphorylation site coverage. Comparing results for young seedlings with those obtained previously for mature *Arabidopsis* leaves identified five proteins that are differentially phosphorylated in these tissues, demonstrating the potential of this technique for investigating the dynamics of protein phosphorylation during plant development.

## Introduction

Seedling establishment is a critical stage in plant development, involving the transition from heterotrophic to autotrophic growth.[1] In *Arabidopsis*, seed germination is driven largely by the metabolism of storage products other than lipids, whereas seedling establishment involves the mobilization of seed oil reserves.[2] Triacylglycerol (TAG) is the predominant source of carbon in the seeds of *Arabidopsis* and related species, including *Brassica napus* (canola),[3] and mobilization of TAG supplies the energy and molecular building blocks required for seedling establishment.[1,4] Utilization of TAG and other seed reserves is thought to be controlled and regulated by multiple pathways,[5] and although considerable progress has been made in understanding dormancy and seed germination [6–8] the cellular mechanisms involved in seedling establishment are less well understood.

Following germination the glycerol released from TAG through lipase action is converted to glyceraldehyde-3-phosphate (G-3-P) and then by isomerization to dihydroxyacetone phosphate (DHAP), which can either undergo glycolysis to pyruvate or conversion to hexose via gluconeogenesis.[9] The free fatty acids are catabolized by ß-oxidation in the glyoxysome. A more complete understanding of how this metabolic program is regulated in *Arabidopsis* would increase our knowledge of post-embryonic development in plants and assist in the improvement of canola and other oilseed crops.

One way to achieve this is to study protein phosphorylation during early stage of seedling establishment because reversible phosphorylation of proteins regulates a wide variety of cellular processes during plant growth and development.[10] However, the analysis of protein phosphorylation can be challenging due to the low relative abundance of phosphoproteins and the possibility of phosphorylation at multiple sites within a given protein.[11,12] Affinity enrichment of phosphorylated proteins and/or the component phosphopeptides obtained by proteolysis can significantly enhance the identification of such proteins and the mapping of phosphorylation sites. However, phosphoproteome analysis of certain plant tissues is complicated by the presence of D-ribulose bisphosphate carboxylase/oxygenase (Rubisco),[11,13] an abundant phosphoprotein that inhibits the detection and analysis of other, less abundant plant proteins. Rubisco depletion columns (e.g. Seppro IgY spin columns; GenWay Biotech, San Diego CA, USA) are commercially available and have been used successfully to deplete Rubisco in total protein extracts. [14,15] Advances in phosphopeptide enrichment strategies have also enabled large-scale phosphoproteomic studies in *Arabidopsis*, providing new insights regarding the potential involvement of protein phosphorylation in various stages of plant development.[11,16–23] Despite these efforts, our knowledge of protein phosphorylation events during the transition from heterotrophic to photoautotrophic growth in young seedlings remains incomplete.

Much of the information currently available in protein phosphorylation databases has been generated using peptide-level enrichment strategies, and although affinity purification of intact phosphoproteins has been demonstrated [11,24] the use of protein-level enrichment for phosphoproteome analysis in plants remains largely unexplored. To complement and expand upon previous investigations involving phosphopeptide enrichment we carried out a survey of protein phosphorylation in post-embryonic *Arabidopsis* seedlings (hereafter referred to as young seedlings) using Rubisco depletion and enrichment of intact phosphoproteins by immobilized metal-ion affinity chromatography (IMAC) combined with two-dimensional gel electrophoresis (2-DE) and liquid chromatography-tandem mass spectrometry (LC-MS/MS). The results of this study were then compared with those obtained previously using Rubisco depletion and protein-level enrichment of phosphoproteins from *Arabidopsis* mature leaves [11] to evaluate this approach for monitoring the dynamics of protein phosphorylation during plant development.

## Materials and Methods

### Materials

Seppro Rubisco IgY Spin Columns (GenWay Biotech, San Diego, CA, USA) were obtained from Sigma-Aldrich (St. Louis, MO, USA; Product No. SEP070). Acrylamide, bisacrylamide solution, IPG dry strips (pH 3–10, NL, 17 cm), carrier ampholytes, Precision Plus Protein standards, TEMED, TBP, DTT, and IAA were purchased from Bio-Rad (Hercules, CA). Urea was from Merck KGaA (Darmstadt, Germany), Tris base from Roche Diagnostics (Indianapolis, IN), PPS silent surfactant from Protein Discovery Inc. (Knoxville, TN), and trypsin (sequencing grade) from Promega (Madison, WI, USA). PHOS-Select iron affinity gel beads and SigmaPrep spin columns were purchased from Sigma-Aldrich (St.Louis, MO, USA). All other chemicals were also from Sigma-Aldrich unless otherwise stated, and were of analytical research grade.

### Plant growth and protein extraction

*Arabidopsis thaliana* (L) Heynh (Col-0) seeds were treated with 50% bleach in MilliQ water (v/v) containing 5.25% sodium hypochlorite for 2 min and then with 50% (v/v) ethanol for 2 min before washing 4 times with sterilized Milli-Q water and cultivating in Petri dishes containing 0.5x Murashige and Skoog [25] mineral salts with BactoAgar. Seeds were allowed to imbibe at 4°C for 4 days in the dark before transferring them to a growth chamber maintained at 22°C and a 16-h light/8-h dark cycle. Week-old whole seedlings (including roots) with 2 seed leaves were collected for protein extraction. One gram of seedlings was ground to a powder in liquid nitrogen with 0.5% (w/w) PVPP and homogenized in 2 ml of ice-cold extraction buffer (pH 7.4) containing 10 mM Tris-HCl, 150 mM NaCl, the serine protease inhibitor PMSF (1 mM, freshly prepared in DMSO) and a protease inhibitor cocktail developed for plant cell and tissue extracts (Sigma P-9596, 0.2% v/v), together with phosphatase inhibitors 20 mM sodium fluoride, 1 mM sodium molybdate, 1 mM sodium orthovanadate, and 1 mM sodium β-glycerophosphate. The slurry was stirred for 30 min on ice, filtered through two layers of cheese cloth and centrifuged at 10,000 × g for 15 min at 4°C. After discarding the pellet the amount of protein in the supernatant was determined using the Bradford assay (Bio-Rad) with BSA as the standard, and the final concentration of the sample adjusted to 1 mg/ml using the extraction buffer.

### Rubisco depletion

Each protein sample was filtered through a 0.45 μm spin filter (Millipore) and 500 μl of the extract, containing about 500 μg of protein, was loaded onto a Seppro IgY column. Rubisco was removed according to the manufacturer’s instructions. The protein flow-through and the bound fraction were collected separately, and each precipitated with 5 volumes of ice-cold methanol and 100 mM ammonium acetate at -20°C overnight. After centrifugation at 10,000 × g for 20 min at 4°C, the resulting pellets were thoroughly washed twice with ice-cold 100% methanol and then with 80% ice-cold methanol. Each pellet was briefly dried using a SpeedVac, re-dissolved in the column incubation buffer (6 M urea, 0.25% CHAPS, 50 mM sodium acetate, pH 4.0) to approximately 1 mg/ml, and used for phosphoprotein enrichment by immobilized metal-ion affinity chromatography (IMAC).

### Phosphoprotein enrichment

Enrichment of intact phosphoproteins from Rubisco-depleted samples was carried out as previously described.[11] Briefly, a 500 μl slurry of PHOS-Select iron affinity gel beads (Sigma) was washed 3 times with 0.1% TFA in 30% acetonitrile and equilibrated 3 times with 500 μl of incubation buffer (6 M urea, 0.25% CHAPS, 50 mM sodium acetate, pH 4.0) with centrifugation at 1,000 × g for 1 min between each step before being loaded onto the column. Two ml of Rubisco-depleted protein sample in incubation buffer were loaded onto each spin column (about 2 mg total protein per 500 μl of bead slurry) and incubated for 1 h at room temperature with gentle shaking. Phosphoproteins bound to the IMAC columns were eluted three times with 200 μl of elution buffer (6 M urea, 50 mM Tris-acetate pH 7.5, 0.1 M EDTA, 0.1 M EGTA, 0.25% CHAPS), each time incubating at room temperature for 10 min with gentle shaking, and then centrifuged at 1,000 × g for 1 min. The 3 eluates were pooled and precipitated with methanol as previously described before re-suspension in lysis buffer (7 M urea, 2 M thiourea, 2% CHAPS) to obtain a total protein concentration of 1 μg/μl prior to 2-D gel electrophoresis (2-DE).

### Gel electrophoresis and in-gel digestion

One-dimensional gel electrophoresis was used to resolve proteins from the bound and flow-through fractions obtained during Rubisco depletion on the Seppro column. Ten μl of each fraction containing approximately 10 μg of protein was mixed with 10 μl of gel sample buffer (0.2 M Tris-HCl, pH 6.8, 2% SDS, 10% glycerol,0.02% bromophenol blue) and separated on a 1.0 mm, 12.5% Criterion Tris/HCl gel in a Criterion Cell (Bio-Rad) (13.3 cm × 8.7 cm) at a constant voltage of 150 V. The separated proteins were visualized using Bio-Safe Coomassie Blue stain (Bio-Rad).

For phosphoproteome analysis, 200 μl (200 μg) of IMAC-enriched phosphoprotein sample was mixed with 200 μl of rehydration buffer (7 M urea, 2 M thiorea, 2% CHAPS, 10 mM DTT, 0.5% IPG buffer, pH 3–10), resolved by 2-DE and visualized by silver staining ^10^. Gel images were recorded using an ImageScanner (GE Healthcare) and Phoretix 2D software (v2004) was used to measure the total number of protein spots visualized in each 2-DE gel image. Proteins of interest were excised manually from each gel and digested with trypsin using a MassPREP protein digestion station, according to the protocol (digestion 5.0) recommended by the manufacturer (Micromass, Manchester, UK). Preparation of tryptic peptide samples for LC-MS/MS analysis was carried out as previously described.[11]

### Mass spectrometry and protein identification

Six μl of each 2-D gel protein digest was analyzed using a nanoAQUITY UPLC system (Waters, Milford, MA, USA) interfaced to a quadrupole time-of-flight (Q-TOF) Ultima Global hybrid tandem mass spectrometer (Waters, Mississauga, ON, Canada). Separations were performed using a Waters BEH130 C_18_ nanoAQUITY UPLC analytical column (75 μm, 1.75 mm × 100 mm) at an initial flow rate of 400 nl/min. Mobile phase solvent A was 0.2% formic acid in water and solvent B was 0.2% formic acid in 100% acetonitrile. Separations were performed using the following 55-min solvent program: 99:1 (%A:%B) for 1 min, changing to 90:10 at 16 min, 55:45 at 45 min, and 20:80 at 46 min, at which point the flow rate was changed to 800 nl/min and the gradient held until 52 min before reverting to 99:1 at 53 min. A 5 min seal wash with 10% acetonitrile in water was carried out after the completion of each run.

The Q-TOF MS was operated in the positive ion mode and TOF MS spectra were acquired over the *m/z* range 400–1900 at the rate of one scan/s. Of the multiply charged (2^+^, 3^+^, or 4^+^) peptide ion peaks rising over a threshold, the three most abundant were automatically selected for CID, and product-ion spectra were acquired over the *m/z* range 50–1900 in TOF MS/MS mode. The CID collision energy was selected automatically according to the *m/z* ratio and charge state of the precursor ion. A real-time exclusion window was used to prevent precursor ions with the same *m/z* from being selected for CID and TOF MS/MS within 2 min of their initial acquisition. Data were also acquired using pre-programmed exclusion lists for keratin and trypsin.

Data were processed using MassLynx 4.1 (Waters, Milford, MA) and searched against NCBInr protein sequence database for *Arabidopsis thaliana* (thale cress) using an in-house Mascot server (Version 2.2, Matrix Sciences, UK) and the following parameters: carbamidomethylation of cysteine as the fixed modification; oxidation of methionine and phosphorylation of serine, threonine and tyrosine as variable modifications; mass tolerances of 0.2 Da for MS and 0.5 Da for MS/MS data; and one missed cleavage for tryptic peptides. Peptide MS/MS spectra used for protein identification had to be of sufficient quality, with a signal-to-noise ratio of 3 or greater for annotated fragment ions, including neutral loss peaks associated with de-phosphorylation during CID. Only peptides matched with significant ion scores (P <0.05) and low expectation values (e-value <0.01) were selected. For unambiguous identification, each peptide MS/MS spectrum had to contain at least three sequential y- or b-type ions. Protein identification was regarded as positive if the Mascot score exceeded the 95% confidence threshold, the matched protein contained at least four top-ranking unique peptides, and protein sequence coverage by the matching peptides was >15%. If the same set of peptides matched multiple members of a protein family, or a protein appeared under different names and accession numbers in the database, the entry with the highest score and/or most descriptive name was reported. When protein isoforms were observed, the data were inspected manually. If several isoforms shared the same set of identified peptides the protein with the most matching peptides was accepted as the correct result. The presence of protein isoforms was confirmed and reported based on the identification of at least two unique peptides.

Since the error tolerance of the MS method used (200 mDa) was greater than the mass difference between phosphorylation and sulfation (9.5 mDa), a second error-tolerant search reporting masses to 0.1 mDa was performed to allow sulfation and phosphorylation to be distinguished. Raw MS/MS spectra matched to phosphorylated peptides in the Mascot search were manually inspected and validated using MassLynx 4.1. The spectra were processed to give singly charged, monoisotopic, centroided peaks and compared with the *in silico* fragmentation masses for the matched peptide to confirm neutral loss of phosphoric acid for serine and threonine phosphorylation, or the mass increment of 80 Da associated with phosphorylation of tyrosine.

## Results

### Phosphoproteome analysis of young seedlings

**Fig 1:**
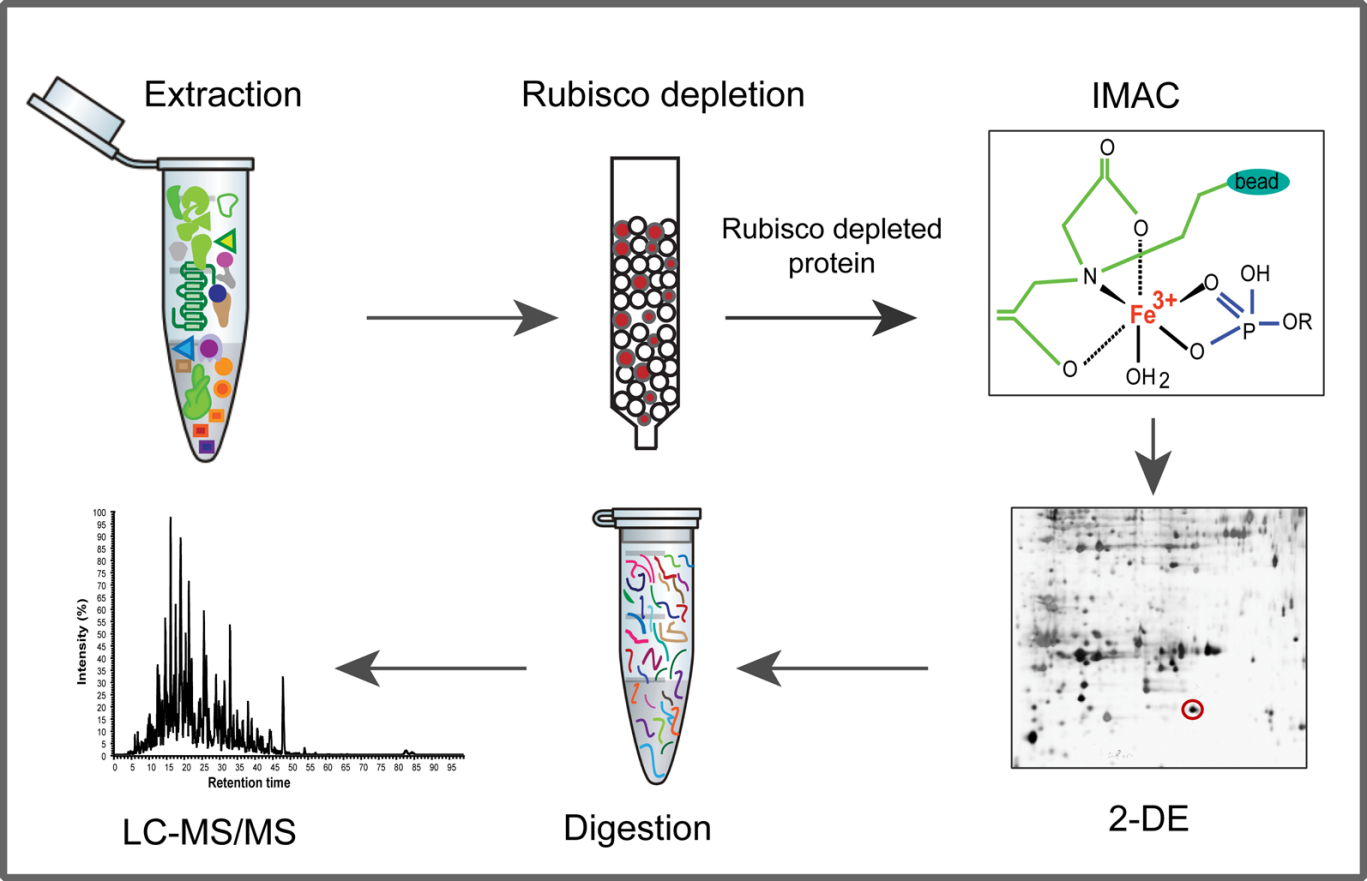
Plant phosphoproteome analysis using Rubisco depletion, IMAC enrichment of phosphoproteins, 2-DE and liquid chromatography-tandem mass spectrometry.

**Fig 2:**
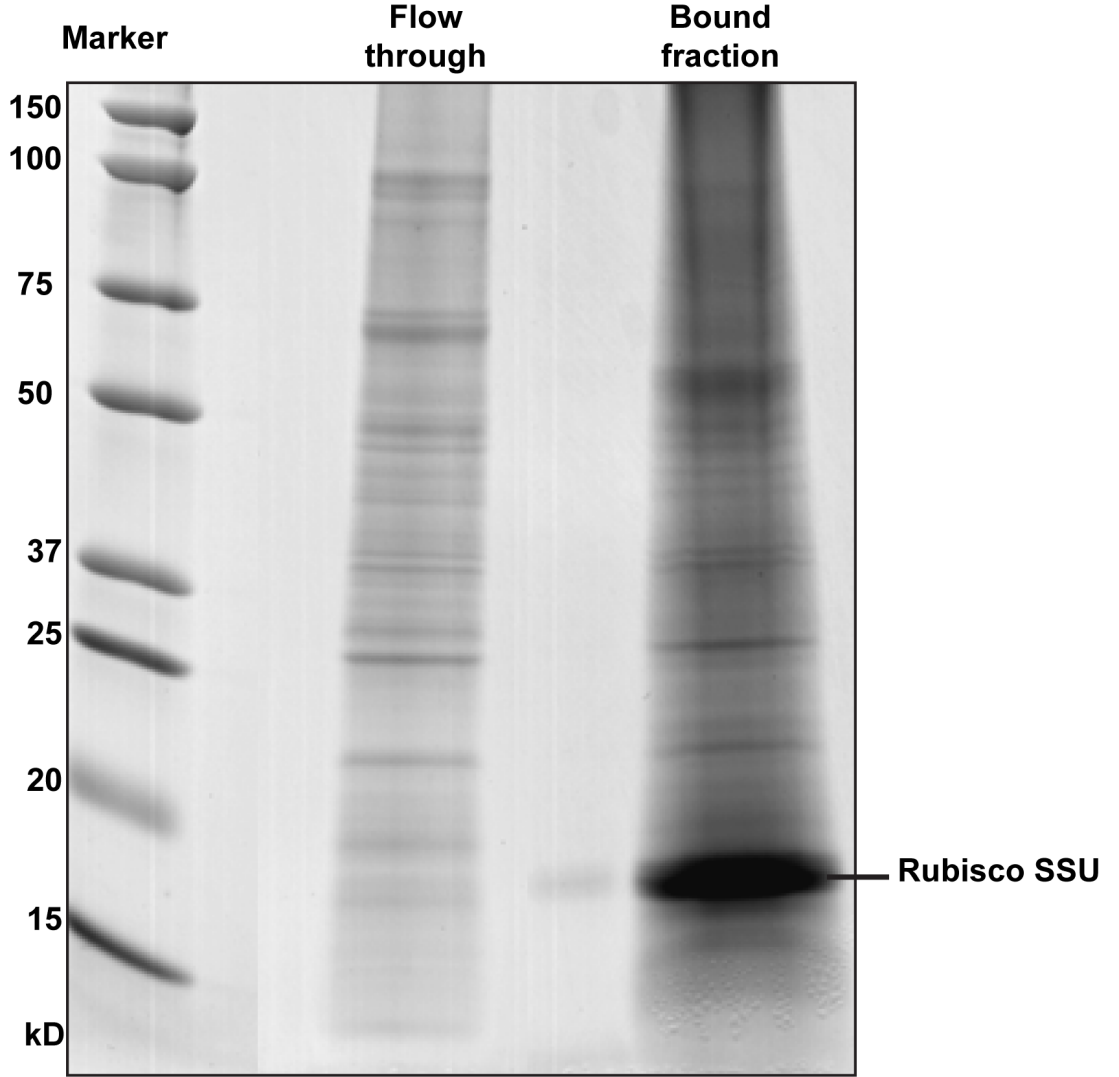
One-dimensional gel electrophoresis of the flow-through and bound protein fractions (10 μg) obtained following depletion of plant protein extracts using Seppro Rubisco IgY spin columns. Molecular weight markers (M) are shown on the left.

A schematic representation of our analytical approach is shown in Fig 1. The molecular weight distributions of proteins in the bound and flow-through samples following Rubisco depletion were investigated by 1-DE (Fig 2). Results show that the Seppro IgY Rubisco-depletion columns are efficient at removing Rubisco from the protein extracts of young seedlings. The Rubisco protein concentrated in the bound fraction is predominantly the small subunit (SSU), whereas both small and large subunits of Rubisco were evident in a previous study of mature *Arabidopsis* leaves.[11] That study also found that Rubisco depletion significantly increased the number of identified phosphoproteins, even without IMAC enrichment, and that only Rubisco and other relatively abundant phosphoproteins were recovered from non-depleted extracts using IMAC, whereas IMAC enrichment more than doubled the number of phosphoproteins identified in depleted extracts. It has recently been demonstrated that the Rubisco SSU up-regulates expression of the Rubisco large subunit (LSU) at the transcriptional level. This coordinated expression of subunits may explain the relatively small amount of Rubisco LSU observed during early growth in young seedlings.[26]

**Fig 3:**
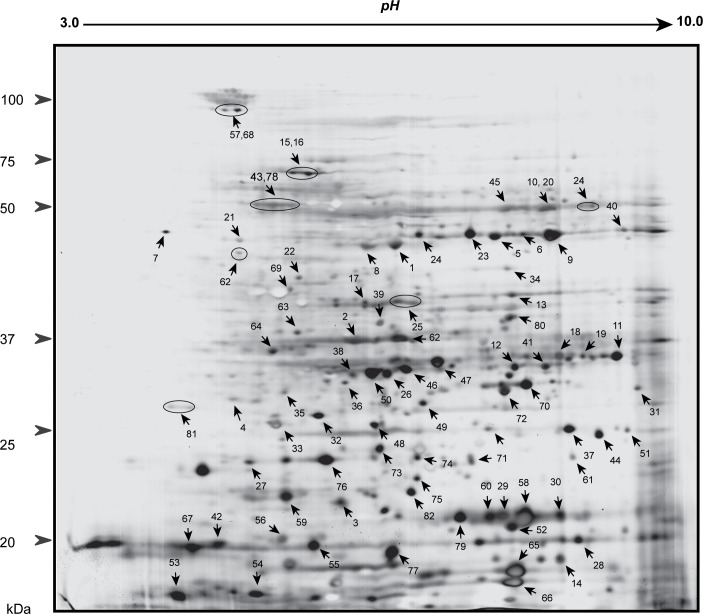
Two-dimensional gel electrophoresis of Rubisco-depleted phosphoproteins enriched by immobilized metal-ion affinity chromatography using PHOS-Select iron affinity gel beads. Phosphoproteins identified by liquid chromatography-tandem mass spectrometry are indicated using arrows and numbers (see Table 1).

IMAC-purified phosphoproteins from the Rubisco-depleted flow-through fraction were subsequently resolved by 2-DE (Fig 3). The reproducibility of both 1- and 2-DE experiments was confirmed by analyzing and comparing three biological replicates (not shown). An average of 175 protein spots were detected in replicate 2-DE gels following IMAC enrichment of Rubisco-depleted extracts. These were excised, trypsinized and analyzed by LC-MS/MS, which identified 156 of the spots based on our acceptance criteria for protein identification (see above). Of these, 105 spots (i.e. 60% of the 175 detected following IMAC) were found to contain a total of 82 different phosphoproteins based on the detection of 144 tryptic phosphopeptides, not counting methionine-oxidized and non-oxidized forms of the same peptide (Table 1, S3 Fig). The spot in which each phosphoprotein had been identified with highest confidence was subsequently labeled on a representative 2-DE gel image (Fig 3). Although significantly depleted in the flow-through fraction (Fig 2) Rubisco SSU was still detectable in 2-DE gels (Fig 3, spot 65).

**Table 1:** List of identified phosphoproteins.

Spot No.*	Gene locus	Protein name	MW/pI	Functional group	Phosphopeptide ^(a)^	*p*site
1	gi|15233613	O-acetylserine (thiol) lyase (OASA1)	33.9/5.9	Amino acid biosynthesis	DV**pT**ELIGNTPLVYLNNVAEGCVGR	T10
2	gi|18404496	Catalytic co-enzyme binding protein ^(b)^	35.8/8.4	Amino acid biosynthesis	ALDLA**pS**KPEGTGTPTK ^(c)^	S302
3	gi|15218373	Cystidine/deoxycystidylate deaminase ^(b,d)^	20.4/5.6	Amino acid biosynthesis	Y**pT**DPTAHAEVTAIR	T75
4	gi|15235213	Caffeoyl-CoA 3–0 methyltransferase	29.3/5.1	Amino acid biosynthesis	TS**pS**TNGEDQKQSQNLR **pT**SSTNGEDQKQSQNLR	S13 T11
5	gi|30691732	Aminoacylase, putative	48.0/5.9	Amino acid biosynthesis	T**pS**KPEIFPASTDAR	T387
6	gi|15224470	Pyridoxin biosynthesis protein PDX1.1 ^(b)^	33.1/5.8	Amino acid biosynthesis	**pT**KGEAGTGNVVEAVR	T165
7	gi|15233161	Peroxiglycinamidine cycloligase	41.6/5.3	Amino acid biosynthesis	GLAHI**pT**GGGFTDNIPR	T296
8	gi|42573371	Carbonic Anhydrase 2 (CA2) ^(b,d)^	28.7/5.4	Cellular metabolism	GNE**pS**YEDAIEALKK ^(e)^ KI**pT**AELQAASSSDSK ^(e,f)^ VCP**pS**HVLDFHPGDAFVVR VLAE**pS**ESSAFEDQCGR ^(e,f)^	S5 T35 S98 S191
9	gi|7769871	NAD malate dehydrogenase, mitochondrial ^(b)^	37.2/8.5	Cellular metabolism	KLFGV**pT**TLDVVR RTQDGG**pT**EVVEAK KPGM**pT**RDDLFNINAGIVK YCPHALINMI**pS**NPVNSTVPIAAEIFK LNPLVSSLSL**pY**DIANTPGVAADVGHINTR LNPLVSSL**pS**LYDIANTPGVAADVGHINTR NGVEEVLDLGPL**pS**DFEKEGLEALKPELK	T175 T251 T114 S146 Y61 S59 S325
10	gi|15219721	Malate dehydrogenase cytoplasmic 1 ^(b,d)^	35.9/6.1	Cellular metabolism	VQ**pT**SSGEKPVR ^(e,f)^	T203
11	gi|15226185	Fructose bisphosphate aldolase ^(b)^	42.5/8.2	Cellular metabolism	Y**pS**AEGENEDAKK	S372
12	gi|30678347	Carbonic anhydrase 1 chloroplast (CA1) ^(b)^	29.8/5.5	Cellular metabolism	VCP**pS**HVLDFQPGDAFVVR VI**pS**ELGDSAFEDQCGR	S98 S189
13	gi|15232468	Malate dehydrogenase (NAD), mitochondrial	36.0/8.3	Cellular metabolism	VVILGAAGGIGQPL**pS**LLMK	S46
14	gi|16398	Nucleotide diphosphate kinase ^(b)^	16.3/7.9	Cellular metabolism	NVIHG**pS**DSVESAR ^(e,f)^ IIGA**pT**NPAASEPGTIR KIIGA**pT**NPAASEPGTIR MEQ**pT**FIM*IKPDGVQR	S116 T90 T90 T4
15	gi|15230595	Phosphoglycerate kinase 1 (PGK1) ^(b)^	50.1/5.9	Cellular metabolism	VLPGVIALDEAIPV**pT**V	T480
16	gi|15220167	3-Isopropyl malate dehydrogenase 2 cytoplasmic	43.5/5.3	Cellular metabolism	ANPLA**pT**ILSAAM*LLK	T338
17	gi|15231715	Fructose bisphosphate aldolase (FBA), putative ^(b,d)^	38.8/6.1	Cellular metabolism	V**pS**PEVIAEHTVR **pT**VPAAVPAIVFLSGGQSEEEATR ^(c,f)^ S**pS**DGKLFVDILK	S239 T254 S84
					IGENEP**pS**EHSIHENNAYGLAR LGDGAAE**pS**LHVK ANSEA**pT**LGTYKGDAK GILAADES**pT**GTIGKR ^(c,f)^ LApSINVENVETNRR ALSDHHVLLEGTLLKPNM*V**pT**PG**pS**DSPK	S155 S350 T333 T33 S42 T230, S233
18	gi|15222848	Glyceraldehyde-3-phosphate dehydrogenase C-2 (GAPC-2) ^(d)^	37.0/6.7	Cellular metabolism	**pT**LLFGEKPVTVFGIR SDLDIV**pS**NASCTTNCLAPLAK ^(c,f)^	T70 S152
19	gi|15229231	Glyceraldehyde-3-phosphate dehydrogenase C subunit (GAPC), cytosolic ^(b,d)^	37.0/6.6	Cellular metabolism	**pT**LLFGEKPVTVFGIR	T70
20	gi|15218869	Isocitrate dehydrogenase (IDH) ^(b)^	46.1/6.1	Cellular metabolism	**pT**IEAEAAHGTVTR	T302
21	gi|15238559	Glutamine synthatase 2 (GS2), mitochondrial ^(b)^	47.8/6.4	Cellular metabolism	**pT**IEKPVEDPSELPK ^(c)^ GGNNILVICDTW**pT**PAGEPIP**pT**NK	T302 T154, T162
22	gi|15222551	Phosphoribulose kinase (PRK), chloroplastic	44.7/5.7	Cellular metabolism	HADFPG**pS**NNGTGLFQTIVGLK	S360
23	gi| 4539316	Fructose bisphosphate aldolase, putative	43.1/6.8	Cellular metabolism	RLDSIGLEN**pT**EANR	T91
24	gi|15226185	Fructose bisphosphate aldolase	42.5/8.2	Cellular metabolism	Y**pS**AEGENEDAKK	S372
25	gi|15227752	Peroxisomal malate dehydrogenase (PMDH1)	37.8/8.1	Cellular metabolism	KLMGV**pT**MLDVVR KPGM*pTRDDLFNINAGIVR	T138 T127
26	gi|15228198	PYK 10 binding protein 1(PBP1) ^(b,d)^	32.1/5.5	Cell defense	**pS**PEEVTGEEHGK Q**pT**SPPFGLEAGTVFELKEEGHK	S196 T254
27	gi|18405982	Avirulense-responsive protein	19.6/5.0	Cell defense	LHACI**pS**PSENGLINGK TVEVVL**pT**DTSEKK	S56 T97
28	gi|15236568	Major latex protein related/ MLP-related	17.5/5.9	Cell defense	EIDDE**pT**KTLTLR V**pY**DVVYQFIPK **pS**LVADMGNHVSK	T79 Y98 S139
29	gi|15223957	Major latex protein related/MLP related	18.0/6.4	Cell defense	FV**pT**SLAADMDDHILK	T138
30	gi|15236566	Major latex-related/ MLP-related	17.6/5.9	Cell defense	RNDDFPEP**pS**GYMK	S131
31	gi|1755154	Germin-like protein	22.0/6.8	Cell defense	AAV**pT**PAFAPAYAGINGLGVSLAR	T72
32	gi|15228199	Jacalin lectin family protein ^(b)^	32.2/5.9	Cell signaling	**pS**PEEVTGEEHGK ASELLHQFGVVM*PL**pT**N	S195 T299
33	gi|15228216	Jacalin lectin family protein	32.0/5.1	Cell signaling	**pT**SPPYGLETQKK KVHVGQGQDGV**pS**SINVVYAK	T104 S37
34	gi|15226403	Cupin family protein ^(b)^	55.9/5.8	Cell signaling	NRPQFLVG**pS**NSLLR **pT**GPFEFVGFTTSAHK G**pS**GSSECEDSYNIYDKK ^(e)^	S456 T433 S320
35	gi|18418598	Cyclase family protein	30.0/5.6	Cell signaling	AGL**pY**SVHCLPLR	Y249
36	gi|15241018	*A*. *thaliana* Ferretin 1 (ATFER1), chloroplastic ^(b)^	28.1/5.7	Electron transport	ADLAIPI**pT**SHASLAR ^(c)^	T80
37	gi|9843639	Rieske FeS protein	24.6/8.8	Electron transport	GPAPL**pS**LALAHADIDEAGK	S196
					GDP**pT**YLVVENDK FLCPCHG**pS**QYNAQGR	T138 S180
38	gi|15231176	ATP synthase D chain, mitochondrial (ATPQ)	19.6/5.1	Membrane transport	V**pT**PEYKPK	T96
39	gi|7525040	ATP synthase CF1 beta subunit	53.9/5.4	Membrane transport	IVGEEHYE**pT**AQQVK	T387
40	gi|15231008	Translocase of outer mitochondrial membrane 40	34.2/6.3	Membrane transport	GKID**pS**NGVASALLEER	S269
41	gi|1143394	V-type proton ATPase	26.2/6.0	Membrane transport	IDY**pS**MQLNASR **pS**NDPHGLHCSGGVVLASR	S71 S178
42	gi|15236722	ATP synthase family ^(b)^	23.9/5.8	Membrane transport	ALD**pS**QIAALSEDIVKK ^(e)^	S203
43	gi|7708276	ATP synthase beta subunit	52.5/5.2	Membrane transport	INP**pTpTpS**GSGVMTLEK	T5, T6, S7
44	gi|15227104	putative ATP synthase subunit	27.6/6.3	Membrane transport	EKI**pT**LDPEDPAAVK	T71
45	gi|15236678	Ascorbate peroxidase 4 (APX4), chloroplastic	38.1/8.6	Oxidative stress	AENEGL**pS**DGLSLIEEVKK	S155
46	gi|15223576	Dehydroascorbate reductase 1 (DHAR1)	23.4/5.6	Oxidative stress	**pT**PAEFASVGSNIFGTFGTFLK	T91
47	gi|15224582	Glutathione S-transferase 10 (ATGSTF10)	24.2/5.5	Oxidative stress	VL**pT**IYAPLFASSK	T4
48	gi|15224581	Glutathione S-transferase 9 (ATGSTF9) ^(b,d)^	24.1/5.5	Oxidative stress	QPAYLALQPFG**pT**VPAVVDGDYK LAGVLDV**pY**EAHLSK	T52 Y146
49	gi|15218640	Glutathione S-transferase 6 (ATGSTF6)	23.5/5.8	Oxidative stress	VFGHPASTA**pT**R	T15
50	gi|15226610	ATPDIL2-1/MEE30/UNE5 (PDI) ^(b)^	39.8/5.8	Oxidative stress	G**pS**DYASKETER ELVAA**pS**EDEKK AGHDYDGGRDLDDFV**pS**FINEK	S321 S280 S243
51	gi|15231718	Peroxiredoxin type 2, chloroplastic	24.7/9.1	Oxidative stress	**pT**ILFAVPGAFTPTCSQK VLNLEEGGAF**pT**NSSAEDMLK VLNLEEGGAF**pT**NSSAEDM*LK	T108 T223 T223
52	gi|30693971	Universal stress protein family protein	17.9/5.7	Oxidative stress	DLKLD**pS**IVMGSR	S125
53	gi|15232567	*A*. *thaliana* thioredoxin M-type 4 (ATHM4), chloroplastic	21.3/9.6	Oxidative stress	IN**pT**DESPNTANR D**pS**IIGAVPRETLEK	T144 S173
54	gi|6539610	Thioredoxin M2, chloroplastic ^(a)^	20.6/9.4	Oxidative stress	TTL**pT**SSLDKFLP LN**pT**DESPNTPGQYGVR	T178 T138
55	gi|3121825	2-Cys peroxiredoxin, chloroplast precursor	29.0/7.7	Oxidative stress	**pS**GGLGDLNYPLISDVTK	S161
56	gi|15223049	Ascorbate peroxidase 1 (APX1), cytosolic	27.8/5.7	Oxidative stress	QM*GL**pS**DKDIVALSGAHTLGR	S152
57	gi|15219086	Protein disulfide isomerase (PDI)-like protein ^(b,d)^	55.8/4.8	Oxidative stress	**pS**ADDASEVVSDKK ^(c)^	S149
58	gi|20197312	Glutathione S-transferase 6 (GST6) ^(b)^	24.1/6.1	Oxidative stress	AI**pT**QYLAEEYSEKGEK	T72
59	gi|18415155	2-Cys peroxiredoxin, chloroplastic	29.9/5.6	Oxidative stress	**pS**FGVLIPDQGIALR **pS**GGLGDLNYPLVSDITK	S189 S168
60	gi|15228407	Superoxide dismutase 1 (MSD1), mitochondrial ^(b)^	25.5/8.5	Oxidative stress	YApSEVYEKENN	S223
61	gi|7658343	Peroxiredoxin IIF ^(b)^	21.3/9.0	Oxidative stress	LAEG**pT**DITSAAPGVSLQK **pS**LGLDKDLSAALLGPR	T35 S147
62	gi|15228194	Sedoheptulose-1,7-bisphosphatase, chloroplastic ^(b,d)^	42.7/6.2	Photosynthesis and respiration	GFPG**pT**HEELLLDEGK	T235
63	gi|15229349	Ribose 5-phosphate isomerase-related ^(b,d)^	29.4/5.7	Photosynthesis and respiration	LL**pS**SGELYDIVGIPTSK **pS**LGIPLVGLDTHPR ^(e,g)^ LQDLFKEFGCE**pS**K	S86 S108 S206
64	gi|414550	Cytosolic triosephosphate isomerase (TPI) ^(b)^	27.4/5.2	Photosynthesis and respiration	AILNE**pS**SEFVGDKVAYALAQGLK VA**pS**PAQAQEVHDELRK	S106 S178
65	gi|13926229	Rubisco small chain 1A (RBCS1A) ^(b)^	14.9/5.7	Photosynthesis and respiration	EHGN**pT**PGYYDGR ^(e)^ KFE**pT**LSYLPDLTDSELAK ^(f)^ LPLFGC**pT**DSAQVLK	S58 T14 T78
66	gi|16194	Rubisco small subunit (RbcS)	20.6/7.6	Photosynthesis and respiration	EHGN**pT**PGYYDGR KFE**pT**LSYLPDLSDVELAK ^(f)^ FE**pT**LSYLPDLSDVELAK LPLFGC**pT**DSAQVLK	T113 T69 T69 T133
67	gi|15223217	Glycine cleavage system H protein, mitochondrial^(b)^	18.0/5.1	Photosynthesis and respiration	VKP**pS**SPAELEALMGPK VKP**pS**SPAELEALM*GPK	S141 S141
68	gi|84468442	Putative Rubisco subunit binding protein ^(b)^	48.6/4.8	Photosynthesis and respiration	HEAAGDG**pT**TTASILAR	T8
69	gi|13926291	PS II oxygen-evolving complex 1 (PSBO1) ^(b)^	35.3/5.6	Photosynthesis and respiration	QLDA**pS**GKPDSFTGK	S221
70	gi|20260472	Glyoxylate reductase ^(b)^	36.7/8.5	Photosynthesis and respiration	**pS**KCDPLVGLGAK **pS**YGLSDEDFSAVIEALK	S85 S320
71	gi|18416540	CIP amino terminal domain containing protein	26.1/9.2	Protein degradation	**pS**MNEDVDLSFKK	S224
72	gi|18390982	ATP dependent Clp protease proteolytic subunit (CLPP), chloroplastic	36.4/8.6	Protein degradation	**pS**VAYNEHRPR VPS**pS**GLM*PASDVLIR	S107 S241
73	gi|15224993	20S proteasome subunit PAA2	27.4/5.8	Protein degradation	LLDQ**pS**SVSHLFPVTK A**pT**SAGMKEQEAVNFLEK	S64 T166
74	gi|2511588	Multicatalytic endopeptidase complex ^(b)^	27.2/5.6	Protein degradation	LLDQ**pS**SVTHLFPITK	S63
75	gi|15219317	20S Proteasome alpha subunit B, putative ^(b)^	25.7/5.8	Protein degradation	KLP**pS**ILVDEASVQK L**pY**KEPIPVTQLVR Y**pT**EDMELDDAIHTAILTLK RYTEDM*ELDDAIH**pT**AILTLK	S54 Y101 T179 T194
76	gi|15242045	Chaperonin 20 (CPN20), chloroplastic ^(b,d)^	26.8/8.9	Protein folding	Y**pT**SIKPLGDR **pY**TSIKPLGDR YT**pS**IKPLGDR **pT**LGGILLPSTAQSKPQGGEVVAVGEGR	T60 Y59 S61 T80
77	gi|15226314	Chaperonin HSP 60A (CPN60A) ^(b,d)^	62.2/5.1	Protein folding	I**pT**AIKDIIPILEK	T272
78	gi|16221	Chaperonin HSP60	61.6/5.7	Protein folding	V**pT**KDGVTVAK	T80
79	gi|62321455	Putative cruciferin 12S seed storage protein	19.9/7.9	Seed storage	GLPLEVI**pT**NGYQISPEEAK VFDQEI**pS**SGQLLVVPQGFSVM*K	T143 S89
80	gi|166678	12S storage protein	50.9/6.8	Seed storage	VFDQEI**pS**SGQLLVVPQGFSVM*K GLPLEVI**pT**NGYQISPEEAKR	S366 T420
					**pT**NENAQVNTLAGR	T395
81	gi|9758672	Unnamed protein product	29.0/5.9	Unclassified	VPELVAK**pT**ELENIAK	T149
82	gi|18391006	Unknown protein ^(b,d)^	20.0/5.4	Unclassified	EI**pS**MPNGLLPLK	S33

Phosphorylated proteins, peptides and residues (phosphosites) identified by mass spectrometry in protein extracts from young *Arabidopsis* seedlings after rubisco depletion, IMAC enrichment and 2-DE separation of phosphoproteins. (a) **pS**, **pT** and **pY** = phosphorylated serine, threonine and tyrosine residues; M* = oxidized methionine. (b) Protein reported in the PhosPhAt 4.0 database. ^30,31^ (c) Peptide reported in the PhoPhAt 4.0 database with a different protein phosphorylation site. ^30 31^ (d) Protein previously reported in *Arabidopsis* seedlings. ^23^ (e) Peptide reported in the PhosPhAt 4.0 database with the same protein phosphorylation site. ^30, 31^ (f) Peptide previously reported in *Arabidopsis* seedlings with a different protein phosphorylation site. ^23^ (g) Peptide previously reported in *Arabidopsis* seedlings with the same protein phosphorylation site. ^23^

**Fig 4:**
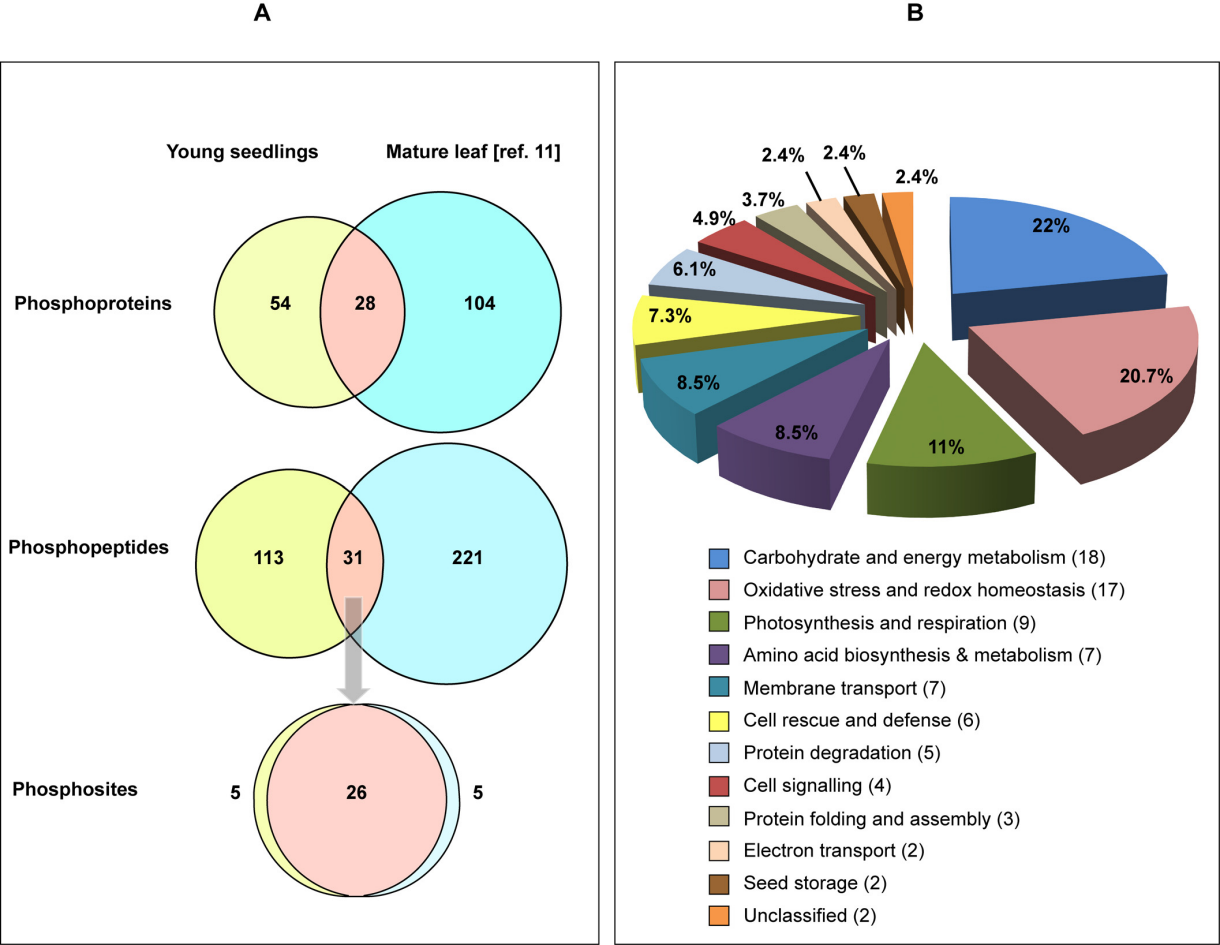
Distribution and functional classification of identified phosphoproteins. (A) Numbers of phosphoproteins and phosphopeptides identified in post-embryonic *Arabidopsis* seedlings and mature *Arabidopsis* leaves,[11] and of the phosphosites identified in phosphopeptides common to both tissues. (B) Functional classification of the phosphoproteins identified in *Arabidopsis* young seedlings according to the KEGG Pathway database (http://www.genome.jp/kegg/pathway.html). Proteins involved in carbohydrate/energy metabolism, oxidative stress/redox regulation and photosynthesis/respiration account for over 50% of the identified phosphoproteome.

The 144 detected phosphopeptides contained a total of 144 unique sites of protein phosphorylation, of which 48% (69) were serine, 48% (69) were threonine, and 4% (6) were tyrosine residues (Table 2; Fig 4A). To assess any differences in phosphorylation occupancy among the S, T and Y residues, we compared our results with those from previous studies that utilized different enrichment methods and plant tissues (Table 2). The distribution observed in this study for *Arabidopsis* seedlings is similar to that obtained using Rubisco depletion and IMAC enrichment of intact phosphoproteins from mature *Arabidopsis* leaves,[11] which contained 52% phosphoserine (pS), 40% phosphothreonine (pT), and 8% phosphotyrosine (pY) residues (Table 2). However, these results differ from those obtained using IMAC to enrich phosphopeptides generated by trypsin digestion of plant phosphoproteins. For example, previous results reported 88% pS, 11% pT and 1% pY in 22-day-old *Arabidopsis* seedlings [22]; 85% pS, 13% pT and 2% pY in 9-day-old *Arabidopsis* seedlings [23]; 85% pS, 11% pT and 4% pY in cultured *Arabidopsis* cells [21]; 86% pS, 13% pT and 1% pY in *Medicago truncatula* roots [26]; and 81% pS, 17% pT, and 2% pY in dormant poplar (*Populus simonii* × *P*. *nigra)* buds [27] when IMAC enrichment was performed at the phosphopeptide level. Rao and Moller [28] reported the occurrence of 77% pS, 17.5% pT and 5.5% pY in eukaryotic phosphoproteins based on a combined Uniprot, Phospho.ELM and Phosida database analysis, which also differs from the present study. By way of comparison, the average pS:pT:pY ratio observed for cellular phosphoproteins in mammals is approximately 1800:200:1,[29] corresponding to 89.95% pS, 10.00% pT and 0.05% pY. These results suggest that peptide- and protein-level enrichment strategies complement each other to some extent and that the latter provides access to a greater proportion of phosphorylated threonine and tyrosine residues, at least in plant phosphoproteins.

**Table 2:** Distribution of phosphorylated residues identified in plant proteins using immobilized metal-ion affinity chromatography of phosphorylated proteins or peptides.

Plant species	Tissue	IMAC target	% pS	% pT	% pY	References
*Arabidopsis thaliana*	seedlings	pProteins	48	48	4	This study
*Arabidopsis thaliana*	leaves	pProteins	52	40	8	11
*Arabidopsis thaliana*	cultured cells	pPeptides	85	11	4	21
*Arabidopsis thaliana*	seedlings	pPeptides	88	11	1	22
*Arabidopsis thaliana*	seedlings	pPeptides	85	13	2	23
*Medicago truncatula*	roots	pPeptides	86	13	1	25
*Poplar simonii × P*. *nigra*	dormant buds	pPeptides	81	17	1	26

These findings are of particular significance given the emerging importance of tyrosine phosphorylation in plant processes such as germination, growth, development, and abiotic stress responses.[30] In particular, our discovery of 6 new tyrosine phosphorylation sites (Table 1) in proteins involved in the mobilization of seed reserves (NAD^+^ MDH), cell defence (MLP), cellular signaling (cyclase family protein), oxidative stress response (GST9), protein degradation (20S proteasome alpha subunit B) and protein folding (chaperonin 20) represents a significant contribution to the list of potential substrates for known and predicted protein tyrosine kinases in plants.[30] It also helps to address the apparent discrepancy between the predicted frequency of pY residues in the Arabidopsis proteome [13] and that observed using peptide-level affinity enrichment strategies, during which the phosphorylated residues in each protein are distributed between tryptic peptides containing only one or two such residues, of which those carrying the more abundant pS modification are likely to predominate in terms of recovery and analysis.

Of the 144 phosphopeptides and 144 phosphosites reported in the present study, only 10 peptides and 1 phosphorylation site matched those identified during a recent survey of the phosphoproteome in hydroponically-grown *Arabidopsis* seedlings, which utilized Ti^4+^-IMAC enrichment of tryptic phosphopeptides from whole protein digests (Table 1).[23] Of those 144 phosphopeptides, 10 phosphopeptides and 5 phosphorylation sites were found in both the P3DB (http://www.p3db.org/) and PhosPhAt 4.0 (http://phosphat.uni-hohenheim.de/) databases, with an additional 8 peptides and 3 phosphorylation sites found only in the PhosPhAt 4.0 database.

Of the identified phosphoproteins previously reported in *Arabidopsis thaliana* seedlings [23] (Table 1) two are isoforms of the same protein, GAPC (spots 18 and 19). Detection of the novel phosphopeptide **pT**LLFGEKPVTVFGIR in both isoforms indicates that both are phosphorylated at T70. However, a second phosphopeptide SDLDIV**pS**NASCTTNCLAPLAK, which had previously been detected in *Arabidopsis* seedlings [23] (though with a different site of phosphorylation), was also identified in one of the isoforms (spot 18) indicating phosphorylation at S152 (Table 1). The concomitant reduction in pI relative to the other isoform (spot 19) is consistent with horizontal separation of these two proteins on the 2-DE gel (Fig 3), demonstrating the utility of our gel-based approach for resolving differentially phosphorylated forms of a given protein. Similarly, vertical separation of two Rubisco polypeptides (Fig 3, spots 65 and 66) reflects the difference in molecular weight between the matched proteins, each of which contained the same number (3) of identified phosphorylation sites (Table 1).

IMAC purification, 2-DE separation, and digestion of intact phosphoproteins to produce a mixture of phosphorylated and non-phosphorylated peptides may have contributed to the relatively small number of multiply-phosphorylated peptides identified during this study, compared with studies in which only phosphorylated peptides were enriched and analyzed by mass spectrometry. [16–18,21,26,27,31] However, the average number of phosphopeptides identified per plant protein (1.8 in young *Arabidopsis* seedlings and 1.9 in mature leaves [11]) compares well with studies that utilize peptide-level enrichment [27]. Furthermore, the phosphoproteins we identified in young seedlings using protein-level enrichment include basic proteins (e.g. APX1, APX4, nucleotide diphosphate kinase) and proteins previously identified as plasma membrane proteins (e.g. CA2, PGK, DHAR1) in *Arabidopsis* seedlings,[12,16,32] suggesting minimal bias towards proteins of a particular polarity, pI or molecular weight.[11] By enabling protein identification using both phosphorylated and non-phosphorylated peptides our approach also provides high confidence in the identification of phosphoproteins and hence, their selection as candidates for further investigation of the role of protein phosphorylation during plant development (which lies beyond the scope of the present study).

### Functional classification of phosphoproteins

The identified phosphoproteins were sorted into functional groups using the KEGG Pathway database (http://www.genome.jp/kegg/pathway.html). The two largest groups were those involved in carbohydrate/energy metabolism (22%) and oxidative stress/redox regulation (20%), which together with photosynthesis and respiration (11%) accounted for more than half of the identified phosphoproteins (Fig 4). Many of these, including glyceraldehyde-3-phosphate dehydrogenase (GAPC-2), triosephosphate isomerase (TPI), phosphoglycerate kinase (PGK1), fructose bisphosphate aldolase (FBA), and malate dehydrogenase (MDH), play important role in processes such as glycolysis, gluconeogenesis and the Calvin cycle during seed germination and the early stages of seedling establishment. Identification of phosphorylated 20S proteasome subunits, proteases, chaperonins, thioredoxins, glutathione transferases (GSTs), dehydroascorbate reductase (DHAR1) and manganese superoxide dismutase (MSD1) is also consistent with the role of proteolytic events in mobilizing TAG and other seed reserves. Comparison of our experimental results with the PhosPhAt 4.0 *Arabidopsis thaliana* phosphorylation site database [33,34], P3DB database [35] and with supplementary information from a recently published survey of the *Aradopsis* seedling phosphoproteome [23] showed that 43 of the 82 phosphoproteins identified in our study have not been reported before (Table 1), and that we were able to identify new phosphorylation sites in previously characterized phosphoproteins such as FBA, GAPC-2, TPI and PMDH1 (S1 Fig), GSTs (S2A Fig and S2B Fig), PRK and IDH. New and known phosphorylation sites were also identified in 12S seed storage proteins (Table 1), further demonstrating the utility of our approach for identifying novel sites of protein phosphorylation in plant tissues.

## Discussion

### Phosphorylation of enzymes involved in post-embryonic development

Many of the enzymes known to be important during the early stages of plant growth were found to be phosphorylated in *Arabidopsis* young seedlings. The glycolytic enzyme triosephosphate isomerase (TPI), for example, plays a central role in chloroplast development [36] and other biochemical pathways by equilibrating the cytosolic pool of DHAP and G-3-P. The latter is required for 1,5-bisphosphate production in the Calvin cycle, whereas DHAP suppresses the production of chlorophyll and 1,5-bisphosphate. Phosphorylation of human TPI has been shown to reduce its activity in converting G-3-P to DHAP, and although it has been suggested that TPI can be phosphorylated at S21 there is evidence that other sites may be subject to phosphorylation.[37] Our discovery of phosphorylated S106 and S178 residues in *Arabidopsis* TPI (Table 1, spot 64) provides new information with which to investigate the role of protein phosphorylation in controlling the activity of this enzyme and thus regulating chloroplast development in young seedlings.

NAD^+^ MDH, a key enzyme in carbohydrate metabolism, is responsible for regenerating NAD^+^ and is involved in the mobilization of seed oil reserves [4] and the photosynthetic assimilation of carbon in developing leaves. [38] We identified several sites of phosphorylation in mitochondrial NAD^+^ MDH (Table 1, spot 9), as well as single site of phosphorylation in cytosolic MDH (Table 1, spot 10). We also observed phosphorylation of 3-isopropyl malate dehydrogenase (spot 16), which is primarily involved in leucine biosynthesis. [39]

**Fig 5:**
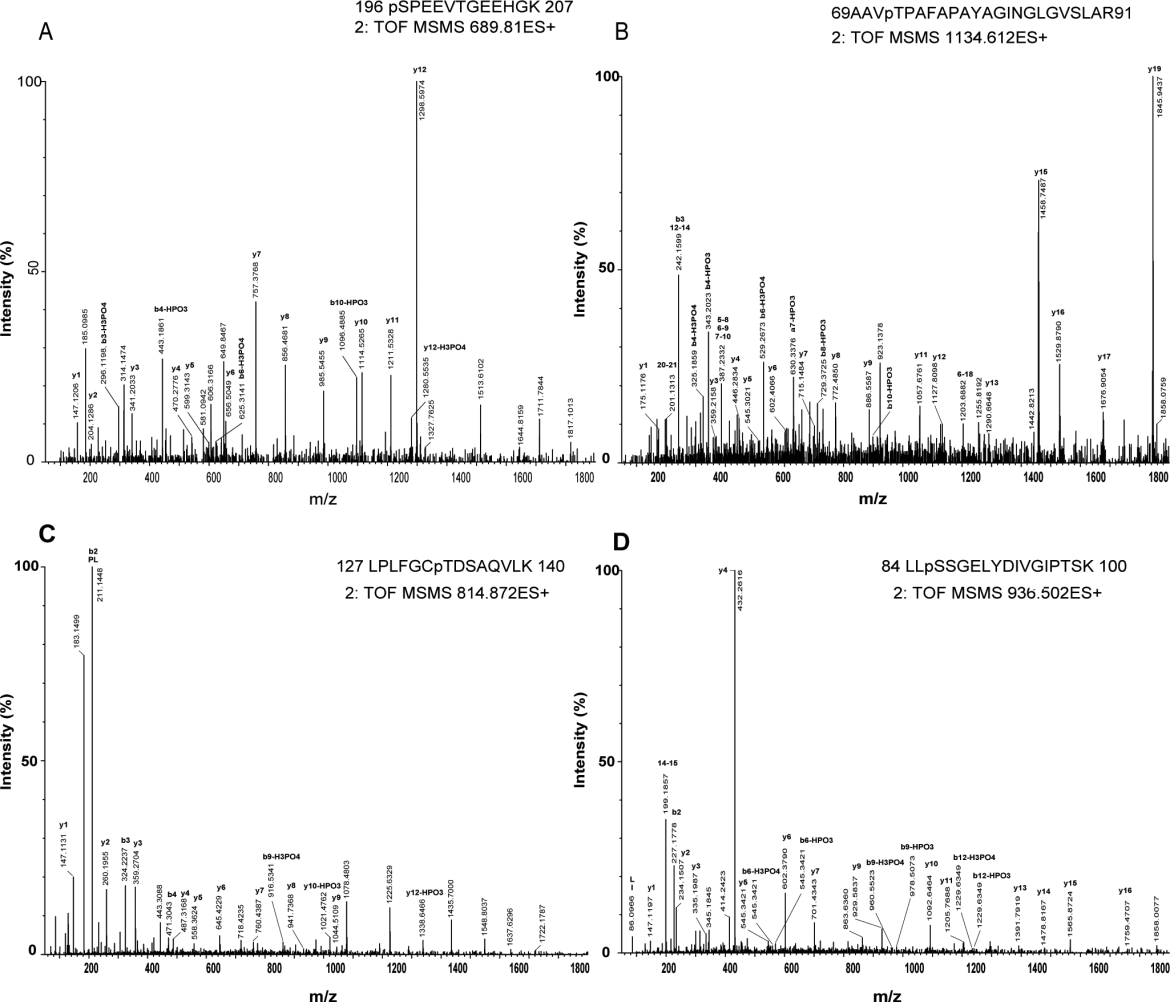
Identification of phosphorylation sites using tandem mass spectrometry (MS/MS). The MS/MS spectra correspond to phosphopeptides with the following mass-to-charge (*m/z*) ratios, as obtained by trypsin digestion of proteins selected from the 2-DE gel shown in Fig 3 (A) *m/z* 689.810, showing phosphorylation of jacalin-lectin family protein (spot 32) at S195; (B) *m/z* 1134.612, showing phosphorylation of germin-like protein (spot 31) at T72; (C) *m/z* 814.872, showing phosphorylation of the Rubisco small subunit (spot 66) at T133; (D) *m/z* 936.502, showing phosphorylation of ribose 5-phosphate isomerase-related protein (spot 63) at S86. Peaks corresponding to sequential loss of intact amino acid residues from the C or N terminus of the peptide are labeled as b- or y-type ions, respectively.

Carbonic anhydrase (CA), a major chloroplast protein, is involved in photosynthesis [40] and the mobilization of seed reserves during the early stages of post-embryonic growth. CA1 is also known to form part of a Rubisco-containing Calvin cycle enzyme complex.[40] Identification of phosphorylation sites in CA (spots 8 and 12), ribose-5-phosphate isomerase (spot 63), Rubisco SSU (spot 66) and PRK (spot 22) may help to elucidate the role of protein phosphorylation in controlling the assimilation and utilization of carbon reserves during the early stages of seedling establishment.[41] Other identified phosphoproteins include members of the jacalin-lectin (Fig 5A), cupin, and cyclase families (spots 32 to 34), all of which are involved in cell signaling. A cupin domain protein (AtPirin1) has also been found to interact with G protein α-subunit GPA1 in *Arabidopsis* to regulate seed germination and seedling development.[42]

Phosphorylation of 20S proteasome subunit PtrPBA1, and increased expression of 20S proteasome α-subunit B and regulatory subunit RPN10, have been observed in poplar dormant terminal buds.[27] We observed phosphorylation of 20S proteasome α-subunit B (spot 75) at S54, T179, T194 and Y101 and of the 20S proteasome subunit PAA2 (spot 73) at S64 and T166 in *Arabidopsis* young seedlings (Table 1). ATP dependent Clp protease proteolytic subunit (CLPP) is a highly conserved, multimeric serine protease [43] that degrades large globular proteins in the presence of an AAA ATPase complex. [44] CLPP (spot 72) was found to be phosphorylated at S107 and S241, and a Clp amino terminal domain-containing protein (spot 71) at S224.

Although there is growing evidence of crosstalk between redox signaling and hormonal response pathways during seed germination,[45] the molecular components involved in this process during post-embryonic development remain elusive. We identified phosphorylated forms of several proteins known to be key regulators of stress response, including APX1 (spot 56), APX4 (spot 45), GST6 (spot 58), ATGSTF9, -10 and -6 (spots 47 to 49 and S2A Fig and S2B Fig), dehydroascorbate reductase 1 (spot 46), thioredoxins M2 and M4 (spots 53 and 54), peroxiredoxins (spots 51 and 59), and manganese superoxide dismutase (spot 60). Phosphorylation of APX1, APX4, peroxiredoxin type-2, GST6, and MSD1 was also observed in mature leaves [11] but at sites other than those observed in young seedlings (Table 3). Thioredoxins and other H_2_O_2_-scavenging enzymes help to protect plants from damage caused by the production of reactive oxygen species (ROS) during seed germination and seedling development.[46] Germin-like protein, which generates H_2_O_2_ from the oxidative breakdown of oxalate,[47] was also found to be phosphorylated in our study (Fig 5B).

**Table 3:** Changes in protein phosphorylation between post-embryonic seedlings and mature leaves. Phosphorylated proteins, peptides and residues (S = serine, T = threonine, Y = tyrosine) identified in post-embryonic seedlings and mature leaves of *Arabidopsis thaliana*. Common phosphopeptides with conserved phosphosites are highlighted in bold and common phosphopeptides with different phosphosites are highlighted in bold and italics.

Gene locus	Protein name	Young seedlings	Mature leaves	Phospho-site(s)
**gi|18404496**	Catalytic co-enzyme binding	**ALDLApSKPEGTGTPTK**	**ALDLApSKPEGTGTPTK**	**S302**
**gi|15218869**	Isocitrate dehydrogenase	pTIEAEAAHGTVTR	-	T302
		-	LVPGWpTKPICIGR	T127
**gi|15219721**	Malate dehydrogenase (MDH)	VQpTSSGEKPVR	-	T203
		-	LSpSALSAASSACDHIR	S243
			NVIIWGNHpSSSQYPDVNHAK	S189
**gi|15222848**	G3P cytosolic-2 (GAPC-2)	**pTLLFGEKPVTVFGIR**	**pTLLFGEKPVTVFGIR**	**T70**
		-	FGIVEGLMTpTVHSITATQK	T181
		SDLDIVpSNASCTTNCLAPLAK	-	S152
**gi|15231715**	Fructose bisphosphate aldolase	**pTVPAAVPAIVFLSGGQSEEEATR**	**pTVPAAVPAIVFLSGGQSEEEATR**	**T254**
		**IGENEPpSEHSIHENNAYGLAR**	**IGENEPpSEHSIHENNAYGLAR**	**S155**
		**LGDGAAEpSLHVK**	**LGDGAAEpSLHVK**	**S350**
		**VpSPEVIAEHTVR**	**VpSPEVIAEHTVR**	**S239**
		ANSEApTLGTYKGDAK	-	T333
		GILAADESpTGTIGKR	-	T33
		LApSINVENVETNRR	-	S42
		pSSDGKLFVDILK	-	S83
		ALSDHHVLLEGTLLKPNM*VpTPGpSDSPK	-	T230,S233
**gi|15227752**	Malate dehydrogenase (PMDH1)	KLMGVpTMLDVVR		T138
		KPGM*pTRDDLFNINAGIVR	-	T127
		-	AIVNIIpSNPVNSTVPIAAEVFK	S159
**gi|15229231**	G3P cytosolic (GAPC)	**pTLLFGEKPVTVFGIR**	**pTLLFGEKPVTVFGIR**	**T70**
		-	FGIVEGLMTpTVHSITATQK	T181
**gi|16398**	Nucleotide diphosphate kinase	**NVIHGpSDSVESAR**	**NVIHGpSDSVESAR**	**S116**
		KIIGApTNPAASEPGTIR	-	T90
		IIGApTNPAASEPGTIR	-	T90
**gi|414550**	Cytosolic triose phosphate isomerase	**AILNEpSSEFVGDKVAYALAQGLK**	**AILNEpSSEFVGDK**	**S106**
		**VApSPAQAQEVHDELRK**	**VApSPAQAQEVHDELRK**	**S178**
		-	VIACVGEpTLEER	T131
**gi|42573371**	Carbonic Anhydrase 2 (CA2)	**KIpTAELQAASSSDSK**	**IpTAELQAASSSDSK**	**T35**
		**VCPpSHVLDFHPGDAFVVR**	**VCPpSHVLDFHPGDAFVVR**	**S98**
		***VLAEpSESSAFEDQCGR***	***VLAESEpSSAFEDQCGR***	***S191*,*S193***
		GNEpSYEDAIEALKK	-	S5
		-	EAVNVpSLANLLTYPFVR	S211
		-	pYAGVGAAIEYAVLHLK	Y126
**gi|7769871**	NAD-malate dehydrogenase	***RTQDGGpTEVVEAK***	***pTQDGGTEVVEAK***	***T251*, *T246***
		KPGMpTRDDLFNINAGIVK	-	T114
		LNPLVSSLpSLYDIANTPGVAADVGHINTR	-	S59
		LNPLVSSLSLpYDIANTPGVAADVGHINTR	-	Y61
		NGVEEVLDLGPLpSDFEKEGLEALKPELK	-	S325
		KLFGVpTTLDVVR	-	T175
		YCPHALINMIpSNPVNSTVPIAAEIFK	-	S146
**gi|15228198**	PYK 10 binding protein 1(PBP1)	**pSPEEVTGEEHGK**	**pSPEEVTGEEHGK**	**S196**
		***QpTSPPFGLEAGTVFELKEEGHK***	***QTpSPPFGLEAGTVFELK***	***T254*,*S255***
		-	GANLWDDGpSTHDAVTK	S20
		-	TpSDVIGSDEGTHFTLQVK	S102
		-	VpYVGQAQDGISAVK	Y178
**gi|1755154**	Germin-like protein	AAVpTPAFAPAYAGINGLGVSLAR	-	T72
		-	GDpSMVFPQGLLHFQLNSGK	S140
**gi|18405982**	Avirulense-responsive protein	LHACIpSPSENGLINGK	-	S56
		***TVEVVLpTDTSEKK***	***pTVEVVLTDTSEKK***	***T97*, *T91***
**gi|9843639**	Rieske FeS protein	**FLCPCHGpSQYNAQGR**	**FLCPCHGpSQYNAQGR**	**S180**
		**GPAPLpSLALAHADIDEAGK**	**GPAPLpSLALAHADIDEAGK**	**S196**
		GDPpTYLVVENDK	-	T138
**gi|1143394**	V-type proton ATPase	**IDYpSMQLNASR**	**IDYpSMQLNASR**	**S71**
		**pSNDPHGLHCSGGVVLASR**	**pSNDPHGLHCSGGVVLASR**	**S178**
**gi|7525040**	ATP synthase CF1 beta subunit	**IVGEEHYEpTAQQVK**	**IVGEEHYEpTAQQVK**	**T387**
		-	TNPpTTSNPEVSIR	T3
		-	VGLpTALTMAEYFR	T252
**gi|15223049**	L-ascorbate peroxidase (APX1)	QM*GLpSDKDIVALSGAHTLGR	-	S152
		-	ELLpSGEKEGLLQLVSDK	S196
**gi|15226610**	ATPDIL2-1/MEE30/UNE5	AGHDYDGGRDLDDFVpSFINEK	DLDDFVpSFINEK	S243
		ELVAApSEDEKK	-	S280
		GpSDYASKETER	-	S321
**gi|15228407**	Mn-superoxide dismutase (MSD1)	YApSEVYEKENN	-	S223
		-	GpSLGSAIDAHFGSLEGLVK	S124
		-	HHQAYVTNpYNNALEQLDQAVNK	Y67
		-	LVVDpTTANQDPLVTK	T171
**gi|15231718**	Peroxiredoxin type 2	**pTILFAVPGAFTPTCSQK**	**pTILFAVPGAFTPTCSSQK**	**T108**
		**VLNLEEGGAFpTNSSAEDMLK**	**VLNLEEGGAFpTNSSAEDMLK**	**T223**
		-	LPDpSTLSYLDPSTGDVK	S82
		VLNLEEGGAFpTNSSAEDM*LK	-	T223
**gi|15236678**	Ascorbate peroxidase 4 (APX4)	AENEGLpSDGLSLIEEVKK	-	S155
		-	GGPIpSYADIIQLAGQSAVK	S178
**gi|20197312**	Glutathione S-transferase (GST6)	**AIpTQYLAEEYSEKGEK**	**AIpTQYLAEEYSEK**	**T72**
		-	GMFGMpTTDPAAVQELEGK	T129
		-	QEAHLALNPFGQIPALEDGDLpTLFESR	T64
**gi|13926229**	Rubisco small chain 1A (RBCS1A)	**EHGNpTPGYYDGR**	**EHGNpTPGYYDGR**	**T58**
		**KFEpTLSYLPDLTDSELAK**	**KFEpTLSYLPDLSDVELAK**	**T14**
		-	FEpTLpSYLPDLSDVELAK	T14
		-	KFEpTLpSYLPDLSDVELAK	T14, S16
		**LPLFGCpTDSAQVLK**	**LPLFGCpTDSAQVLK**	**T78**
**gi|15229349**	Ribose 5-phosphate isomerase	***LLpSGSELYDIVGIPTSK***	***LLSpSGELYDIVGIPTSK***	***S86*, *S87***
		**pSLGIPLVGLDTHPR**	**pSLGIPLVGLDTHPR**	**S108**
		LQDLFKEFGCEpSK	-	S206
**gi|15226314**	Chaperonin 60 alpha (CPN60A)	IpTAIKDIIPILEK	-	T272
		-	HGLLpSVTSGANPVSLK	S150
**gi|15242045**	Chaperonin 20 (CPN20)	**YpTSIKPLGDR**	**YpTSIKPLGDR**	**T60**
		pYTSIKPLGDR	-	Y59
		pTLGGILLPSTAQSKPQGGEVVAVGEGR	-	T80
**gi|16221**	Chaperonin HSP60	VpTKDGVTVAK	-	T80
		-	GIpSMAVDAVVTNLK	S151

Heat shock proteins (HSPs) are involved in bud dormancy [48] and phosphorylation of HSPs and chaperonin has been reported in *Arabidopsis* [21,49] and poplar.[27] Our results confirm phosphorylation of these proteins in *Arabidopsis* seedlings and identify sites of phosphorylation in HSP60 (T80) and chaperonin 20 (adjacent residues Y59, T60 and S61) that, to the best of our knowledge, have not been reported before (S2C Fig and S2D Fig).

### Comparing protein phosphorylation at different stages of development

In a previous study we used IMAC to recover and identify 132 phosphoproteins with 252 component phosphopeptides in mature *Arabidopsis* leaf extracts (Fig 4A), following polyethylene glycol (PEG) fractionation to deplete Rubisco.[11] Having now used IMAC to recover and identify intact phosphoproteins in Rubisco-depleted extracts from young seedlings we decided to compare the results of the two studies. Of the 82 phosphoproteins identified in post-embryonic seedlings 28 were also identified in mature leaves, with 26 component phosphopeptides showing the same sites of phosphorylation in both tissues (Fig 4A, Table 3). For example, phosphorylation of the Rubisco small chain 1A at T58, T14 and T78 was observed in both seedlings and leaves, confirming phosphorylation of the protein at those sites. However, some of the phosphopeptides spanning the same amino acid sequence in both tissues showed a difference in protein phosphorylation state between young seedlings and mature leaves. For example, the CA2 peptide VLAESESSAFEDQCGR was identified in both tissues but was phosphorylated at S191 in seedlings and at S193 in leaves. Tryptic peptides showing differential phosphorylation of four other proteins (NAD^+^ MDH, PBP1, avirulence responsive protein, and ribose 5-phosphate isomerase) were also observed (Table 3), suggesting that these proteins may play a significant role in *Arabidopsis* development.

Comparing the phosphorylation status of 12S seed storage protein (cruciferin) in young seedlings (Table 1, spot 80) and dormant *Arabidopsis* seeds [50] shows that certain phosphorylation sites (T395 and T420) are common to both tissues, thereby validating the current method with reference to results obtained during a previous in-depth study of cruciferin phosphorylation. However, an apparent shift in phosphorylation site from S367 in dormant seeds to S366 in post-embryonic seedlings again demonstrates the ability to detect subtle changes in phosphorylation status that may have implications for seed storage protein mobilization and other processes during plant development,[50] although further investigations are required to confirm the significance of these findings.

## Conclusions

Seedling establishment involves the efficient utilization of endogenous protein reserves and external resources, requiring that developmental and metabolic programs adapt to the prevailing environmental conditions.[51] Using a combination of Rubisco depletion and IMAC enrichment of intact phosphoproteins we identified and characterized the phosphorylated forms of 82 proteins expressed in *Arabidopsis* young seedlings. These included enzymes involved in chloroplast development, mobilization of TAG, and other processes known to be important during the early stages of plant development. Comparison of our results for young seedlings with those obtained previously for *Arabidopsis* seeds [50] and mature leaves [11] shows that some of these proteins undergo differential phosphorylation during plant growth, and that protein level enrichment appears to enhance detection of pT and pY residues. Our study complements previous investigations by identifying an additional 43 proteins and 136 residues that undergo phosphorylation in *Arabidopsis* young seedlings. By purifying and enriching phosphorylated proteins under non-denaturing conditions our approach also lends itself to the study of phosphorylation in endogenous protein complexes and during protein-protein interactions.

## Supporting Information

S1 Fig Identification of novel phosphorylation sites using tandem mass spectrometry (MS/MS). The MS/MS spectra correspond to phosphopeptides with the following mass-to-charge (*m/z*) ratios, as obtained by trypsin digestion of proteins selected from the 2-DE gel shown in Fig 3. (A) *m/z* 701.377, showing phosphorylation of FBA (spot 17) at S84; (B) *m/z* 878.968, showing phosphorylation of GAPC-2 (spot 18) at T70; (C) *m/z* 619.981, showing phosphorylation of cytosolic TPI (spot 64) at S178; and (D) *m/z* 721.369, showing phosphorylation of PMDH1 (spot 25) at T138.
(PDF)

S2 Fig Identification of novel phosphorylation sites using tandem mass spectrometry. The MS/MS spectra correspond to phosphopeptides with the following mass-to-charge (*m/z*) ratios, as obtained by trypsin digestion of proteins selected from the 2-DE gel shown in Fig 3. (A) *m/z* 745.425, showing phosphorylation of ATGST10 (spot 47) at T4; (B) *m/z* 612.269, showing phosphorylation of ATGST6 (spot 49) at T15; (C) *m/z* 549.250, showing phosphorylation of HSP60 (spot 78) at T80; and (D) *m/z* 615.308, showing phosphorylation of chaperonin 20 (spot 76) at S61.
(PDF)

S3 Fig Phosphopeptide MS/MS spectra and MASCOT search results for selected phosphoproteins. (PDF)
