# Correction: Enrichment and Analysis of Intact Phosphoproteins in Arabidopsis Seedlings

**DOI:** 10.1371/journal.pone.0134535

**Published:** 2015-07-29

**Authors:** The PLOS ONE Staff

There are errors in the current addresses for authors Uma. K. Aryal and Andrew R. S. Ross. The correct current address for Uma. K. Aryal is Department of Biochemistry, Purdue University, West Lafayette, IN 47907, USA. The correct current address for Andrew R. S. Ross is Fisheries and Oceans Canada, Institute of Ocean Sciences, Sidney, BC, V8L 4B2, Canada. The publisher apologizes for the errors.
