# Supplementary material for: Enrichment and Analysis of Intact Phosphoproteins in Arabidopsis Seedlings
Source: PLoS One. 2015 Jul 9;10(7):e0130763. doi: 10.1371/journal.pone.0130763 (PMC4497735; doi:10.1371/journal.pone.0130763)

83 SpSDGKLFVDILK 94
2: TOF MSMS 701.377 ES$^+$

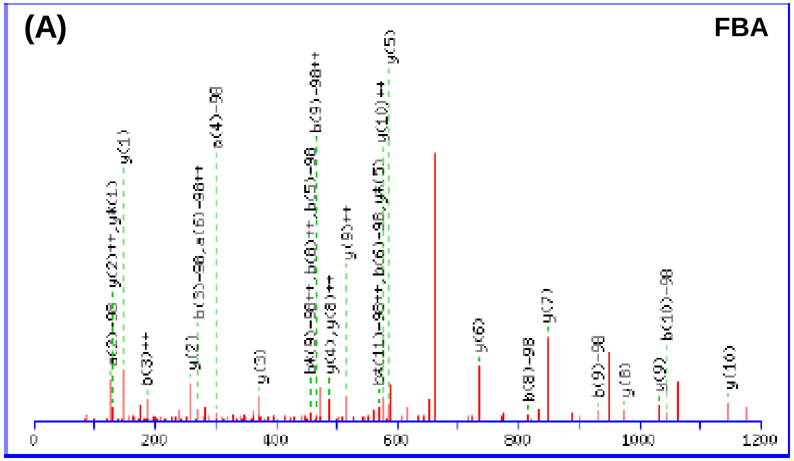

70 pTLLFGEKPVTVFGIR 84
2: TOF MSMS 878.968 ES$^+$

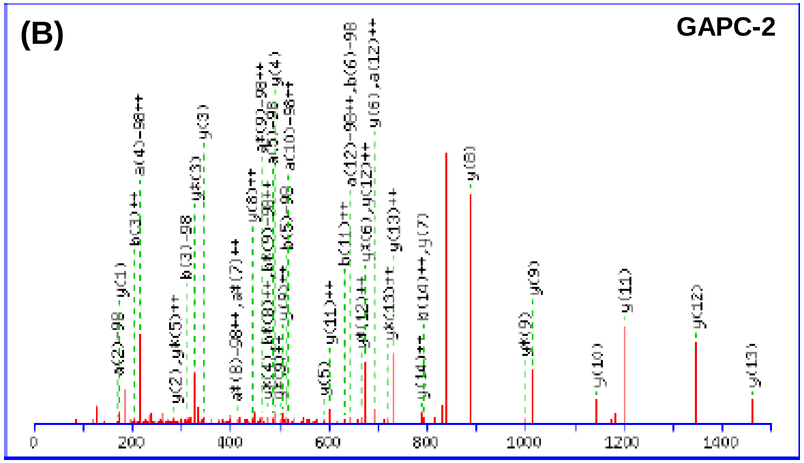

176 VApSPAQAQEVHDELRK 191
2: TOF MSMS 619.981 ES$^+$

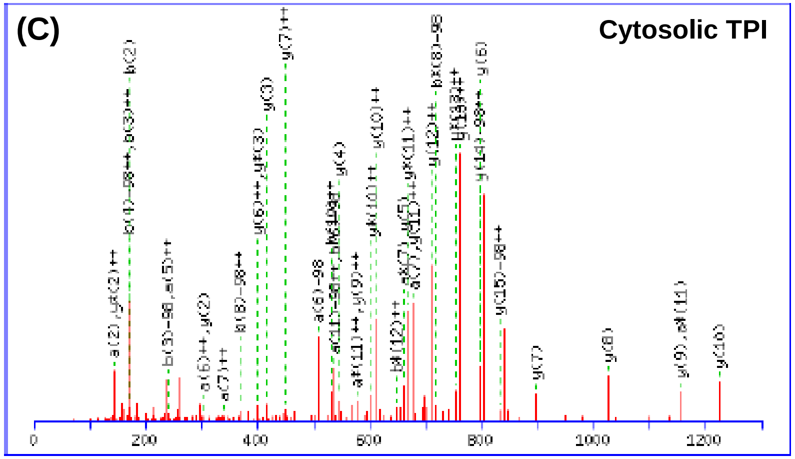

133 KLMGVpTMLDVVR 144
2: TOF MSMS 721.369 ES$^+$

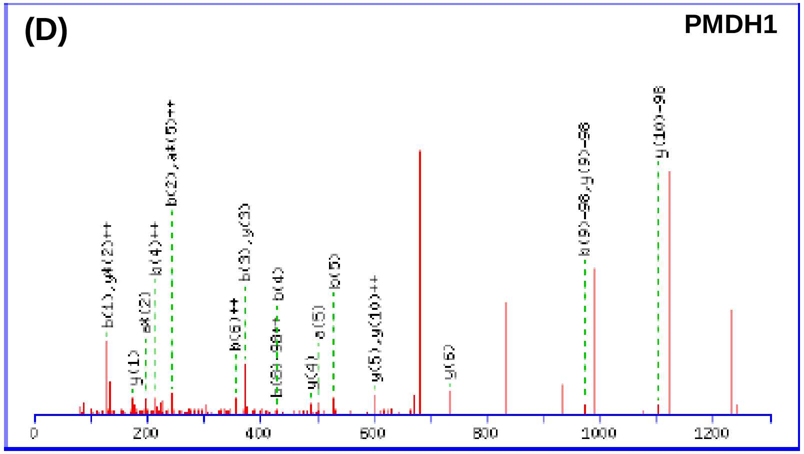

Supplement: S1 Fig — The MS/MS spectra correspond to phosphopeptides with the following mass-to-charge (m/z) ratios, as obtained by trypsin digestion of proteins selected from the 2-DE gel shown in Fig 3. (A) m/z 701.377, showing phosphorylation of FBA (spot 17) at S84; (B) m/z 878.968, showing phosphorylation of GAPC-2 (spot 18) at T70; (C) m/z 619.981, showing phosphorylation of cytosolic TPI (spot 64) at S178; and (D) m/z 721.369, showing phosphorylation of PMDH1 (spot 25) at T138. (PDF) [file pone.0130763.s001.pdf]
