# Supplementary figures and images for: Enrichment and Analysis of Intact Phosphoproteins in Arabidopsis Seedlings
Source: PLoS One. 2015 Jul 9;10(7):e0130763. doi: 10.1371/journal.pone.0130763 (PMC4497735; doi:10.1371/journal.pone.0130763)

2 VLPTIYAPLFASSK 14  
2: TOF MSMS 745.425 ES<sup>+</sup>

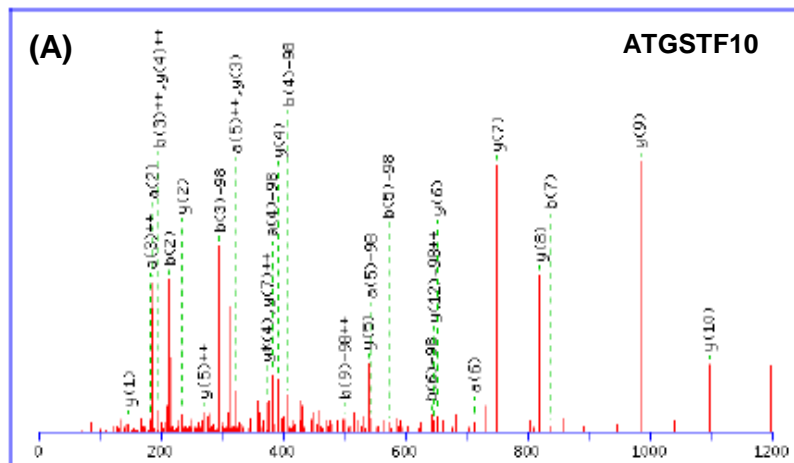

Supplement: S2 Fig — The MS/MS spectra correspond to phosphopeptides with the following mass-to-charge (m/z) ratios, as obtained by trypsin digestion of proteins selected from the 2-DE gel shown in Fig 3. (A) m/z 745.425, showing phosphorylation of ATGST10 (spot 47) at T4; (B) m/z 612.269, showing phosphorylation of ATGST6 (spot 49) at T15; (C) m/z 549.250, showing phosphorylation of HSP60 (spot 78) at T80; and (D) m/z 615.308, showing phosphorylation of chaperonin 20 (spot 76) at S61. (PDF) [file pone.0130763.s002.pdf]
