# Supplementary material for: Enrichment and Analysis of Intact Phosphoproteins in Arabidopsis Seedlings
Source: PLoS One. 2015 Jul 9;10(7):e0130763. doi: 10.1371/journal.pone.0130763 (PMC4497735; doi:10.1371/journal.pone.0130763)
Supplement: S3 Fig — (PDF) [file pone.0130763.s003.pdf]

Figure S3: CID MS/MS spectra of selected phosphopeptides with MASCOT ion scores and matched fragment ions. The numbers in the top right corner correspond to the 2-DE gel spots shown in Figure 3 and the identified proteins listed in Table 1. Lower-case letters after the spot number (e.g. 4a, 4b) indicate different phosphopeptides that match the same phosphoprotein.

MS/MS Fragmentation of DVTELIGNTPLVYLNNVAEGCVGR
Found in gi|15233613, OASA1 (O-ACETYLSERINE (THIOL) LYASE (OAS-TL) ISOFORM A1) [Arabidopsis thaliana]

Match to Query 191: 2682.348448 from(1342.181500,2+) intensity(3037.0000)
Title: 187: Sum of 4 scans in range 1886 (rt=47.7167, f=2, i=219) to 1889 (rt=47.8197, f=2, i=222)

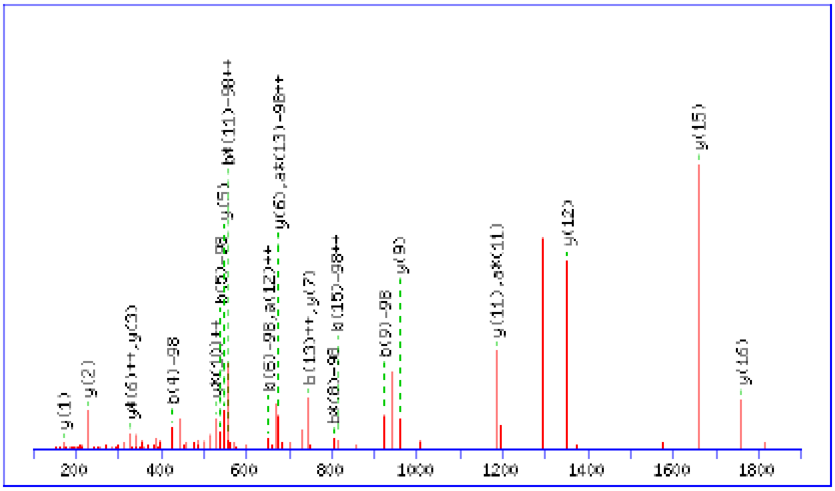

Monoisotopic mass of neutral peptide Mr(calc): 2682.2724
Fixed modifications: Carbamidomethyl (C)
Variable modifications:
T3 : Phospho (ST), with neutral losses 97.9769(shown in table), 0.0000
Ions Score: 60 **Expect:** 0.0032
Matches (Bold Red): 24/404 fragment ions using 35 most intense peaks

#	a	a^{++}	a*	a^{*++}	b	b^{++}	b*	b^{*++}	Seq.	y	y^{++}	y*	y^{*++}	#
1	88.0393	44.5233			116.0342	58.5207			D					24
2	187.1077	94.0575			215.1026	108.0550			V	2470.2759	1235.6416	2453.2493	1227.1283	23
3	270.1448	135.5761			298.1397	149.5735			T	2371.2075	1186.1074	2354.1809	1177.5941	22
4	399.1874	200.0973			**427.1823**	214.0948			E	2288.1703	1144.5888	2271.1438	1136.0755	21
5	512.2715	256.6394			**540.2664**	270.6368			L	2159.1278	1080.0675	2142.1012	1071.5542	20
6	625.3555	313.1814			**653.3505**	327.1789			I	2046.0437	1023.5255	2029.0171	1015.0122	19
7	682.3770	341.6921			710.3719	355.6896			G	1932.9596	966.9834	1915.9331	958.4702	18
8	796.4199	398.7136	779.3934	390.2003	824.4149	412.7111	**807.3883**	404.1978	N	1875.9382	938.4727	1858.9116	929.9594	17
9	897.4676	449.2374	880.4411	440.7242	**925.4625**	463.2349	908.4360	454.7216	T	**1761.8952**	881.4513	1744.8687	872.9380	16
10	994.5204	497.7638	977.4938	489.2506	1022.5153	511.7613	1005.4887	503.2480	P	**1660.8476**	830.9274	1643.8210	822.4141	15
11	1107.6044	554.3059	1090.5779	545.7926	1135.5994	568.3033	1118.5728	**559.7900**	L	1563.7948	782.4010	1546.7682	773.8878	14
12	1206.6729	603.8401	1189.6463	595.3268	1234.6678	617.8375	1217.6412	609.3243	V	1450.7107	725.8590	1433.6842	717.3457	13
13	1369.7362	685.3717	1352.7096	**676.8585**	1397.7311	699.3692	1380.7046	690.8559	Y	**1351.6423**	676.3248	1334.6158	667.8115	12
14	1482.8203	741.9138	1465.7937	733.4005	1510.8152	755.9112	1493.7886	747.3979	L	**1188.5790**	594.7931	1171.5524	586.2799	11
15	1596.8632	798.9352	1579.8366	790.4220	1624.8581	**812.9327**	1607.8315	804.4194	N	1075.4949	538.2511	1058.4684	**529.7378**	10
16	1710.9061	855.9567	1693.8796	847.4434	1738.9010	869.9541	1721.8745	861.4409	N	**961.4520**	481.2296	944.4254	472.7164	9
17	1809.9745	905.4909	1792.9480	896.9776	1837.9694	919.4884	1820.9429	910.9751	V	847.4091	424.2082	830.3825	415.6949	8
18	1881.0116	941.0095	1863.9851	932.4962	1909.0065	955.0069	1891.9800	946.4936	A	**748.3406**	374.6740	731.3141	366.1607	7
19	2010.0542	1005.5308	1993.0277	997.0175	2038.0491	1019.5282	2021.0226	1011.0149	E	**677.3035**	339.1554	660.2770	**330.6421**	6
20	2067.0757	1034.0415	2050.0491	1025.5282	2095.0706	1048.0389	2078.0441	1039.5257	G	**548.2609**	274.6341	531.2344	266.1208	5
21	2227.1063	1114.0568	2210.0798	1105.5435	2255.1013	1128.0543	2238.0747	1119.5410	C	491.2395	246.1234	474.2129	237.6101	4
22	2326.1748	1163.5910	2309.1482	1155.0777	2354.1697	1177.5885	2337.1431	1169.0752	V	**331.2088**	166.1081	314.1823	157.5948	3
23	2383.1962	1192.1017	2366.1697	1183.5885	2411.1911	1206.0992	2394.1646	1197.5859	G	**232.1404**	116.5738	215.1139	108.0606	2
24									R	**175.1190**	88.0631	158.0924	79.5498	1

MS/MS Fragmentation of ALDLASKPEGTGTPTK
Found in gi|18404496, binding / catalytic/ coenzyme binding [Arabidopsis thaliana]

Match to Query 109: 1664.742148 from(833.378350,2+) intensity(11738.0000)
Title: 44: Sum of 6 scans in range 1214 (rt=26.4625, f=2, i=74) to 1221 (rt=26.7493, f=3, i=47)

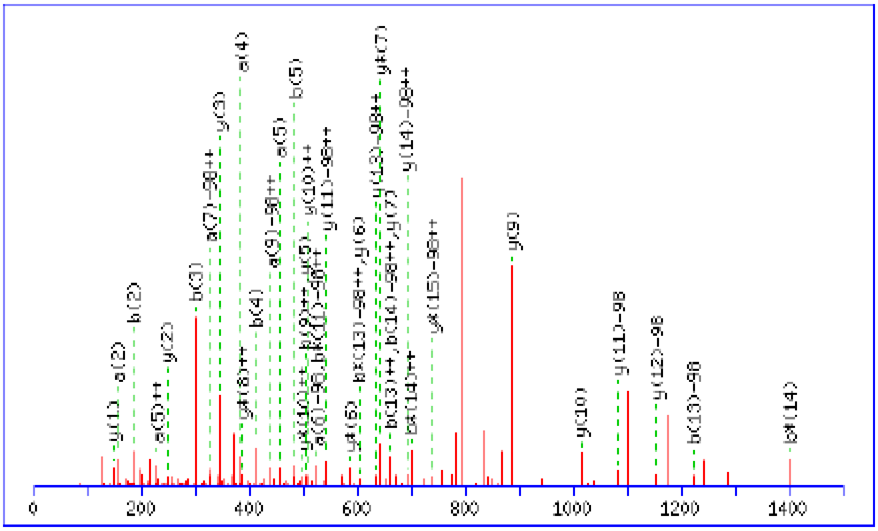

Monoisotopic mass of neutral peptide Mr(calc): 1664.8022
Fixed modifications: Carbamidomethyl (C)
Variable modifications:

S6 : Phospho (ST), with neutral losses 97.9769(shown in table), 0.0000
Ions Score: 54 **Expect:** 0.0092
Matches (Bold Red): 38/252 fragment ions using 61 most intense peaks

#	a	a++	a*	a*++	b	b++	b*	b*++	Seq.	y	y++	y*	y*++	#
1	44.0495	22.5284			72.0444	36.5258			A					16
2	**157.1335**	79.0704			**185.1285**	93.0679			L	1496.7955	748.9014	1479.7689	**740.3881**	15
3	272.1605	136.5839			**300.1554**	150.5813			D	1383.7114	**692.3594**	1366.6849	683.8461	14
4	**385.2445**	193.1259			**413.2395**	207.1234			L	1268.6845	**634.8459**	1251.6579	626.3326	13
5	**456.2817**	**228.6445**			**484.2766**	242.6419			A	**1155.6004**	578.3038	1138.5739	569.7906	12
6	**525.3031**	263.1552			553.2980	277.1527			S	**1084.5633**	**542.7853**	1067.5368	534.2720	11
7	653.3981	**327.2027**	636.3715	318.6894	681.3930	341.2001	664.3664	332.6869	K	**1015.5419**	**508.2746**	998.5153	**499.7613**	10
8	750.4508	375.7291	733.4243	367.2158	778.4458	389.7265	761.4192	381.2132	P	**887.4469**	444.2271	870.4203	435.7138	9
9	879.4934	**440.2504**	862.4669	431.7371	907.4884	454.2478	890.4618	445.7345	E	790.3941	395.7007	773.3676	**387.1874**	8
10	936.5149	468.7611	919.4884	460.2478	964.5098	482.7585	947.4833	474.2453	G	**661.3515**	331.1794	**644.3250**	322.6661	7
11	1037.5626	519.2849	1020.5360	510.7717	1065.5575	533.2824	1048.5309	**524.7691**	T	**604.3301**	302.6687	**587.3035**	294.1554	6
12	1094.5840	547.7957	1077.5575	539.2824	1122.5790	561.7931	1105.5524	553.2798	G	**503.2824**	252.1448	486.2558	243.6316	5
13	1195.6317	598.3195	1178.6052	589.8062	**1223.6266**	612.3170	1206.6001	**603.8037**	T	446.2609	223.6341	429.2344	215.1208	4
14	1292.6845	646.8459	1275.6579	638.3326	1320.6794	**660.8433**	1303.6529	652.3301	P	**345.2132**	173.1103	328.1867	164.5970	3
15	1393.7322	697.3697	1376.7056	688.8564	1421.7271	711.3672	1404.7005	702.8539	T	**248.1605**	124.5839	231.1339	116.0706	2
16									K	**147.1128**	74.0600	130.0863	65.5468	1

MS/MS Fragmentation of TSSTNGEDQKQSQNLR
Found in gi|15235213, caffeoyl-CoA 3-O-methyltransferase, putative [Arabidopsis thaliana]

Match to Query 90: 1871.893728 from(936.954140,2+) intensity(4522.0000)
Title: 3: Sum of 4 scans in range 1045 (rt=20.0895, f=2, i=13) to 1048 (rt=20.1921, f=2, i=16)

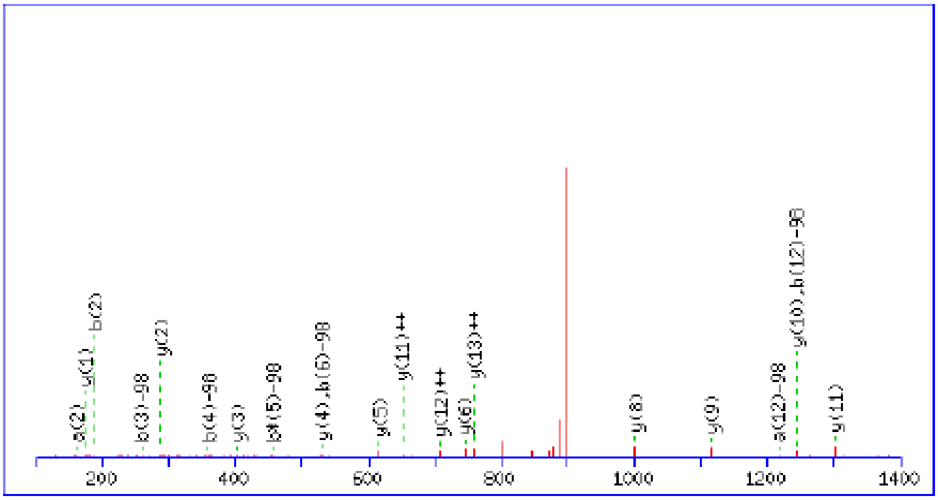

Monoisotopic mass of neutral peptide Mr(calc): 1871.8010
Fixed modifications: Carbamidomethyl (C)
Variable modifications:

S3 : Phospho (ST), with neutral losses 97.9769(shown in table), 0.0000
Ions Score: 68 **Expect:** 0.00048
Matches (Bold Red): 21/268 fragment ions using 34 most intense peaks

#	a	a++	a*	a*++	b	b++	b*	b*++	Seq.	y	y++	y*	y*++	#
1	74.0600	37.5337			102.0550	51.5311			T					16
2	**161.0921**	81.0497			**189.0870**	95.0471			S	1673.7838	837.3955	1656.7572	828.8822	15
3	230.1135	115.5604			**258.1084**	129.5579			S	1586.7517	793.8795	1569.7252	785.3662	14
4	331.1612	166.0842			**359.1561**	180.0817			T	1517.7303	**759.3688**	1500.7037	750.8555	13
5	445.2041	223.1057	428.1776	214.5924	473.1990	237.1032	**456.1725**	228.5899	N	1416.6826	**708.8449**	1399.6560	700.3317	12
6	502.2256	251.6164	485.1990	243.1032	**530.2205**	265.6139	513.1940	257.1006	G	**1302.6397**	**651.8235**	1285.6131	643.3102	11
7	631.2682	316.1377	614.2416	307.6245	659.2631	330.1352	642.2366	321.6219	E	**1245.6182**	623.3127	1228.5917	614.7995	10
8	746.2951	373.6512	729.2686	365.1379	774.2900	387.6487	757.2635	379.1354	D	**1116.5756**	558.7914	1099.5491	550.2782	9
9	874.3537	437.6805	857.3272	429.1672	902.3486	451.6779	885.3221	443.1647	Q	**1001.5487**	501.2780	984.5221	492.7647	8
10	1002.4487	501.7280	985.4221	493.2147	1030.4436	515.7254	1013.4170	507.2122	K	873.4901	437.2487	856.4635	428.7354	7
11	1130.5072	565.7573	1113.4807	557.2440	1158.5022	579.7547	1141.4756	571.2414	Q	**745.3951**	373.2012	728.3686	364.6879	6
12	**1217.5393**	609.2733	1200.5127	600.7600	**1245.5342**	623.2707	1228.5076	614.7575	S	**617.3365**	309.1719	600.3100	300.6586	5
13	1345.5979	673.3026	1328.5713	664.7893	1373.5928	687.3000	1356.5662	678.7867	Q	**530.3045**	265.6559	513.2780	257.1426	4
14	1459.6408	730.3240	1442.6142	721.8108	1487.6357	744.3215	1470.6091	735.8082	N	**402.2459**	201.6266	385.2194	193.1133	3
15	1572.7248	786.8661	1555.6983	778.3528	1600.7198	800.8635	1583.6932	792.3502	L	**288.2030**	144.6051	271.1765	136.0919	2
16									R	**175.1190**	88.0631	158.0924	79.5498	1

MS/MS Fragmentation of TSSTNGEDQKQSQNLR
Found in gi|15235213, caffeoyl-CoA 3-O-methyltransferase, putative [Arabidopsis thaliana]

Match to Query 112: 1871.700088 from(936.857320,2+) intensity(2461.0000)
Title: 6: Sum of 3 scans in range 1261 (rt=23.9491, f=2, i=24) to 1265 (rt=24.1168, f=2, i=26)

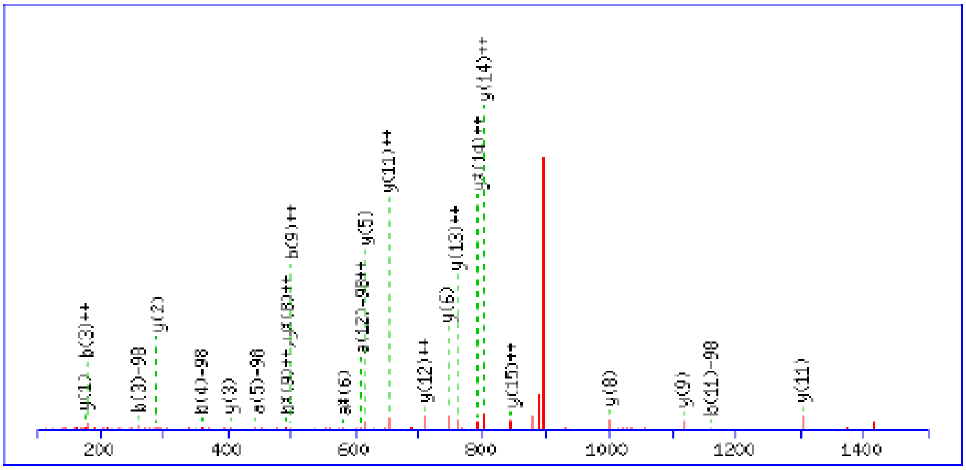

Monoisotopic mass of neutral peptide Mr(calc): 1871.8010
Fixed modifications: Carbamidomethyl (C)
Variable modifications:

T1 : Phospho (ST), with neutral losses 97.9769(shown in table), 0.0000
Ions Score: 44 **Expect:** 0.11
Matches (Bold Red): 24/268 fragment ions using 44 most intense peaks

#	a	a++	a*	a*++	b	b++	b*	b*++	Seq.	y	y++	y*	y*++	#
1	56.0495	28.5284			84.0444	42.5258			T					16
2	143.0815	72.0444			171.0764	86.0418			S	1691.7943	**846.4008**	1674.7678	837.8875	15
3	230.1135	115.5604			**258.1084**	129.5579			S	1604.7623	**802.8848**	1587.7358	**794.3715**	14
4	331.1612	166.0842			**359.1561**	180.0817			T	1517.7303	**759.3688**	1500.7037	750.8555	13
5	**445.2041**	223.1057	428.1776	214.5924	473.1990	237.1032	456.1725	228.5899	N	1416.6826	**708.8449**	1399.6560	700.3317	12
6	502.2256	251.6164	485.1990	243.1032	530.2205	265.6139	513.1940	257.1006	G	**1302.6397**	**651.8235**	1285.6131	643.3102	11
7	631.2682	316.1377	614.2416	307.6245	659.2631	330.1352	642.2366	321.6219	E	1245.6182	623.3127	1228.5917	614.7995	10
8	746.2951	373.6512	729.2686	365.1379	774.2900	387.6487	757.2635	379.1354	D	**1116.5756**	558.7914	1099.5491	550.2782	9
9	874.3537	437.6805	857.3272	429.1672	902.3486	451.6779	885.3221	443.1647	Q	**1001.5487**	501.2780	984.5221	**492.7647**	8
10	1002.4487	501.7280	985.4221	493.2147	1030.4436	515.7254	1013.4170	507.2122	K	873.4901	437.2487	856.4635	428.7354	7
11	1130.5072	565.7573	1113.4807	557.2440	**1158.5022**	579.7547	1141.4756	571.2414	Q	**745.3951**	373.2012	728.3686	364.6879	6
12	1217.5393	**609.2733**	1200.5127	600.7600	1245.5342	623.2707	1228.5076	614.7575	S	**617.3365**	309.1719	600.3100	300.6586	5
13	1345.5979	673.3026	1328.5713	664.7893	1373.5928	687.3000	1356.5662	678.7867	Q	530.3045	265.6559	513.2780	257.1426	4
14	1459.6408	730.3240	1442.6142	721.8108	1487.6357	744.3215	1470.6091	735.8082	N	**402.2459**	201.6266	385.2194	193.1133	3
15	1572.7248	786.8661	1555.6983	778.3528	1600.7198	800.8635	1583.6932	792.3502	L	**288.2030**	144.6051	271.1765	136.0919	2
16									R	**175.1190**	88.0631	158.0924	79.5498	1

MS/MS Fragmentation of TSKPEIFPASTDAR

Found in gi|30691732, aminoacylase, putative / N-acyl-L-amino-acid amidohydrolase, putative [Arabidopsis thaliana]

Match to Query 98: 1598.779188 from(800.396870,2+) intensity(5555.0000)
Title: 43: Sum of 3 scans in range 1473 (rt=30.7951, f=2, i=84) to 1479 (rt=31.0434, f=2, i=86)

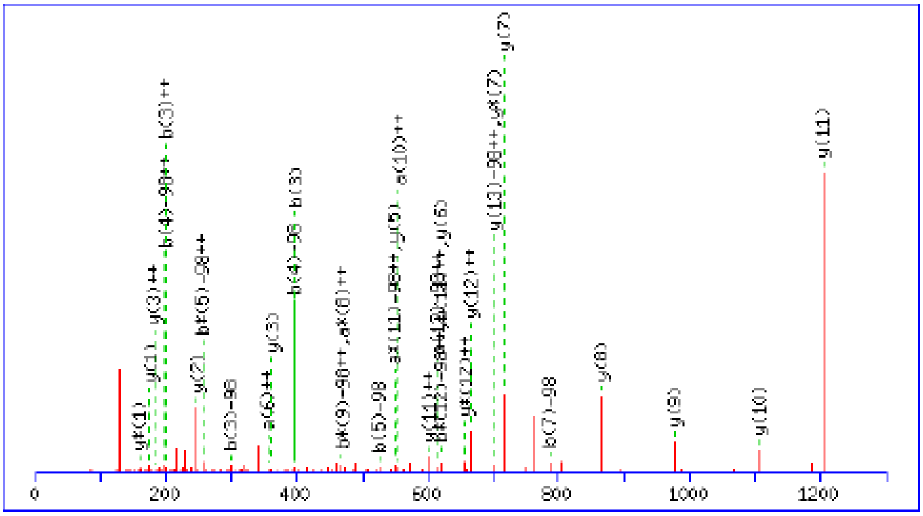

Monoisotopic mass of neutral peptide Mr(calc): 1598.7341
Fixed modifications: Carbamidomethyl (C)
Variable modifications:

S2 : Phospho (ST), with neutral losses 97.9769(shown in table), 0.0000
Ions Score: 56 **Expect:** 0.0057
Matches (Bold Red): 33/244 fragment ions using 53 most intense peaks

#	a	a^{++}	a^{*}	a^{*++}	b	b^{++}	b^{*}	b^{*++}	Seq.	y	y^{++}	y^{*}	y^{*++}	#
1	74.0600	37.5337			102.0550	51.5311			T					14
2	143.0815	72.0444			171.0764	86.0418			S	1400.7168	**700.8621**	1383.6903	692.3488	13
3	271.1765	136.0919	254.1499	127.5786	**299.1714**	150.0893	282.1448	141.5761	K	1331.6954	**666.3513**	1314.6688	**657.8381**	12
4	368.2292	184.6182	351.2027	176.1050	**396.2241**	**198.6157**	379.1976	190.1024	P	**1203.6004**	**602.3039**	1186.5739	593.7906	11
5	497.2718	249.1395	480.2453	240.6263	**525.2667**	263.1370	508.2402	**254.6237**	E	**1106.5477**	553.7775	1089.5211	545.2642	10
6	610.3559	305.6816	593.3293	297.1683	638.3508	319.6790	621.3242	311.1658	I	**977.5051**	489.2562	960.4785	480.7429	9
7	757.4243	379.2158	740.3977	370.7025	**785.4192**	393.2132	768.3927	384.7000	F	**864.4210**	432.7141	847.3945	424.2009	8
8	854.4771	427.7422	837.4505	419.2289	882.4720	441.7396	865.4454	433.2264	P	**717.3526**	359.1799	**700.3260**	350.6667	7
9	925.5142	463.2607	908.4876	454.7474	953.5091	477.2582	936.4825	**468.7449**	A	**620.2998**	310.6536	603.2733	302.1403	6
10	1012.5462	506.7767	995.5197	498.2635	1040.5411	520.7742	1023.5146	512.2609	S	**549.2627**	275.1350	532.2362	266.6217	5
11	1113.5939	557.3006	1096.5673	**548.7873**	1141.5888	571.2980	1124.5622	562.7848	T	462.2307	231.6190	445.2041	223.1057	4
12	1228.6208	**614.8140**	1211.5943	606.3008	1256.6157	628.8115	1239.5892	**620.2982**	D	**361.1830**	**181.0951**	344.1565	172.5819	3
13	1299.6579	650.3326	1282.6314	641.8193	1327.6529	664.3301	1310.6263	655.8168	A	**246.1561**	123.5817	229.1295	115.0684	2
14									R	**175.1190**	88.0631	**158.0924**	79.5498	1

MS/MS Fragmentation of TKGEAGTGNVVEAVR
Found in gi|15224470, ATPDX1.1 (PYRIDOXINE BIOSYNTHESIS 1.1); protein heterodimerization [Arabidopsis thaliana]

Match to Query 86: 1566.819188 from(784.416870,2+) intensity(1431.0000)
Title: 22: Sum of 3 scans in range 1498 (rt=30.2096, f=3, i=17) to 1504 (rt=30.4556, f=3, i=19)

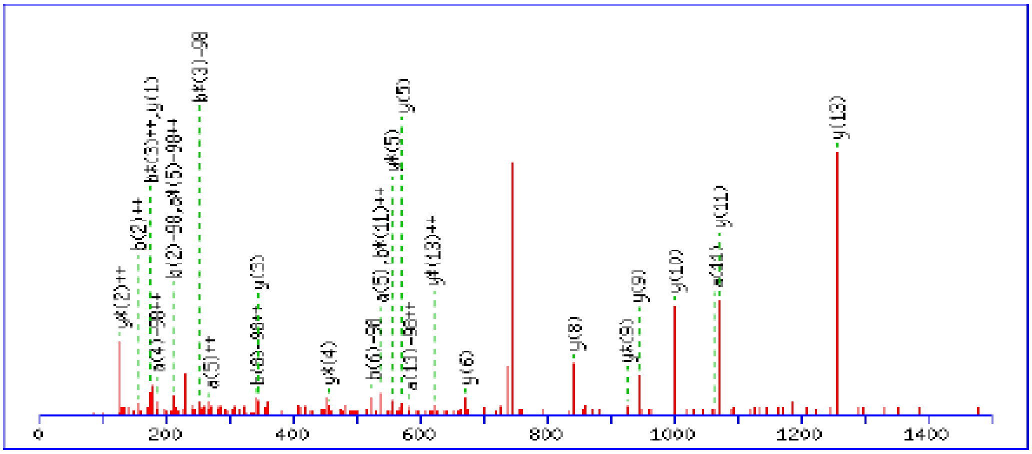

Monoisotopic mass of neutral peptide Mr(calc): 1566.7403
Fixed modifications: Carbamidomethyl (C)
Variable modifications:

T1 : Phospho (ST), with neutral losses 97.9769(shown in table), 0.0000
Ions Score: 29 **Expect:** 4.4
Matches (Bold Red): 27/272 fragment ions using 59 most intense peaks

#	a	a++	a*	a*++	b	b++	b*	b*++	Seq.	y	y++	y*	y*++	#
1	56.0495	28.5284			84.0444	42.5258			T					15
2	184.1444	92.5759	167.1179	84.0626	**212.1393**	106.5733	195.1128	98.0600	K	1386.7336	693.8704	1369.7070	685.3571	14
3	241.1659	121.0866	224.1393	112.5733	269.1608	135.0840	**252.1343**	126.5708	G	**1258.6386**	629.8229	1241.6121	**621.3097**	13
4	370.2085	**185.6079**	353.1819	177.0946	398.2034	199.6053	381.1769	191.0921	E	1201.6171	601.3122	1184.5906	592.7989	12
5	441.2456	221.1264	424.2191	**212.6132**	469.2405	235.1239	452.2140	226.6106	A	**1072.5745**	536.7909	1055.5480	528.2776	11
6	498.2671	249.6372	481.2405	241.1239	**526.2620**	263.6346	509.2354	255.1214	G	**1001.5374**	501.2724	984.5109	492.7591	10
7	599.3147	300.1610	582.2882	291.6477	627.3097	314.1585	610.2831	305.6452	T	**944.5160**	472.7616	**927.4894**	464.2483	9
8	656.3362	328.6717	639.3097	320.1585	684.3311	**342.6692**	667.3046	334.1559	G	**843.4683**	422.2378	826.4417	413.7245	8
9	770.3791	385.6932	753.3526	377.1799	798.3741	399.6907	781.3475	391.1774	N	786.4468	393.7271	769.4203	385.2138	7
10	869.4476	435.2274	852.4210	426.7141	897.4425	449.2249	880.4159	440.7116	V	**672.4039**	336.7056	655.3774	328.1923	6
11	968.5160	484.7616	951.4894	476.2483	996.5109	498.7591	979.4843	490.2458	V	**573.3355**	287.1714	**556.3089**	278.6581	5
12	1097.5586	549.2829	1080.5320	540.7696	1125.5535	563.2804	1108.5269	554.7671	E	474.2671	237.6372	**457.2405**	229.1239	4
13	1168.5957	**584.8015**	1151.5691	576.2882	1196.5906	598.7989	1179.5640	590.2857	A	**345.2245**	173.1159	328.1979	164.6026	3
14	1267.6641	634.3357	1250.6375	625.8224	1295.6590	648.3331	1278.6325	639.8199	V	274.1874	137.5973	257.1608	**129.0840**	2
15									R	**175.1190**	88.0631	158.0924	79.5498	1

MS/MS Fragmentation of GLAHITGGGFTDNIPR
Found in gi|15233161, ATPURM/PUR5; phosphoribosylformylglycinamidine cyclo-ligase [Arabidopsis thaliana]

Match to Query 82: 1704.752268 from(853.383410,2+) intensity(2011.0000)
Title: 61: Sum of 3 scans in range 1641 (rt=35.3288, f=2, i=123) to 1647 (rt=35.5771, f=2, i=125)

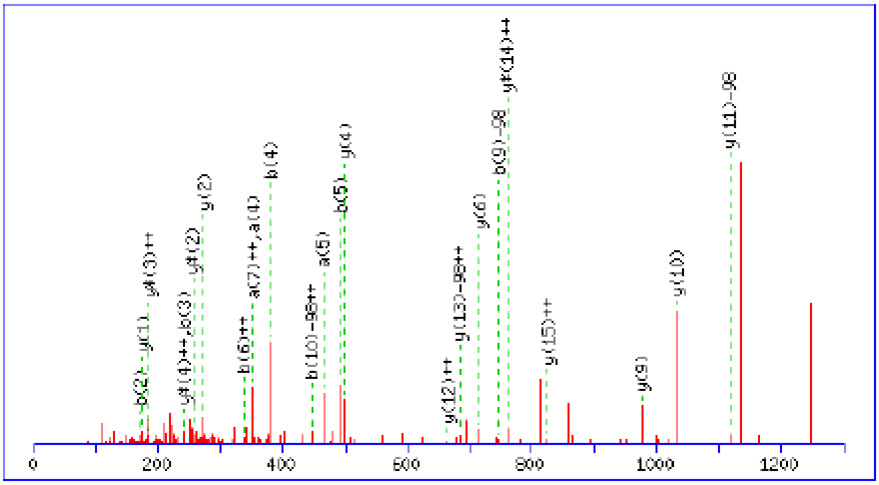

Monoisotopic mass of neutral peptide Mr(calc): 1704.7985
Fixed modifications: Carbamidomethyl (C)
Variable modifications:

T6 : Phospho (ST), with neutral losses 97.9769(shown in table), 0.0000
Ions Score: 20 **Expect:** 22
Matches (Bold Red): 24/204 fragment ions using 70 most intense peaks

#	a	a++	a*	a*++	b	b++	b*	b*++	Seq.	y	y++	y*	y*++	#
1	30.0338	15.5206			58.0287	29.5180			G					16
2	143.1179	72.0626			**171.1128**	86.0600			L	1550.8074	775.9073	1533.7808	767.3941	15
3	214.1550	107.5811			**242.1499**	121.5786			A	1437.7233	719.3653	1420.6968	710.8520	14
4	**351.2139**	176.1106			**379.2088**	190.1081			H	1366.6862	**683.8467**	1349.6597	675.3335	13
5	**464.2980**	232.6526			**492.2929**	246.6501			I	1229.6273	615.3173	1212.6008	606.8040	12
6	547.3351	274.1712			575.3300	288.1686			T	**1116.5432**	558.7753	1099.5167	550.2620	11
7	604.3566	302.6819			632.3515	316.6794			G	**1033.5061**	517.2567	1016.4796	508.7434	10
8	661.3780	331.1926			689.3729	345.1901			G	**976.4847**	488.7460	959.4581	480.2327	9
9	718.3995	359.7034			**746.3944**	373.7008			G	919.4632	460.2352	902.4367	451.7220	8
10	865.4679	433.2376			893.4628	**447.2350**			F	862.4417	431.7245	845.4152	423.2112	7
11	966.5156	483.7614			994.5105	497.7589			T	**715.3733**	358.1903	698.3468	349.6770	6
12	1081.5425	541.2749			1109.5374	555.2724			D	614.3256	307.6665	597.2991	299.1532	5
13	1195.5854	598.2964	1178.5589	589.7831	1223.5804	612.2938	1206.5538	603.7805	N	**499.2987**	250.1530	482.2722	**241.6397**	4
14	1308.6695	654.8384	1291.6430	646.3251	1336.6644	668.8358	1319.6379	660.3226	I	385.2558	193.1315	368.2292	**184.6183**	3
15	1405.7223	703.3648	1388.6957	694.8515	1433.7172	717.3622	1416.6906	708.8490	P	**272.1717**	136.5895	**255.1452**	128.0762	2
16									R	**175.1190**	88.0631	158.0924	79.5498	1

MS/MS Fragmentation of GNESYEDAIEALKK
Found in gi|42573371, CA2 (BETA CARBONIC ANHYDRASE 2); carbonate dehydratase/ zinc ion binding [Arabidopsis thaliana]

Match to Query 85: 1645.734268 from(823.874410,2+) intensity(10808.0000)
Title: 57: Sum of 4 scans in range 1596 (rt=33.9838, f=4, i=70) to 1599 (rt=34.0858, f=4, i=73)

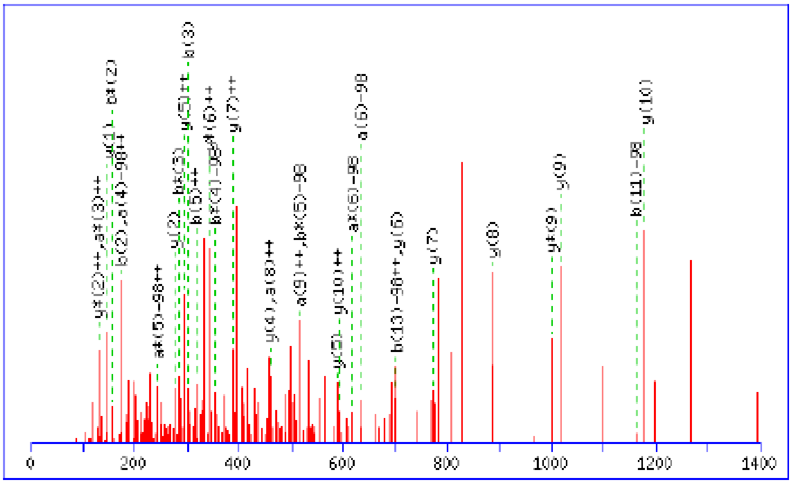

Monoisotopic mass of neutral peptide Mr(calc): 1645.7236
Fixed modifications: Carbamidomethyl (C)
Variable modifications:

S4 : Phospho (ST), with neutral losses 97.9769(shown in table), 0.0000
Ions Score: 40 **Expect:** 0.27
Matches (Bold Red): 31/244 fragment ions using 55 most intense peaks

#	a	a^{++}	a^{*}	a^{*++}	b	b^{++}	b^{*}	b^{*++}	Seq.	y	y^{++}	y^{*}	y^{*++}	#
1	30.0338	15.5206			58.0287	29.5180			G					14
2	144.0768	72.5420	127.0502	64.0287	**172.0717**	86.5395	**155.0451**	78.0262	N	1491.7326	746.3699	1474.7060	737.8566	13
3	273.1193	137.0633	256.0928	**128.5500**	**301.1143**	151.0608	**284.0877**	142.5475	E	1377.6896	689.3485	1360.6631	680.8352	12
4	342.1408	**171.5740**	325.1143	163.0608	370.1357	185.5715	**353.1092**	177.0582	S	1248.6470	624.8272	1231.6205	616.3139	11
5	505.2041	253.1057	488.1776	**244.5924**	533.1990	267.1032	**516.1725**	258.5899	Y	**1179.6256**	**590.3164**	1162.5990	581.8032	10
6	**634.2467**	317.6270	**617.2202**	309.1137	662.2416	331.6245	645.2151	323.1112	E	**1016.5623**	508.7848	**999.5357**	500.2715	9
7	749.2737	375.1405	732.2471	366.6272	777.2686	389.1379	760.2420	380.6247	D	**887.5197**	444.2635	870.4931	435.7502	8
8	820.3108	410.6590	803.2842	402.1458	848.3057	424.6565	831.2791	416.1432	A	**772.4927**	**386.7500**	755.4662	378.2367	7
9	933.3948	467.2011	916.3683	458.6878	961.3898	481.1985	944.3632	472.6852	I	**701.4556**	351.2314	684.4291	**342.7182**	6
10	1062.4374	531.7224	1045.4109	523.2091	1090.4324	545.7198	1073.4058	537.2065	E	**588.3715**	**294.6894**	571.3450	286.1761	5
11	1133.4746	567.2409	1116.4480	558.7276	**1161.4695**	581.2384	1144.4429	572.7251	A	**459.3289**	230.1681	442.3024	221.6548	4
12	1246.5586	623.7829	1229.5321	615.2697	1274.5535	637.7804	1257.5270	629.2671	L	388.2918	194.6496	371.2653	186.1363	3
13	1374.6536	687.8304	1357.6270	679.3172	1402.6485	**701.8279**	1385.6219	693.3146	K	**275.2078**	138.1075	258.1812	**129.5942**	2
14									K	**147.1128**	74.0600	130.0863	65.5468	1

8b

MS/MS Fragmentation of VCPSHVLDFHPGDAFVVR
Found in gi|42573371, CA2 (BETA CARBONIC ANHYDRASE 2); carbonate dehydratase/ zinc ion binding [Arabidopsis thaliana]

Match to Query 110: 2130.870702 from(711.297510,3+) intensity(7668.0000)

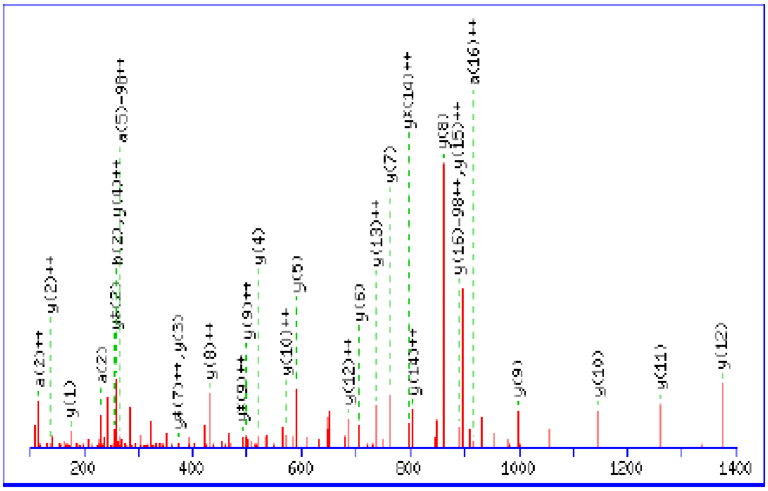

Monoisotopic mass of neutral peptide Mr(calc): 2130.9711
Fixed modifications: Carbamidomethyl (C)
Variable modifications:
S4 : Phospho (ST), with neutral losses 97.9769(shown in table), 0.0000

Ions Score: 50 **Expect:** 0.028
Matches (Bold Red): 30/204 fragment ions using 68 most intense peaks

#	a	a^{++}	b	b^{++}	Seq.	y	y^{++}	y*	y^{*++}	#
1	72.0808	36.5440	100.0757	50.5415	V					18
2	**232.1114**	**116.5594**	**260.1063**	130.5568	C	1934.9330	967.9701	1917.9065	959.4569	17
3	329.1642	165.0857	357.1591	179.0832	P	1774.9024	**887.9548**	1757.8758	879.4415	16
4	398.1856	199.5965	426.1806	213.5939	S	1677.8496	839.4284	1660.8230	830.9152	15
5	535.2446	**268.1259**	563.2395	282.1234	H	1608.8281	**804.9177**	1591.8016	**796.4044**	14
6	634.3130	317.6601	662.3079	331.6576	V	1471.7692	**736.3883**	1454.7427	727.8750	13
7	747.3970	374.2022	775.3919	388.1996	L	**1372.7008**	**686.8540**	1355.6743	678.3408	12
8	862.4240	431.7156	890.4189	445.7131	D	**1259.6167**	630.3120	1242.5902	621.7987	11
9	1009.4924	505.2498	1037.4873	519.2473	F	**1144.5898**	**572.7985**	1127.5633	564.2853	10
10	1146.5513	573.7793	1174.5462	587.7767	H	**997.5214**	**499.2643**	980.4948	**490.7511**	9
11	1243.6041	622.3057	1271.5990	636.3031	P	**860.4625**	**430.7349**	843.4359	422.2216	8
12	1300.6255	650.8164	1328.6204	664.8139	G	**763.4097**	382.2085	746.3832	**373.6952**	7
13	1415.6525	708.3299	1443.6474	722.3273	D	**706.3883**	353.6978	689.3617	345.1845	6
14	1486.6896	743.8484	1514.6845	757.8459	A	**591.3613**	296.1843	574.3348	287.6710	5
15	1633.7580	817.3826	1661.7529	831.3801	F	**520.3242**	**260.6657**	503.2976	252.1525	4
16	1732.8264	866.9168	1760.8213	880.9143	V	**373.2558**	187.1315	356.2292	178.6183	3
17	1831.8948	916.4511	1859.8897	930.4485	V	274.1874	**137.5973**	**257.1608**	129.0840	2
18					R	**175.1190**	88.0631	158.0924	79.5498	1

MS/MS Fragmentation of VQTSSGEKPVR
Found in gi|15219721, malate dehydrogenase, cytosolic, putative [Arabidopsis thaliana]

Match to Query 58: 1266.546608 from(634.280580,2+) intensity(5884.0000)
Title: 12: Sum of 3 scans in range 1069 (rt=21.3144, f=3, i=8) to 1075 (rt=21.5602, f=3, i=10)

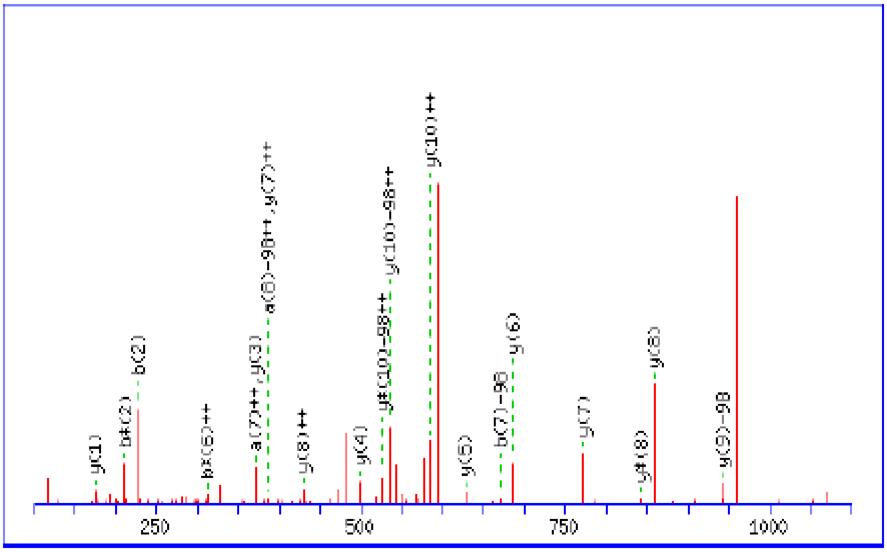

Monoisotopic mass of neutral peptide Mr(calc): 1266.5969
Fixed modifications: Carbamidomethyl (C)
Variable modifications:

T3 : Phospho (ST), with neutral losses 97.9769(shown in table), 0.0000
Ions Score: 52 **Expect:** 0.012
Matches (**Bold Red**): 20/188 fragment ions using 31 most intense peaks

#	a	a++	a*	a*++	b	b++	b*	b*++	Seq.	y	y++	y*	y*++	#
1	72.0808	36.5440			100.0757	50.5415			V					11
2	200.1394	100.5733	183.1128	92.0600	**228.1343**	114.5708	**211.1077**	106.0575	Q	1070.5589	**535.7831**	1053.5323	**527.2698**	10
3	283.1765	142.0919	266.1499	133.5786	311.1714	156.0893	294.1448	147.5761	T	**942.5003**	471.7538	925.4738	463.2405	9
4	370.2085	185.6079	353.1819	177.0946	398.2034	199.6053	381.1769	191.0921	S	**859.4632**	**430.2352**	**842.4367**	421.7220	8
5	457.2405	229.1239	440.2140	220.6106	485.2354	243.1214	468.2089	234.6081	S	**772.4312**	**386.7192**	755.4046	378.2060	7
6	514.2620	257.6346	497.2354	249.1214	542.2569	271.6321	525.2303	263.1188	G	**685.3992**	343.2032	668.3726	334.6899	6
7	643.3046	322.1559	626.2780	313.6427	**671.2995**	336.1534	654.2729	327.6401	E	**628.3777**	314.6925	611.3511	306.1792	5
8	771.3995	**386.2034**	754.3730	377.6901	799.3945	400.2009	782.3679	391.6876	K	**499.3351**	250.1712	482.3085	241.6579	4
9	868.4523	434.7298	851.4258	426.2165	896.4472	448.7272	879.4207	440.2140	P	**371.2401**	186.1237	354.2136	177.6104	3
10	967.5207	484.2640	950.4942	475.7507	995.5156	498.2615	978.4891	489.7482	V	274.1874	137.5973	257.1608	129.0840	2
11									R	**175.1190**	88.0631	158.0924	79.5498	1

MS/MS Fragmentation of YSAEGENEDAKK
Found in gi|15226185, fructose-bisphosphate aldolase, putative [Arabidopsis thaliana]

Match to Query 91: 1419.522748 from(710.768650,2+) intensity(1050.0000)
Title: 9: Sum of 3 scans in range 1058 (rt=20.9289, f=2, i=39) to 1062 (rt=21.0928, f=2, i=41)

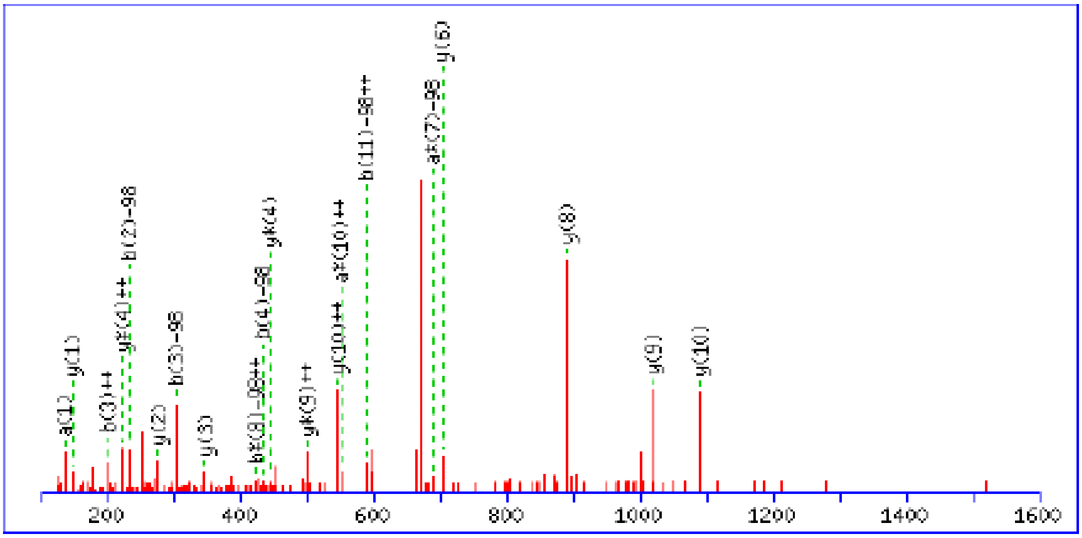

Monoisotopic mass of neutral peptide Mr(calc): 1419.5555
Fixed modifications: Carbamidomethyl (C)
Variable modifications:

S2 : Phospho (ST), with neutral losses 97.9769(shown in table), 0.0000
Ions Score: 20 **Expect:** 25
Matches (**Bold Red**): 20/172 fragment ions using 57 most intense peaks

#	a	a++	a*	a*++	b	b++	b*	b*++	Seq.	y	y++	y*	y*++	#
1	**136.0757**	68.5415			164.0706	82.5389			Y					12
2	205.0971	103.0522			**233.0921**	117.0497			S	1159.5226	580.2649	1142.4960	571.7516	11
3	276.1343	138.5708			**304.1292**	152.5682			A	**1090.5011**	**545.7542**	1073.4746	537.2409	10
4	405.1769	203.0921			**433.1718**	217.0895			E	**1019.4640**	510.2356	1002.4374	**501.7224**	9
5	462.1983	231.6028			490.1932	245.6003			G	**890.4214**	445.7143	873.3949	437.2011	8
6	591.2409	296.1241			619.2358	310.1216			E	833.3999	417.2036	816.3734	408.6903	7
7	705.2838	353.1456	**688.2573**	344.6323	733.2788	367.1430	716.2522	358.6297	N	**704.3573**	352.6823	687.3308	344.1690	6
8	834.3264	417.6669	817.2999	409.1536	862.3213	431.6643	845.2948	**423.1510**	E	590.3144	295.6608	573.2879	287.1476	5
9	949.3534	475.1803	932.3268	466.6670	977.3483	489.1778	960.3217	480.6645	D	461.2718	231.1395	**444.2453**	**222.6263**	4
10	1020.3905	510.6989	1003.3639	502.1856	1048.3854	524.6963	1031.3589	516.1831	A	**346.2449**	173.6261	329.2183	165.1128	3
11	1148.4855	574.7464	1131.4589	566.2331	1176.4804	**588.7438**	1159.4538	580.2305	K	**275.2078**	138.1075	258.1812	129.5942	2
12									K	**147.1128**	74.0600	130.0863	65.5468	1

MS/MS Fragmentation of VCPSHVLDFQPGDAFVVR
Found in gi|30678347, CA1 (CARBONIC ANHYDRASE 1); carbonate dehydratase/ zinc ion binding [Arabidopsis thaliana]

Match to Query 101: 2121.990248 from(1062.002400,2+) intensity(16367.0000)
Title: 97: Sum of 3 scans in range 1597 (rt=37.7824, f=2, i=212) to 1599 (rt=37.8505, f=2, i=214)

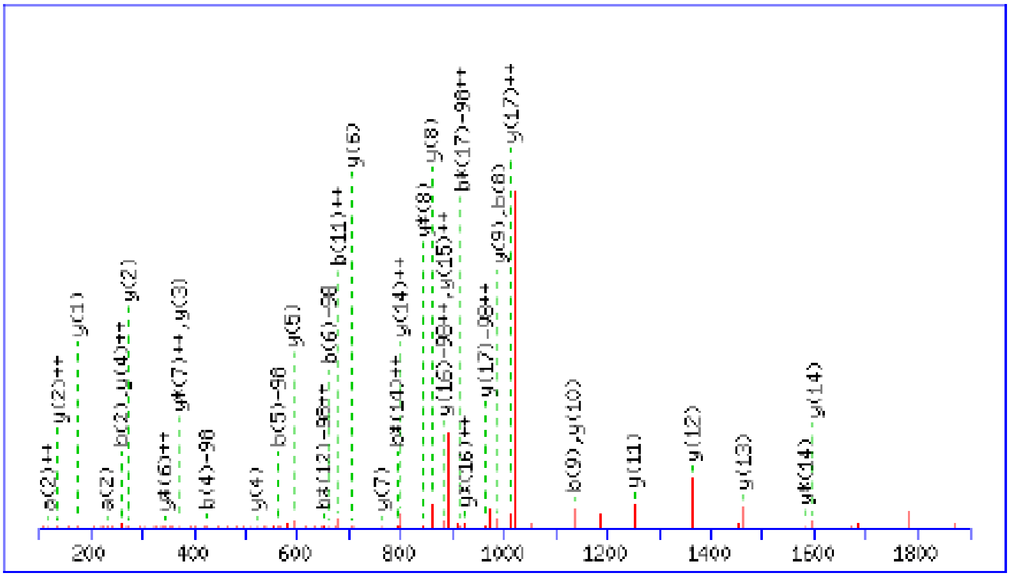

Monoisotopic mass of neutral peptide Mr(calc): 2121.9707
Fixed modifications: Carbamidomethyl (C)
Variable modifications:

S4 : Phospho (ST), with neutral losses 97.9769(shown in table), 0.0000
Ions Score: 73 **Expect:** 0.00013
Matches (Bold Red): 38/268 fragment ions using 67 most intense peaks

#	a	a^{++}	a*	a^{*++}	b	b^{++}	b*	b^{*++}	Seq.	y	y^{++}	y*	y^{*++}	#
1	72.0808	36.5440			100.0757	50.5415			V					18
2	**232.1114**	**116.5594**			**260.1063**	130.5568			C	1925.9327	**963.4700**	1908.9061	954.9567	17
3	329.1642	165.0857			357.1591	179.0832			P	1765.9020	**883.4547**	1748.8755	874.9414	16
4	398.1856	199.5965			**426.1806**	213.5939			S	1668.8493	834.9283	1651.8227	826.4150	15
5	535.2446	268.1259			**563.2395**	282.1234			H	**1599.8278**	**800.4175**	**1582.8013**	791.9043	14
6	634.3130	317.6601			**662.3079**	331.6576			V	**1462.7689**	731.8881	1445.7423	723.3748	13
7	747.3970	374.2022			775.3919	388.1996			L	**1363.7005**	682.3539	1346.6739	673.8406	12
8	862.4240	431.7156			890.4189	445.7131			D	**1250.6164**	625.8118	1233.5899	617.2986	11
9	1009.4924	505.2498			1037.4873	519.2473			F	**1135.5895**	568.2984	1118.5629	559.7851	10
10	1137.5510	569.2791	1120.5244	560.7658	1165.5459	583.2766	1148.5193	574.7633	Q	**988.5211**	494.7642	971.4945	486.2509	9
11	1234.6037	617.8055	1217.5772	609.2922	1262.5986	631.8030	1245.5721	623.2897	P	**860.4625**	430.7349	**843.4359**	422.2216	8
12	1291.6252	646.3162	1274.5986	637.8030	1319.6201	660.3137	1302.5936	**651.8004**	G	**763.4097**	382.2085	746.3832	**373.6952**	7
13	1406.6521	703.8297	1389.6256	695.3164	1434.6471	717.8272	1417.6205	709.3139	D	**706.3883**	353.6978	689.3617	**345.1845**	6
14	1477.6893	739.3483	1460.6627	730.8350	1505.6842	753.3457	1488.6576	744.8324	A	**591.3613**	296.1843	574.3348	287.6710	5
15	1624.7577	812.8825	1607.7311	804.3692	1652.7526	826.8799	1635.7260	818.3667	F	**520.3242**	**260.6657**	503.2976	252.1525	4
16	1723.8261	862.4167	1706.7995	853.9034	1751.8210	876.4141	1734.7944	867.9009	V	**373.2558**	187.1315	356.2292	178.6183	3
17	1822.8945	911.9509	1805.8679	903.4376	1850.8894	925.9483	1833.8629	**917.4351**	V	**274.1874**	**137.5973**	257.1608	129.0840	2
18									R	**175.1190**	88.0631	158.0924	79.5498	1

MS/MS Fragmentation of NVIHGSDSVESAR
Found in gi|16398, nucleoside diphosphate kinase [Arabidopsis thaliana]

Match to Query 58: 1449.583788 from(725.799170,2+) intensity(9135.0000)
Title: 20: Sum of 3 scans in range 1123 (rt=22.9684, f=2, i=46) to 1127 (rt=23.1323, f=2, i=48)

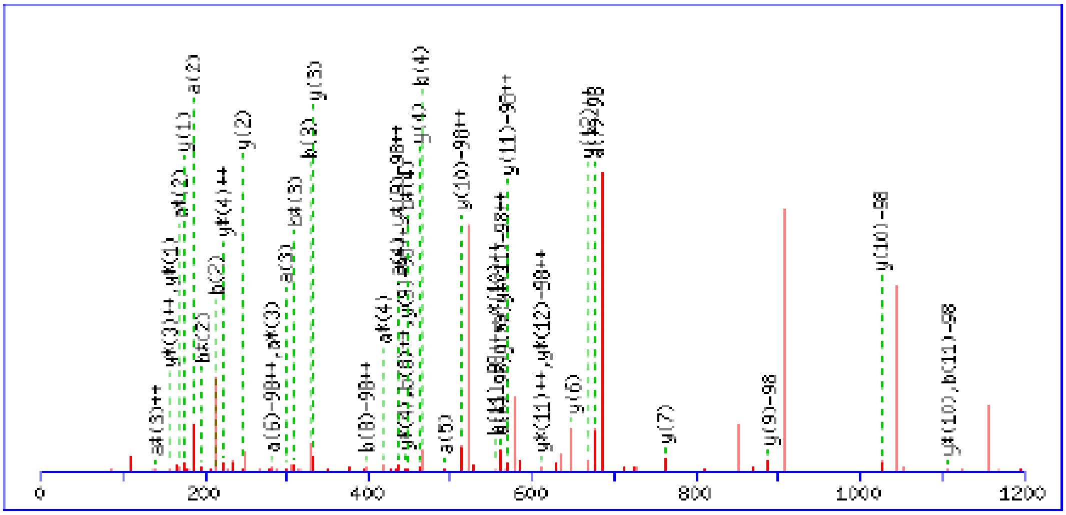

Monoisotopic mass of neutral peptide Mr(calc): 1449.6249
Fixed modifications: Carbamidomethyl (C)
Variable modifications:

S6 : Phospho (ST), with neutral losses 97.9769(shown in table), 0.0000
Ions Score: 36 **Expect:** 0.68
Matches (Bold Red): 44/220 fragment ions using 74 most intense peaks

#	a	a++	a*	a*++	b	b++	b*	b*++	Seq.	y	y++	y*	y*++	#
1	87.0553	44.0313	70.0287	35.5180	115.0502	58.0287	98.0237	49.5155	N					13
2	**186.1237**	93.5655	**169.0972**	85.0522	**214.1186**	107.5629	**197.0921**	99.0497	V	1238.6124	619.8098	1221.5858	**611.2966**	12
3	**299.2078**	150.1075	**282.1812**	**141.5942**	**327.2027**	164.1050	**310.1761**	155.5917	I	1139.5440	**570.2756**	1122.5174	**561.7623**	11
4	**436.2667**	218.6370	**419.2401**	210.1237	**464.2616**	232.6344	**447.2350**	224.1212	H	**1026.4599**	**513.7336**	1009.4334	505.2203	10
5	**493.2881**	247.1477	476.2616	238.6344	521.2831	261.1452	504.2565	252.6319	G	**889.4010**	**445.2041**	872.3744	**436.6909**	9
6	**562.3096**	**281.6584**	545.2830	273.1452	590.3045	295.6559	573.2780	287.1426	S	832.3795	416.6934	815.3530	408.1801	8
7	**677.3365**	339.1719	660.3100	330.6586	705.3315	353.1694	688.3049	344.6561	D	**763.3581**	382.1827	746.3315	373.6694	7
8	764.3686	382.6879	747.3420	374.1746	792.3635	**396.6854**	775.3369	388.1721	S	**648.3311**	324.6692	631.3046	316.1559	6
9	863.4370	432.2221	846.4104	423.7089	891.4319	446.2196	874.4053	437.7063	V	**561.2991**	281.1532	544.2726	272.6399	5
10	992.4796	496.7434	975.4530	488.2302	1020.4745	510.7409	1003.4479	502.2276	E	**462.2307**	231.6190	**445.2041**	**223.1057**	4
11	1079.5116	540.2594	1062.4851	531.7462	**1107.5065**	**554.2569**	1090.4800	545.7436	S	**333.1881**	167.0977	316.1615	**158.5844**	3
12	1150.5487	575.7780	1133.5222	567.2647	1178.5436	589.7755	1161.5171	581.2622	A	**246.1561**	123.5817	229.1295	115.0684	2
13									R	**175.1190**	88.0631	**158.0924**	79.5498	1

MS/MS Fragmentation of **KIIGATNPAASEPGTIR**
Found in **gi|16398**, nucleoside diphosphate kinase [Arabidopsis thaliana]

Match to Query 96: 1774.841208 from(888.427880,2+) intensity(3803.0000)
Title: 41: Sum of 4 scans in range 1267 (rt=27.195, f=3, i=42) to 1273 (rt=27.434, f=3, i=45)

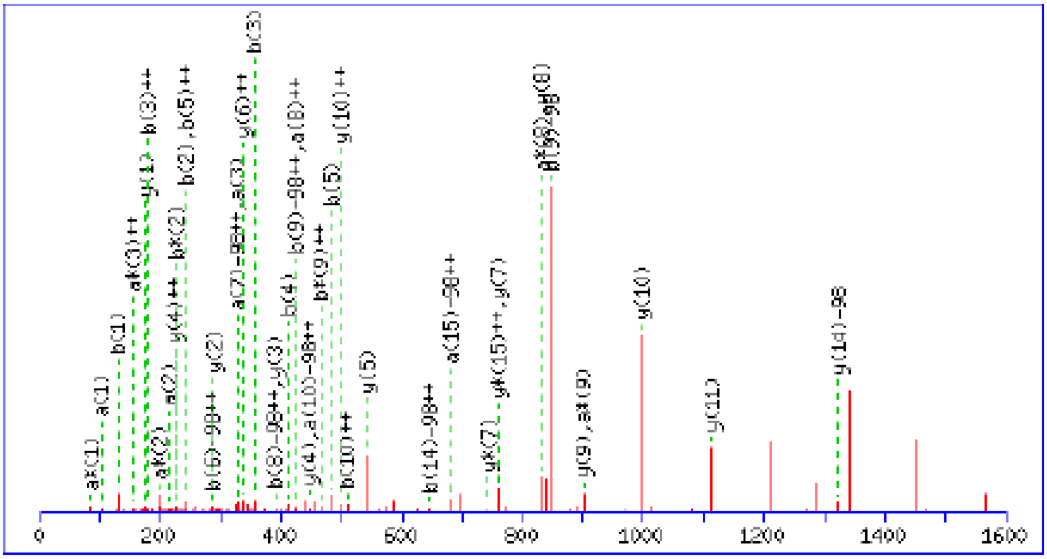

Monoisotopic mass of neutral peptide Mr(calc): 1774.8978
Fixed modifications: Carbamidomethyl (C)
Variable modifications:

T6 : Phospho (ST), with neutral losses 97.9769(shown in table), 0.0000
Ions Score: 59 **Expect:** 0.0032
Matches (Bold Red): 43/300 fragment ions using 71 most intense peaks

#	a	a^{++}	a^{*}	a^{*++}	b	b^{++}	b^{*}	b^{*++}	Seq.	y	y^{++}	y^{*}	y^{*++}	#
1	**101.1073**	51.0573	**84.0808**	42.5440	**129.1022**	65.0548	112.0757	56.5415	K					17
2	**214.1914**	107.5993	**197.1648**	99.0861	**242.1863**	121.5968	**225.1598**	113.0835	I	1549.8333	775.4203	1532.8067	766.9070	16
3	**327.2755**	164.1414	310.2489	**155.6281**	**355.2704**	**178.1388**	338.2438	169.6255	I	1436.7492	718.8782	1419.7227	710.3650	15
4	384.2969	192.6521	367.2704	184.1388	**412.2918**	206.6496	395.2653	198.1363	G	**1323.6651**	662.3362	1306.6386	653.8229	14
5	455.3340	228.1707	438.3075	219.6574	**483.3289**	**242.1681**	466.3024	233.6548	A	1266.6437	633.8255	1249.6171	625.3122	13
6	538.3711	269.6892	521.3446	261.1759	566.3661	**283.6867**	549.3395	275.1734	T	1195.6066	598.3069	1178.5800	589.7936	12
7	652.4141	**326.7107**	635.3875	318.1974	680.4090	340.7081	663.3824	332.1949	N	**1112.5695**	556.7884	1095.5429	548.2751	11
8	749.4668	375.2371	732.4403	366.7238	777.4617	**389.2345**	760.4352	380.7212	P	**998.5265**	**499.7669**	981.5000	491.2536	10
9	820.5039	410.7556	803.4774	402.2423	**848.4989**	**424.7531**	831.4723	416.2398	A	**901.4738**	451.2405	884.4472	442.7272	9
10	891.5411	**446.2742**	874.5145	437.7609	919.5360	460.2716	902.5094	451.7583	A	**830.4367**	415.7220	813.4101	407.2087	8
11	978.5731	489.7902	961.5465	481.2769	1006.5680	503.7876	989.5414	495.2744	S	**759.3995**	380.2034	**742.3730**	371.6901	7
12	1107.6157	554.3115	1090.5891	545.7982	1135.6106	568.3089	1118.5840	559.7957	E	672.3675	**336.6874**	655.3410	328.1741	6
13	1204.6684	602.8379	1187.6419	594.3246	1232.6634	616.8353	1215.6368	608.3220	P	**543.3249**	272.1661	526.2984	263.6528	5
14	1261.6899	631.3486	1244.6634	622.8353	1289.6848	**645.3460**	1272.6583	636.8328	G	**446.2722**	**223.6397**	429.2456	215.1264	4
15	1362.7376	**681.8724**	1345.7110	673.3592	1390.7325	695.8699	1373.7060	687.3566	T	**389.2507**	195.1290	372.2241	186.6157	3
16	1475.8216	738.4145	1458.7951	729.9012	1503.8166	752.4119	1486.7900	743.8986	I	**288.2030**	144.6051	271.1765	136.0919	2
17									R	**175.1190**	88.0631	158.0924	79.5498	1

MS/MS Fragmentation of VLPGVIALDEAIPVTV
Found in gi|15230595, PGK1 (PHOSPHOGLYCERATE KINASE 1); phosphoglycerate kinase [Arabidopsis thaliana]

Match to Query 124: 1684.910208 from(843.462380,2+) intensity(5697.0000)
Title: 155: Sum of 4 scans in range 1891 (rt=45.0774, f=2, i=230) to 1894 (rt=45.1866, f=2, i=233)

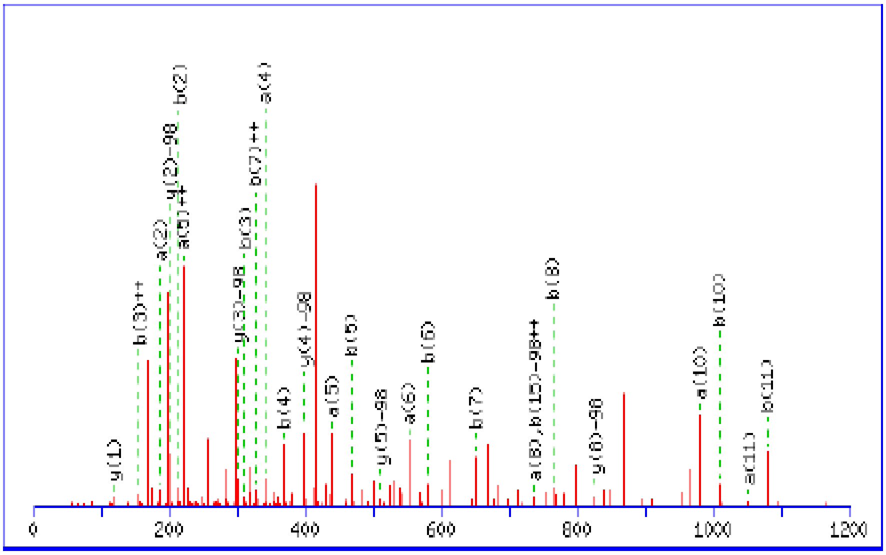

Monoisotopic mass of neutral peptide Mr(calc): 1684.9052
Fixed modifications: Carbamidomethyl (C)
Variable modifications:

T15 : Phospho (ST), with neutral losses 97.9769(shown in table), 0.0000
Ions Score: 33 **Expect:** 1.3
Matches (Bold Red): 26/122 fragment ions using 79 most intense peaks

#	a	a++	b	b++	Seq.	y	y++	#
1	72.0808	36.5440	100.0757	50.5415	V			16
2	**185.1648**	93.0861	**213.1598**	107.0835	L	1488.8672	744.9372	15
3	282.2176	141.6124	**310.2125**	**155.6099**	P	1375.7831	688.3952	14
4	**339.2391**	170.1232	**367.2340**	184.1206	G	1278.7304	639.8688	13
5	**438.3075**	**219.6574**	**466.3024**	233.6548	V	1221.7089	611.3581	12
6	**551.3915**	276.1994	**579.3865**	290.1969	I	1122.6405	561.8239	11
7	622.4287	311.7180	**650.4236**	**325.7154**	A	1009.5564	505.2819	10
8	**735.5127**	368.2600	**763.5076**	382.2575	L	938.5193	469.7633	9
9	850.5397	425.7735	878.5346	439.7709	D	**825.4353**	413.2213	8
10	**979.5823**	490.2948	**1007.5772**	504.2922	E	710.4083	355.7078	7
11	**1050.6194**	525.8133	**1078.6143**	539.8108	A	581.3657	291.1865	6
12	1163.7034	582.3554	1191.6984	596.3528	I	**510.3286**	255.6679	5
13	1260.7562	630.8817	1288.7511	644.8792	P	**397.2445**	199.1259	4
14	1359.8246	680.4159	1387.8195	694.4134	V	**300.1918**	150.5995	3
15	1442.8617	721.9345	1470.8566	**735.9320**	T	**201.1234**	101.0653	2
16					V	**118.0863**	59.5468	1

MS/MS Fragmentation of ANPLATILSAAMLLK
Found in gi|15220167, 3-isopropylmalate dehydrogenase, chloroplast, putative [Arabidopsis thaliana]

Match to Query 119: 1621.855768 from(811.935160,2+) intensity(10443.0000)
Title: 160: Sum of 4 scans in range 2010 (rt=47.7413, f=2, i=244) to 2013 (rt=47.8508, f=2, i=247)

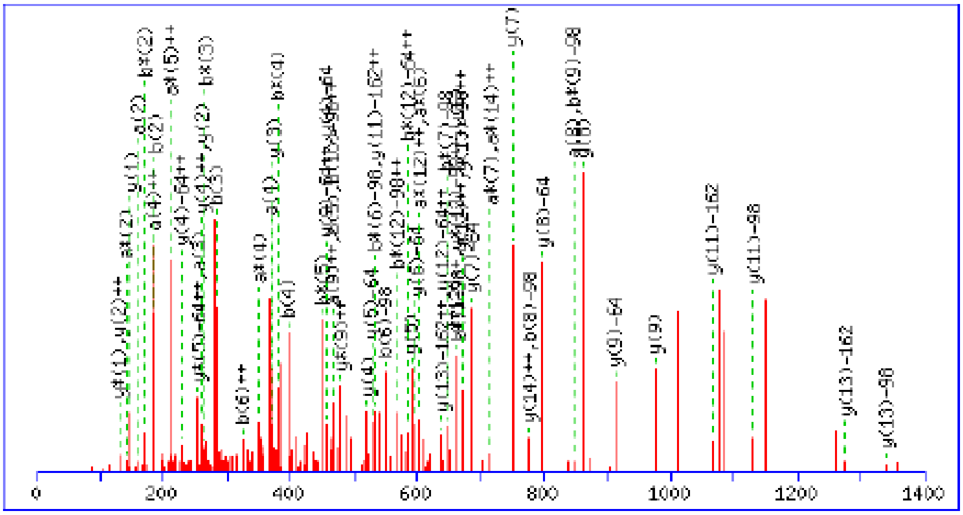

Monoisotopic mass of neutral peptide Mr(calc): 1621.8514
Fixed modifications: Carbamidomethyl (C)
Variable modifications:

T6 : Phospho (ST), with neutral losses 97.9769(shown in table), 0.0000
M12 : Oxidation (M), with neutral losses 63.9983(shown in table), 0.0000
Ions Score: 65 **Expect:** 0.00087
Matches (**Bold Red**): 65/368 fragment ions using 76 most intense peaks

#	a	a++	a*	a*++	b	b++	b*	b*++	Seq.	y	y++	y*	y*++	#
1	44.0495	22.5284			72.0444	36.5258			A					15
2	**158.0924**	79.5498	**141.0659**	71.0366	**186.0873**	93.5473	**169.0608**	85.0340	N	1389.8464	695.4268	1372.8199	686.9136	14
3	**255.1452**	128.0762	238.1186	119.5629	**283.1401**	142.0737	**266.1135**	133.5604	P	**1275.8035**	**638.4054**	1258.7769	629.8921	13
4	**368.2292**	**184.6183**	**351.2027**	176.1050	**396.2241**	198.6157	**379.1976**	190.1024	L	1178.7507	589.8790	1161.7242	581.3657	12
5	439.2663	220.1368	422.2398	**211.6235**	**467.2613**	234.1343	**450.2347**	225.6210	A	**1065.6667**	**533.3370**	1048.6401	524.8237	11
6	522.3035	261.6554	505.2769	253.1421	**550.2984**	275.6528	**533.2718**	267.1395	T	994.6295	497.8184	977.6030	489.3051	10
7	635.3875	318.1974	618.3610	309.6841	**663.3824**	332.1949	**646.3559**	323.6816	I	**911.5924**	**456.2999**	894.5659	447.7866	9
8	748.4716	374.7394	731.4450	366.2262	**776.4665**	388.7369	759.4399	380.2236	L	**798.5084**	399.7578	781.4818	391.2445	8
9	835.5036	418.2554	818.4771	409.7422	863.4985	432.2529	**846.4720**	423.7396	S	**685.4243**	343.2158	668.3978	334.7025	7
10	906.5407	453.7740	889.5142	445.2607	934.5356	**467.7715**	917.5091	459.2582	A	**598.3923**	299.6998	581.3657	291.1865	6
11	977.5778	489.2926	960.5513	480.7793	1005.5727	503.2900	988.5462	494.7767	A	**527.3552**	264.1812	510.3286	**255.6679**	5
12	1060.6150	530.8111	1043.5884	522.2978	1088.6099	544.8086	1071.5833	536.2953	M	**456.3181**	**228.6627**	439.2915	220.1494	4
13	1173.6990	587.3531	1156.6725	578.8399	1201.6939	601.3506	1184.6674	592.8373	L	**373.2809**	187.1441	356.2544	178.6308	3
14	1286.7831	643.8952	1269.7565	635.3819	1314.7780	657.8926	1297.7514	649.3794	L	**260.1969**	**130.6021**	243.1703	122.0888	2
15									K	**147.1128**	74.0600	**130.0863**	65.5468	1

17a

MS/MS Fragmentation of SSDGKLFVDILK
Found in gi|15231715, fructose-bisphosphate aldolase, putative [Arabidopsis thaliana]

Match to Query 82: 1400.697908 from(701.356230,2+) intensity(12635.0000)
Title: 126: Sum of 3 scans in range 1692 (rt=39.5577, f=2, i=168) to 1694 (rt=39.6331, f=2, i=170)

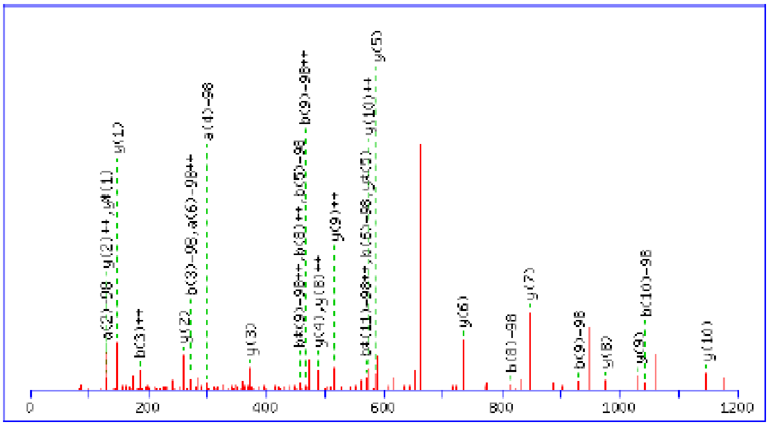

Monoisotopic mass of neutral peptide Mr(calc): 1400.6952
Fixed modifications: Carbamidomethyl (C)
Variable modifications:

S2 : Phospho (ST), with neutral losses 97.9769(shown in table), 0.0000
Ions Score: 64 **Expect:** 0.001
Matches (Bold Red): 30/188 fragment ions using 41 most intense peaks

#	a	a++	a*	a*++	b	b++	b*	b*++	Seq.	y	y++	y*	y*++	#
1	60.0444	30.5258			88.0393	44.5233			S					12
2	**129.0658**	65.0366			157.0608	79.0340			S	1216.6936	608.8504	1199.6670	600.3372	11
3	244.0928	122.5500			**272.0877**	136.5475			D	**1147.6721**	**574.3397**	1130.6456	565.8264	10
4	**301.1143**	151.0608			329.1092	165.0582			G	**1032.6452**	**516.8262**	1015.6186	508.3130	9
5	429.2092	215.1082	412.1827	206.5950	**457.2041**	229.1057	440.1776	220.5924	K	**975.6237**	**488.3155**	958.5972	479.8022	8
6	542.2933	**271.6503**	525.2667	263.1370	**570.2882**	285.6477	553.2616	277.1345	L	**847.5288**	424.2680	830.5022	415.7547	7
7	689.3617	345.1845	672.3351	336.6712	717.3566	359.1819	700.3301	350.6687	F	**734.4447**	367.7260	717.4182	359.2127	6
8	788.4301	394.7187	771.4036	386.2054	**816.4250**	408.7161	799.3985	400.2029	V	**587.3763**	294.1918	**570.3497**	285.6785	5
9	903.4570	452.2322	886.4305	443.7189	**931.4520**	**466.2296**	914.4254	**457.7163**	D	**488.3079**	244.6576	471.2813	236.1443	4
10	1016.5411	508.7742	999.5146	500.2609	**1044.5360**	522.7717	1027.5095	514.2584	I	**373.2809**	187.1441	356.2544	178.6308	3
11	1129.6252	565.3162	1112.5986	556.8030	1157.6201	579.3137	1140.5935	**570.8004**	L	**260.1969**	**130.6021**	243.1703	122.0888	2
12									K	**147.1128**	74.0600	**130.0863**	65.5468	1

17b

MS/MS Fragmentation of ANSEATLGTYKGDAK
Found in gi|15231715, fructose-bisphosphate aldolase, putative [Arabidopsis thaliana]

Match to Query 110: 1604.715748 from(803.365150,2+) intensity(3611.0000)
Title: 33: Sum of 3 scans in range 1295 (rt=26.4849, f=2, i=61) to 1301 (rt=26.7332, f=2, i=63)

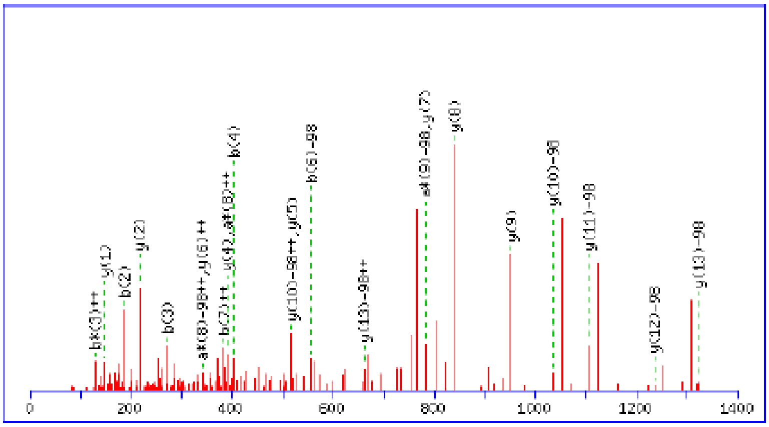


```
Monoisotopic mass of neutral peptide Mr(calc): 1604.7083
Fixed modifications: Carbamidomethyl (C)
Variable modifications:

T6      : Phospho (ST), with neutral losses 97.9769(shown in table), 0.0000
Ions Score: 71  Expect: 0.00021
Matches (Bold Red): 23/256 fragment ions using 39 most intense peaks
```

#	a	a^{++}	a^{*}	a^{*++}	b	b^{++}	b^{*}	b^{*++}	Seq.	y	y^{++}	y^{*}	y^{*++}	#
1	44.0495	22.5284			72.0444	36.5258			A					15
2	158.0924	79.5498	141.0659	71.0366	**186.0873**	93.5473	169.0608	85.0340	N	1436.7016	718.8544	1419.6750	710.3412	14
3	245.1244	123.0659	228.0979	114.5526	**273.1193**	137.0633	256.0928	**128.5500**	S	**1322.6587**	**661.8330**	1305.6321	653.3197	13
4	374.1670	187.5871	357.1405	179.0739	**402.1619**	201.5846	385.1354	193.0713	E	**1235.6266**	618.3170	1218.6001	609.8037	12
5	445.2041	223.1057	428.1776	214.5924	473.1991	237.1032	456.1725	228.5899	A	**1106.5840**	553.7957	1089.5575	545.2824	11
6	528.2412	264.6243	511.2147	256.1110	**556.2362**	278.6217	539.2096	270.1084	T	**1035.5469**	**518.2771**	1018.5204	509.7638	10
7	641.3253	321.1663	624.2988	312.6530	669.3202	335.1637	652.2937	326.6505	L	**952.5098**	476.7586	935.4833	468.2453	9
8	698.3468	349.6770	681.3202	**341.1637**	726.3417	363.6745	709.3151	355.1612	G	**839.4258**	420.2165	822.3992	411.7032	8
9	799.3945	400.2009	**782.3679**	391.6876	827.3894	414.1983	810.3628	405.6850	T	**782.4043**	391.7058	765.3777	383.1925	7
10	962.4578	481.7325	945.4312	473.2193	990.4527	495.7300	973.4261	487.2167	Y	681.3566	**341.1819**	664.3301	332.6687	6
11	1090.5527	545.7800	1073.5262	537.2667	1118.5477	559.7775	1101.5211	551.2642	K	**518.2933**	259.6503	501.2667	251.1370	5
12	1147.5742	574.2907	1130.5477	565.7775	1175.5691	588.2882	1158.5426	579.7749	G	**390.1983**	195.6028	373.1718	187.0895	4
13	1262.6012	631.8042	1245.5746	623.2909	1290.5961	645.8017	1273.5695	637.2884	D	333.1769	167.0921	316.1503	158.5788	3
14	1333.6383	667.3228	1316.6117	658.8095	1361.6332	681.3202	1344.6066	672.8070	A	**218.1499**	109.5786	201.1234	101.0653	2
15									K	**147.1128**	74.0600	130.0863	65.5468	1

MS/MS Fragmentation of IGENEPSEHSIHENAYGLAR
Found in gi|15231715, fructose-bisphosphate aldolase, putative [Arabidopsis thaliana]

Match to Query 143: 2301.993132 from(768.338320,3+) intensity(10816.0000)

Title: 54: Scan 1365 (rt=28.9821, f=3, i=43)

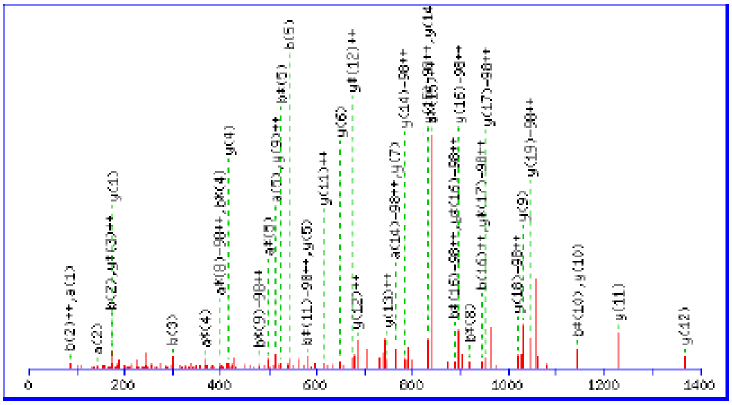

Monoisotopic mass of neutral peptide Mr(calc): 2302.0015
Fixed modifications: Carbamidomethyl (C)
Variable modifications:
S7 : Phospho (ST), with neutral losses 97.9769(shown in table), 0.0000
Ions Score: 52 **Expect:** 0.018
Matches (Bold Red): 44/344 fragment ions using 72 most intense peaks

#	a	a++	a*	a*++	b	b++	b*	b*++	Seq.	y	y++	y*	y*++	#
1	**86.0964**	43.5519			114.0913	57.5493			I					20
2	**143.1179**	72.0626			**171.1128**	**86.0600**			G	2091.9479	**1046.4776**	2074.9213	1037.9643	19
3	272.1605	136.5839			**300.1554**	150.5813			E	2034.9264	**1017.9668**	2017.8998	1009.4536	18
4	386.2034	193.6053	**369.1769**	185.0921	414.1983	207.6028	**397.1718**	199.0895	N	1905.8838	**953.4455**	1888.8573	**944.9323**	17
5	**515.2460**	258.1266	**498.2195**	249.6134	**543.2409**	272.1241	**526.2144**	263.6108	E	1791.8409	**896.4241**	1774.8143	**887.9108**	16
6	612.2988	306.6530	595.2722	298.1397	640.2937	320.6505	623.2671	312.1372	P	1662.7983	**831.9028**	1645.7717	823.3895	15
7	681.3202	341.1637	664.2937	332.6505	709.3151	355.1612	692.2886	346.6479	S	1565.7455	**783.3764**	1548.7190	774.8631	14
8	810.3628	405.6850	793.3363	**397.1718**	838.3577	419.6825	821.3312	411.1692	E	1496.7241	**748.8657**	1479.6975	740.3524	13
9	947.4217	474.2145	930.3952	465.7012	975.4166	488.2120	958.3901	**479.6987**	H	**1367.6815**	**684.3444**	1350.6549	**675.8311**	12
10	1034.4538	517.7305	1017.4272	509.2172	1062.4487	531.7280	1045.4221	523.2147	S	**1230.6226**	**615.8149**	1213.5960	607.3016	11
11	1147.5378	574.2725	1130.5113	565.7593	1175.5327	588.2700	1158.5062	**579.7567**	I	**1143.5905**	572.2989	1126.5640	563.7856	10
12	1284.5967	642.8020	1267.5702	634.2887	1312.5916	656.7995	1295.5651	648.2862	H	**1030.5065**	**515.7569**	1013.4799	507.2436	9
13	1413.6393	707.3233	1396.6128	698.8100	1441.6342	721.3208	1424.6077	712.8075	E	893.4476	447.2274	876.4210	438.7141	8
14	1527.6823	**764.3448**	1510.6557	755.8315	1555.6772	778.3422	1538.6506	769.8289	N	**764.4050**	382.7061	747.3784	374.1928	7
15	1598.7194	799.8633	1581.6928	791.3500	1626.7143	813.8608	1609.6877	805.3475	A	**650.3620**	325.6847	633.3355	317.1714	6
16	1761.7827	881.3950	1744.7561	872.8817	1789.7776	895.3924	1772.7511	**886.8792**	Y	**579.3249**	290.1661	562.2984	281.6528	5
17	1818.8042	909.9057	1801.7776	901.3924	1846.7991	923.9032	1829.7725	915.3899	G	**416.2616**	208.6344	399.2350	200.1212	4
18	1931.8882	966.4477	1914.8617	957.9345	1959.8831	980.4452	1942.8566	971.9319	L	359.2401	180.1237	342.2136	**171.6104**	3
19	2002.9253	1001.9663	1985.8988	993.4530	2030.9203	1015.9638	2013.8937	1007.4505	A	246.1561	123.5817	229.1295	115.0684	2
20									R	**175.1190**	88.0631	158.0924	79.5498	1

MS/MS Fragmentation of TVPAAVPAIVFLSGGQSEEEATR
Found in gi|15231715, fructose-bisphosphate aldolase, putative [Arabidopsis thaliana]

Match to Query 146: 2408.166048 from(1205.090300,2+) intensity(17010.0000)
Title: 144: Sum of 3 scans in range 1793 (rt=42.5555, f=2, i=205) to 1795 (rt=42.6231, f=2, i=207)

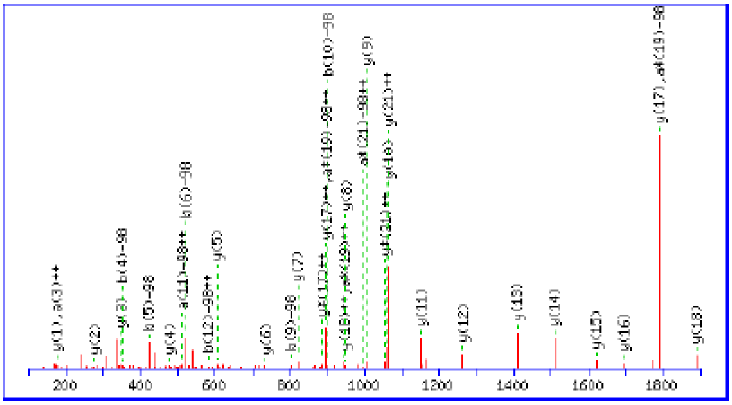

Monoisotopic mass of neutral peptide Mr(calc): 2408.1625
Fixed modifications: Carbamidomethyl (C)
Variable modifications:
T1 : Phospho (ST), with neutral losses 97.9769(shown in table), 0.0000
Ions Score: 124 **Expect:** 9.9e-10
Matches (**Bold Red**): 35/320 fragment ions using 57 most intense peaks

#	a	a⁺⁺	a*	a*⁺⁺	b	b⁺⁺	b*	b*⁺⁺	Seq.	y	y⁺⁺	y*	y*⁺⁺	#
1	56.0495	28.5284			84.0444	42.5258			T					23
2	155.1179	78.0626			183.1128	92.0600			V	2228.1557	1114.5815	2211.1292	1106.0682	22
3	252.1706	126.5890			280.1656	140.5864			P	2129.0873	**1065.0473**	2112.0608	**1056.5340**	21
4	323.2078	162.1075			**351.2027**	176.1050			A	2032.0346	1016.5209	2015.0080	1008.0076	20
5	394.2449	197.6261			**422.2398**	211.6235			A	1960.9974	981.0024	1943.9709	972.4891	19
6	493.3133	247.1603			**521.3082**	261.1577			V	**1889.9603**	**945.4838**	1872.9338	936.9705	18
7	590.3661	295.6867			618.3610	309.6841			P	**1790.8919**	**895.9496**	1773.8654	**887.4363**	17
8	661.4032	331.2052			689.3981	345.2027			A	**1693.8392**	847.4232	1676.8126	838.9099	16
9	774.4872	387.7473			**802.4821**	401.7447			I	**1622.8020**	811.9047	1605.7755	803.3914	15
10	873.5556	437.2815			**901.5506**	451.2789			V	**1509.7180**	755.3626	1492.6914	746.8494	14
11	1020.6241	**510.8157**			1048.6190	524.8131			F	**1410.6496**	705.8284	1393.6230	697.3151	13
12	1133.7081	567.3577			1161.7030	**581.3552**			L	**1263.5812**	632.2942	1246.5546	623.7809	12
13	1220.7402	610.8737			1248.7351	624.8712			S	**1150.4971**	575.7522	1133.4705	567.2389	11
14	1277.7616	639.3844			1305.7565	653.3819			G	**1063.4651**	532.2362	1046.4385	523.7229	10
15	1334.7831	667.8952			1362.7780	681.8926			G	**1006.4436**	503.7254	989.4170	495.2122	9
16	1462.8417	731.9245	1445.8151	723.4112	1490.8366	745.9219	1473.8100	737.4086	Q	**949.4221**	475.2147	932.3956	466.7014	8
17	1549.8737	775.4405	1532.8471	766.9272	1577.8686	789.4379	1560.8420	780.9247	S	**821.3636**	411.1854	804.3370	402.6721	7
18	1678.9163	839.9618	1661.8897	831.4485	1706.9112	853.9592	1689.8846	845.4460	E	**734.3315**	367.6694	717.3050	359.1561	6
19	1807.9589	904.4831	**1790.9323**	**895.9698**	1835.9538	918.4805	1818.9272	909.9673	E	**605.2889**	303.1481	588.2624	294.6348	5
20	1937.0015	969.0044	1919.9749	960.4911	1964.9964	983.0018	1947.9698	974.4886	E	**476.2463**	238.6268	459.2198	230.1135	4
21	2008.0386	1004.5229	1991.0120	**996.0097**	2036.0335	1018.5204	2019.0069	1010.0071	A	**347.2037**	174.1055	330.1772	165.5922	3
22	2109.0863	1055.0468	2092.0597	1046.5335	2137.0812	1069.0442	2120.0546	1060.5309	T	**276.1666**	138.5870	259.1401	130.0737	2
23									R	**175.1190**	88.0631	158.0924	79.5498	1

MS/MS Fragmentation of SSDGKLFVDILK
Found in gi|15231715, fructose-bisphosphate aldolase, putative [Arabidopsis thaliana]

Match to Query 92: 1400.741228 from(701.377890,2+) intensity(4884.0000)
Title: 126: Sum of 3 scans in range 1694 (rt=39.554, f=3, i=117) to 1698 (rt=39.7151, f=3, i=119)

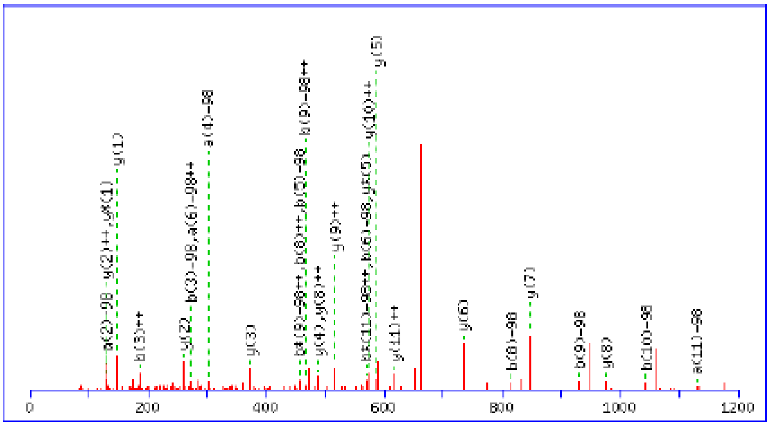


```
Monoisotopic mass of neutral peptide Mr(calc): 1400.6952
Fixed modifications: Carbamidomethyl (C)
Variable modifications:

S1     : Phospho (ST), with neutral losses 97.9769(shown in table), 0.0000
Ions Score: 46  Expect: 0.075
Matches (Bold Red): 30/188 fragment ions using 47 most intense peaks
```

#	a	a++	a*	a*++	b	b++	b*	b*++	Seq.	y	y++	y*	y*++	#
1	42.0338	21.5205			70.0287	35.5180			S					12
2	**129.0658**	65.0366			157.0608	79.0340			S	1234.7042	**617.8557**	1217.6776	609.3424	11
3	244.0928	122.5500			**272.0877**	136.5475			D	1147.6721	**574.3397**	1130.6456	565.8264	10
4	**301.1143**	151.0608			329.1092	165.0582			G	1032.6452	**516.8262**	1015.6186	508.3130	9
5	429.2092	215.1082	412.1827	206.5950	**457.2041**	229.1057	440.1776	220.5924	K	**975.6237**	**488.3155**	958.5972	479.8022	8
6	542.2933	**271.6503**	525.2667	263.1370	**570.2882**	285.6477	553.2616	277.1345	L	**847.5288**	424.2680	830.5022	415.7547	7
7	689.3617	345.1845	672.3351	336.6712	717.3566	359.1819	700.3301	350.6687	F	**734.4447**	367.7260	717.4182	359.2127	6
8	788.4301	394.7187	771.4036	386.2054	**816.4250**	408.7161	799.3985	400.2029	V	**587.3763**	294.1918	**570.3497**	285.6785	5
9	903.4570	452.2322	886.4305	443.7189	**931.4520**	**466.2296**	914.4254	**457.7163**	D	**488.3079**	244.6576	471.2813	236.1443	4
10	1016.5411	508.7742	999.5146	500.2609	**1044.5360**	522.7717	1027.5095	514.2584	I	**373.2809**	187.1441	356.2544	178.6308	3
11	**1129.6252**	565.3162	1112.5986	556.8030	1157.6201	579.3137	1140.5935	**570.8004**	L	**260.1969**	**130.6021**	243.1703	122.0888	2
12									K	**147.1128**	74.0600	**130.0863**	65.5468	1

MS/MS Fragmentation of TLLFGEKPVTVFGIR
Found in gi|15222848, GAPC-2; glyceraldehyde-3-phosphate dehydrogenase [Arabidopsis thaliana]

Match to Query 101: 1755.922828 from(878.968690,2+) intensity(11038.0000)
Title: 122: Sum of 4 scans in range 1651 (rt=40.2666, f=2, i=176) to 1654 (rt=40.3684, f=2, i=179)

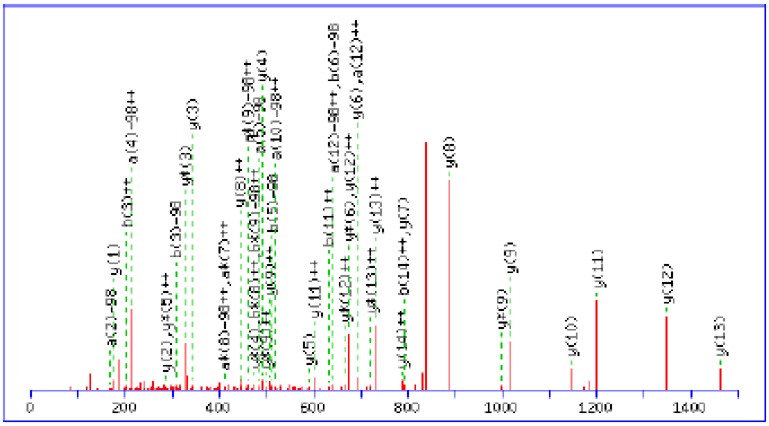

Monoisotopic mass of neutral peptide Mr(calc): 1755.9325
Fixed modifications: Carbamidomethyl (C)
Variable modifications:

T1 : Phospho (ST), with neutral losses 97.9769(shown in table), 0.0000
Ions Score: 83 **Expect:** 1.3e-05
Matches (Bold Red): 44/232 fragment ions using 65 most intense peaks

#	a	a^{++}	a^{*}	a^{*++}	b	b^{++}	b^{*}	b^{*++}	Seq.	y	y^{++}	y^{*}	y^{*++}	#
1	56.0495	28.5284			84.0444	42.5258			T					15
2	**169.1335**	85.0704			197.1284	99.0679			L	1575.9257	**788.4665**	1558.8992	779.9532	14
3	282.2176	141.6124			**310.2125**	155.6099			L	**1462.8417**	**731.9245**	1445.8151	**723.4112**	13
4	429.2860	**215.1466**			457.2809	229.1441			F	**1349.7576**	**675.3824**	1332.7311	**666.8692**	12
5	**486.3075**	243.6574			**514.3024**	257.6548			G	**1202.6892**	**601.8482**	1185.6626	593.3350	11
6	615.3501	308.1787			**643.3450**	322.1761			E	**1145.6677**	573.3375	1128.6412	564.8242	10
7	743.4450	372.2262	726.4185	363.7129	771.4399	386.2236	754.4134	377.7103	K	**1016.6251**	**508.8162**	**999.5986**	**500.3029**	9
8	840.4978	420.7525	823.4712	**412.2393**	868.4927	434.7500	851.4662	426.2367	P	**888.5302**	**444.7687**	871.5036	436.2554	8
9	939.5662	470.2867	922.5397	**461.7735**	967.5611	484.2842	950.5346	**475.7709**	V	**791.4774**	396.2423	774.4509	387.7291	7
10	1040.6139	**520.8106**	1023.5873	512.2973	1068.6088	534.8080	1051.5823	526.2948	T	**692.4090**	346.7081	**675.3824**	338.1949	6
11	1139.6823	570.3448	1122.6558	561.8315	1167.6772	584.3422	1150.6507	575.8290	V	**591.3613**	296.1843	574.3348	**287.6710**	5
12	1286.7507	**643.8790**	1269.7242	635.3657	1314.7456	657.8765	1297.7191	649.3632	F	**492.2929**	246.6501	**475.2663**	238.1368	4
13	1343.7722	672.3897	1326.7456	663.8765	1371.7671	686.3872	1354.7405	677.8739	G	**345.2245**	173.1159	**328.1979**	164.6026	3
14	1456.8562	728.9318	1439.8297	720.4185	1484.8512	742.9292	1467.8246	734.4159	I	**288.2030**	144.6051	271.1765	136.0919	2
15									R	**175.1190**	88.0631	158.0924	79.5498	1

MS/MS Fragmentation of TIEKPVEDPSELPK
Found in gi|15238559, GS2 (GLUTAMINE SYNTHETASE 2); glutamate-ammonia ligase [Arabidopsis thaliana]

Match to Query 90: 1660.860968 from(831.437760,2+) intensity(6347.0000)
Title: 39: Sum of 3 scans in range 1423 (rt=29.3406, f=2, i=49) to 1429 (rt=29.5888, f=2, i=51)

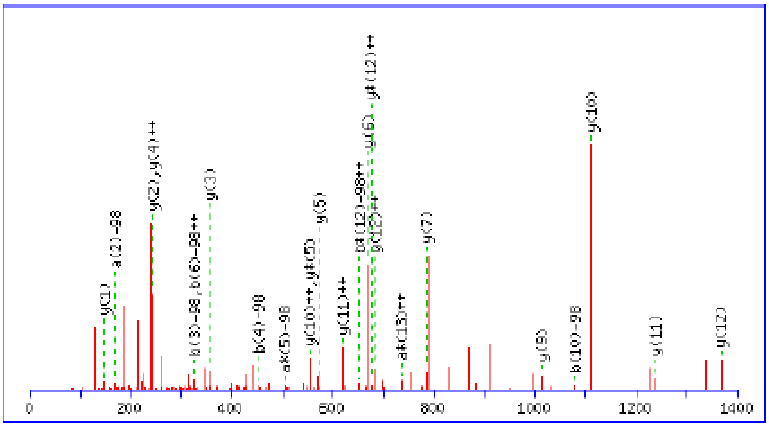

Monoisotopic mass of neutral peptide Mr(calc): 1660.7961
Fixed modifications: Carbamidomethyl (C)
Variable modifications:

T1 : Phospho (ST), with neutral losses 97.9769(shown in table), 0.0000
Ions Score: 44 **Expect:** 0.097
Matches (Bold Red): 24/236 fragment ions using 52 most intense peaks

#	a	a++	a*	a*++	b	b++	b*	b*++	Seq.	y	y++	y*	y*++	#
1	56.0495	28.5284			84.0444	42.5258			T					14
2	**169.1335**	85.0704			197.1284	99.0679			I	1480.7894	740.8983	1463.7628	732.3850	13
3	298.1761	149.5917			**326.1710**	163.5892			E	**1367.7053**	**684.3563**	1350.6787	**675.8430**	12
4	426.2711	213.6392	409.2445	205.1259	**454.2660**	227.6366	437.2395	219.1234	K	**1238.6627**	**619.8350**	1221.6361	611.3217	11
5	523.3239	262.1656	**506.2973**	253.6523	551.3188	276.1630	534.2922	267.6497	P	**1110.5677**	**555.7875**	1093.5412	547.2742	10
6	622.3923	311.6998	605.3657	303.1865	650.3872	**325.6972**	633.3606	317.1840	V	**1013.5150**	507.2611	996.4884	498.7478	9
7	751.4349	376.2211	734.4083	367.7078	779.4298	390.2185	762.4032	381.7053	E	914.4466	457.7269	897.4200	449.2136	8
8	866.4618	433.7345	849.4353	425.2213	894.4567	447.7320	877.4302	439.2187	D	**785.4040**	393.2056	768.3774	384.6923	7
9	963.5146	482.2609	946.4880	473.7476	991.5095	496.2584	974.4829	487.7451	P	**670.3770**	335.6921	653.3505	327.1789	6
10	1050.5466	525.7769	1033.5200	517.2637	**1078.5415**	539.7744	1061.5150	531.2611	S	**573.3243**	287.1658	**556.2977**	278.6525	5
11	1179.5892	590.2982	1162.5626	581.7850	1207.5841	604.2957	1190.5576	595.7824	E	486.2922	**243.6498**	469.2657	235.1365	4
12	1292.6733	646.8403	1275.6467	638.3270	1320.6682	660.8377	1303.6416	**652.3244**	L	**357.2496**	179.1285	340.2231	170.6152	3
13	1389.7260	695.3666	1372.6995	686.8534	1417.7209	709.3641	1400.6944	700.8508	P	**244.1656**	122.5864	227.1390	114.0731	2
14									K	**147.1128**	74.0600	130.0863	65.5468	1

21b

MS/MS Fragmentation of **GGNNILVICDTWTPAGEPIPTNK**
Found in **gi|15238559**, GS2 (GLUTAMINE SYNTHETASE 2); glutamate-ammonia ligase [Arabidopsis thaliana]

Match to Query 111: 2626.423302 from(876.481710,3+) intensity(19331.0000)

Title: 88: Scan 1622 (rt=36.3847, f=2, i=96)

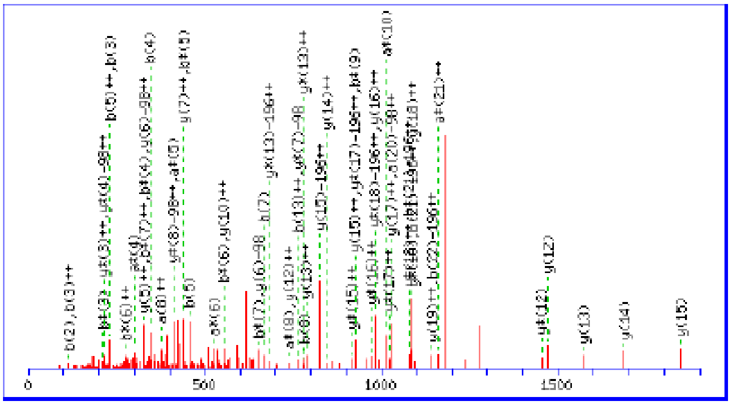


```
Monoisotopic mass of neutral peptide Mr(calc): 2626.1540
Fixed modifications: Carbamidomethyl (C)
Variable modifications:
T13    : Phospho (ST), with neutral losses 0.0000(shown in table), 97.9769
T21    : Phospho (ST), with neutral losses 0.0000(shown in table), 97.9769
Ions Score: 49  Expect: 0.04
Matches (Bold Red): 60/416 fragment ions using 65 most intense peaks
```

#	a	a++	a*	a*++	b	b++	b*	b*++	Seq.	y	y++	y*	y*++	#
1	30.0338	15.5206			58.0287	29.5180			G					23
2	87.0553	44.0313			**115.0502**	58.0287			G	2570.1398	1285.5735	2553.1132	1277.0602	22
3	201.0982	101.0527	184.0717	92.5395	**229.0931**	**115.0502**	**212.0666**	106.5369	N	2513.1183	1257.0628	2496.0918	1248.5495	21
4	315.1411	158.0742	**298.1146**	149.5609	**343.1361**	172.0717	**326.1095**	163.5584	N	2399.0754	1200.0413	2382.0488	1191.5281	20
5	428.2252	214.6162	**411.1987**	206.1030	**456.2201**	**228.6137**	**439.1936**	220.1004	I	2285.0325	**1143.0199**	2268.0059	1134.5066	19
6	541.3093	271.1583	**524.2827**	262.6450	569.3042	285.1557	**552.2776**	**276.6425**	L	2171.9484	**1086.4778**	2154.9218	**1077.9646**	18
7	640.3777	320.6925	623.3511	312.1792	**668.3726**	334.6899	**651.3461**	**326.1767**	V	2058.8643	**1029.9358**	2041.8378	**1021.4225**	17
8	753.4617	**377.2345**	**736.4352**	368.7212	**781.4567**	391.2320	764.4301	382.7187	I	1959.7959	**980.4016**	1942.7694	**971.8883**	16
9	913.4924	457.2498	896.4658	448.7366	941.4873	471.2473	**924.4608**	462.7340	C	**1846.7118**	**923.8596**	1829.6853	**915.3463**	15
10	1028.5193	514.7633	**1011.4928**	506.2500	1056.5143	528.7608	1039.4877	520.2475	D	**1686.6812**	**843.8442**	1669.6547	835.3310	14
11	1129.5670	565.2871	1112.5405	556.7739	1157.5619	579.2846	1140.5354	570.7713	T	**1571.6543**	**786.3308**	1554.6277	**777.8175**	13
12	1315.6463	658.3268	1298.6198	649.8135	1343.6412	672.3243	1326.6147	663.8110	W	**1470.6066**	**735.8069**	**1453.5800**	727.2937	12
13	1496.6603	748.8338	1479.6338	740.3205	1524.6553	**762.8313**	1507.6287	754.3180	T	1284.5273	642.7673	1267.5007	634.2540	11
14	1593.7131	797.3602	1576.6866	788.8469	1621.7080	811.3576	1604.6815	802.8444	P	1103.5133	**552.2603**	**1086.4867**	543.7470	10
15	1664.7502	832.8787	1647.7237	824.3655	1692.7451	846.8762	1675.7186	838.3629	A	1006.4605	503.7339	989.4339	495.2206	9
16	1721.7717	861.3895	1704.7451	852.8762	1749.7666	875.3869	1732.7400	866.8737	G	935.4234	468.2153	918.3968	459.7021	8
17	1850.8143	925.9108	1833.7877	917.3975	1878.8092	939.9082	1861.7826	931.3950	E	878.4019	**439.7046**	861.3754	431.1913	7
18	1947.8670	974.4372	1930.8405	965.9239	1975.8620	988.4346	1958.8354	979.9213	P	749.3593	375.1833	732.3328	366.6700	6
19	2060.9511	1030.9792	2043.9245	1022.4659	2088.9460	1044.9766	2071.9195	1036.4634	I	652.3066	**326.6569**	635.2800	318.1436	5
20	2158.0039	1079.5056	2140.9773	1070.9923	2185.9988	1093.5030	2168.9722	1084.9898	P	539.2225	270.1149	522.1959	261.6016	4
21	2339.0179	1170.0126	2321.9913	**1161.4993**	2367.0128	1184.0100	2349.9862	1175.4968	T	442.1697	221.5885	425.1432	**213.0752**	3
22	2453.0608	1227.0340	2436.0342	1218.5208	2481.0557	1241.0315	2464.0292	1232.5182	N	261.1557	131.0815	244.1292	122.5682	2
23									K	147.1128	74.0600	130.0863	65.5468	1

MS/MS Fragmentation of HADFPGSNNGTGLFQTIVGLK
Found in gi|15222551, PRK (PHOSPHORIBULOKINASE); ATP binding / phosphoribulokinase/ protein binding [Arabidopsis thaliana]

Match to Query 125: 2252.177448 from(1127.096000,2+) intensity(10995.0000)
Title: 125: Sum of 3 scans in range 1793 (rt=42.2086, f=2, i=155) to 1795 (rt=42.284, f=2, i=157)

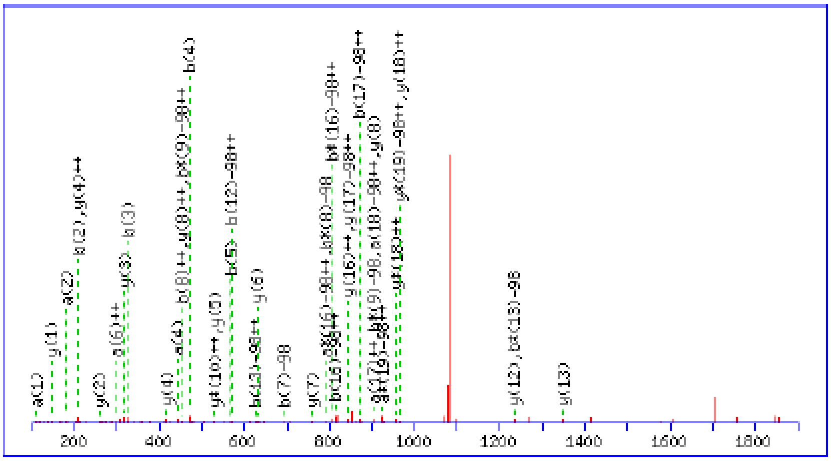

Monoisotopic mass of neutral peptide Mr(calc): 2252.0627
Fixed modifications: Carbamidomethyl (C)
Variable modifications:
S7 : Phospho (ST), with neutral losses 97.9769(shown in table), 0.0000
Ions Score: 48 **Expect:** 0.043

Matches (Bold Red): 41/344 fragment ions using 61 most intense peaks

#	a	a++	a*	a*++	b	b++	b*	b*++	Seq.	y	y++	y*	y*++	#
1	110.0713	55.5393			138.0662	69.5367			H					21
2	181.1084	91.0578			209.1033	105.0553			A	2018.0342	1009.5207	2001.0076	1001.0074	20
3	296.1353	148.5713			324.1302	162.5688			D	1946.9970	974.0022	1929.9705	965.4889	19
4	443.2037	222.1055			471.1987	236.1030			F	1831.9701	916.4887	1814.9436	907.9754	18
5	540.2565	270.6319			568.2514	284.6293			P	1684.9017	842.9545	1667.8751	834.4412	17
6	597.2780	299.1426			625.2729	313.1401			G	1587.8489	794.4281	1570.8224	785.9148	16
7	666.2994	333.6534			694.2943	347.6508			S	1530.8275	765.9174	1513.8009	757.4041	15
8	780.3424	390.6748	763.3158	382.1615	808.3373	404.6723	791.3107	396.1590	N	1461.8060	731.4066	1444.7795	722.8934	14
9	894.3853	447.6963	877.3587	439.1830	922.3802	461.6937	905.3536	453.1805	N	1347.7631	674.3852	1330.7365	665.8719	13
10	951.4067	476.2070	934.3802	467.6937	979.4017	490.2045	962.3751	481.6912	G	1233.7202	617.3637	1216.6936	608.8504	12
11	1052.4544	526.7309	1035.4279	518.2176	1080.4493	540.7283	1063.4228	532.2150	T	1176.6987	588.8530	1159.6721	580.3397	11
12	1109.4759	555.2416	1092.4493	546.7283	1137.4708	569.2390	1120.4443	560.7258	G	1075.6510	538.3291	1058.6245	529.8159	10
13	1222.5600	611.7836	1205.5334	603.2703	1250.5549	625.7811	1233.5283	617.2678	L	1018.6295	509.8184	1001.6030	501.3051	9
14	1369.6284	685.3178	1352.6018	676.8045	1397.6233	699.3153	1380.5967	690.8020	F	905.5455	453.2764	888.5189	444.7631	8
15	1497.6869	749.3471	1480.6604	740.8338	1525.6819	763.3446	1508.6553	754.8313	Q	758.4771	379.7422	741.4505	371.2289	7
16	1598.7346	799.8710	1581.7081	791.3577	1626.7295	813.8684	1609.7030	805.3551	T	630.4185	315.7129	613.3919	307.1996	6
17	1711.8187	856.4130	1694.7921	847.8997	1739.8136	870.4104	1722.7871	861.8972	I	529.3708	265.1890	512.3443	256.6758	5
18	1810.8871	905.9472	1793.8606	897.4339	1838.8820	919.9446	1821.8555	911.4314	V	416.2867	208.6470	399.2602	200.1337	4
19	1867.9086	934.4579	1850.8820	925.9446	1895.9035	948.4554	1878.8769	939.9421	G	317.2183	159.1128	300.1918	150.5995	3
20	1980.9926	991.0000	1963.9661	982.4867	2008.9875	1004.9974	1991.9610	996.4841	L	260.1969	130.6021	243.1703	122.0888	2
21									K	147.1128	74.0600	130.0863	65.5468	1

25a

MS/MS Fragmentation of KLMGVTMLDVVR

Found in gi|15227752, PMDH1 (PEROXISOMAL NAD-MALATE DEHYDROGENASE 1); malate dehydrogenase [Arabidopsis thaliana]

Match to Query 106: 1440.724248 from(721.369400,2+) intensity(8464.0000)
Title: 117: Sum of 2 scans in range 1669 (rt=40.3748, f=3, i=121) to 1672 (rt=40.4975, f=3, i=122)

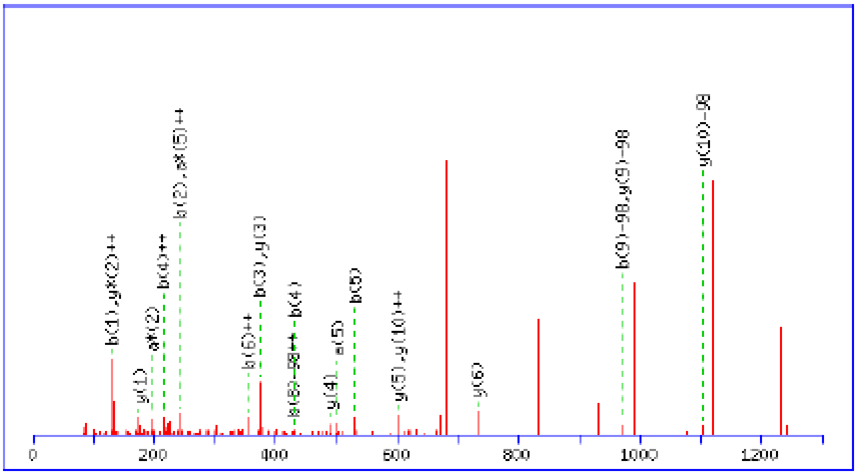

Monoisotopic mass of neutral peptide Mr(calc): 1440.7234
Fixed modifications: Carbamidomethyl (C)
Variable modifications:

T6 : Phospho (ST), with neutral losses 97.9769(shown in table), 0.0000
Ions Score: 45 **Expect:** 0.08
Matches (Bold Red): 21/200 fragment ions using 29 most intense peaks

#	a	a^{++}	a*	a^{*++}	b	b^{++}	b*	b^{*++}	Seq.	y	y^{++}	y*	y^{*++}	#
1	101.1073	51.0573	84.0808	42.5440	**129.1022**	65.0548	112.0757	56.5415	K					12
2	214.1914	107.5993	**197.1648**	99.0861	**242.1863**	121.5968	225.1598	113.0835	L	1215.6588	608.3330	1198.6323	599.8198	11
3	345.2319	173.1196	328.2053	164.6063	**373.2268**	187.1170	356.2002	178.6038	M	**1102.5747**	551.7910	1085.5482	543.2777	10
4	402.2533	201.6303	385.2268	193.1170	**430.2483**	**215.6278**	413.2217	207.1145	G	**971.5343**	486.2708	954.5077	477.7575	9
5	**501.3218**	251.1645	484.2952	**242.6512**	**529.3167**	265.1620	512.2901	256.6487	V	914.5128	457.7600	897.4862	449.2468	8
6	584.3589	292.6831	567.3323	284.1698	612.3538	306.6805	595.3272	298.1673	T	815.4444	408.2258	798.4178	399.7126	7
7	715.3993	358.2033	698.3728	349.6900	743.3943	372.2008	726.3677	363.6875	M	**732.4073**	366.7073	715.3807	358.1940	6
8	828.4834	414.7453	811.4569	406.2321	856.4783	**428.7428**	839.4518	420.2295	L	**601.3668**	301.1870	584.3402	292.6738	5
9	943.5104	472.2588	926.4838	463.7455	**971.5053**	486.2563	954.4787	477.7430	D	**488.2827**	244.6450	471.2562	236.1317	4
10	1042.5788	521.7930	1025.5522	513.2797	1070.5737	535.7905	1053.5471	527.2772	V	**373.2558**	187.1315	356.2292	178.6183	3
11	1141.6472	571.3272	1124.6206	562.8140	1169.6421	585.3247	1152.6155	576.8114	V	274.1874	137.5973	257.1608	**129.0840**	2
12									R	**175.1190**	88.0631	158.0924	79.5498	1

25b

MS/MS Fragmentation of KPGMTRDDLFNINAGIVR
Found in gi|15227752, PMDH1 (PEROXISOMAL NAD-MALATE DEHYDROGENASE 1); malate dehydrogenase [Arabidopsis thaliana]

Match to Query 139: 2111.969592 from(704.997140,3+) intensity(8870.0000)
Title: 85: Sum of 2 scans in range 1558 (rt=36.3227, f=3, i=91) to 1560 (rt=36.4045, f=3, i=92)

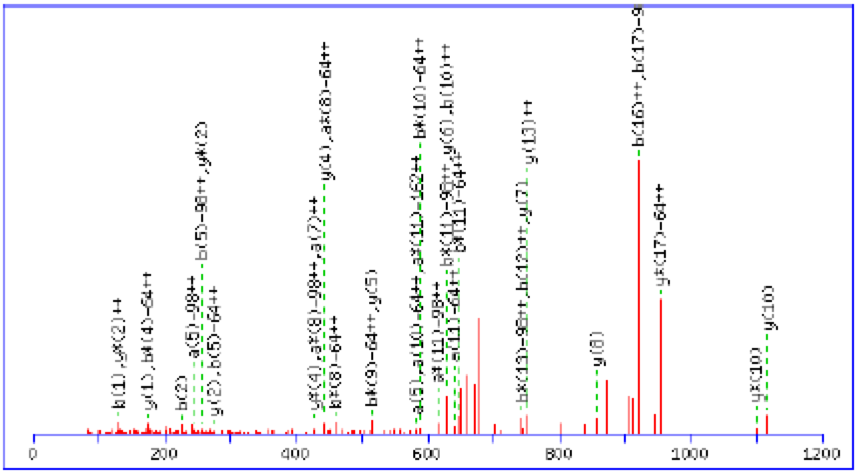

Monoisotopic mass of neutral peptide Mr(calc): 2112.0187
Fixed modifications: Carbamidomethyl (C)
Variable modifications:

M4 : Oxidation (M), with neutral losses 63.9983(shown in table), 0.0000
T5 : Phospho (ST), with neutral losses 0.0000(shown in table), 97.9769
Ions Score: 31 **Expect:** 2.2
Matches (Bold Red): 39/564 fragment ions using 32 most intense peaks

#	a	a++	a*	a*++	b	b++	b*	b*++	Seq.	y	y++	y*	y*++	#
1	101.1073	51.0573	84.0808	42.5440	**129.1022**	65.0548	112.0757	56.5415	K					18
2	198.1601	99.5837	181.1335	91.0704	**226.1550**	113.5811	209.1285	105.0679	P	1920.9328	960.9700	1903.9062	**952.4567**	17
3	255.1816	128.0944	238.1550	119.5811	283.1765	142.0919	266.1499	133.5786	G	1823.8800	912.4436	1806.8534	903.9304	16
4	338.2187	169.6130	321.1921	161.0997	366.2136	183.6104	349.1870	**175.0972**	M	1766.8585	883.9329	1749.8320	875.4196	15
5	519.2327	260.1200	502.2061	251.6067	547.2276	**274.1174**	530.2010	265.6042	T	1683.8214	842.4143	1666.7949	833.9011	14
6	675.3338	338.1705	658.3072	329.6573	703.3287	352.1680	686.3022	343.6547	R	1502.8074	**751.9073**	1485.7809	743.3941	13
7	790.3607	395.6840	773.3342	387.1707	818.3556	409.6815	801.3291	401.1682	D	1346.7063	673.8568	1329.6797	665.3435	12
8	905.3877	453.1975	888.3611	**444.6842**	933.3826	467.1949	916.3560	**458.6817**	D	1231.6793	616.3433	1214.6528	607.8300	11
9	1018.4717	509.7395	1001.4452	501.2262	1046.4666	523.7370	1029.4401	**515.2237**	L	**1116.6524**	558.8298	**1099.6259**	550.3166	10
10	1165.5401	**583.2737**	1148.5136	574.7604	1193.5351	597.2712	1176.5085	**588.7579**	F	1003.5683	502.2878	986.5418	493.7745	9
11	1279.5831	**640.2952**	1262.5565	631.7819	1307.5780	654.2926	1290.5514	**645.7794**	N	**856.4999**	428.7536	839.4734	420.2403	8
12	1392.6671	696.8372	1375.6406	688.3239	1420.6621	710.8347	1403.6355	702.3214	I	**742.4570**	371.7321	725.4304	363.2189	7
13	1506.7101	753.8587	1489.6835	745.3454	1534.7050	767.8561	1517.6784	759.3429	N	**629.3729**	315.1901	612.3464	306.6768	6
14	1577.7472	789.3772	1560.7206	780.8640	1605.7421	803.3747	1588.7155	794.8614	A	**515.3300**	258.1686	498.3035	249.6554	5
15	1634.7686	817.8880	1617.7421	809.3747	1662.7636	831.8854	1645.7370	823.3721	G	**444.2929**	222.6501	**427.2663**	214.1368	4
16	1747.8527	874.4300	1730.8262	865.9167	1775.8476	888.4274	1758.8211	879.9142	I	387.2714	194.1394	370.2449	185.6261	3
17	1846.9211	923.9642	1829.8946	915.4509	1874.9160	937.9617	1857.8895	929.4484	V	**274.1874**	137.5973	**257.1608**	**129.0840**	2
18									R	**175.1190**	88.0631	158.0924	79.5498	1

26a

MS/MS Fragmentation of SPEEVTGEEHGK
Found in gi|15228198, PBP1 (PYK10-BINDING PROTEIN 1) [Arabidopsis thaliana]

Match to Query 55: 1377.607008 from(689.810780,2+) intensity(3880.0000)
Title: 8: Sum of 4 scans in range 1062 (rt=20.7834, f=2, i=28) to 1065 (rt=20.8861, f=2, i=31)

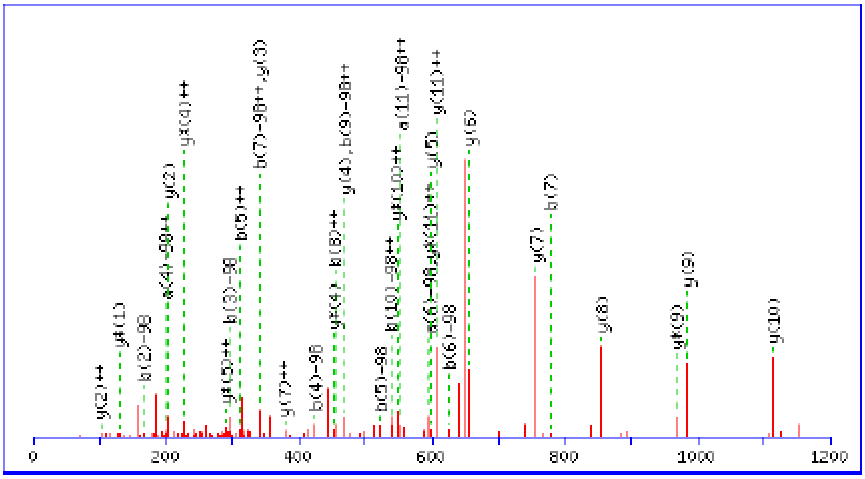

Monoisotopic mass of neutral peptide Mr(calc): 1377.5449
Fixed modifications: Carbamidomethyl (C)
Variable modifications:

S1 : Phospho (ST), with neutral losses 97.9769(shown in table), 0.0000
Ions Score: 50 **Expect:** 0.023
Matches (Bold Red): 33/132 fragment ions using 57 most intense peaks

#	a	a++	b	b++	Seq.	y	y++	y*	y*++	#
1	42.0338	21.5205	70.0287	35.5180	S					12
2	139.0866	70.0469	**167.0815**	84.0444	P	1211.5539	**606.2806**	1194.5273	**597.7673**	11
3	268.1292	134.5682	**296.1241**	148.5657	E	**1114.5011**	557.7542	1097.4746	**549.2409**	10
4	397.1718	**199.0895**	**425.1667**	213.0870	E	**985.4585**	493.2329	**968.4320**	484.7196	9
5	496.2402	248.6237	**524.2351**	262.6212	V	**856.4159**	428.7116	839.3894	420.1983	8
6	**597.2879**	299.1476	**625.2828**	313.1450	T	**757.3475**	**379.1774**	740.3210	370.6641	7
7	654.3093	327.6583	682.3042	**341.6558**	G	**656.2998**	328.6536	639.2733	320.1403	6
8	783.3519	392.1796	811.3468	406.1771	E	**599.2784**	300.1428	582.2518	**291.6295**	5
9	912.3945	456.7009	940.3894	**470.6984**	E	**470.2358**	235.6215	**453.2092**	**227.1082**	4
10	1049.4534	525.2303	1077.4483	**539.2278**	H	**341.1932**	171.1002	324.1666	162.5870	3
11	1106.4749	**553.7411**	1134.4698	567.7385	G	**204.1343**	**102.5708**	187.1077	94.0575	2
12					K	147.1128	74.0600	**130.0863**	65.5468	1

26b

MS/MS Fragmentation of QTSPPFGLEAGTVFELKEEGHK
Found in gi|15228198, PBP1 (PYK10-BINDING PROTEIN 1) [Arabidopsis thaliana]

Match to Query 124: 2480.264772 from(827.762200,3+) intensity(4544.0000)
Title: 98: Sum of 4 scans in range 1472 (rt=35.3596, f=2, i=139) to 1475 (rt=35.4622, f=2, i=142)

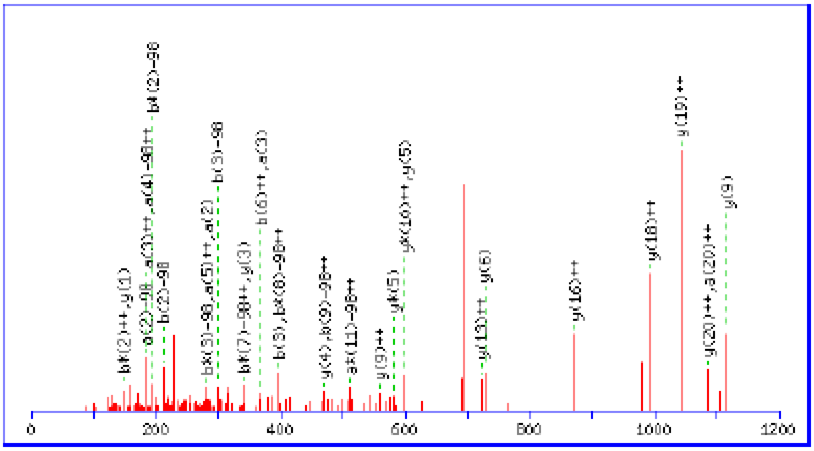


```
Monoisotopic mass of neutral peptide Mr(calc): 2480.1625
Fixed modifications: Carbamidomethyl (C)
Variable modifications:
T2      : Phospho (ST), with neutral losses 97.9769(shown in table), 0.0000
Ions Score: 36  Expect: 0.77
Matches (Bold Red): 32/416 fragment ions using 35 most intense peaks
```

#	a	a^{++}	a^*	a^{*++}	b	b^{++}	b^*	b^{*++}	Seq.	y	y^{++}	y^*	y^{*++}	#
1	101.0709	51.0391	84.0444	42.5258	129.0659	65.0366	112.0393	56.5233	Q					22
2	**184.1080**	92.5577	167.0815	84.0444	**212.1030**	106.5551	**195.0764**	98.0418	T	2255.1343	1128.0708	2238.1077	1119.5575	21
3	271.1401	136.0737	254.1135	127.5604	**299.1350**	150.0711	**282.1084**	141.5579	S	2172.0972	**1086.5522**	2155.0706	1078.0389	20
4	368.1928	**184.6001**	351.1663	176.0868	396.1878	198.5975	379.1612	190.0842	P	2085.0651	**1043.0362**	2068.0386	1034.5229	19
5	465.2456	233.1264	448.2191	224.6132	493.2405	247.1239	476.2140	238.6106	P	1988.0124	**994.5098**	1970.9858	985.9965	18
6	612.3140	306.6606	595.2875	298.1474	640.3089	320.6581	623.2824	312.1448	F	1890.9596	945.9834	1873.9331	937.4702	17
7	669.3355	335.1714	652.3089	326.6581	697.3304	349.1688	680.3038	**340.6556**	G	1743.8912	**872.4492**	1726.8646	863.9360	16
8	782.4195	391.7134	765.3930	383.2001	810.4145	405.7109	793.3879	**397.1976**	L	1686.8697	843.9385	1669.8432	835.4252	15
9	911.4621	456.2347	894.4356	447.7214	939.4571	**470.2322**	922.4305	461.7189	E	1573.7857	787.3965	1556.7591	778.8832	14
10	982.4993	491.7533	965.4727	483.2400	1010.4942	505.7507	993.4676	497.2374	A	1444.7431	**722.8752**	1427.7165	714.3619	13
11	1039.5207	520.2640	1022.4942	**511.7507**	1067.5156	534.2615	1050.4891	525.7482	G	1373.7060	687.3566	1356.6794	678.8433	12
12	1140.5684	570.7878	1123.5418	562.2746	1168.5633	584.7853	1151.5368	576.2720	T	1316.6845	658.8459	1299.6579	650.3326	11
13	1239.6368	620.3220	1222.6103	611.8088	1267.6317	634.3195	1250.6052	625.8062	V	1215.6368	608.3220	1198.6103	**599.8088**	10
14	1386.7052	693.8562	1369.6787	685.3430	1414.7001	707.8537	1397.6736	699.3404	F	**1116.5684**	**558.7878**	1099.5419	550.2746	9
15	1515.7478	758.3775	1498.7213	749.8643	1543.7427	772.3750	1526.7162	763.8617	E	969.5000	485.2536	952.4734	476.7404	8
16	1628.8319	814.9196	1611.8053	806.4063	1656.8268	828.9170	1639.8002	820.4038	L	840.4574	420.7323	823.4308	412.2191	7
17	1756.9268	878.9671	1739.9003	870.4538	1784.9218	892.9645	1767.8952	884.4512	K	**727.3733**	364.1903	710.3468	355.6770	6
18	1885.9694	943.4884	1868.9429	934.9751	1913.9644	957.4858	1896.9378	948.9725	E	**599.2784**	300.1428	**582.2518**	291.6295	5
19	2015.0120	1008.0097	1997.9855	999.4964	2043.0069	1022.0071	2025.9804	1013.4938	E	**470.2358**	235.6215	453.2092	227.1082	4
20	2072.0335	1036.5204	2055.0069	1028.0071	2100.0284	1050.5178	2083.0019	1042.0046	G	**341.1932**	171.1002	324.1666	162.5870	3
21	2209.0924	1105.0498	2192.0659	1096.5366	2237.0873	1119.0473	2220.0608	1110.5340	H	284.1717	142.5895	267.1452	134.0762	2
22									K	**147.1128**	74.0600	130.0863	65.5468	1

MS/MS Fragmentation of LHACISPSENGLINGK
Found in gi|18405982, avirulence-responsive protein, putative / avirulence induced gene (AIG) protein, putative [Arabidopsis thaliana]

Match to Query 73: 1788.854888 from(895.434720,2+) intensity(9634.0000)
Title: 44: Sum of 6 scans in range 1420 (rt=30.3695, f=2, i=108) to 1427 (rt=30.6565, f=3, i=49)

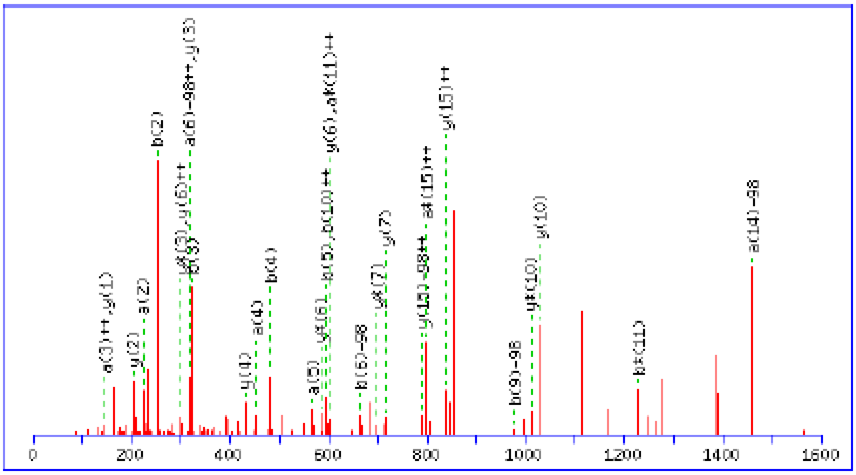

Monoisotopic mass of neutral peptide Mr(calc): 1788.8230
Fixed modifications: Carbamidomethyl (C)
Variable modifications:

S6 : Phospho (ST), with neutral losses 97.9769(shown in table), 0.0000
Ions Score: 39 **Expect:** 0.35
Matches (Bold Red)**:** 30/228 fragment ions using 48 most intense peaks

#	a	a++	a*	a*++	b	b++	b*	b*++	Seq.	y	y++	y*	y*++	#
1	86.0964	43.5519			114.0913	57.5493			L					16
2	**223.1553**	112.0813			**251.1503**	126.0788			H	1578.7693	**789.8883**	1561.7427	781.3750	15
3	294.1925	**147.5999**			**322.1874**	161.5973			A	1441.7104	721.3588	1424.6838	712.8456	14
4	**454.2231**	227.6152			**482.2180**	241.6126			C	1370.6733	685.8403	1353.6467	677.3270	13
5	**567.3072**	284.1572			**595.3021**	298.1547			I	1210.6426	605.8249	1193.6161	597.3117	12
6	636.3286	**318.6679**			**664.3235**	332.6654			S	1097.5586	549.2829	1080.5320	540.7696	11
7	733.3814	367.1943			761.3763	381.1918			P	**1028.5371**	514.7722	**1011.5105**	506.2589	10
8	820.4134	410.7103			848.4083	424.7078			S	931.4843	466.2458	914.4578	457.7325	9
9	949.4560	475.2316			**977.4509**	489.2291			E	844.4523	422.7298	827.4258	414.2165	8
10	1063.4989	532.2531	1046.4724	523.7398	1091.4938	546.2506	1074.4673	537.7373	N	**715.4097**	358.2085	**698.3832**	349.6952	7
11	1120.5204	560.7638	1103.4938	552.2506	1148.5153	574.7613	1131.4888	566.2480	G	**601.3668**	**301.1870**	**584.3402**	292.6738	6
12	1233.6045	617.3059	1216.5779	608.7926	1261.5994	631.3033	1244.5728	622.7901	L	544.3453	272.6763	527.3188	264.1630	5
13	1346.6885	673.8479	1329.6620	665.3346	1374.6834	687.8454	1357.6569	679.3321	I	**431.2613**	216.1343	414.2347	207.6210	4
14	**1460.7315**	730.8694	1443.7049	722.3561	1488.7264	744.8668	1471.6998	736.3535	N	**318.1772**	159.5922	**301.1506**	151.0790	3
15	1517.7529	759.3801	1500.7264	750.8668	1545.7478	773.3776	1528.7213	764.8643	G	**204.1343**	102.5708	187.1077	94.0575	2
16									K	**147.1128**	74.0600	130.0863	65.5468	1

27b

MS/MS Fragmentation of TVEVVLTDTSEKK
Found in gi|18405982, avirulence-responsive protein, putative / avirulence induced gene (AIG) protein, putative [Arabidopsis thaliana]

Match to Query 60: 1527.779168 from(764.896860,2+) intensity(1343.0000)
Title: 33: Sum of 3 scans in range 1352 (rt=28.0506, f=3, i=28) to 1358 (rt=28.2967, f=3, i=30)

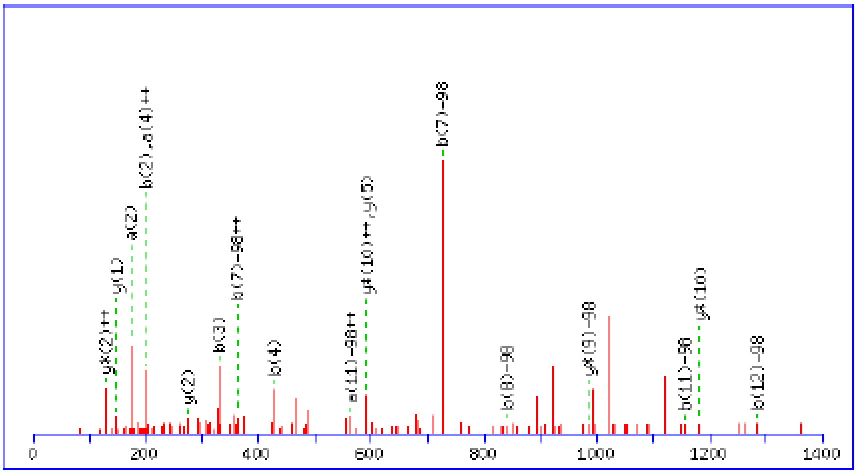

Monoisotopic mass of neutral peptide Mr(calc): 1527.7433
Fixed modifications: Carbamidomethyl (C)
Variable modifications:

T7 : Phospho (ST), with neutral losses 97.9769(shown in table), 0.0000
Ions Score: 23 **Expect:** 14
Matches (Bold Red): 18/152 fragment ions using 48 most intense peaks

#	a	a++	a*	a*++	b	b++	b*	b*++	Seq.	y	y++	y*	y*++	#
1	74.0600	37.5337			102.0550	51.5311			T					13
2	173.1285	87.0679			201.1234	101.0653			V	1329.7260	665.3666	1312.6995	656.8534	12
3	302.1710	151.5892			330.1660	165.5866			E	1230.6576	615.8324	1213.6311	607.3192	11
4	401.2395	201.1234			429.2344	215.1208			V	1101.6150	551.3111	1084.5885	542.7979	10
5	500.3079	250.6576			528.3028	264.6550			V	1002.5466	501.7769	985.5200	493.2637	9
6	613.3919	307.1996			641.3869	321.1971			L	903.4782	452.2427	886.4516	443.7295	8
7	696.4290	348.7182			724.4240	362.7156			T	790.3941	395.7007	773.3676	387.1874	7
8	811.4560	406.2316			839.4509	420.2291			D	707.3570	354.1821	690.3305	345.6689	6
9	912.5037	456.7555			940.4986	470.7529			T	592.3301	296.6687	575.3035	288.1554	5
10	999.5357	500.2715			1027.5306	514.2689			S	491.2824	246.1448	474.2558	237.6316	4
11	1128.5783	564.7928			1156.5732	578.7902			E	404.2504	202.6288	387.2238	194.1155	3
12	1256.6733	628.8403	1239.6467	620.3270	1284.6682	642.8377	1267.6416	634.3244	K	275.2078	138.1075	258.1812	129.5942	2
13									K	147.1128	74.0600	130.0863	65.5468	1

MS/MS Fragmentation of **EIDDETKTLTLR**
Found in **gi|15236568**, major latex protein-related / MLP-related [Arabidopsis thaliana]

Match to Query 101: 1512.670208 from(757.342380,2+) intensity(9464.0000)
Title: 62: Sum of 4 scans in range 1300 (rt=28.6692, f=3, i=55) to 1307 (rt=28.9492, f=3, i=58)

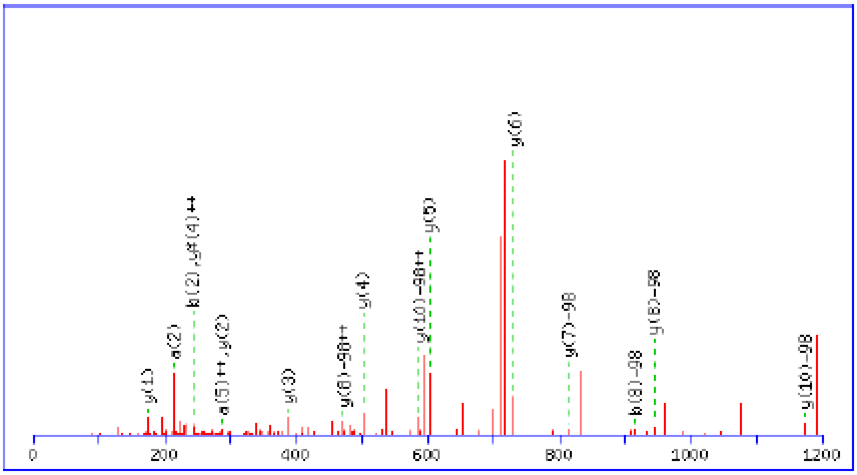

Monoisotopic mass of neutral peptide Mr(calc): 1512.7072
Fixed modifications: Carbamidomethyl (C)
Variable modifications:

T6 : Phospho (ST), with neutral losses 97.9769(shown in table), 0.0000
Ions Score: 45 **Expect:** 0.085
Matches (Bold Red): 16/172 fragment ions using 36 most intense peaks

#	a	a^{++}	a^{*}	a^{*++}	b	b^{++}	b^{*}	b^{*++}	Seq.	y	y^{++}	y^{*}	y^{*++}	#
1	102.0550	51.5311			130.0499	65.5286			E					12
2	**215.1390**	108.0731			**243.1339**	122.0706			I	1286.6951	643.8512	1269.6685	635.3379	11
3	330.1660	165.5866			358.1609	179.5841			D	**1173.6110**	**587.3091**	1156.5844	578.7959	10
4	445.1929	223.1001			473.1878	237.0975			D	1058.5840	529.7957	1041.5575	521.2824	9
5	574.2355	**287.6214**			602.2304	301.6188			E	**943.5571**	**472.2822**	926.5306	463.7689	8
6	657.2726	329.1399			685.2675	343.1374			T	**814.5145**	407.7609	797.4880	399.2476	7
7	785.3676	393.1874	768.3410	384.6741	813.3625	407.1849	796.3359	398.6716	K	**731.4774**	366.2423	714.4509	357.7291	6
8	886.4152	443.7113	869.3887	435.1980	**914.4102**	457.7087	897.3836	449.1954	T	**603.3824**	302.1949	586.3559	293.6816	5
9	999.4993	500.2533	982.4728	491.7400	1027.4942	514.2508	1010.4677	505.7375	L	**502.3348**	251.6710	485.3082	**243.1577**	4
10	1100.5470	550.7771	1083.5204	542.2639	1128.5419	564.7746	1111.5154	556.2613	T	**389.2507**	195.1290	372.2241	186.6157	3
11	1213.6311	607.3192	1196.6045	598.8059	1241.6260	621.3166	1224.5994	612.8033	L	**288.2030**	144.6051	271.1765	136.0919	2
12									R	**175.1190**	88.0631	158.0924	79.5498	1

MS/MS Fragmentation of SLVADMGNHVSK
Found in gi|15236568, major latex protein-related / MLP-related [Arabidopsis thaliana]

Match to Query 67: 1336.547248 from(669.280900,2+) intensity(5229.0000)
Title: 69: Sum of 3 scans in range 1329 (rt=29.7212, f=3, i=65) to 1335 (rt=29.9674, f=3, i=67)

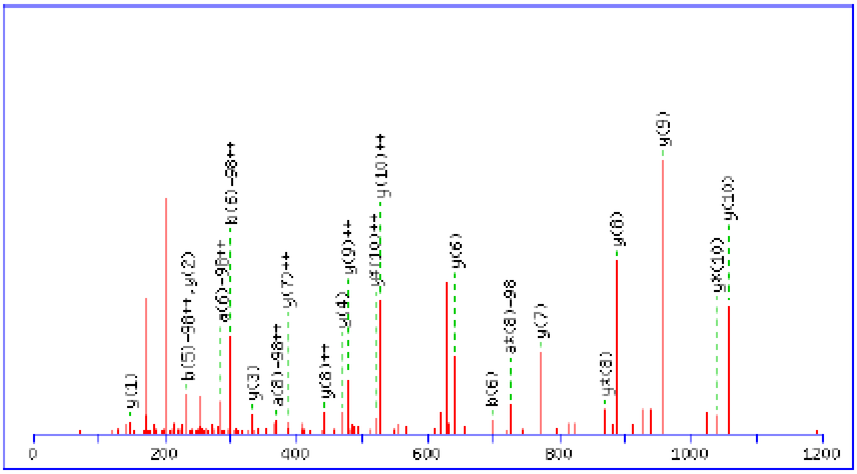

Monoisotopic mass of neutral peptide Mr(calc): 1336.5846
Fixed modifications: Carbamidomethyl (C)
Variable modifications:

S1 : Phospho (ST), with neutral losses 97.9769(shown in table), 0.0000
Ions Score: 63 **Expect:** 0.0013
Matches (Bold Red): 22/164 fragment ions using 33 most intense peaks

#	a	a^{++}	a*	a^{*++}	b	b^{++}	b*	b^{*++}	Seq.	y	y^{++}	y*	y^{*++}	#
1	42.0338	21.5205			70.0287	35.5180			S					12
2	155.1179	78.0626			183.1128	92.0600			L	1170.5936	585.8004	1153.5670	577.2871	11
3	254.1863	127.5968			282.1812	141.5942			V	**1057.5095**	**529.2584**	**1040.4830**	**520.7451**	10
4	325.2234	163.1153			353.2183	177.1128			A	**958.4411**	**479.7242**	941.4145	471.2109	9
5	440.2504	220.6288			468.2453	**234.6263**			D	**887.4040**	**444.2056**	**870.3774**	435.6924	8
6	571.2908	**286.1491**			599.2858	**300.1465**			M	**772.3770**	**386.6922**	755.3505	378.1789	7
7	628.3123	314.6598			656.3072	328.6572			G	**641.3365**	321.1719	624.3100	312.6586	6
8	742.3552	**371.6813**	**725.3287**	363.1680	770.3501	385.6787	753.3236	377.1654	N	584.3151	292.6612	567.2885	284.1479	5
9	879.4141	440.2107	862.3876	431.6974	907.4091	454.2082	890.3825	445.6949	H	**470.2722**	235.6397	453.2456	227.1264	4
10	978.4826	489.7449	961.4560	481.2316	1006.4775	503.7424	989.4509	495.2291	V	**333.2132**	167.1103	316.1867	158.5970	3
11	1065.5146	533.2609	1048.4880	524.7477	1093.5095	547.2584	1076.4829	538.7451	S	**234.1448**	117.5761	217.1183	109.0628	2
12									K	**147.1128**	74.0600	130.0863	65.5468	1

MS/MS Fragmentation of FVTSLAADMDDHILK
Found in gi|15223957, major latex protein-related / MLP-related [Arabidopsis thaliana]

Match to Query 125: 1754.732728 from(878.373640,2+) intensity(10785.0000)
Title: 128: Sum of 7 scans in range 1511 (rt=37.6599, f=3, i=126) to 1518 (rt=37.9398, f=4, i=145)

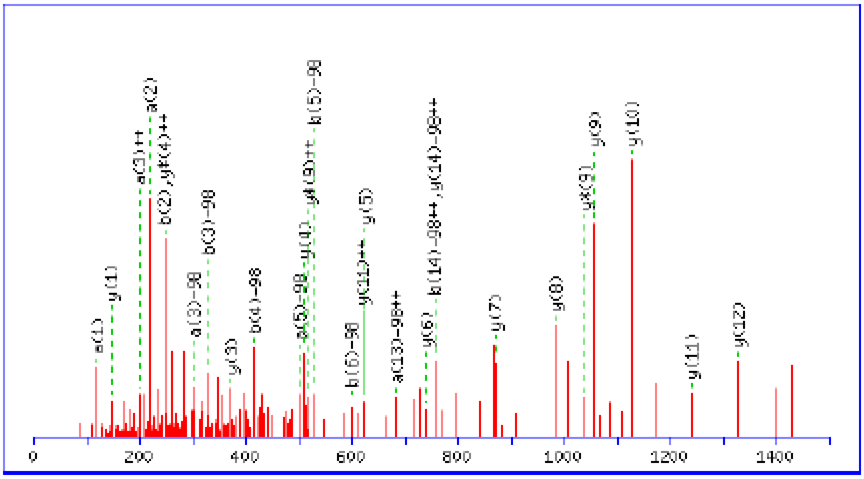


```
Monoisotopic mass of neutral peptide Mr(calc): 1754.7950
Fixed modifications: Carbamidomethyl (C)
Variable modifications:

T3      : Phospho (ST), with neutral losses 97.9769(shown in table), 0.0000
Ions Score: 69  Expect: 0.00029
Matches (Bold Red): 28/168 fragment ions using 47 most intense peaks
```

#	a	a++	b	b++	Seq.	y	y++	y*	y*++	#
1	120.0808	60.5440	148.0757	74.5415	F					15
2	219.1492	110.0782	247.1441	124.0757	V	1510.7570	755.8821	1493.7305	747.3689	14
3	302.1863	151.5968	330.1812	165.5942	T	1411.6886	706.3479	1394.6620	697.8347	13
4	389.2183	195.1128	417.2132	209.1103	S	1328.6515	664.8294	1311.6249	656.3161	12
5	502.3024	251.6548	530.2973	265.6523	L	1241.6194	621.3134	1224.5929	612.8001	11
6	573.3395	287.1734	601.3344	301.1708	A	1128.5354	564.7713	1111.5088	556.2581	10
7	644.3766	322.6919	672.3715	336.6894	A	1057.4983	529.2528	1040.4717	520.7395	9
8	759.4036	380.2054	787.3985	394.2029	D	986.4612	493.7342	969.4346	485.2209	8
9	890.4440	445.7257	918.4390	459.7231	M	871.4342	436.2207	854.4077	427.7075	7
10	1005.4710	503.2391	1033.4659	517.2366	D	740.3937	370.7005	723.3672	362.1872	6
11	1120.4979	560.7526	1148.4928	574.7501	D	625.3668	313.1870	608.3402	304.6738	5
12	1257.5568	629.2821	1285.5518	643.2795	H	510.3398	255.6736	493.3133	247.1603	4
13	1370.6409	685.8241	1398.6358	699.8215	I	373.2809	187.1441	356.2544	178.6308	3
14	1483.7250	742.3661	1511.7199	756.3636	L	260.1969	130.6021	243.1703	122.0888	2
15					K	147.1128	74.0600	130.0863	65.5468	1

MS/MS Fragmentation of RNDDFPEPSGYMK
Found in gi|15236566, major latex protein-related / MLP-related [Arabidopsis thaliana]

Match to Query 93: 1634.679588 from(818.347070,2+) intensity(5783.0000)
Title: 58: Sum of 6 scans in range 1564 (rt=33.3919, f=2, i=94) to 1571 (rt=33.6804, f=3, i=68)

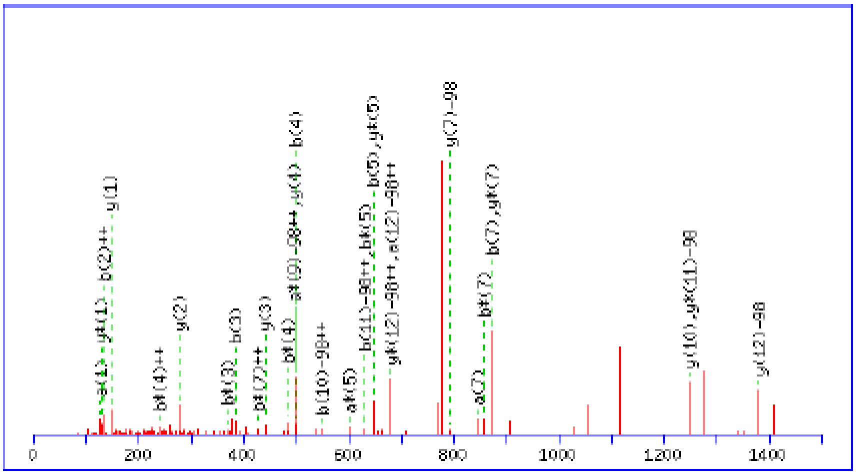

Monoisotopic mass of neutral peptide Mr(calc): 1634.6436
Fixed modifications: Carbamidomethyl (C)
Variable modifications:

S9 : Phospho (ST), with neutral losses 97.9769(shown in table), 0.0000
Ions Score: 34 **Expect:** 0.99
Matches (Bold Red): 30/208 fragment ions using 41 most intense peaks

#	a	a++	a*	a*++	b	b++	b*	b*++	Seq.	y	y++	y*	y*++	#
1	**129.1135**	65.0604	112.0869	56.5471	157.1084	79.0578	140.0818	70.5446	R					13
2	243.1564	122.0818	226.1299	113.5686	271.1513	**136.0793**	254.1248	127.5660	N	**1381.5729**	691.2901	1364.5463	**682.7768**	12
3	358.1833	179.5953	341.1568	171.0820	**386.1783**	193.5928	**369.1517**	185.0795	D	1267.5300	634.2686	**1250.5034**	625.7553	11
4	473.2103	237.1088	456.1837	228.5955	**501.2052**	251.1062	**484.1787**	**242.5930**	D	1152.5030	576.7551	1135.4765	568.2419	10
5	620.2787	310.6430	**603.2522**	302.1297	**648.2736**	324.6404	**631.2471**	316.1272	F	1037.4761	519.2417	1020.4495	510.7284	9
6	717.3315	359.1694	700.3049	350.6561	745.3264	373.1668	728.2998	364.6536	P	890.4077	445.7075	873.3811	437.1942	8
7	**846.3741**	423.6907	829.3475	415.1774	**874.3690**	437.6881	**857.3424**	**429.1748**	E	**793.3549**	397.1811	776.3283	388.6678	7
8	943.4268	472.2170	926.4003	463.7038	971.4217	486.2145	954.3952	477.7012	P	664.3123	332.6598	647.2858	324.1465	6
9	1012.4483	506.7278	995.4217	**498.2145**	1040.4432	520.7252	1023.4166	512.2120	S	567.2595	284.1334	550.2330	275.6201	5
10	1069.4697	535.2385	1052.4432	526.7252	1097.4647	**549.2360**	1080.4381	540.7227	G	**498.2381**	249.6227	481.2115	241.1094	4
11	1232.5331	616.7702	1215.5065	608.2569	1260.5280	**630.7676**	1243.5014	622.2544	Y	**441.2166**	221.1119	424.1901	212.5987	3
12	1363.5736	**682.2904**	1346.5470	673.7771	1391.5685	696.2879	1374.5419	687.7746	M	**278.1533**	139.5803	261.1267	131.0670	2
13									K	**147.1128**	74.0600	**130.0863**	65.5468	1

MS/MS Fragmentation of AAVTPAFAPAYAGINGLGVSLAR
Found in gi|1755154, germin-like protein [Arabidopsis thaliana]

Match to Query 102: 2266.205248 from(1134.109900,2+) intensity(2794.0000)
Title: 98: Sum of 4 scans in range 1850 (rt=41.9819, f=2, i=209) to 1853 (rt=42.0911, f=2, i=212)

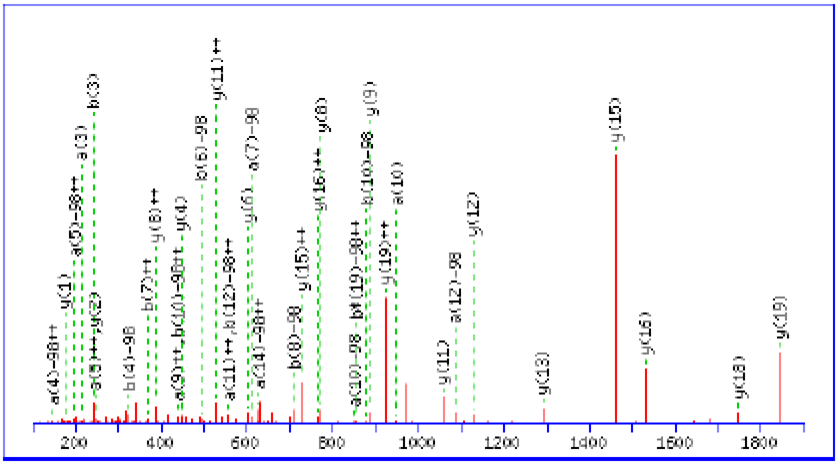

Monoisotopic mass of neutral peptide Mr(calc): 2266.1511
Fixed modifications: Carbamidomethyl (C)
Variable modifications:
T4 : Phospho (ST), with neutral losses 97.9769(shown in table), 0.0000

Ions Score: 68 **Expect:** 0.00047
Matches (**Bold Red**): 38/328 fragment ions using 69 most intense peaks

#	a	a++	a*	a*++	b	b++	b*	b*++	Seq.	y	y++	y*	y*++	#
1	44.0495	22.5284			72.0444	36.5258			A					23
2	115.0866	58.0469			143.0815	72.0444			A	2098.1444	1049.5758	2081.1178	1041.0626	22
3	**214.1550**	107.5811			**242.1499**	121.5786			V	2027.1073	1014.0573	2010.0807	1005.5440	21
4	297.1921	**149.0997**			**325.1870**	163.0972			T	1928.0389	964.5231	1911.0123	956.0098	20
5	394.2449	**197.6261**			422.2398	211.6235			P	**1845.0017**	**923.0045**	1827.9752	914.4912	19
6	465.2820	233.1446			**493.2769**	247.1421			A	**1747.9490**	874.4781	1730.9224	865.9649	18
7	**612.3504**	306.6788			640.3453	320.6763			F	1676.9119	838.9596	1659.8853	830.4463	17
8	683.3875	342.1974			**711.3824**	356.1949			A	**1529.8435**	**765.4254**	1512.8169	756.9121	16
9	780.4403	390.7238			808.4352	404.7212			P	**1458.8063**	**729.9068**	1441.7798	721.3935	15
10	**851.4774**	426.2423			**879.4723**	**440.2398**			A	1361.7536	681.3804	1344.7270	672.8672	14
11	1014.5407	507.7740			1042.5356	521.7715			Y	**1290.7165**	645.8619	1273.6899	637.3486	13
12	**1085.5778**	543.2926			1113.5728	**557.2900**			A	**1127.6531**	564.3302	1110.6266	555.8169	12
13	1142.5993	571.8033			1170.5942	585.8007			G	**1056.6160**	**528.8116**	1039.5895	520.2984	11
14	1255.6834	**628.3453**			1283.6783	642.3428			I	999.5946	500.3009	982.5680	491.7876	10
15	1369.7263	685.3668	1352.6997	676.8535	1397.7212	699.3642	1380.6947	690.8510	N	**886.5105**	443.7589	869.4839	435.2456	9
16	1426.7478	713.8775	1409.7212	705.3642	1454.7427	727.8750	1437.7161	719.3617	G	**772.4676**	**386.7374**	755.4410	378.2241	8
17	1539.8318	770.4195	1522.8053	761.9063	1567.8267	784.4170	1550.8002	775.9037	L	715.4461	358.2267	698.4196	349.7134	7
18	1596.8533	798.9303	1579.8267	790.4170	1624.8482	812.9277	1607.8216	804.4145	G	**602.3620**	301.6847	585.3355	293.1714	6
19	1695.9217	848.4645	1678.8951	839.9512	1723.9166	862.4619	1706.8901	**853.9487**	V	545.3406	273.1739	528.3140	264.6606	5
20	1782.9537	891.9805	1765.9272	883.4672	1810.9486	905.9780	1793.9221	897.4647	S	**446.2722**	223.6397	429.2456	215.1264	4
21	1896.0378	948.5225	1879.0112	940.0093	1924.0327	962.5200	1907.0062	954.0067	L	359.2401	180.1237	342.2136	171.6104	3
22	1967.0749	984.0411	1950.0484	975.5278	1995.0698	998.0385	1978.0433	989.5253	A	**246.1561**	123.5817	229.1295	115.0684	2
23									R	**175.1190**	88.0631	158.0924	79.5498	1

MS/MS Fragmentation of **ASELLHQFGVHVMPLTN**
Found in **gi|15228216**, jacalin lectin family protein [Arabidopsis thaliana]

Match to Query 98: 1988.039948 from(995.027250,2+) intensity(5595.0000)
Title: 99: Sum of 6 scans in range 1525 (rt=36.4958, f=2, i=156) to 1532 (rt=36.7829, f=3, i=109)

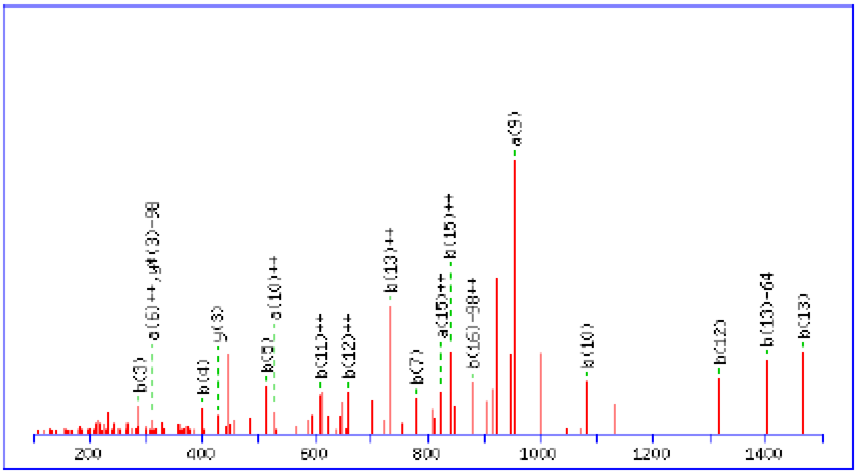

Monoisotopic mass of neutral peptide Mr(calc): 1987.9227
Fixed modifications: Carbamidomethyl (C)
Variable modifications:

M13 : Oxidation (M), with neutral losses 0.0000(shown in table), 63.9983
T16 : Phospho (ST), with neutral losses 97.9769(shown in table), 0.0000
Ions Score: 39 **Expect:** 0.43
Matches (Bold Red): 19/372 fragment ions using 31 most intense peaks

#	a	a++	a*	a*++	b	b++	b*	b*++	Seq.	y	y++	y*	y*++	#
1	44.0495	22.5284			72.0444	36.5258			A					17
2	131.0815	66.0444			159.0764	80.0418			S	1819.9160	910.4616	1802.8894	901.9483	16
3	260.1241	130.5657			**288.1190**	144.5631			E	1732.8839	866.9456	1715.8574	858.4323	15
4	373.2082	187.1077			**401.2031**	201.1052			L	1603.8413	802.4243	1586.8148	793.9110	14
5	486.2922	243.6497			**514.2871**	257.6472			L	1490.7573	745.8823	1473.7307	737.3690	13
6	623.3511	**312.1792**			651.3461	326.1767			H	1377.6732	689.3402	1360.6467	680.8270	12
7	751.4097	376.2085	734.3832	367.6952	**779.4046**	390.2060	762.3781	381.6927	Q	1240.6143	620.8108	1223.5878	612.2975	11
8	898.4781	449.7427	881.4516	441.2294	926.4730	463.7402	909.4465	455.2269	F	1112.5557	556.7815	1095.5292	548.2682	10
9	**955.4996**	478.2534	938.4730	469.7402	983.4945	492.2509	966.4680	483.7376	G	965.4873	483.2473	948.4608	474.7340	9
10	1054.5680	**527.7876**	1037.5415	519.2744	**1082.5629**	541.7851	1065.5364	533.2718	V	908.4658	454.7366	891.4393	446.2233	8
11	1191.6269	596.3171	1174.6004	587.8038	1219.6218	**610.3146**	1202.5953	601.8013	H	809.3974	405.2024	792.3709	396.6891	7
12	1290.6953	645.8513	1273.6688	637.3380	**1318.6902**	**659.8488**	1301.6637	651.3355	V	672.3385	336.6729	655.3120	328.1596	6
13	1437.7307	719.3690	1420.7042	710.8557	**1465.7257**	**733.3665**	1448.6991	724.8532	M	573.2701	287.1387	556.2436	278.6254	5
14	1534.7835	767.8954	1517.7570	759.3821	1562.7784	781.8928	1545.7519	773.3796	P	426.2347	213.6210	409.2082	205.1077	4
15	1647.8676	**824.4374**	1630.8410	815.9241	1675.8625	**838.4349**	1658.8359	829.9216	L	329.1819	165.0946	**312.1554**	156.5813	3
16	1730.9047	865.9560	1713.8781	857.4427	1758.8996	**879.9534**	1741.8730	871.4402	T	216.0979	108.5526	199.0713	100.0393	2
17									N	133.0608	67.0340	116.0342	58.5207	1

MS/MS Fragmentation of TSPPYGLETQKK
Found in gi|15228216, jacalin lectin family protein [Arabidopsis thaliana]

Match to Query 81: 1427.589888 from(714.802220,2+) intensity(4490.0000)
Title: 39: Sum of 4 scans in range 1399 (rt=28.8066, f=4, i=37) to 1404 (rt=28.9949, f=4, i=40)

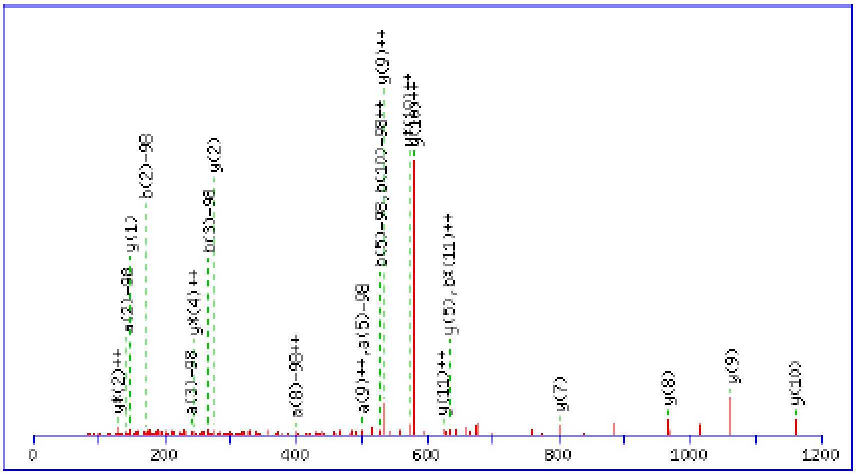

Monoisotopic mass of neutral peptide Mr(calc): 1427.6697
Fixed modifications: Carbamidomethyl (C)
Variable modifications:

T1 : Phospho (ST), with neutral losses 97.9769(shown in table), 0.0000
Ions Score: 26 **Expect:** 5.9
Matches (Bold Red): 23/148 fragment ions using 62 most intense peaks

#	a	a++	a*	a*++	b	b++	b*	b*++	Seq.	y	y++	y*	y*++	#
1	56.0495	28.5284			84.0444	42.5258			T					12
2	**143.0815**	72.0444			**171.0764**	86.0418			S	1247.6630	**624.3352**	1230.6365	615.8219	11
3	**240.1343**	120.5708			**268.1292**	134.5682			P	**1160.6310**	**580.8191**	1143.6045	**572.3059**	10
4	337.1870	169.0971			365.1819	183.0946			P	**1063.5782**	**532.2928**	1046.5517	523.7795	9
5	**500.2504**	250.6288			**528.2453**	264.6263			Y	**966.5255**	483.7664	949.4989	475.2531	8
6	557.2718	279.1395			585.2667	293.1370			G	**803.4621**	402.2347	786.4356	393.7214	7
7	670.3559	335.6816			698.3508	349.6790			L	746.4407	373.7240	729.4141	365.2107	6
8	799.3985	**400.2029**			827.3934	414.2003			E	**633.3566**	317.1819	616.3301	308.6687	5
9	900.4462	450.7267			928.4411	464.7242			T	504.3140	252.6607	487.2875	**244.1474**	4
10	1028.5047	514.7560	1011.4782	506.2427	1056.4996	**528.7535**	1039.4731	520.2402	Q	403.2663	202.1368	386.2398	193.6235	3
11	1156.5997	578.8035	1139.5731	570.2902	1184.5946	592.8009	1167.5681	584.2877	K	**275.2078**	138.1075	258.1812	**129.5942**	2
12									K	**147.1128**	74.0600	130.0863	65.5468	1

33b

MS/MS Fragmentation of KVHVGQGQDGVSSINVVYAK
Found in gi|15228216, jacalin lectin family protein [Arabidopsis thaliana]

Match to Query 128: 2163.934512 from(722.318780,3+) intensity(4085.0000)
Title: 65: Sum of 4 scans in range 1500 (rt=32.416, f=4, i=68) to 1505 (rt=32.6043, f=4, i=71)

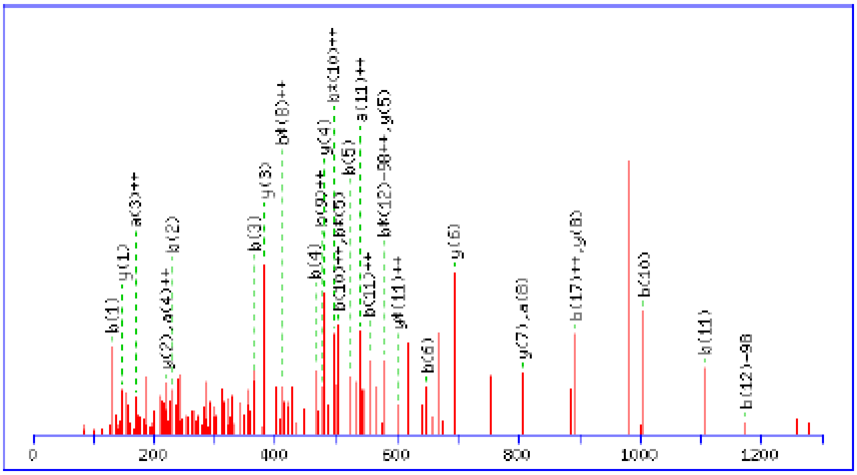

Monoisotopic mass of neutral peptide Mr(calc): 2164.0678
Fixed modifications: Carbamidomethyl (C)
Variable modifications:

S12 : Phospho (ST), with neutral losses 97.9769(shown in table), 0.0000
Ions Score: 61 **Expect:** 0.0019
Matches (**Bold Red**): 30/336 fragment ions using 48 most intense peaks

#	a	a^{++}	a^{*}	a^{*++}	b	b^{++}	b^{*}	b^{*++}	Seq.	y	y^{++}	y^{*}	y^{*++}	#
1	101.1073	51.0573	84.0808	42.5440	**129.1022**	65.0548	112.0757	56.5415	K					20
2	200.1757	100.5915	183.1492	92.0782	**228.1707**	114.5890	211.1441	106.0757	V	1939.0032	970.0052	1921.9766	961.4920	19
3	337.2347	**169.1210**	320.2081	160.6077	**365.2296**	183.1184	348.2030	174.6051	H	1839.9348	920.4710	1822.9082	911.9578	18
4	436.3031	**218.6552**	419.2765	210.1419	**464.2980**	232.6526	447.2714	224.1394	V	1702.8759	851.9416	1685.8493	843.4283	17
5	493.3245	247.1659	476.2980	238.6526	**521.3194**	261.1634	**504.2929**	252.6501	G	1603.8075	802.4074	1586.7809	793.8941	16
6	621.3831	311.1952	604.3566	302.6819	**649.3780**	325.1926	632.3515	316.6794	Q	1546.7860	773.8966	1529.7594	765.3834	15
7	678.4046	339.7059	661.3780	331.1926	706.3995	353.7034	689.3729	345.1901	G	1418.7274	709.8673	1401.7009	701.3541	14
8	**806.4631**	403.7352	789.4366	395.2219	834.4581	417.7327	817.4315	**409.2194**	Q	1361.7059	681.3566	1344.6794	672.8433	13
9	921.4901	461.2487	904.4635	452.7354	949.4850	**475.2461**	932.4585	466.7329	D	1233.6474	617.3273	1216.6208	608.8140	12
10	978.5116	489.7594	961.4850	481.2461	**1006.5065**	**503.7569**	989.4799	**495.2436**	G	1118.6204	559.8139	1101.5939	551.3006	11
11	1077.5800	**539.2936**	1060.5534	530.7803	**1105.5749**	**553.2911**	1088.5483	544.7778	V	1061.5990	531.3031	1044.5724	522.7898	10
12	1146.6014	573.8044	1129.5749	565.2911	**1174.5963**	587.8018	1157.5698	**579.2885**	S	962.5305	481.7689	945.5040	473.2556	9
13	1233.6335	617.3204	1216.6069	608.8071	1261.6284	631.3178	1244.6018	622.8045	S	**893.5091**	447.2582	876.4825	438.7449	8
14	1346.7175	673.8624	1329.6910	665.3491	1374.7124	687.8599	1357.6859	679.3466	I	**806.4771**	403.7422	789.4505	395.2289	7
15	1460.7604	730.8839	1443.7339	722.3706	1488.7554	744.8813	1471.7288	736.3680	N	**693.3930**	347.2001	676.3665	338.6869	6
16	1559.8289	780.4181	1542.8023	771.9048	1587.8238	794.4155	1570.7972	785.9023	V	**579.3501**	290.1787	562.3235	281.6654	5
17	1658.8973	829.9523	1641.8707	821.4390	1686.8922	843.9497	1669.8656	835.4365	V	**480.2817**	240.6445	463.2551	232.1312	4
18	1821.9606	911.4839	1804.9341	902.9707	1849.9555	925.4814	1832.9290	916.9681	Y	**381.2132**	191.1103	364.1867	182.5970	3
19	1892.9977	947.0025	1875.9712	938.4892	1920.9926	961.0000	1903.9661	952.4867	A	**218.1499**	109.5786	201.1234	101.0653	2
20									K	**147.1128**	74.0600	130.0863	65.5468	1

34a

MS/MS Fragmentation of NRPQFLVGSNSLLR
Found in gi|15226403, cupin family protein [Arabidopsis thaliana]

Match to Query 83: 1679.898228 from(840.956390,2+) intensity(14015.0000)
Title: 72: Sum of 4 scans in range 1629 (rt=37.2387, f=2, i=136) to 1633 (rt=37.4021, f=3, i=99)

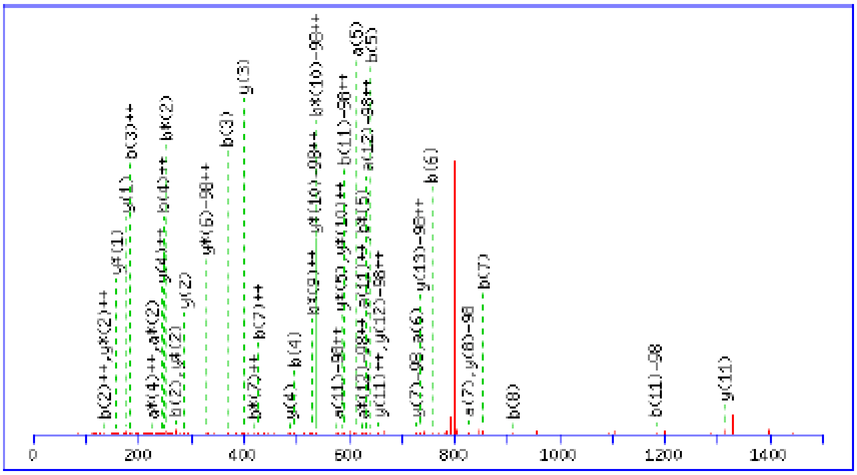

Monoisotopic mass of neutral peptide Mr(calc): 1679.8508
Fixed modifications: Carbamidomethyl (C)
Variable modifications:

S9 : Phospho (ST), with neutral losses 97.9769(shown in table), 0.0000
Ions Score: 25 **Expect:** 7.9
Matches (**Bold Red**): 45/228 fragment ions using 84 most intense peaks

#	a	a^{++}	a^*	a^{*++}	b	b^{++}	b^*	b^{*++}	Seq.	y	y^{++}	y^*	y^{*++}	#
1	87.0553	44.0313	70.0287	35.5180	115.0502	58.0287	98.0237	49.5155	N					14
2	243.1564	122.0818	**226.1299**	113.5686	**271.1513**	**136.0793**	**254.1248**	127.5660	R	1468.8383	**734.9228**	1451.8118	726.4095	13
3	340.2092	170.6082	323.1826	162.0949	**368.2041**	**184.6057**	351.1775	176.0924	P	1312.7372	**656.8722**	1295.7106	648.3590	12
4	468.2677	234.6375	451.2412	**226.1242**	**496.2627**	**248.6350**	479.2361	240.1217	Q	1215.6844	608.3459	1198.6579	599.8326	11
5	**615.3362**	308.1717	598.3096	299.6584	**643.3311**	322.1692	**626.3045**	313.6559	F	1087.6258	544.3166	1070.5993	**535.8033**	10
6	**728.4202**	364.7137	711.3937	356.2005	**756.4151**	378.7112	739.3886	370.1979	L	940.5574	470.7824	923.5309	462.2691	9
7	**827.4886**	414.2480	810.4621	405.7347	**855.4835**	**428.2454**	838.4570	**419.7321**	V	**827.4734**	414.2403	810.4468	405.7270	8
8	884.5101	442.7587	867.4835	434.2454	**912.5050**	456.7561	895.4785	448.2429	G	**728.4050**	364.7061	711.3784	356.1928	7
9	953.5316	477.2694	936.5050	468.7561	981.5265	491.2669	964.4999	482.7536	S	671.3835	336.1954	654.3569	**327.6821**	6
10	1067.5745	534.2909	1050.5479	525.7776	1095.5694	548.2883	1078.5428	**539.7751**	N	602.3620	301.6847	**585.3355**	293.1714	5
11	1154.6065	**577.8069**	1137.5800	569.2936	**1182.6014**	**591.8043**	1165.5749	583.2911	S	**488.3191**	**244.6632**	471.2926	236.1499	4
12	1267.6906	**634.3489**	1250.6640	**625.8356**	1295.6855	648.3464	1278.6589	639.8331	L	**401.2871**	201.1472	384.2605	192.6339	3
13	1380.7746	690.8910	1363.7481	682.3777	1408.7696	704.8884	1391.7430	696.3751	L	**288.2030**	144.6051	**271.1765**	**136.0919**	2
14									R	**175.1190**	88.0631	**158.0924**	79.5498	1

MS/MS Fragmentation of TGPFEFVGFTTSAHK
Found in gi|15226403, cupin family protein [Arabidopsis thaliana]

Match to Query 85: 1704.796848 from(853.405700,2+) intensity(3781.0000)
Title: 88: Sum of 3 scans in range 1712 (rt=39.9779, f=3, i=111) to 1718 (rt=40.224, f=3, i=113)

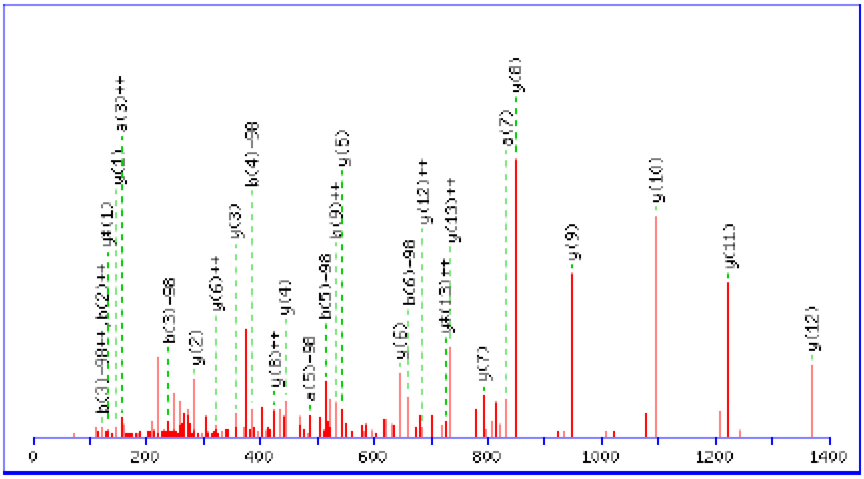

Monoisotopic mass of neutral peptide Mr(calc): 1704.7549
Fixed modifications: Carbamidomethyl (C)
Variable modifications:

T1 : Phospho (ST), with neutral losses 97.9769(shown in table), 0.0000
Ions Score: 59 **Expect:** 0.0032
Matches (Bold Red): 28/168 fragment ions using 68 most intense peaks

#	a	a++	b	b++	Seq.	y	y++	y*	y*++	#
1	56.0495	28.5284	84.0444	42.5258	T					15
2	113.0709	57.0391	141.0658	71.0366	G	1524.7482	762.8777	1507.7216	754.3644	14
3	210.1237	105.5655	**238.1186**	**119.5629**	P	1467.7267	**734.3670**	1450.7001	**725.8537**	13
4	357.1921	179.0997	**385.1870**	193.0972	F	**1370.6739**	**685.8406**	1353.6474	677.3273	12
5	**486.2347**	243.6210	**514.2296**	257.6184	E	**1223.6055**	612.3064	1206.5790	603.7931	11
6	633.3031	317.1552	**661.2980**	331.1527	F	**1094.5629**	547.7851	1077.5364	539.2718	10
7	732.3715	366.6894	760.3664	380.6869	V	**947.4945**	474.2509	930.4680	465.7376	9
8	789.3930	395.2001	817.3879	409.1976	G	**848.4261**	**424.7167**	831.3995	416.2034	8
9	936.4614	468.7343	964.4563	482.7318	F	**791.4046**	396.2060	774.3781	387.6927	7
10	1037.5091	519.2582	1065.5040	533.2556	T	**644.3362**	**322.6717**	627.3097	314.1585	6
11	1138.5568	569.7820	1166.5517	583.7795	T	**543.2885**	272.1479	526.2620	263.6346	5
12	1225.5888	613.2980	1253.5837	627.2955	S	**442.2409**	221.6241	425.2143	213.1108	4
13	1296.6259	648.8166	1324.6208	662.8141	A	**355.2088**	178.1081	338.1823	169.5948	3
14	1433.6848	717.3460	1461.6797	731.3435	H	**284.1717**	142.5895	267.1452	134.0762	2
15					K	**147.1128**	74.0600	**130.0863**	65.5468	1

34c

MS/MS Fragmentation of TGPFEFVGFTTSAHK
Found in gi|15226403, cupin family protein [Arabidopsis thaliana]

Match to Query 84: 1704.711888 from(853.363220,2+) intensity(7484.0000)
Title: 86: Sum of 4 scans in range 1660 (rt=37.9304, f=2, i=174) to 1663 (rt=38.0332, f=2, i=177)

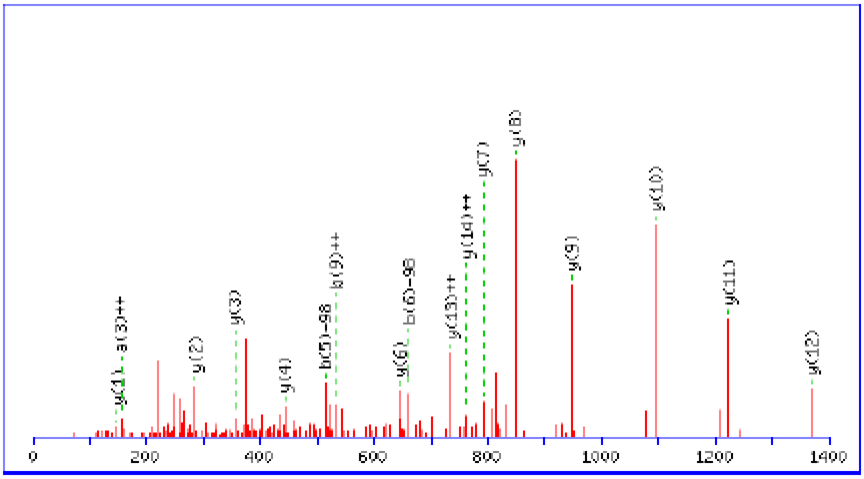

Monoisotopic mass of neutral peptide Mr(calc): 1704.7549
Fixed modifications: Carbamidomethyl (C)
Variable modifications:

T1 : Phospho (ST), with neutral losses 97.9769(shown in table), 0.0000
Ions Score: 70 Expect: 0.00024
Matches (Bold Red): 17/168 fragment ions using 34 most intense peaks

#	a	a++	b	b++	Seq.	y	y++	y*	y*++	#
1	56.0495	28.5284	84.0444	42.5258	T					15
2	113.0709	57.0391	141.0658	71.0366	G	1524.7482	**762.8777**	1507.7216	754.3644	14
3	210.1237	105.5655	238.1186	119.5629	P	1467.7267	**734.3670**	1450.7001	725.8537	13
4	357.1921	179.0997	385.1870	193.0972	F	**1370.6739**	685.8406	1353.6474	677.3273	12
5	486.2347	243.6210	**514.2296**	257.6184	E	**1223.6055**	612.3064	1206.5790	603.7931	11
6	633.3031	317.1552	**661.2980**	331.1527	F	**1094.5629**	547.7851	1077.5364	539.2718	10
7	732.3715	366.6894	760.3664	380.6869	V	**947.4945**	474.2509	930.4680	465.7376	9
8	789.3930	395.2001	817.3879	409.1976	G	**848.4261**	424.7167	831.3995	416.2034	8
9	936.4614	468.7343	964.4563	482.7318	F	**791.4046**	396.2060	774.3781	387.6927	7
10	1037.5091	519.2582	1065.5040	533.2556	T	**644.3362**	322.6717	627.3097	314.1585	6
11	1138.5568	569.7820	1166.5517	583.7795	T	543.2885	272.1479	526.2620	263.6346	5
12	1225.5888	613.2980	1253.5837	627.2955	S	**442.2409**	221.6241	425.2143	213.1108	4
13	1296.6259	648.8166	1324.6208	662.8141	A	**355.2088**	178.1081	338.1823	169.5948	3
14	1433.6848	717.3460	1461.6797	731.3435	H	**284.1717**	142.5895	267.1452	134.0762	2
15					K	**147.1128**	74.0600	130.0863	65.5468	1

MS/MS Fragmentation of GPAPLSLALAHADIDEAGK
Found in gi|9843639, Rieske FeS protein [Arabidopsis thaliana]

Match to Query 114: 1924.836248 from(963.425400,2+) intensity(4912.0000)
Title: 93: Sum of 3 scans in range 1606 (rt=37.6763, f=3, i=99) to 1612 (rt=37.9221, f=3, i=101)

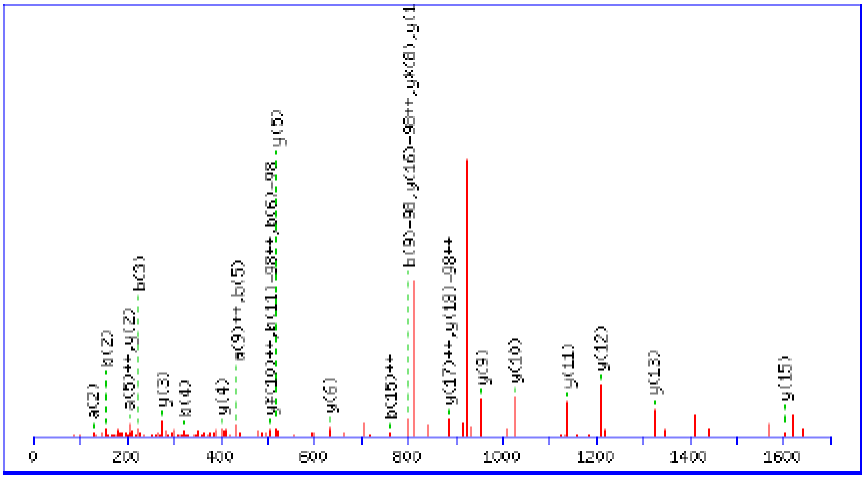


```
Monoisotopic mass of neutral peptide Mr(calc): 1924.9295
Fixed modifications: Carbamidomethyl (C)
Variable modifications:

S6      : Phospho (ST), with neutral losses 0.0000(shown in table), 97.9769
Ions Score: 56  Expect: 0.0072
Matches (Bold Red): 28/216 fragment ions using 42 most intense peaks
```

#	a	a++	b	b++	Seq.	y	y++	y*	y*++	#
1	30.0338	15.5206	58.0287	29.5180	G					19
2	**127.0866**	64.0469	**155.0815**	78.0444	P	1868.9154	934.9613	1851.8888	926.4480	18
3	198.1237	99.5655	**226.1186**	113.5629	A	1771.8626	**886.4349**	1754.8361	877.9217	17
4	295.1765	148.0919	**323.1714**	162.0893	P	1700.8255	850.9164	1683.7989	842.4031	16
5	408.2605	**204.6339**	**436.2554**	218.6314	L	**1603.7727**	**802.3900**	1586.7462	793.8767	15
6	575.2589	288.1331	603.2538	302.1305	S	1490.6887	745.8480	1473.6621	737.3347	14
7	688.3429	344.6751	716.3379	358.6726	L	**1323.6903**	662.3488	1306.6638	653.8355	13
8	759.3801	380.1937	787.3750	394.1911	A	**1210.6062**	605.8068	1193.5797	597.2935	12
9	872.4641	**436.7357**	900.4590	450.7332	L	**1139.5691**	570.2882	1122.5426	561.7749	11
10	943.5012	472.2543	971.4962	486.2517	A	**1026.4851**	513.7462	1009.4585	**505.2329**	10
11	1080.5602	540.7837	1108.5551	554.7812	H	**955.4480**	478.2276	938.4214	469.7143	9
12	1151.5973	576.3023	1179.5922	590.2997	A	818.3890	409.6982	**801.3625**	401.1849	8
13	1266.6242	633.8157	1294.6191	647.8132	D	747.3519	374.1796	730.3254	365.6663	7
14	1379.7083	690.3578	1407.7032	704.3552	I	**632.3250**	316.6661	615.2984	308.1529	6
15	1494.7352	747.8712	1522.7301	**761.8687**	D	**519.2409**	260.1241	502.2144	251.6108	5
16	1623.7778	812.3925	1651.7727	826.3900	E	**404.2140**	202.6106	387.1874	194.0974	4
17	1694.8149	847.9111	1722.8098	861.9086	A	**275.1714**	138.0893	258.1448	129.5761	3
18	1751.8364	876.4218	1779.8313	890.4193	G	**204.1343**	102.5708	187.1077	94.0575	2
19					K	147.1128	74.0600	130.0863	65.5468	1

MS/MS Fragmentation of GDPTYLVVENDK
Found in gi|9843639, Rieske FeS protein [Arabidopsis thaliana]

Match to Query 41: 1428.803168 from(715.408860,2+) intensity(5516.0000)
Title: 51: Sum of 3 scans in range 1418 (rt=30.8156, f=2, i=72) to 1422 (rt=30.9797, f=2, i=74)

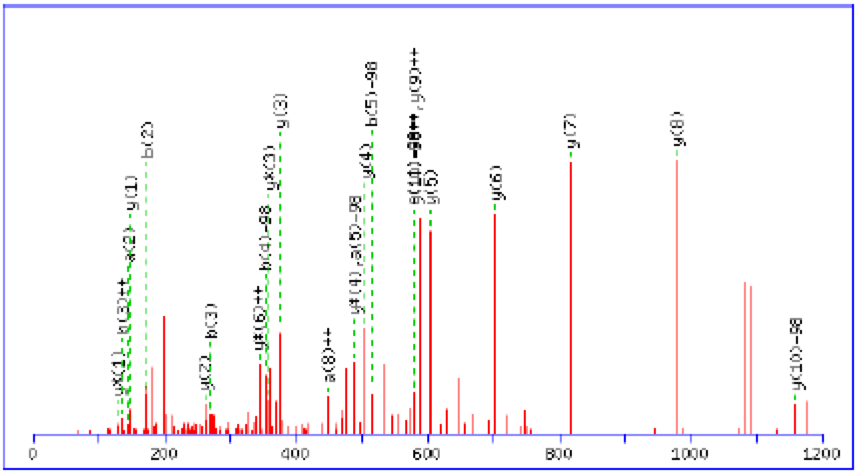


```
Monoisotopic mass of neutral peptide Mr(calc): 1428.6174
Fixed modifications: Carbamidomethyl (C)
Variable modifications:
T4      : Phospho (ST), with neutral losses 97.9769(shown in table), 0.0000
Ions Score: 40  Expect: 0.28
Matches (Bold Red): 24/148 fragment ions using 55 most intense peaks
```

#	a	a^{++}	a*	a^{*++}	b	b^{++}	b*	b^{*++}	Seq.	y	y^{++}	y*	y^{*++}	#
1	30.0338	15.5206			58.0287	29.5180			G					12
2	**145.0608**	73.0340			**173.0557**	87.0315			D	1274.6263	637.8168	1257.5998	629.3035	11
3	242.1135	121.5604			**270.1084**	**135.5579**			P	**1159.5994**	**580.3033**	1142.5728	571.7900	10
4	325.1506	163.0790			**353.1456**	177.0764			T	1062.5466	531.7769	1045.5200	523.2637	9
5	**488.2140**	244.6106			**516.2089**	258.6081			Y	**979.5095**	490.2584	962.4829	481.7451	8
6	601.2980	301.1527			629.2929	315.1501			L	**816.4462**	408.7267	799.4196	400.2134	7
7	700.3664	350.6869			728.3614	364.6843			V	**703.3621**	352.1847	686.3355	**343.6714**	6
8	799.4349	400.2211			827.4298	414.2185			V	**604.2937**	302.6505	587.2671	294.1372	5
9	928.4775	464.7424			956.4724	478.7398			E	**505.2253**	253.1163	**488.1987**	244.6030	4
10	1042.5204	521.7638	1025.4938	513.2506	1070.5153	535.7613	1053.4887	527.2480	N	**376.1827**	188.5950	**359.1561**	180.0817	3
11	1157.5473	**579.2773**	1140.5208	570.7640	1185.5422	593.2748	1168.5157	584.7615	D	**262.1397**	131.5735	245.1132	123.0602	2
12									K	**147.1128**	74.0600	**130.0863**	65.5468	1

MS/MS Fragmentation of FLCPCHGSQYNAQGR
Found in gi|9843639, Rieske FeS protein [Arabidopsis thaliana]

Match to Query 122: 1873.914608 from(937.964580,2+) intensity(17730.0000)
Title: 23: Sum of 6 scans in range 1280 (rt=26.0716, f=2, i=33) to 1287 (rt=26.358, f=3, i=28)

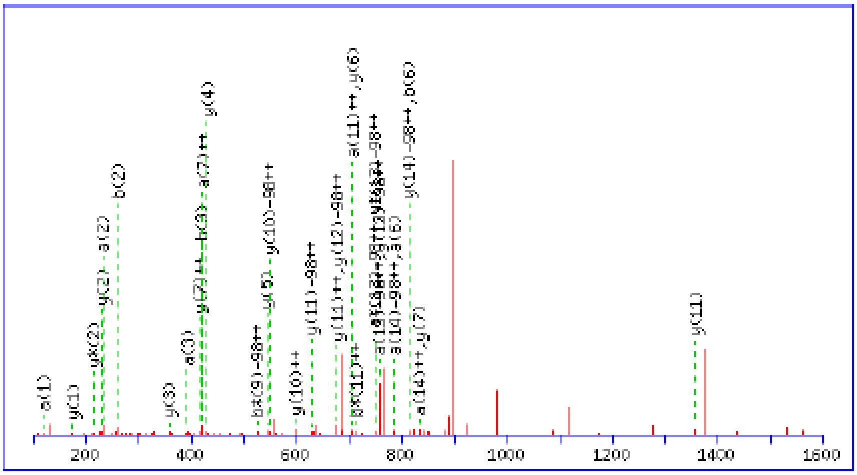

Monoisotopic mass of neutral peptide Mr(calc): 1873.7389
Fixed modifications: Carbamidomethyl (C)
Variable modifications:

S8 : Phospho (ST), with neutral losses 97.9769(shown in table), 0.0000
Ions Score: 28 **Expect:** 4.9
Matches (Bold Red): 33/216 fragment ions using 62 most intense peaks

#	a	a^{++}	a*	a^{*++}	b	b^{++}	b*	b^{*++}	Seq.	y	y^{++}	y*	y^{*++}	#
1	**120.0808**	60.5440			148.0757	74.5415			F					15
2	**233.1648**	117.0861			**261.1598**	131.0835			L	1629.7009	**815.3541**	1612.6744	806.8408	14
3	**393.1955**	197.1014			**421.1904**	211.0988			C	1516.6168	**758.8121**	1499.5903	**750.2988**	13
4	490.2483	245.6278			518.2432	259.6252			P	1356.5862	**678.7967**	1339.5596	670.2835	12
5	650.2789	325.6431			678.2738	339.6405			C	1259.5334	**630.2703**	1242.5069	621.7571	11
6	**787.3378**	394.1725			**815.3327**	408.1700			H	1099.5028	**550.2550**	1082.4762	541.7418	10
7	844.3593	**422.6833**			872.3542	436.6807			G	962.4439	481.7256	945.4173	473.2123	9
8	913.3807	457.1940			941.3756	471.1915			S	905.4224	453.2148	888.3958	444.7016	8
9	1041.4393	521.2233	1024.4128	512.7100	1069.4342	535.2208	1052.4077	**526.7075**	Q	**836.4009**	**418.7041**	819.3744	410.1908	7
10	1204.5026	602.7550	1187.4761	594.2417	1232.4976	616.7524	1215.4710	608.2391	Y	**708.3424**	354.6748	691.3158	346.1615	6
11	1318.5456	659.7764	1301.5190	651.2631	1346.5405	673.7739	1329.5139	665.2606	N	**545.2790**	273.1432	528.2525	264.6299	5
12	1389.5827	695.2950	1372.5561	686.7817	1417.5776	709.2924	1400.5510	700.7792	A	**431.2361**	216.1217	414.2096	207.6084	4
13	1517.6413	**759.3243**	1500.6147	**750.8110**	1545.6362	773.3217	1528.6096	764.8085	Q	**360.1990**	180.6031	343.1724	172.0899	3
14	1574.6627	**787.8350**	1557.6362	779.3217	1602.6576	801.8325	1585.6311	793.3192	G	**232.1404**	116.5738	**215.1139**	108.0606	2
15									R	**175.1190**	88.0631	158.0924	79.5498	1

MS/MS Fragmentation of **IVGEEHYETAQQVK**
Found in gi|7525040, ATP synthase CF1 beta subunit [Arabidopsis thaliana]

Match to Query 109: 1709.756828 from(855.885690,2+) intensity(2558.0000)
Title: 22: Sum of 4 scans in range 1172 (rt=24.1006, f=4, i=17) to 1179 (rt=24.3808, f=4, i=20)

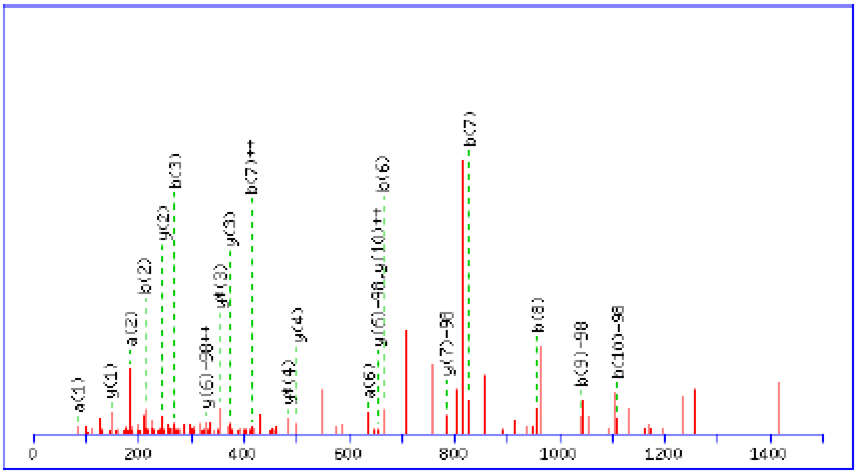

Monoisotopic mass of neutral peptide Mr(calc): 1709.7662
Fixed modifications: Carbamidomethyl (C)
Variable modifications:

T9 : Phospho (ST), with neutral losses 97.9769(shown in table), 0.0000
Ions Score: 24 **Expect:** 9.2
Matches (**Bold Red**): 21/180 fragment ions using 52 most intense peaks

#	a	a++	a*	a*++	b	b++	b*	b*++	Seq.	y	y++	y*	y*++	#
1	**86.0964**	43.5519			114.0913	57.5493			I					14
2	**185.1648**	93.0861			**213.1598**	107.0835			V	1499.7125	750.3599	1482.6859	741.8466	13
3	242.1863	121.5968			**270.1812**	135.5942			G	1400.6441	700.8257	1383.6175	692.3124	12
4	371.2289	186.1181			399.2238	200.1155			E	1343.6226	672.3149	1326.5961	663.8017	11
5	500.2715	250.6394			528.2664	264.6368			E	1214.5800	607.7936	1197.5535	599.2804	10
6	**637.3304**	319.1688			**665.3253**	333.1663			H	1085.5374	543.2724	1068.5109	534.7591	9
7	800.3937	400.7005			**828.3886**	**414.6980**			Y	948.4785	474.7429	931.4520	466.2296	8
8	929.4363	465.2218			**957.4312**	479.2193			E	**785.4152**	393.2112	768.3886	384.6980	7
9	1012.4734	506.7404			**1040.4683**	520.7378			T	**656.3726**	**328.6899**	639.3460	320.1767	6
10	1083.5105	542.2589			**1111.5055**	556.2564			A	573.3355	287.1714	556.3089	278.6581	5
11	1211.5691	606.2882	1194.5426	597.7749	1239.5640	620.2857	1222.5375	611.7724	Q	**502.2984**	251.6528	**485.2718**	243.1396	4
12	1339.6277	670.3175	1322.6012	661.8042	1367.6226	684.3149	1350.5961	675.8017	Q	**374.2398**	187.6235	**357.2132**	179.1103	3
13	1438.6961	719.8517	1421.6696	711.3384	1466.6910	733.8492	1449.6645	725.3359	V	**246.1812**	123.5942	229.1547	115.0810	2
14									K	**147.1128**	74.0600	130.0863	65.5468	1

MS/MS Fragmentation of SNDPHGLHCSGGVVLASR
Found in gi|1143394, V-type proton-ATPase [Arabidopsis thaliana]

Match to Query 102: 1941.971562 from(648.331130,3+) intensity(2452.0000)
Title: 29: Sum of 4 scans in range 1481 (rt=30.369, f=4, i=13) to 1488 (rt=30.6493, f=4, i=16)

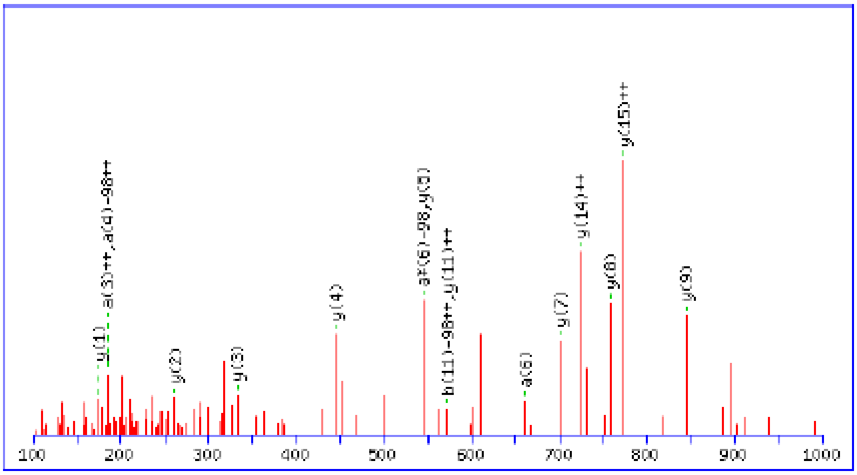

Monoisotopic mass of neutral peptide Mr(calc): 1941.8517
Fixed modifications: Carbamidomethyl (C)
Variable modifications:

S1 : Phospho (ST), with neutral losses 0.0000(shown in table), 97.9769
Ions Score: 37 **Expect:** 0.55
Matches (Bold Red): 16/332 fragment ions using 27 most intense peaks

#	a	a++	a*	a*++	b	b++	b*	b*++	Seq.	y	y++	y*	y*++	#
1	140.0107	70.5090			168.0056	84.5065			S					18
2	254.0536	127.5305	237.0271	119.0172	282.0486	141.5279	265.0220	133.0146	N	1775.8606	888.4339	1758.8340	879.9207	17
3	369.0806	**185.0439**	352.0540	176.5307	397.0755	199.0414	380.0489	190.5281	D	1661.8176	831.4125	1644.7911	822.8992	16
4	466.1333	233.5703	449.1068	225.0570	494.1283	247.5678	477.1017	239.0545	P	1546.7907	**773.8990**	1529.7642	765.3857	15
5	603.1923	302.0998	586.1657	293.5865	631.1872	316.0972	614.1606	307.5840	H	1449.7379	**725.3726**	1432.7114	716.8593	14
6	**660.2137**	330.6105	643.1872	322.0972	688.2086	344.6080	671.1821	336.0947	G	1312.6790	656.8432	1295.6525	648.3299	13
7	773.2978	387.1525	756.2712	378.6393	801.2927	401.1500	784.2662	392.6367	L	1255.6576	628.3324	1238.6310	619.8191	12
8	910.3567	455.6820	893.3302	447.1687	938.3516	469.6794	921.3251	461.1662	H	1142.5735	**571.7904**	1125.5470	563.2771	11
9	1070.3873	535.6973	1053.3608	527.1840	1098.3823	549.6948	1081.3557	541.1815	C	1005.5146	503.2609	988.4880	494.7477	10
10	1157.4194	579.2133	1140.3928	570.7001	1185.4143	593.2108	1168.3877	584.6975	S	**845.4839**	423.2456	828.4574	414.7323	9
11	1214.4408	607.7241	1197.4143	599.2108	1242.4358	621.7215	1225.4092	613.2082	G	**758.4519**	379.7296	741.4254	371.2163	8
12	1271.4623	636.2348	1254.4358	627.7215	1299.4572	650.2322	1282.4307	641.7190	G	**701.4305**	351.2189	684.4039	342.7056	7
13	1370.5307	685.7690	1353.5042	677.2557	1398.5256	699.7665	1381.4991	691.2532	V	644.4090	322.7081	627.3824	314.1949	6
14	1469.5991	735.3032	1452.5726	726.7899	1497.5940	749.3007	1480.5675	740.7874	V	**545.3406**	273.1739	528.3140	264.6606	5
15	1582.6832	791.8452	1565.6566	783.3320	1610.6781	805.8427	1593.6516	797.3294	L	**446.2722**	223.6397	429.2456	215.1264	4
16	1653.7203	827.3638	1636.6938	818.8505	1681.7152	841.3613	1664.6887	832.8480	A	**333.1881**	167.0977	316.1615	158.5844	3
17	1740.7523	870.8798	1723.7258	862.3665	1768.7473	884.8773	1751.7207	876.3640	S	**262.1510**	131.5791	245.1244	123.0659	2
18									R	**175.1190**	88.0631	158.0924	79.5498	1

MS/MS Fragmentation of INPTTSGSGVMTLEK
Found in gi|7708276, ATP synthase beta subunit [Dischidia lanceolata]

Match to Query 82: 1773.788728 from(887.901640,2+) intensity(1636.0000)
Title: 90: Sum of 4 scans in range 1792 (rt=41.059, f=2, i=172) to 1795 (rt=41.1615, f=2, i=175)

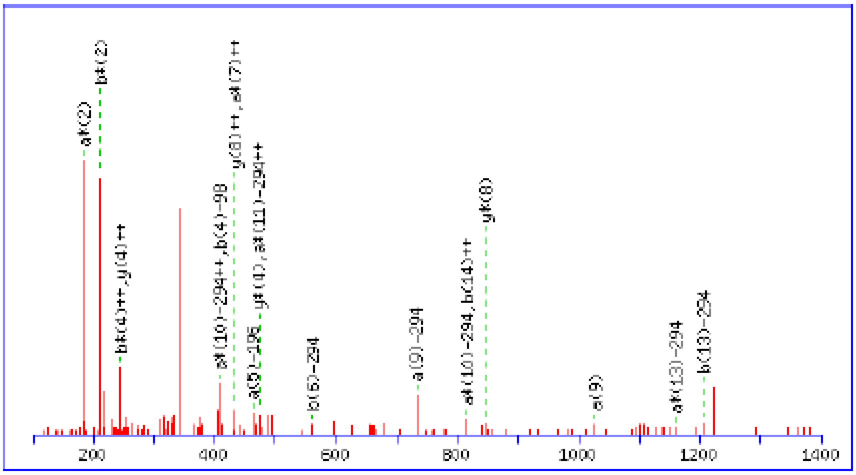

Monoisotopic mass of neutral peptide Mr(calc): 1773.6698

T4 : Phospho (ST), with neutral losses 97.9769(shown in table), 0.0000
T5 : Phospho (ST), with neutral losses 97.9769(shown in table), 0.0000
S6 : Phospho (ST), with neutral losses 97.9769(shown in table), 0.0000
Ions Score: 12 **Expect**: 1.7e+02
Matches (**Bold Red**): 19/272 fragment ions using 31 most intense peaks

#	a	a++	a*	a*++	b	b++	b*	b*++	Seq.	y	y++	y*	y*++	#
1	86.0964	43.5519			114.0913	57.5493			I					15
2	200.1394	100.5733	**183.1128**	92.0600	228.1343	114.5708	**211.1077**	106.0575	N	1367.6624	684.3348	1350.6358	675.8215	14
3	297.1921	149.0997	280.1656	140.5864	325.1870	163.0972	308.1605	154.5839	P	1253.6194	627.3134	1236.5929	618.8001	13
4	380.2292	190.6182	363.2027	182.1050	**408.2241**	204.6157	391.1976	196.1024	T	1156.5667	578.7870	1139.5401	570.2737	12
5	**463.2663**	232.1368	446.2398	223.6235	491.2612	246.1343	474.2347	237.6210	T	1073.5296	537.2684	1056.5030	528.7551	11
6	532.2878	266.6475	515.2612	258.1343	**560.2827**	280.6450	543.2562	272.1317	S	990.4925	495.7499	973.4659	487.2366	10
7	589.3093	295.1583	572.2827	286.6450	617.3042	309.1557	600.2776	300.6424	G	921.4710	461.2391	904.4444	452.7259	9
8	676.3413	338.6743	659.3147	330.1610	704.3362	352.6717	687.3096	344.1585	S	864.4495	**432.7284**	**847.4230**	424.2151	8
9	**733.3627**	367.1850	716.3362	358.6717	761.3577	381.1825	744.3311	372.6692	G	777.4175	389.2124	760.3910	380.6991	7
10	832.4312	416.7192	**815.4046**	**408.2059**	860.4261	430.7167	843.3995	422.2034	V	720.3960	360.7017	703.3695	352.1884	6
11	963.4716	482.2395	946.4451	**473.7262**	991.4666	496.2369	974.4400	487.7236	M	621.3276	311.1675	604.3011	302.6542	5
12	1064.5193	532.7633	1047.4928	524.2500	1092.5142	546.7608	1075.4877	538.2475	T	490.2871	**245.6472**	**473.2606**	237.1339	4
13	1177.6034	589.3053	**1160.5768**	580.7921	**1205.5983**	603.3028	1188.5718	594.7895	L	389.2395	195.1234	372.2129	186.6101	3
14	1306.6460	653.8266	1289.6194	645.3134	1334.6409	667.8241	1317.6143	659.3108	E	276.1554	138.5813	259.1288	130.0681	2
15									K	147.1128	74.0600	130.0863	65.5468	1

MS/MS Fragmentation of AENEGLSDGLSLIEEVKK
Found in gi|15236678, APX4 (ASCORBATE PEROXIDASE 4); peroxidase [Arabidopsis thaliana]

Match to Query 115: 2009.892248 from(1005.953400,2+) intensity(8023.0000)
Title: 107: Sum of 2 scans in range 1525 (rt=37.2078, f=2, i=183) to 1528 (rt=37.3305, f=2, i=184)

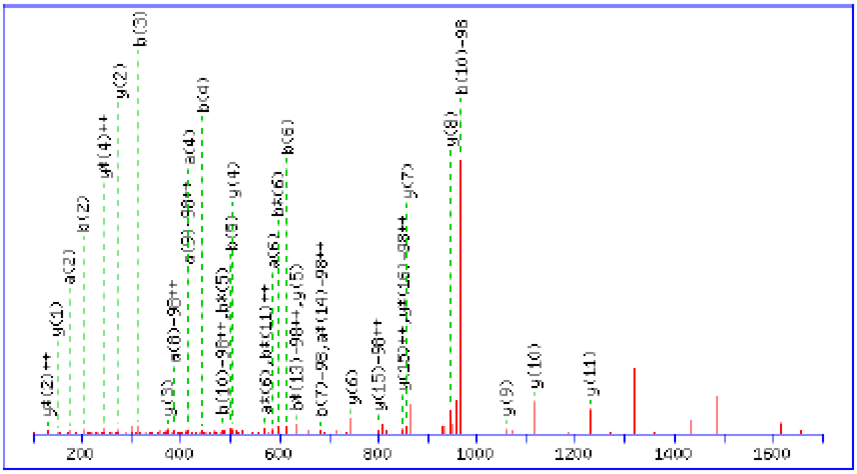

Monoisotopic mass of neutral peptide Mr(calc): 2009.9558
Fixed modifications: Carbamidomethyl (C)
Variable modifications:

S7 : Phospho (ST), with neutral losses 97.9769(shown in table), 0.0000
Ions Score: 74 **Expect:** 0.0001
Matches (Bold Red): 35/308 fragment ions using 49 most intense peaks

#	a	a++	a*	a*++	b	b++	b*	b*++	Seq.	y	y++	y*	y*++	#
1	44.0495	22.5284			72.0444	36.5258			A					18
2	**173.0921**	87.0497			**201.0870**	101.0471			E	1841.9491	921.4782	1824.9225	912.9649	17
3	287.1350	144.0711	270.1084	135.5579	**315.1299**	158.0686	298.1034	149.5553	N	1712.9065	856.9569	1695.8799	**848.4436**	16
4	**416.1776**	208.5924	399.1510	200.0792	**444.1725**	222.5899	427.1460	214.0766	E	1598.8636	**799.9354**	1581.8370	791.4221	15
5	473.1991	237.1032	456.1725	228.5899	**501.1940**	251.1006	**484.1674**	242.5873	G	1469.8210	735.4141	1452.7944	726.9009	14
6	**586.2831**	293.6452	**569.2566**	285.1319	**614.2780**	307.6427	**597.2515**	299.1294	L	1412.7995	706.9034	1395.7730	698.3901	13
7	655.3046	328.1559	638.2780	319.6426	**683.2995**	342.1534	666.2729	333.6401	S	1299.7155	650.3614	1282.6889	641.8481	12
8	770.3315	**385.6694**	753.3050	377.1561	798.3264	399.6669	781.2999	391.1536	D	**1230.6940**	615.8506	1213.6674	607.3374	11
9	827.3530	**414.1801**	810.3264	405.6669	855.3479	428.1776	838.3213	419.6643	G	**1115.6671**	558.3372	1098.6405	549.8239	10
10	940.4370	470.7222	923.4105	462.2089	**968.4320**	**484.7196**	951.4054	476.2063	L	**1058.6456**	529.8264	1041.6190	521.3132	9
11	1027.4691	514.2382	1010.4425	505.7249	1055.4640	528.2356	1038.4374	519.7224	S	**945.5615**	473.2844	928.5350	464.7711	8
12	1140.5531	570.7802	1123.5266	562.2669	1168.5481	584.7777	1151.5215	576.2644	L	**858.5295**	429.7684	841.5029	421.2551	7
13	1253.6372	627.3222	1236.6107	618.8090	1281.6321	641.3197	1264.6056	**632.8064**	I	**745.4454**	373.2264	728.4189	364.7131	6
14	1382.6798	691.8435	1365.6532	**683.3303**	1410.6747	705.8410	1393.6482	697.3277	E	**632.3614**	316.6843	615.3348	308.1710	5
15	1511.7224	756.3648	1494.6958	747.8516	1539.7173	770.3623	1522.6908	761.8490	E	**503.3188**	252.1630	486.2922	**243.6498**	4
16	1610.7908	805.8990	1593.7643	797.3858	1638.7857	819.8965	1621.7592	811.3832	V	**374.2762**	187.6417	357.2496	179.1285	3
17	1738.8858	869.9465	1721.8592	861.4332	1766.8807	883.9440	1749.8541	875.4307	K	**275.2078**	138.1075	258.1812	**129.5942**	2
18									K	**147.1128**	74.0600	130.0863	65.5468	1

MS/MS Fragmentation of TPAEFASVGSNIFGTFGTFLK
Found in gi|15223576, DHAR1 (DEHYDROASCORBATE REDUCTASE); glutathione dehydrogenase (ascorbate)

Match to Query 192: 2270.189048 from(1136.101800,2+) intensity(3553.0000)
Title: 200: Sum of 4 scans in range 1893 (rt=48.1008, f=2, i=230) to 1896 (rt=48.2034, f=2, i=233)

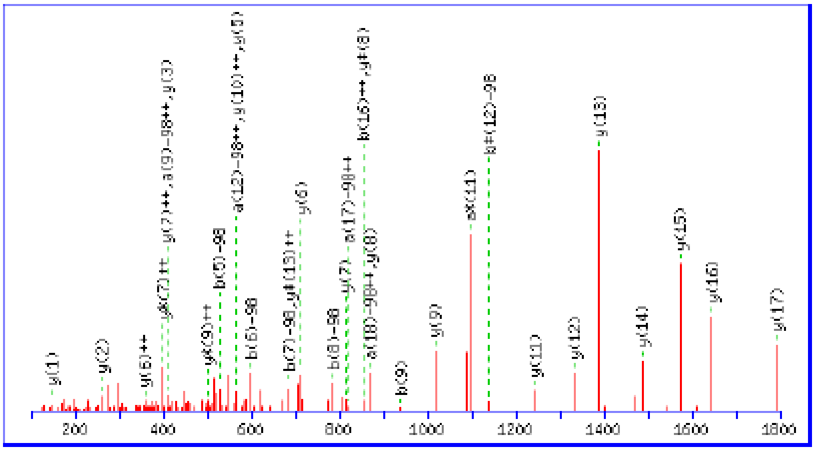


```
Monoisotopic mass of neutral peptide Mr(calc): 2270.0661
Fixed modifications: Carbamidomethyl (C)
Variable modifications:
T1      : Phospho (ST), with neutral losses 97.9769(shown in table), 0.0000
Ions Score: 96  Expect: 6.4e-07
Matches (Bold Red): 34/320 fragment ions using 53 most intense peaks
```

#	a	a++	a*	a*++	b	b++	b*	b*++	Seq.	y	y++	y*	y*++	#
1	56.0495	28.5284			84.0444	42.5258			T					21
2	153.1022	77.0548			181.0971	91.0522			P	2090.0593	1045.5333	2073.0328	1037.0200	20
3	224.1393	112.5733			252.1343	126.5708			A	1993.0066	997.0069	1975.9800	988.4936	19
4	353.1819	177.0946			381.1769	191.0921			E	1921.9694	961.4884	1904.9429	952.9751	18
5	500.2504	250.6288			528.2453	264.6263			F	1792.9268	896.9671	1775.9003	888.4538	17
6	571.2875	286.1474			599.2824	300.1448			A	1645.8584	823.4329	1628.8319	814.9196	16
7	658.3195	329.6634			686.3144	343.6608			S	1574.8213	787.9143	1557.7948	779.4010	15
8	757.3879	379.1976			785.3828	393.1950			V	1487.7893	744.3983	1470.7627	735.8850	14
9	814.4094	407.7083			842.4043	421.7058			G	1388.7209	694.8641	1371.6943	686.3508	13
10	901.4414	451.2243			929.4363	465.2218			S	1331.6994	666.3533	1314.6729	657.8401	12
11	1015.4843	508.2458	998.4578	499.7325	1043.4792	522.2433	1026.4527	513.7300	N	1244.6674	622.8373	1227.6408	614.3241	11
12	1128.5684	564.7878	1111.5418	556.2746	1156.5633	578.7853	1139.5368	570.2720	I	1130.6245	565.8159	1113.5979	557.3026	10
13	1275.6368	638.3220	1258.6103	629.8088	1303.6317	652.3195	1286.6052	643.8062	F	1017.5404	509.2738	1000.5138	500.7606	9
14	1332.6583	666.8328	1315.6317	658.3195	1360.6532	680.8302	1343.6266	672.3170	G	870.4720	435.7396	853.4454	427.2264	8
15	1433.7059	717.3566	1416.6794	708.8433	1461.7009	731.3541	1444.6743	722.8408	T	813.4505	407.2289	796.4240	398.7156	7
16	1580.7744	790.8908	1563.7478	782.3775	1608.7693	804.8883	1591.7427	796.3750	F	712.4028	356.7051	695.3763	348.1918	6
17	1637.7958	819.4016	1620.7693	810.8883	1665.7907	833.3990	1648.7642	824.8857	G	565.3344	283.1709	548.3079	274.6576	5
18	1738.8435	869.9254	1721.8170	861.4121	1766.8384	883.9228	1749.8119	875.4096	T	508.3130	254.6601	491.2864	246.1468	4
19	1885.9119	943.4596	1868.8854	934.9463	1913.9068	957.4571	1896.8803	948.9438	F	407.2653	204.1363	390.2387	195.6230	3
20	1998.9960	1000.0016	1981.9694	991.4884	2026.9909	1013.9991	2009.9644	1005.4858	L	260.1969	130.6021	243.1703	122.0888	2
21									K	147.1128	74.0600	130.0863	65.5468	1

MS/MS Fragmentation of VLTIYAPLFASSK
Found in gi|15224582, ATGSTF10 (EARLY DEHYDRATION-INDUCED 13); glutathione transferase

Match to Query 119: 1488.837268 from(745.425910,2+) intensity(13333.0000)
Title: 160: Sum of 2 scans in range 1627 (rt=41.1931, f=2, i=165) to 1630 (rt=41.3158, f=2, i=166)

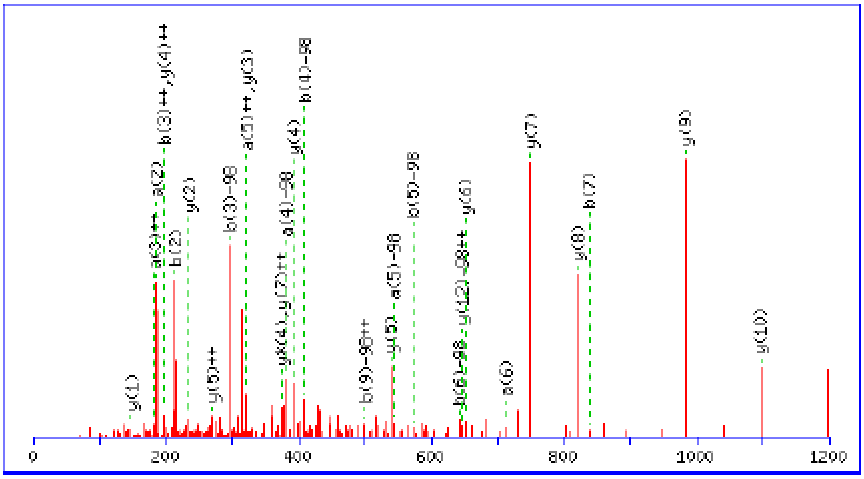

Monoisotopic mass of neutral peptide Mr(calc): 1488.7629
Fixed modifications: Carbamidomethyl (C)
Variable modifications:

T3 : Phospho (ST), with neutral losses 97.9769(shown in table), 0.0000
Ions Score: 46 **Expect:** 0.063
Matches (Bold Red): 29/144 fragment ions using 67 most intense peaks

#	a	a++	b	b++	Seq.	y	y++	y*	y*++	#
1	72.0808	36.5440	100.0757	50.5415	V					13
2	**185.1648**	93.0861	**213.1598**	107.0835	L	1292.7249	**646.8661**	1275.6983	638.3528	12
3	268.2019	134.6046	**296.1969**	148.6021	T	1179.6408	590.3241	1162.6143	581.8108	11
4	**381.2860**	191.1466	**409.2809**	205.1441	I	**1096.6037**	548.8055	1079.5772	540.2922	10
5	**544.3493**	272.6783	**572.3443**	286.6758	Y	**983.5197**	492.2635	966.4931	483.7502	9
6	615.3865	308.1969	**643.3814**	322.1943	A	**820.4563**	410.7318	803.4298	402.2185	8
7	712.4392	356.7232	740.4341	370.7207	P	**749.4192**	**375.2132**	732.3927	366.7000	7
8	825.5233	413.2653	853.5182	427.2627	L	**652.3665**	326.6869	635.3399	318.1736	6
9	972.5917	486.7995	1000.5866	**500.7969**	F	**539.2824**	**270.1448**	522.2558	261.6316	5
10	1043.6288	522.3180	1071.6237	536.3155	A	**392.2140**	**196.6106**	**375.1874**	188.0974	4
11	1130.6608	565.8341	1158.6558	579.8315	S	**321.1769**	161.0921	304.1503	152.5788	3
12	1217.6929	609.3501	1245.6878	623.3475	S	**234.1448**	117.5761	217.1183	109.0628	2
13					K	**147.1128**	74.0600	130.0863	65.5468	1

MS/MS Fragmentation of QPAYLALQPFGTVPAVVDGDYK
Found in gi|15224581, ATGSTF9 (Arabidopsis thaliana Glutathione S-transferase (class phi) 9); glutathione transferase

Match to Query 152: 2428.318848 from(1215.166700,2+) intensity(3995.0000)
Title: 137: Sum of 4 scans in range 1662 (rt=41.3837, f=3, i=142) to 1667 (rt=41.5819, f=3, i=145)
[illegible]

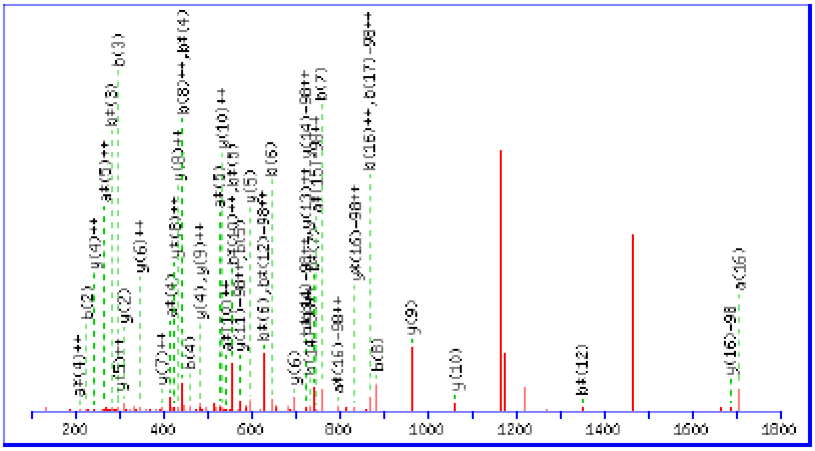

Monoisotopic mass of neutral peptide Mr(calc): 2428.1716
Fixed modifications: Carbamidomethyl (C)
Variable modifications:
T12 : Phospho (ST), with neutral losses 97.9769(shown in table), 0.0000
Ions Score: 46 **Expect:** 0.073
Matches (Bold Red): 48/376 fragment ions using 65 most intense peaks

#	a	a++	a*	a*++	b	b++	b*	b*++	Seq.	y	y++	y*	y*++	#
1	101.0709	51.0391	84.0444	42.5258	129.0659	65.0366	112.0393	56.5233	Q					22
2	198.1237	99.5655	181.0972	91.0522	**226.1186**	113.5629	209.0921	105.0497	P	2203.1434	1102.0753	2186.1168	1093.5621	21
3	269.1608	135.0840	252.1343	126.5708	**297.1557**	149.0815	**280.1292**	140.5682	A	2106.0906	1053.5489	2089.0641	1045.0357	20
4	432.2241	216.6157	**415.1976**	**208.1024**	**460.2191**	230.6132	**443.1925**	222.0999	Y	2035.0535	1018.0304	2018.0270	1009.5171	19
5	545.3082	273.1577	**528.2817**	**264.6445**	**573.3031**	287.1552	**556.2766**	278.6419	L	1871.9902	936.4987	1854.9636	927.9854	18
6	616.3453	308.6763	599.3188	300.1630	**644.3402**	322.6738	**627.3137**	314.1605	A	1758.9061	879.9567	1741.8796	871.4434	17
7	729.4294	365.2183	712.4028	356.7051	**757.4243**	379.2158	**740.3978**	370.7025	L	**1687.8690**	844.4381	1670.8424	**835.9249**	16
8	857.4880	429.2476	840.4614	420.7343	**885.4829**	**443.2451**	868.4563	434.7318	Q	1574.7849	787.8961	1557.7584	779.3828	15
9	954.5407	477.7740	937.5142	469.2607	982.5356	491.7715	965.5091	483.2582	P	1446.7264	**723.8668**	1429.6998	715.3535	14
10	1101.6091	551.3082	1084.5826	**542.7949**	1129.6041	565.3057	1112.5775	**556.7924**	F	1349.6736	675.3404	1332.6470	666.8272	13
11	1158.6306	579.8189	1141.6041	571.3057	1186.6255	593.8164	1169.5990	585.3031	G	1202.6052	601.8062	1185.5786	593.2930	12
12	1241.6677	621.3375	1224.6412	612.8242	1269.6626	635.3350	1252.6361	**626.8217**	T	1145.5837	**573.2955**	1128.5572	564.7822	11
13	1340.7361	670.8717	1323.7096	662.3584	1368.7310	684.8692	1351.7045	676.3559	V	**1062.5466**	**531.7769**	1045.5201	523.2637	10
14	1437.7889	719.3981	1420.7623	710.8848	1465.7838	**733.3955**	1448.7573	**724.8823**	P	**963.4782**	**482.2427**	946.4516	473.7295	9
15	1508.8260	754.9166	1491.7995	**746.4034**	1536.8209	768.9141	1519.7944	760.4008	A	866.4254	**433.7164**	849.3989	**425.2031**	8
16	1607.8944	804.4508	1590.8679	**795.9376**	1635.8893	818.4483	1618.8628	809.9350	V	795.3883	**398.1978**	778.3618	389.6845	7
17	1706.9628	853.9851	1689.9363	845.4718	1734.9578	**867.9825**	1717.9312	859.4692	V	**696.3199**	**348.6636**	679.2933	340.1503	6
18	1821.9898	911.4985	1804.9632	902.9853	1849.9847	925.4960	1832.9581	916.9827	D	**597.2515**	**299.1294**	580.2249	290.6161	5
19	1879.0112	940.0093	1861.9847	931.4960	1907.0062	954.0067	1889.9796	945.4934	G	**482.2245**	**241.6159**	465.1980	233.1026	4
20	1994.0382	997.5227	1977.0116	989.0095	2022.0331	1011.5202	2005.0066	1003.0069	D	**425.2031**	213.1052	408.1765	204.5919	3
21	2157.1015	1079.0544	2140.0750	1070.5411	2185.0964	1093.0519	2168.0699	1084.5386	Y	**310.1761**	155.5917	293.1496	147.0784	2
22									K	147.1128	74.0600	130.0863	65.5468	1

MS/MS Fragmentation of VFGHPASTATR
Found in gi|15218640, ATGSTF6 (EARLY RESPONSIVE TO DEHYDRATION 11); glutathione transferase

Match to Query 62: 1222.523988 from(612.269270,2+) intensity(8040.0000)
Title: 20: Sum of 3 scans in range 1144 (rt=23.3826, f=2, i=57) to 1150 (rt=23.6282, f=2, i=59)

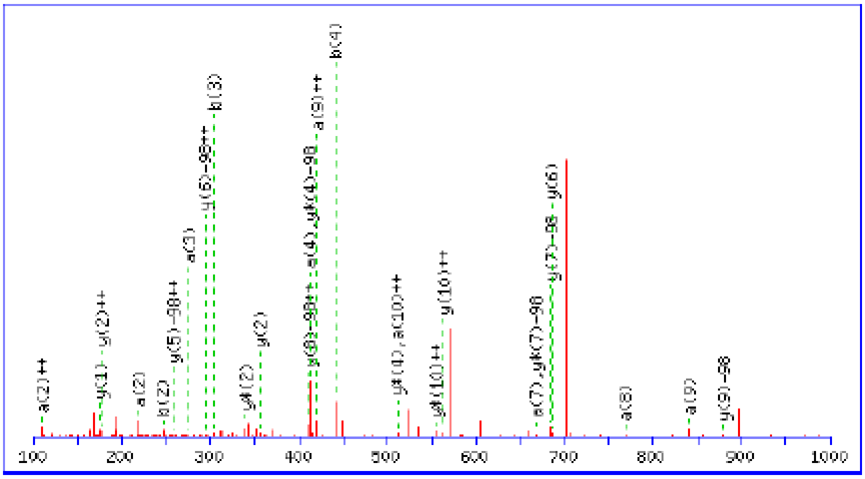


```
Monoisotopic mass of neutral peptide Mr(calc): 1222.5496
Fixed modifications: Carbamidomethyl (C)
Variable modifications:

T10    : Phospho (ST), with neutral losses 97.9769(shown in table), 0.0000
Ions Score: 19  Expect: 28
Matches (Bold Red): 27/120 fragment ions using 53 most intense peaks
```

#	a	a++	b	b++	Seq.	y	y++	y*	y*++	#
1	72.0808	36.5440	100.0757	50.5415	V					11
2	**219.1492**	**110.0782**	**247.1441**	124.0757	F	1026.5115	513.7594	1009.4850	505.2461	10
3	**276.1707**	138.5890	**304.1656**	152.5864	G	**879.4431**	440.2252	862.4166	431.7119	9
4	**413.2296**	207.1184	**441.2245**	221.1159	H	822.4217	**411.7145**	805.3951	403.2012	8
5	510.2823	255.6448	538.2772	269.6423	P	**685.3628**	343.1850	**668.3362**	334.6717	7
6	581.3194	291.1634	609.3144	305.1608	A	588.3100	**294.6586**	571.2834	286.1454	6
7	**668.3515**	334.6794	696.3464	348.6768	S	517.2729	**259.1401**	500.2463	250.6268	5
8	**769.3991**	385.2032	797.3941	399.2007	T	430.2409	215.6241	**413.2143**	207.1108	4
9	**840.4363**	**420.7218**	868.4312	434.7192	A	329.1932	165.1002	312.1666	156.5869	3
10	923.4734	462.2403	951.4683	476.2378	T	258.1561	129.5817	241.1295	121.0684	2
11					R	**175.1190**	88.0631	158.0924	79.5498	1

MS/MS Fragmentation of GSDYASKETER
Found in gi|15226610, ATPDIL2-1/MEE30/UNE5 (PDI-LIKE 2-1); thiol-disulfide exchange intermediate

Match to Query 78: 1321.565008 from(661.789780,2+) intensity(1968.0000)
Title: 7: Sum of 3 scans in range 1232 (rt=23.5899, f=3, i=5) to 1238 (rt=23.8315, f=3, i=7)

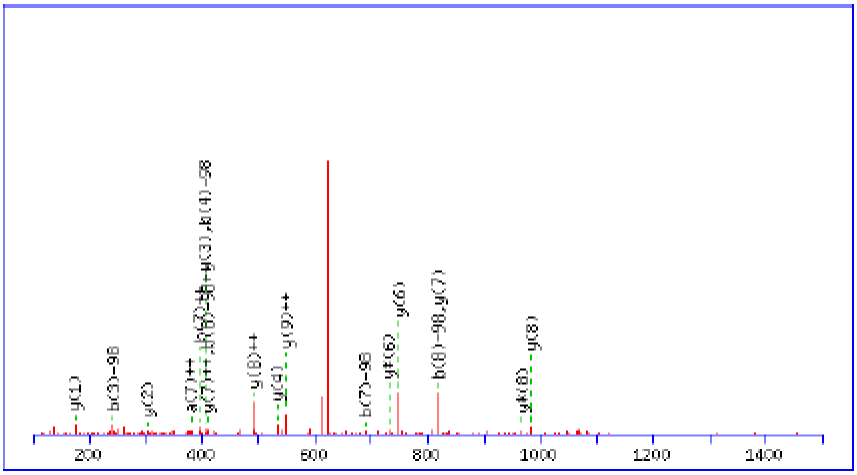

Monoisotopic mass of neutral peptide Mr(calc): 1321.5187
Fixed modifications: Carbamidomethyl (C)
Variable modifications:

S2 : Phospho (ST), with neutral losses 97.9769(shown in table), 0.0000
Ions Score: 28 **Expect:** 3.9
Matches (**Bold Red**): 19/152 fragment ions using 49 most intense peaks

#	a	a++	a*	a*++	b	b++	b*	b*++	Seq.	y	y++	y*	y*++	#
1	30.0338	15.5206			58.0287	29.5180			G					11
2	99.0553	50.0313			127.0502	64.0287			S	1167.5277	584.2675	1150.5011	575.7542	10
3	214.0822	107.5448			**242.0771**	121.5422			D	1098.5062	**549.7567**	1081.4796	541.2435	9
4	377.1456	189.0764			**405.1405**	203.0739			Y	**983.4793**	**492.2433**	**966.4527**	483.7300	8
5	448.1827	224.5950			476.1776	238.5924			A	**820.4159**	**410.7116**	803.3894	402.1983	7
6	535.2147	268.1110			563.2096	282.1084			S	**749.3788**	375.1930	**732.3523**	366.6798	6
7	663.3097	332.1585	646.2831	323.6452	**691.3046**	346.1559	674.2780	337.6427	K	662.3468	331.6770	645.3202	323.1638	5
8	792.3523	396.6798	775.3257	388.1665	**820.3472**	**410.6772**	803.3206	402.1639	E	**534.2518**	267.6295	517.2253	259.1163	4
9	893.3999	447.2036	876.3734	438.6903	921.3948	461.2011	904.3683	452.6878	T	**405.2092**	203.1082	388.1827	194.5950	3
10	1022.4425	511.7249	1005.4160	503.2116	1050.4374	525.7224	1033.4109	517.2091	E	**304.1615**	152.5844	287.1350	144.0711	2
11									R	**175.1190**	88.0631	158.0924	79.5498	1

MS/MS Fragmentation of **ELVAASEDEKK**
Found in gi|15226610, ATPDIL2-1/MEE30/UNE5 (PDI-LIKE 2-1); thiol-disulfide exchange intermediate

Match to Query 73: 1297.623708 from(649.819130,2+) intensity(5370.0000)
Title: 20: Sum of 3 scans in range 1292 (rt=25.6891, f=2, i=51) to 1298 (rt=25.9374, f=2, i=53)

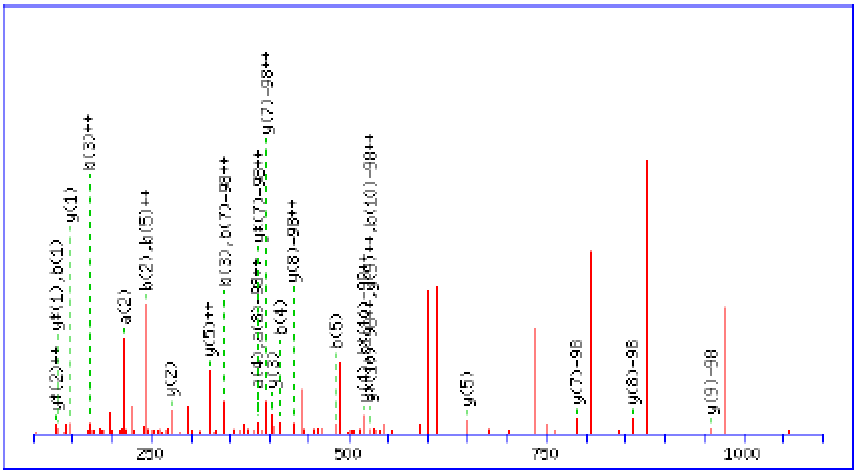

Monoisotopic mass of neutral peptide Mr(calc): 1297.5802
Fixed modifications: Carbamidomethyl (C)
Variable modifications:

S6 : Phospho (ST), with neutral losses 97.9769(shown in table), 0.0000
Ions Score: 42 **Expect:** 0.12
Matches (**Bold Red**): 29/128 fragment ions using 45 most intense peaks

#	a	a++	a*	a*++	b	b++	b*	b*++	Seq.	y	y++	y*	y*++	#
1	102.0550	51.5311			**130.0499**	65.5286			E					11
2	**215.1390**	108.0731			**243.1339**	122.0706			L	1071.5681	536.2877	1054.5415	**527.7744**	10
3	314.2074	157.6074			**342.2023**	**171.6048**			V	**958.4840**	479.7456	941.4574	471.2324	9
4	**385.2445**	193.1259			**413.2395**	207.1234			A	**859.4156**	**430.2114**	842.3890	421.6982	8
5	456.2817	228.6445			**484.2766**	**242.6419**			A	**788.3785**	**394.6929**	771.3519	**386.1796**	7
6	525.3031	263.1552			553.2980	277.1527			S	717.3414	359.1743	700.3148	350.6610	6
7	654.3457	327.6765			682.3406	**341.6740**			E	**648.3199**	**324.6636**	631.2933	316.1503	5
8	769.3727	**385.1900**			797.3676	399.1874			D	**519.2773**	260.1423	502.2508	251.6290	4
9	898.4152	449.7113			926.4102	463.7087			E	**404.2504**	202.6288	387.2238	194.1155	3
10	1026.5102	513.7587	1009.4837	505.2455	1054.5051	**527.7562**	1037.4786	**519.2429**	K	**275.2078**	138.1075	258.1812	**129.5942**	2
11									K	**147.1128**	74.0600	**130.0863**	65.5468	1

50c

MS/MS Fragmentation of AGHDYDGGRDLDDFVSFINEK
Found in gi|15226610, ATPDIL2-1/MEE30/UNE5 (PDI-LIKE 2-1); thiol-disulfide exchange intermediate

Match to Query 148: 2449.087662 from(817.369830,3+) intensity(7295.0000)

Title: 120: Scan 1666 (rt=38.6842, f=2, i=147)

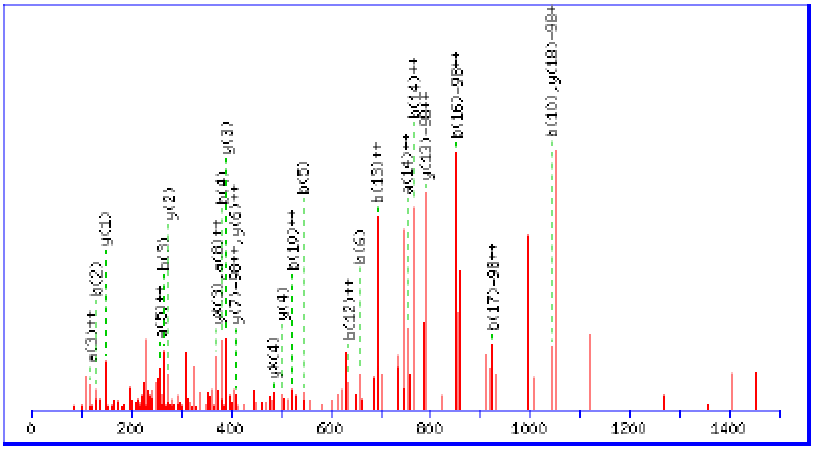

Monoisotopic mass of neutral peptide Mr(calc): 2449.0224
Fixed modifications: Carbamidomethyl (C)
Variable modifications:
S16 : Phospho (ST), with neutral losses 97.9769(shown in table), 0.0000
Ions Score: 35 **Expect:** 0.92
Matches (Bold Red): 26/308 fragment ions using 45 most intense peaks

#	a	a++	a*	a*++	b	b++	b*	b*++	Seq.	y	y++	y*	y*++	#
1	44.0495	22.5284			72.0444	36.5258			A					21
2	101.0709	51.0391			**129.0659**	65.0366			G	2281.0156	1141.0114	2263.9891	1132.4982	20
3	238.1299	**119.5686**			**266.1248**	133.5660			H	2223.9941	1112.5007	2206.9676	1103.9874	19
4	353.1568	177.0820			**381.1517**	191.0795			D	2086.9352	**1043.9713**	2069.9087	1035.4580	18
5	516.2201	**258.6137**			**544.2150**	272.6112			Y	1971.9083	986.4578	1954.8817	977.9445	17
6	631.2471	316.1272			**659.2420**	330.1246			D	1808.8450	904.9261	1791.8184	896.4128	16
7	688.2685	344.6379			716.2634	358.6354			G	1693.8180	847.4126	1676.7915	838.8994	15
8	745.2900	**373.1486**			773.2849	387.1461			G	1636.7966	818.9019	1619.7700	810.3886	14
9	901.3911	451.1992	884.3646	442.6859	929.3860	465.1966	912.3595	456.6834	R	1579.7751	**790.3912**	1562.7485	781.8779	13
10	1016.4180	508.7127	999.3915	500.1994	**1044.4130**	**522.7101**	1027.3864	514.1968	D	1423.6740	712.3406	1406.6474	703.8274	12
11	1129.5021	565.2547	1112.4756	556.7414	1157.4970	579.2522	1140.4705	570.7389	L	1308.6470	654.8272	1291.6205	646.3139	11
12	1244.5291	622.7682	1227.5025	614.2549	1272.5240	**636.7656**	1255.4974	628.2523	D	1195.5630	598.2851	1178.5364	589.7718	10
13	1359.5560	680.2816	1342.5294	671.7684	1387.5509	**694.2791**	1370.5244	685.7658	D	1080.5360	540.7717	1063.5095	532.2584	9
14	1506.6244	**753.8158**	1489.5979	745.3026	1534.6193	**767.8133**	1517.5928	759.3000	F	965.5091	483.2582	948.4825	474.7449	8
15	1605.6928	803.3501	1588.6663	794.8368	1633.6877	817.3475	1616.6612	808.8342	V	818.4407	**409.7240**	801.4141	401.2107	7
16	1674.7143	837.8608	1657.6877	829.3475	1702.7092	**851.8582**	1685.6826	843.3450	S	719.3723	360.1898	702.3457	351.6765	6
17	1821.7827	911.3950	1804.7561	902.8817	1849.7776	**925.3924**	1832.7511	916.8792	F	650.3508	325.6790	633.3243	317.1658	5
18	1934.8668	967.9370	1917.8402	959.4237	1962.8617	981.9345	1945.8351	973.4212	I	**503.2824**	252.1448	**486.2558**	243.6316	4
19	2048.9097	1024.9585	2031.8831	1016.4452	2076.9046	1038.9559	2059.8781	1030.4427	N	**390.1983**	195.6028	**373.1718**	187.0895	3
20	2177.9523	1089.4798	2160.9257	1080.9665	2205.9472	1103.4772	2188.9206	1094.9640	E	**276.1554**	138.5813	259.1288	130.0681	2
21									K	**147.1128**	74.0600	130.0863	65.5468	1

MS/MS Fragmentation of **TILFAVPGAFTPTCSQK**
Found in gi|15231718, peroxiredoxin type 2, putative [Arabidopsis thaliana]

Match to Query 83: 1916.958688 from(959.486620,2+) intensity(3775.0000)
Title: 88: Sum of 4 scans in range 1881 (rt=42.4339, f=2, i=241) to 1884 (rt=42.5431, f=2, i=244)

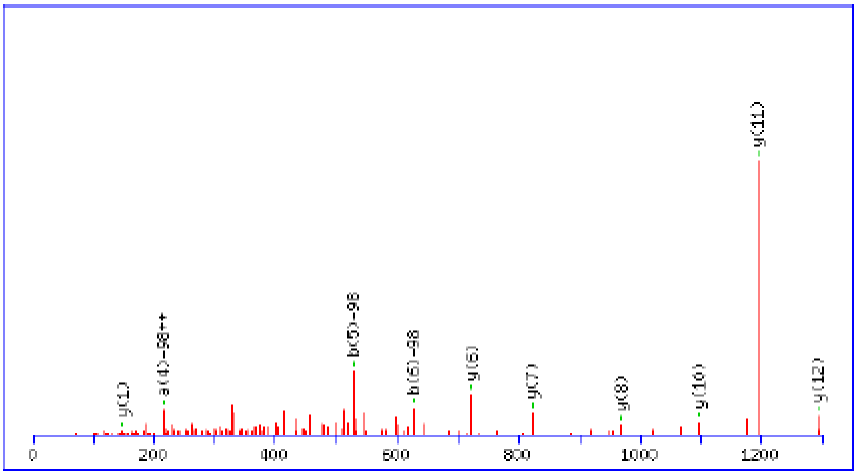

Monoisotopic mass of neutral peptide Mr(calc): 1916.9108
Fixed modifications: Carbamidomethyl (C)
Variable modifications:

T1 : Phospho (ST), with neutral losses 97.9769(shown in table), 0.0000
Ions Score: 49 **Expect:** 0.035
Matches (**Bold Red**): 10/200 fragment ions using 16 most intense peaks

#	a	a++	a*	a*++	b	b++	b*	b*++	Seq.	y	y++	y*	y*++	#
1	56.0495	28.5284			84.0444	42.5258			T					17
2	169.1335	85.0704			197.1284	99.0679			I	1736.9040	868.9556	1719.8775	860.4424	16
3	282.2176	141.6124			310.2125	155.6099			L	1623.8199	812.4136	1606.7934	803.9003	15
4	429.2860	**215.1466**			457.2809	229.1441			F	1510.7359	755.8716	1493.7093	747.3583	14
5	500.3231	250.6652			**528.3180**	264.6627			A	1363.6675	682.3374	1346.6409	673.8241	13
6	599.3915	300.1994			**627.3865**	314.1969			V	**1292.6304**	646.8188	1275.6038	638.3055	12
7	696.4443	348.7258			724.4392	362.7232			P	**1193.5619**	597.2846	1176.5354	588.7713	11
8	753.4658	377.2365			781.4607	391.2340			G	**1096.5092**	548.7582	1079.4826	540.2450	10
9	824.5029	412.7551			852.4978	426.7525			A	1039.4877	520.2475	1022.4612	511.7342	9
10	971.5713	486.2893			999.5662	500.2867			F	**968.4506**	484.7289	951.4240	476.2157	8
11	1072.6190	536.8131			1100.6139	550.8106			T	**821.3822**	411.1947	804.3556	402.6815	7
12	1169.6717	585.3395			1197.6667	599.3370			P	**720.3345**	360.6709	703.3080	352.1576	6
13	1270.7194	635.8633			1298.7143	649.8608			T	623.2817	312.1445	606.2552	303.6312	5
14	1430.7501	715.8787			1458.7450	729.8761			C	522.2341	261.6207	505.2075	253.1074	4
15	1517.7821	759.3947			1545.7770	773.3921			S	362.2034	181.6053	345.1769	173.0921	3
16	1645.8407	823.4240	1628.8141	814.9107	1673.8356	837.4214	1656.8090	828.9082	Q	275.1714	138.0893	258.1448	129.5761	2
17									K	**147.1128**	74.0600	130.0863	65.5468	1

MS/MS Fragmentation of VLNLEEGGAFTNSSAEDMLK
Found in gi|15231718, peroxiredoxin type 2, putative [Arabidopsis thaliana]

Match to Query 84: 2220.020848 from(1111.017700,2+) intensity(3954.0000)
Title: 61: Sum of 4 scans in range 1715 (rt=37.0621, f=3, i=52) to 1720 (rt=37.2581, f=3, i=55)

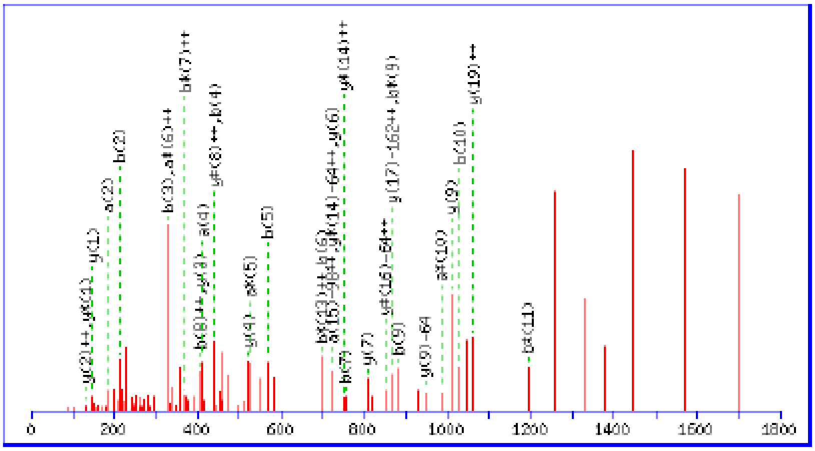


```
Monoisotopic mass of neutral peptide Mr(calc): 2219.9657
Fixed modifications: Carbamidomethyl (C)
Variable modifications:
T11    : Phospho (ST), with neutral losses 0.0000(shown in table), 97.9769
M18    : Oxidation (M), with neutral losses 0.0000(shown in table), 63.9983
Ions Score: 44  Expect: 0.12
```

Matches (**Bold Red**): 34/472 fragment ions using 42 most intense peaks

#	a	a++	a*	a*++	b	b++	b*	b*++	Seq.	y	y++	y*	y*++	#
1	72.0808	36.5440			100.0757	50.5415			V					20
2	**185.1648**	93.0861			**213.1598**	107.0835			L	2121.9046	**1061.4559**	2104.8781	1052.9427	19
3	299.2078	150.1075	282.1812	141.5942	**327.2027**	164.1050	310.1761	155.5917	N	2008.8205	1004.9139	1991.7940	996.4006	18
4	**412.2918**	206.6496	395.2653	198.1363	**440.2867**	220.6470	423.2602	212.1337	L	1894.7776	947.8924	1877.7511	939.3792	17
5	541.3344	271.1708	**524.3079**	262.6576	**569.3293**	285.1683	552.3028	276.6550	E	1781.6936	891.3504	1764.6670	882.8371	16
6	670.3770	335.6921	653.3505	**327.1789**	**698.3719**	349.6896	681.3454	341.1763	E	1652.6510	826.8291	1635.6244	818.3158	15
7	727.3985	364.2029	710.3719	355.6896	**755.3934**	378.2003	738.3668	**369.6871**	G	1523.6084	762.3078	1506.5818	**753.7945**	14
8	784.4199	392.7136	767.3934	384.2003	812.4149	**406.7111**	795.3883	398.1978	G	1466.5869	733.7971	1449.5604	725.2838	13
9	855.4571	428.2322	838.4305	419.7189	**883.4520**	442.2296	**866.4254**	433.7164	A	1409.5654	705.2864	1392.5389	696.7731	12
10	1002.5255	501.7664	**985.4989**	493.2531	**1030.5204**	515.7638	1013.4938	507.2506	F	1338.5283	669.7678	1321.5018	661.2545	11
11	1183.5395	592.2734	1166.5129	583.7601	1211.5344	606.2708	**1194.5078**	597.7576	T	1191.4599	596.2336	1174.4334	587.7203	10
12	1297.5824	649.2948	1280.5559	640.7816	1325.5773	663.2923	1308.5508	654.7790	N	**1010.4459**	505.7266	993.4194	497.2133	9
13	1384.6144	692.8109	1367.5879	684.2976	1412.6093	706.8083	1395.5828	**698.2950**	S	896.4030	448.7051	879.3764	**440.1919**	8
14	1471.6465	736.3269	1454.6199	727.8136	1499.6414	750.3243	1482.6148	741.8111	S	**809.3710**	405.1891	792.3444	396.6758	7
15	1542.6836	771.8454	1525.6570	763.3321	1570.6785	785.8429	1553.6519	777.3296	A	**722.3389**	361.6731	705.3124	353.1598	6
16	1671.7262	836.3667	1654.6996	827.8534	1699.7211	850.3642	1682.6945	841.8509	E	651.3018	326.1545	634.2753	317.6413	5
17	1786.7531	893.8802	1769.7266	885.3669	1814.7480	907.8776	1797.7215	899.3644	D	**522.2592**	261.6332	505.2327	253.1200	4
18	1933.7885	967.3979	1916.7620	958.8846	1961.7834	981.3954	1944.7569	972.8821	M	**407.2323**	204.1198	390.2057	195.6065	3
19	2046.8726	1023.9399	2029.8460	1015.4267	2074.8675	1037.9374	2057.8409	1029.4241	L	260.1969	**130.6021**	243.1703	122.0888	2
20									K	**147.1128**	74.0600	**130.0863**	65.5468	1

MS/MS Fragmentation of **DLKLDSIVMGSR**
Found in **gi|30693971**, universal stress protein (USP) family protein [Arabidopsis thaliana]

Match to Query 97: 1428.630708 from(715.322630,2+) intensity(6960.0000)
Title: 98: Sum of 2 scans in range 1390 (rt=33.3454, f=4, i=110) to 1393 (rt=33.4681, f=4, i=111)

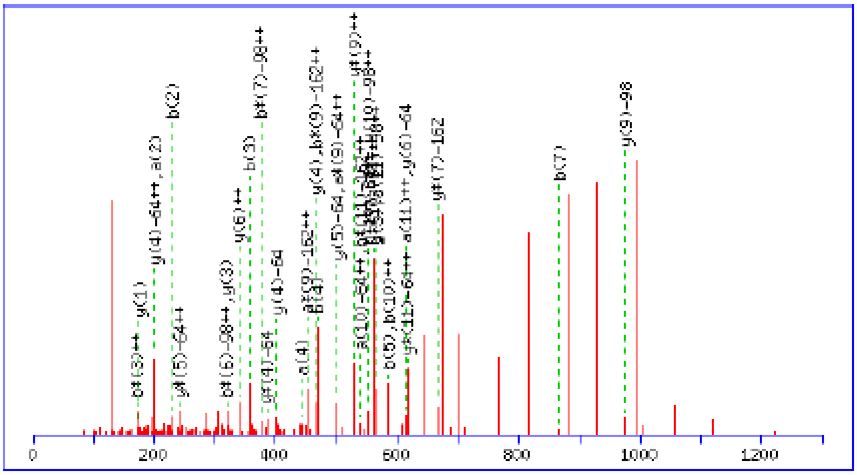

Monoisotopic mass of neutral peptide Mr(calc): 1428.6684
Fixed modifications: Carbamidomethyl (C)
Variable modifications:

S6 : Phospho (ST), with neutral losses 0.0000(shown in table), 97.9769
M9 : Oxidation (M), with neutral losses 63.9983(shown in table), 0.0000
Ions Score: 25 **Expect:** 8.7
Matches (**Bold Red**): 36/292 fragment ions using 52 most intense peaks

#	a	a++	a*	a*++	b	b++	b*	b*++	Seq.	y	y++	y*	y*++	#
1	88.0393	44.5233			116.0342	58.5207			D					12
2	**201.1234**	101.0653			**229.1183**	115.0628			L	1250.6504	625.8289	1233.6239	**617.3156**	11
3	329.2183	165.1128	312.1918	156.5995	**357.2132**	179.1103	340.1867	**170.5970**	K	1137.5664	569.2868	1120.5398	**560.7735**	10
4	**442.3024**	221.6548	425.2758	213.1416	**470.2973**	235.6523	453.2708	227.1390	L	1009.4714	505.2393	992.4449	496.7261	9
5	557.3293	279.1683	540.3028	270.6550	**585.3243**	293.1658	568.2977	284.6525	D	896.3873	448.6973	879.3608	440.1840	8
6	724.3277	362.6675	707.3011	354.1542	752.3226	376.6649	735.2961	368.1517	S	781.3604	391.1838	764.3338	382.6706	7
7	837.4118	419.2095	820.3852	410.6962	**865.4067**	433.2070	848.3801	424.6937	I	**614.3620**	307.6847	597.3355	299.1714	6
8	936.4802	468.7437	919.4536	460.2304	964.4751	482.7412	947.4485	474.2279	V	**501.2780**	251.1426	484.2514	**242.6294**	5
9	1019.5173	510.2623	1002.4907	**501.7490**	1047.5122	524.2597	1030.4857	515.7465	M	**402.2096**	**201.6084**	**385.1830**	193.0951	4
10	1076.5388	**538.7730**	1059.5122	530.2597	1104.5337	**552.7705**	1087.5071	544.2572	G	**319.1724**	160.0899	302.1459	151.5766	3
11	1163.5708	582.2890	1146.5442	573.7758	1191.5657	596.2865	1174.5391	587.7732	S	262.1510	131.5791	245.1244	123.0659	2
12									R	**175.1190**	88.0631	158.0924	79.5498	1

MS/MS Fragmentation of INTDESPNTANR
Found in gi|15232567, ATHM4 (Arabidopsis thioredoxin M-type 4); thiol-disulfide exchange intermediate

Match to Query 85: 1410.652768 from(706.333660,2+) intensity(3982.0000)
Title: 9: Sum of 3 scans in range 1106 (rt=21.6825, f=2, i=27) to 1112 (rt=21.9286, f=2, i=29)

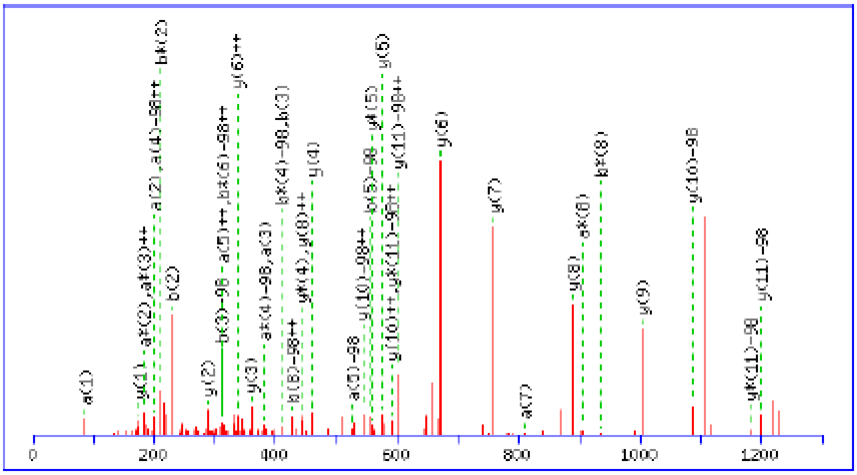

Monoisotopic mass of neutral peptide Mr(calc): 1410.5776
Fixed modifications: Carbamidomethyl (C)
Variable modifications:

T3 : Phospho (ST), with neutral losses 97.9769(shown in table), 0.0000
Ions Score: 64 **Expect:** 0.00099
Matches (Bold Red): 40/208 fragment ions using 71 most intense peaks

#	a	a++	a*	a*++	b	b++	b*	b*++	Seq.	y	y++	y*	y*++	#
1	**86.0964**	43.5519			114.0913	57.5493			I					12
2	**200.1394**	100.5733	**183.1128**	92.0600	**228.1343**	114.5708	**211.1077**	106.0575	N	**1200.5240**	**600.7656**	**1183.4974**	**592.2523**	11
3	283.1765	142.0919	266.1499	133.5786	**311.1714**	156.0893	294.1448	147.5761	T	**1086.4810**	**543.7442**	1069.4545	535.2309	10
4	398.2034	**199.6053**	**381.1769**	191.0921	426.1983	213.6028	**409.1718**	205.0895	D	**1003.4439**	502.2256	986.4174	493.7123	9
5	**527.2460**	264.1266	510.2194	255.6134	**555.2409**	278.1241	538.2144	269.6108	E	**888.4170**	**444.7121**	871.3904	436.1989	8
6	614.2780	307.6426	597.2515	299.1294	642.2729	321.6401	625.2464	**313.1268**	S	**759.3744**	380.1908	742.3478	371.6776	7
7	711.3308	356.1690	694.3042	347.6558	739.3257	370.1665	722.2992	361.6532	P	**672.3424**	**336.6748**	655.3158	328.1615	6
8	825.3737	413.1905	808.3472	404.6772	853.3686	**427.1880**	836.3421	418.6747	N	**575.2896**	288.1484	**558.2630**	279.6352	5
9	926.4214	463.7143	909.3948	455.2011	954.4163	477.7118	937.3898	469.1985	T	**461.2467**	231.1270	**444.2201**	222.6137	4
10	997.4585	499.2329	980.4320	490.7196	1025.4534	513.2303	1008.4269	504.7171	A	**360.1990**	180.6031	343.1724	172.0899	3
11	1111.5014	556.2544	1094.4749	547.7411	1139.4963	570.2518	1122.4698	561.7385	N	**289.1619**	145.0846	272.1353	136.5713	2
12									R	**175.1190**	88.0631	158.0924	79.5498	1

MS/MS Fragmentation of TTLTSSLDKFLP
Found in gi|6539610, thioredoxin m2 [Arabidopsis thaliana]

Match to Query 75: 1401.751248 from(701.882900,2+) intensity(8335.0000)
Title: 115: Sum of 4 scans in range 1717 (rt=41.2027, f=2, i=186) to 1720 (rt=41.3054, f=2, i=189)

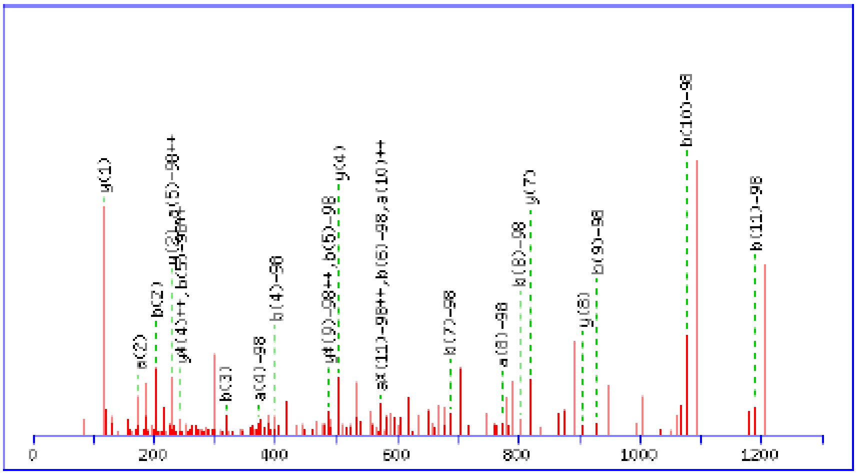

Monoisotopic mass of neutral peptide Mr(calc): 1401.6793
Fixed modifications: Carbamidomethyl (C)
Variable modifications:

T4 : Phospho (ST), with neutral losses 97.9769(shown in table), 0.0000
Ions Score: 49 **Expect:** 0.035
Matches (**Bold Red**): 24/150 fragment ions using 53 most intense peaks

#	a	a^{++}	a*	a^{*++}	b	b^{++}	b*	b^{*++}	Seq.	y	y^{++}	y*	y^{*++}	#
1	74.0600	37.5337			102.0550	51.5311			T					12
2	**175.1077**	88.0575			**203.1026**	102.0550			T	1203.6620	602.3346	1186.6354	593.8213	11
3	288.1918	144.5995			**316.1867**	158.5970			L	1102.6143	551.8108	1085.5877	543.2975	10
4	**371.2289**	186.1181			**399.2238**	200.1155			T	989.5302	495.2687	972.5037	**486.7555**	9
5	458.2609	**229.6341**			**486.2558**	**243.6316**			S	**906.4931**	453.7502	889.4666	445.2369	8
6	545.2929	273.1501			**573.2879**	287.1476			S	**819.4611**	410.2342	802.4345	401.7209	7
7	658.3770	329.6921			**686.3719**	343.6896			L	732.4291	366.7182	715.4025	358.2049	6
8	**773.4040**	387.2056			**801.3989**	401.2031			D	619.3450	310.1761	602.3184	301.6629	5
9	901.4989	451.2531	884.4724	442.7398	**929.4938**	465.2506	912.4673	456.7373	K	**504.3180**	252.6627	487.2915	**244.1494**	4
10	1048.5673	524.7873	1031.5408	516.2740	**1076.5622**	538.7848	1059.5357	530.2715	F	376.2231	188.6152			3
11	1161.6514	581.3293	1144.6248	**572.8161**	**1189.6463**	595.3268	1172.6198	586.8135	L	**229.1547**	115.0810			2
12									P	**116.0706**	58.5389			1

54b

MS/MS Fragmentation of LNTDESPNTPGQYGVR
Found in gi|6539610, thioredoxin m2 [Arabidopsis thaliana]

Match to Query 99: 1826.870868 from(914.442710,2+) intensity(4926.0000)
Title: 29: Sum of 4 scans in range 1280 (rt=26.3602, f=4, i=15) to 1287 (rt=26.6405, f=4, i=18)

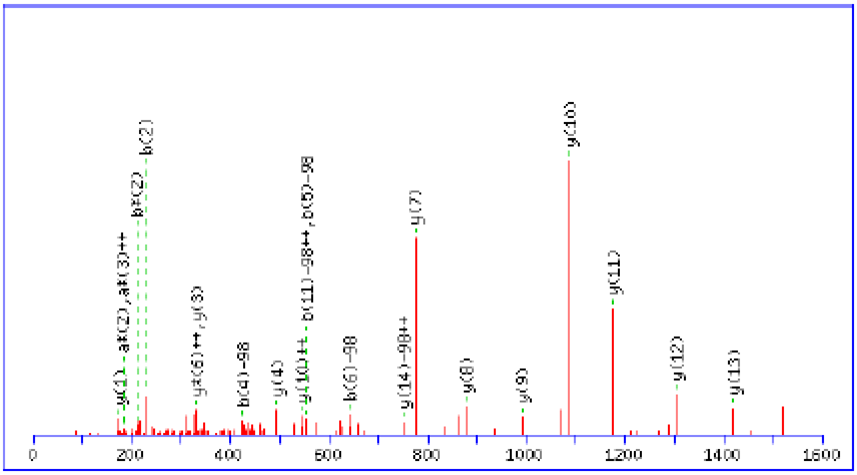


```
Monoisotopic mass of neutral peptide Mr(calc): 1826.7836
Fixed modifications: Carbamidomethyl (C)
Variable modifications:


T3     : Phospho (ST), with neutral losses 97.9769(shown in table), 0.0000
Ions Score: 68  Expect: 0.00047
Matches (Bold Red): 21/288 fragment ions using 30 most intense peaks
```

#	a	a++	a*	a*++	b	b++	b*	b*++	Seq.	y	y++	y*	y*++	#
1	86.0964	43.5519			114.0913	57.5493			L					16
2	200.1394	100.5733	**183.1128**	92.0600	**228.1343**	114.5708	**211.1077**	106.0575	N	1616.7299	808.8686	1599.7034	800.3553	15
3	283.1765	142.0919	266.1499	133.5786	311.1714	156.0893	294.1448	147.5761	T	1502.6870	**751.8471**	1485.6605	743.3339	14
4	398.2034	199.6053	381.1769	191.0921	**426.1983**	213.6028	409.1718	205.0895	D	**1419.6499**	710.3286	1402.6233	701.8153	13
5	527.2460	264.1266	510.2194	255.6134	**555.2409**	278.1241	538.2144	269.6108	E	**1304.6230**	652.8151	1287.5964	644.3018	12
6	614.2780	307.6426	597.2515	299.1294	**642.2729**	321.6401	625.2464	313.1268	S	**1175.5804**	588.2938	1158.5538	579.7805	11
7	711.3308	356.1690	694.3042	347.6558	739.3257	370.1665	722.2992	361.6532	P	**1088.5483**	**544.7778**	1071.5218	536.2645	10
8	825.3737	413.1905	808.3472	404.6772	853.3686	427.1880	836.3421	418.6747	N	**991.4956**	496.2514	974.4690	487.7381	9
9	926.4214	463.7143	909.3948	455.2011	954.4163	477.7118	937.3898	469.1985	T	**877.4526**	439.2300	860.4261	430.7167	8
10	1023.4742	512.2407	1006.4476	503.7274	1051.4691	526.2382	1034.4425	517.7249	P	**776.4050**	388.7061	759.3784	380.1928	7
11	1080.4956	540.7514	1063.4691	532.2382	1108.4905	**554.7489**	1091.4640	546.2356	G	679.3522	340.1797	662.3257	**331.6665**	6
12	1208.5542	604.7807	1191.5276	596.2675	1236.5491	618.7782	1219.5226	610.2649	Q	622.3307	311.6690	605.3042	303.1557	5
13	1371.6175	686.3124	1354.5910	677.7991	1399.6124	700.3099	1382.5859	691.7966	Y	**494.2722**	247.6397	477.2456	239.1264	4
14	1428.6390	714.8231	1411.6124	706.3099	1456.6339	728.8206	1439.6074	720.3073	G	**331.2088**	166.1081	314.1823	157.5948	3
15	1527.7074	764.3573	1510.6809	755.8441	1555.7023	778.3548	1538.6758	769.8415	V	274.1874	137.5973	257.1608	129.0840	2
16									R	**175.1190**	88.0631	158.0924	79.5498	1

MS/MS Fragmentation of SGGLGDLNYPLISDVTK
Found in gi|3121825, 2-Cys peroxiredoxin BAS1, chloroplast precursor (Thiol-specific antioxidant protein)

Match to Query 112: 1827.960448 from(914.987500,2+) intensity(2889.0000)
Title: 119: Sum of 4 scans in range 1651 (rt=39.4038, f=2, i=221) to 1654 (rt=39.5052, f=2, i=224)

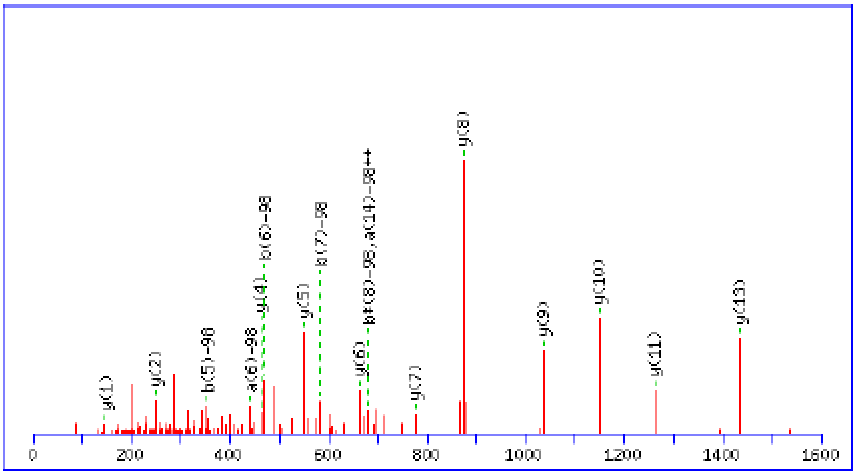

Monoisotopic mass of neutral peptide Mr(calc): 1827.8656
Fixed modifications: Carbamidomethyl (C)
Variable modifications:

S1 : Phospho (ST), with neutral losses 97.9769(shown in table), 0.0000
Ions Score: 70 **Expect:** 0.00031
Matches (Bold Red): 17/264 fragment ions using 31 most intense peaks

#	a	a++	a*	a*++	b	b++	b*	b*++	Seq.	y	y++	y*	y*++	#
1	42.0338	21.5205			70.0287	35.5180			S					17
2	99.0553	50.0313			127.0502	64.0287			G	1661.8745	831.4409	1644.8479	822.9276	16
3	156.0767	78.5420			184.0717	92.5395			G	1604.8530	802.9301	1587.8265	794.4169	15
4	269.1608	135.0840			297.1557	149.0815			L	1547.8316	774.4194	1530.8050	765.9061	14
5	326.1823	163.5948			**354.1772**	177.5922			G	**1434.7475**	717.8774	1417.7209	709.3641	13
6	**441.2092**	221.1082			**469.2041**	235.1057			D	1377.7260	689.3666	1360.6995	680.8534	12
7	554.2933	277.6503			**582.2882**	291.6477			L	**1262.6991**	631.8532	1245.6725	623.3399	11
8	668.3362	334.6717	651.3097	326.1585	696.3311	348.6692	**679.3046**	340.1559	N	**1149.6150**	575.3111	1132.5885	566.7979	10
9	831.3995	416.2034	814.3730	407.6901	859.3945	430.2009	842.3679	421.6876	Y	**1035.5721**	518.2897	1018.5455	509.7764	9
10	928.4523	464.7298	911.4258	456.2165	956.4472	478.7272	939.4207	470.2140	P	**872.5088**	436.7580	855.4822	428.2447	8
11	1041.5364	521.2718	1024.5098	512.7585	1069.5313	535.2693	1052.5047	526.7560	L	**775.4560**	388.2316	758.4294	379.7184	7
12	1154.6204	577.8139	1137.5939	569.3006	1182.6153	591.8113	1165.5888	583.2980	I	**662.3719**	331.6896	645.3454	323.1763	6
13	1241.6525	621.3299	1224.6259	612.8166	1269.6474	635.3273	1252.6208	626.8140	S	**549.2879**	275.1476	532.2613	266.6343	5
14	1356.6794	**678.8433**	1339.6529	670.3301	1384.6743	692.8408	1367.6478	684.3275	D	**462.2558**	231.6316	445.2293	223.1183	4
15	1455.7478	728.3775	1438.7213	719.8643	1483.7427	742.3750	1466.7162	733.8617	V	347.2289	174.1181	330.2023	165.6048	3
16	1556.7955	778.9014	1539.7689	770.3881	1584.7904	792.8988	1567.7639	784.3856	T	**248.1605**	124.5839	231.1339	116.0706	2
17									K	**147.1128**	74.0600	130.0863	65.5468	1

MS/MS Fragmentation of QMGLSDKDIVALSGAHTLGR
Found in gi|15223049, APX1 (ASCORBATE PEROXIDASE 1); L-ascorbate peroxidase [Arabidopsis thaliana]

Match to Query 119: 2164.121562 from(722.381130,3+) intensity(2838.0000)
Title: 74: Sum of 4 scans in range 1424 (rt=32.0625, f=3, i=86) to 1428 (rt=32.2118, f=3, i=89)

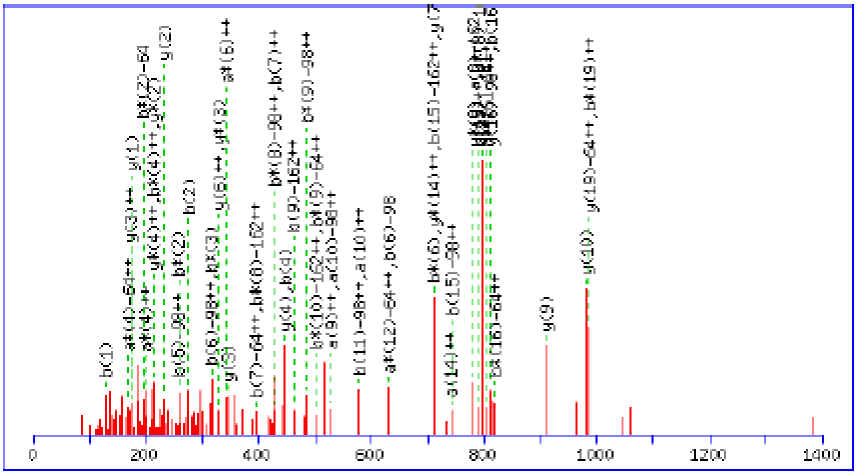

Monoisotopic mass of neutral peptide Mr(calc): 2164.0348
Fixed modifications: Carbamidomethyl (C)
Variable modifications:

M2 : Oxidation (M), with neutral losses 0.0000(shown in table), 63.9983
S5 : Phospho (ST), with neutral losses 97.9769(shown in table), 0.0000
Ions Score: 33 **Expect:** 1.5
Matches (Bold Red): 56/636 fragment ions using 59 most intense peaks

#	a	a++	a*	a*++	b	b++	b*	b*++	Seq.	y	y++	y*	y*++	#
1	101.0709	51.0391	84.0444	42.5258	**129.0659**	65.0366	112.0393	56.5233	Q					20
2	248.1063	124.5568	231.0798	116.0435	**276.1013**	138.5543	**259.0747**	130.0410	M	1939.0066	970.0069	1921.9800	961.4936	19
3	305.1278	153.0675	288.1013	144.5543	333.1227	167.0650	**316.0962**	158.5517	G	1791.9712	896.4892	1774.9446	887.9759	18
4	418.2119	209.6096	401.1853	**201.0963**	**446.2068**	223.6070	429.1802	**215.0938**	L	1734.9497	867.9785	1717.9232	859.4652	17
5	487.2333	244.1203	470.2068	235.6070	515.2282	**258.1178**	498.2017	249.6045	S	1621.8656	**811.4365**	1604.8391	802.9232	16
6	602.2603	301.6338	585.2337	293.1205	**630.2552**	**315.6312**	613.2286	307.1180	D	1552.8442	776.9257	1535.8176	768.4125	15
7	730.3552	365.6813	713.3287	357.1680	758.3501	379.6787	741.3236	371.1654	K	1437.8172	719.4123	1420.7907	**710.8990**	14
8	845.3822	423.1947	828.3556	414.6815	873.3771	437.1922	856.3505	**428.6789**	D	1309.7223	655.3648	1292.6957	646.8515	13
9	958.4662	479.7368	941.4397	471.2235	986.4612	493.7342	969.4346	**485.2209**	I	1194.6953	597.8513	1177.6688	589.3380	12
10	1057.5347	**529.2710**	1040.5081	520.7577	1085.5296	543.2684	1068.5030	534.7551	V	1081.6113	541.3093	1064.5847	532.7960	11
11	1128.5718	564.7895	1111.5452	556.2762	1156.5667	**578.7870**	1139.5401	570.2737	A	**982.5429**	491.7751	965.5163	483.2618	10
12	1241.6558	621.3316	1224.6293	612.8183	1269.6507	635.3290	1252.6242	626.8157	L	**911.5057**	456.2565	894.4792	447.7432	9
13	1328.6879	664.8476	1311.6613	656.3343	1356.6828	678.8450	1339.6562	670.3318	S	**798.4217**	399.7145	**781.3951**	391.2012	8
14	1385.7093	693.3583	1368.6828	684.8450	1413.7042	707.3558	1396.6777	698.8425	G	**711.3896**	356.1985	694.3631	347.6852	7
15	1456.7464	728.8769	1439.7199	720.3636	1484.7414	**742.8743**	1467.7148	734.3610	A	654.3682	**327.6877**	637.3416	319.1745	6
16	1593.8053	797.4063	1576.7788	788.8930	1621.8003	**811.4038**	1604.7737	802.8905	H	583.3311	292.1692	566.3045	283.6559	5
17	1694.8530	847.9302	1677.8265	839.4169	1722.8479	861.9276	1705.8214	853.4143	T	**446.2722**	223.6397	429.2456	**215.1264**	4
18	1807.9371	904.4722	1790.9105	895.9589	1835.9320	918.4696	1818.9055	909.9564	L	**345.2245**	**173.1159**	**328.1979**	164.6026	3
19	1864.9586	932.9829	1847.9320	924.4696	1892.9535	946.9804	1875.9269	938.4671	G	**232.1404**	116.5738	**215.1139**	108.0606	2
20									R	**175.1190**	88.0631	158.0924	79.5498	1

MS/MS Fragmentation of AITQYLAEEYSEKGEK
Found in gi|20197312, glutathione S-transferase (GST6) [Arabidopsis thaliana]

Match to Query 79: 1937.844688 from(969.929620,2+) intensity(3336.0000)
Title: 54: Sum of 4 scans in range 1406 (rt=31.1518, f=4, i=44) to 1411 (rt=31.3428, f=4, i=47)

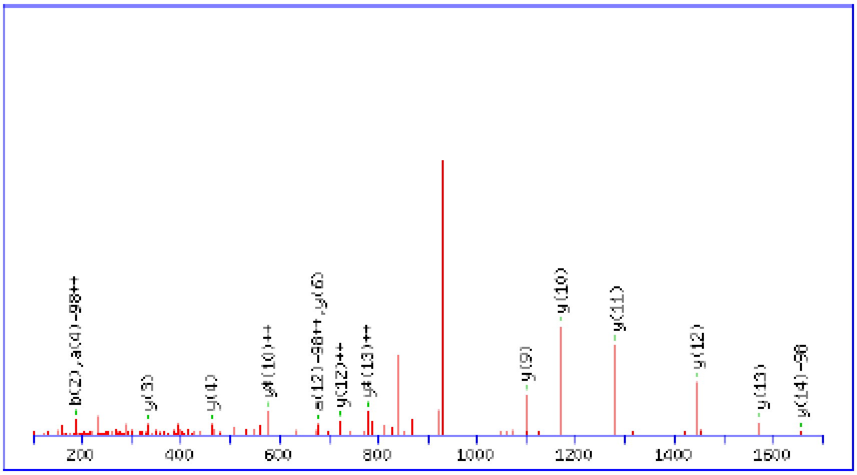

Monoisotopic mass of neutral peptide Mr(calc): 1937.8659
Fixed modifications: Carbamidomethyl (C)
Variable modifications:

T3 : Phospho (ST), with neutral losses 97.9769(shown in table), 0.0000
Ions Score: 71 **Expect:** 0.00023
Matches (Bold Red): 15/276 fragment ions using 18 most intense peaks

#	a	a++	a*	a*++	b	b++	b*	b*++	Seq.	y	y++	y*	y*++	#
1	44.0495	22.5284			72.0444	36.5258			A					16
2	157.1335	79.0704			**185.1285**	93.0679			I	1769.8592	885.4332	1752.8327	876.9200	15
3	240.1706	120.5890			268.1656	134.5864			T	**1656.7752**	828.8912	1639.7486	820.3779	14
4	368.2292	**184.6182**	351.2027	176.1050	396.2241	198.6157	379.1976	190.1024	Q	**1573.7380**	787.3727	1556.7115	**778.8594**	13
5	531.2926	266.1499	514.2660	257.6366	559.2875	280.1474	542.2609	271.6341	Y	**1445.6795**	**723.3434**	1428.6529	714.8301	12
6	644.3766	322.6919	627.3501	314.1787	672.3715	336.6894	655.3450	328.1761	L	**1282.6161**	641.8117	1265.5896	633.2984	11
7	715.4137	358.2105	698.3872	349.6972	743.4086	372.2080	726.3821	363.6947	A	**1169.5321**	585.2697	1152.5055	**576.7564**	10
8	844.4563	422.7318	827.4298	414.2185	872.4512	436.7293	855.4247	428.2160	E	**1098.4950**	549.7511	1081.4684	541.2378	9
9	973.4989	487.2531	956.4724	478.7398	1001.4938	501.2506	984.4673	492.7373	E	969.4524	485.2298	952.4258	476.7165	8
10	1136.5622	568.7848	1119.5357	560.2715	1164.5572	582.7822	1147.5306	574.2689	Y	840.4098	420.7085	823.3832	412.1953	7
11	1223.5943	612.3008	1206.5677	603.7875	1251.5892	626.2982	1234.5626	617.7850	S	**677.3464**	339.1769	660.3199	330.6636	6
12	1352.6369	**676.8221**	1335.6103	668.3088	1380.6318	690.8195	1363.6052	682.3063	E	590.3144	295.6608	573.2879	287.1476	5
13	1480.7318	740.8696	1463.7053	732.3563	1508.7267	754.8670	1491.7002	746.3537	K	**461.2718**	231.1396	444.2453	222.6263	4
14	1537.7533	769.3803	1520.7267	760.8670	1565.7482	783.3777	1548.7217	774.8645	G	**333.1769**	167.0921	316.1503	158.5788	3
15	1666.7959	833.9016	1649.7693	825.3883	1694.7908	847.8990	1677.7643	839.3858	E	276.1554	138.5813	259.1288	130.0681	2
16									K	147.1128	74.0600	130.0863	65.5468	1

MS/MS Fragmentation of SFGVLIPDQGIALR
Found in gi|18415155, 2-cys peroxiredoxin, chloroplast, putative [Arabidopsis thaliana]

Match to Query 74: 1564.724728 from(783.369640,2+) intensity(4943.0000)
Title: 100: Sum of 4 scans in range 1790 (rt=41.9823, f=2, i=218) to 1793 (rt=42.0849, f=2, i=221)

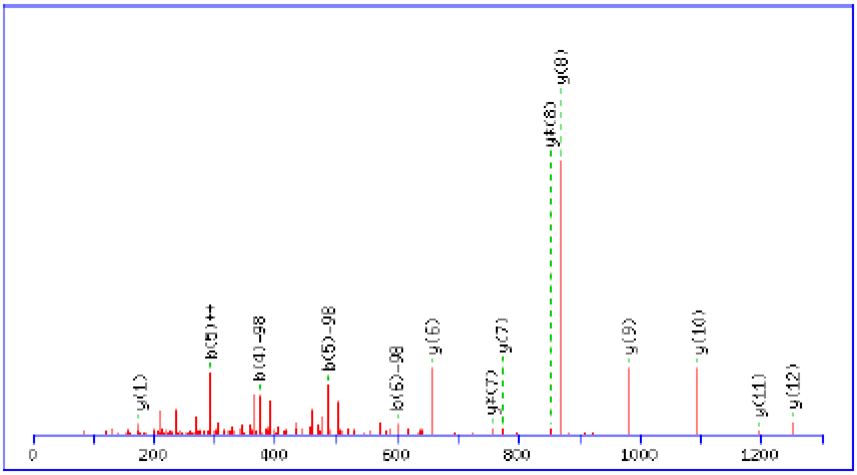

Monoisotopic mass of neutral peptide Mr(calc): 1564.8014
Fixed modifications: Carbamidomethyl (C)
Variable modifications:

S1 : Phospho (ST), with neutral losses 97.9769(shown in table), 0.0000
Ions Score: 55 **Expect:** 0.0074
Matches (Bold Red): 14/196 fragment ions using 24 most intense peaks

#	a	a++	a*	a*++	b	b++	b*	b*++	Seq.	y	y++	y*	y*++	#
1	42.0338	21.5205			70.0287	35.5180			S					14
2	189.1022	95.0548			217.0971	109.0522			F	1398.8104	699.9088	1381.7838	691.3955	13
3	246.1237	123.5655			274.1186	137.5629			G	**1251.7419**	626.3746	1234.7154	617.8613	12
4	345.1921	173.0997			**373.1870**	187.0971			V	**1194.7205**	597.8639	1177.6939	589.3506	11
5	458.2762	229.6417			**486.2711**	243.6392			L	**1095.6521**	548.3297	1078.6255	539.8164	10
6	571.3602	286.1838			**599.3552**	300.1812			I	**982.5680**	491.7876	965.5415	483.2744	9
7	668.4130	334.7101			696.4079	348.7076			P	**869.4839**	435.2456	**852.4574**	426.7323	8
8	783.4399	392.2236			811.4349	406.2211			D	**772.4312**	386.7192	**755.4046**	378.2060	7
9	911.4985	456.2529	894.4720	447.7396	939.4934	470.2504	922.4669	461.7371	Q	**657.4042**	329.2058	640.3777	320.6925	6
10	968.5200	484.7636	951.4934	476.2504	996.5149	498.7611	979.4884	490.2478	G	529.3457	265.1765	512.3191	256.6632	5
11	1081.6040	541.3057	1064.5775	532.7924	1109.5990	555.3031	1092.5724	546.7898	I	472.3242	236.6657	455.2976	228.1525	4
12	1152.6412	576.8242	1135.6146	568.3109	1180.6361	590.8217	1163.6095	582.3084	A	359.2401	180.1237	342.2136	171.6104	3
13	1265.7252	633.3663	1248.6987	624.8530	1293.7201	647.3637	1276.6936	638.8504	L	288.2030	144.6051	271.1765	136.0919	2
14									R	**175.1190**	88.0631	158.0924	79.5498	1

59b

MS/MS Fragmentation of SGGLGDLNYPLVSDITK
Found in gi|18415155, 2-cys peroxiredoxin, chloroplast, putative [Arabidopsis thaliana]

Match to Query 91: 1827.762028 from(914.888290,2+) intensity(2764.0000)
Title: 98: Sum of 4 scans in range 1768 (rt=41.4156, f=2, i=210) to 1771 (rt=41.5182, f=2, i=213)

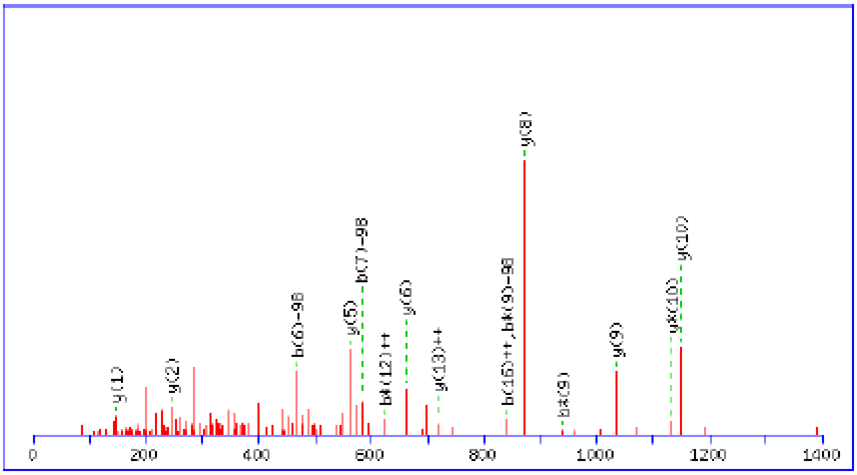

Monoisotopic mass of neutral peptide Mr(calc): 1827.8656
Fixed modifications: Carbamidomethyl (C)
Variable modifications:

S1 : Phospho (ST), with neutral losses 97.9769(shown in table), 0.0000
Ions Score: 33 **Expect:** 1.3
Matches (**Bold Red**): 15/264 fragment ions using 25 most intense peaks

#	a	a++	a*	a*++	b	b++	b*	b*++	Seq.	y	y++	y*	y*++	#
1	42.0338	21.5205			70.0287	35.5180			S					17
2	99.0553	50.0313			127.0502	64.0287			G	1661.8745	831.4409	1644.8479	822.9276	16
3	156.0767	78.5420			184.0717	92.5395			G	1604.8530	802.9301	1587.8265	794.4169	15
4	269.1608	135.0840			297.1557	149.0815			L	1547.8316	774.4194	1530.8050	765.9061	14
5	326.1823	163.5948			354.1772	177.5922			G	1434.7475	**717.8774**	1417.7209	709.3641	13
6	441.2092	221.1082			**469.2041**	235.1057			D	1377.7260	689.3666	1360.6995	680.8534	12
7	554.2933	277.6503			**582.2882**	291.6477			L	1262.6991	631.8532	1245.6725	623.3399	11
8	668.3362	334.6717	651.3097	326.1585	696.3311	348.6692	679.3046	340.1559	N	**1149.6150**	575.3111	**1132.5885**	566.7979	10
9	831.3995	416.2034	814.3730	407.6901	859.3945	430.2009	**842.3679**	421.6876	Y	**1035.5721**	518.2897	1018.5455	509.7764	9
10	928.4523	464.7298	911.4258	456.2165	956.4472	478.7272	939.4207	470.2140	P	**872.5088**	436.7580	855.4822	428.2447	8
11	1041.5364	521.2718	1024.5098	512.7585	1069.5313	535.2693	1052.5047	526.7560	L	775.4560	388.2316	758.4294	379.7184	7
12	1140.6048	570.8060	1123.5782	562.2928	1168.5997	584.8035	1151.5731	576.2902	V	**662.3719**	331.6896	645.3454	323.1763	6
13	1227.6368	614.3220	1210.6103	605.8088	1255.6317	628.3195	1238.6052	619.8062	S	**563.3035**	282.1554	546.2770	273.6421	5
14	1342.6637	671.8355	1325.6372	663.3222	1370.6587	685.8330	1353.6321	677.3197	D	476.2715	238.6394	459.2449	230.1261	4
15	1455.7478	728.3775	1438.7213	719.8643	1483.7427	742.3750	1466.7162	733.8617	I	361.2445	181.1259	344.2180	172.6126	3
16	1556.7955	778.9014	1539.7689	770.3881	1584.7904	792.8988	1567.7639	784.3856	T	**248.1605**	124.5839	231.1339	116.0706	2
17									K	**147.1128**	74.0600	130.0863	65.5468	1

MS/MS Fragmentation of YASEVYEKENN
Found in gi|15228407, MSD1 (MANGANESE SUPEROXIDE DISMUTASE 1); manganese superoxide dismutase

Match to Query 72: 1424.513428 from(713.263990,2+) intensity(3252.0000)
Title: 22: Sum of 3 scans in range 1403 (rt=28.2986, f=3, i=17) to 1409 (rt=28.5447, f=3, i=19)

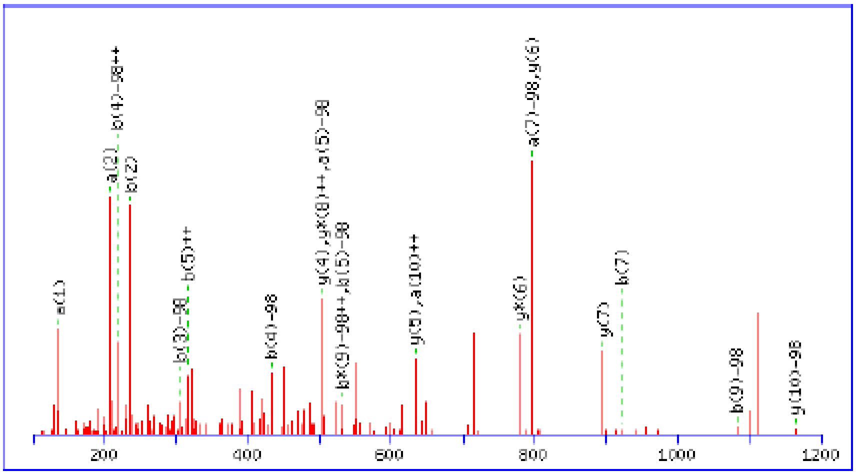

Monoisotopic mass of neutral peptide Mr(calc): 1424.5497
Fixed modifications: Carbamidomethyl (C)
Variable modifications:

S3 : Phospho (ST), with neutral losses 97.9769(shown in table), 0.0000
Ions Score: 30 **Expect:** 2.3
Matches (Bold Red): 21/144 fragment ions using 32 most intense peaks

#	a	a++	a*	a*++	b	b++	b*	b*++	Seq.	y	y++	y*	y*++	#
1	**136.0757**	68.5415			164.0706	82.5389			Y					11
2	**207.1128**	104.0600			**235.1077**	118.0575			A	**1164.5168**	582.7620	1147.4902	574.2487	10
3	276.1343	138.5708			**304.1292**	152.5682			S	1093.4796	547.2435	1076.4531	538.7302	9
4	405.1769	203.0921			**433.1718**	**217.0895**			E	1024.4582	512.7327	1007.4316	**504.2195**	8
5	**504.2453**	252.6263			**532.2402**	266.6237			V	**895.4156**	448.2114	878.3890	439.6982	7
6	667.3086	334.1579			695.3035	348.1554			Y	**796.3472**	398.6772	**779.3206**	390.1640	6
7	**796.3512**	398.6792			824.3461	412.6767			E	**633.2838**	317.1456	616.2573	308.6323	5
8	924.4462	462.7267	907.4196	454.2134	952.4411	476.7242	935.4145	468.2109	K	**504.2413**	252.6243	487.2147	244.1110	4
9	1053.4887	527.2480	1036.4622	518.7347	**1081.4837**	541.2455	1064.4571	**532.7322**	E	376.1463	188.5768	359.1197	180.0635	3
10	1167.5317	584.2695	1150.5051	575.7562	1195.5266	598.2669	1178.5000	589.7537	N	247.1037	124.0555	230.0771	115.5422	2
11									N	133.0608	67.0340	116.0342	58.5207	1

MS/MS Fragmentation of GFPGTHEFLLLDEGK
Found in gi|15228194, SBPASE (SEDOHEPTULOSE-BISPHOSPHATASE); phosphoric ester hydrolase/ sedoheptulose-bisphosphatase

Match to Query 117: 1738.908068 from(870.461310,2+) intensity(3434.0000)
Title: 117: Sum of 4 scans in range 1703 (rt=39.6156, f=4, i=125) to 1710 (rt=39.8921, f=4, i=128)

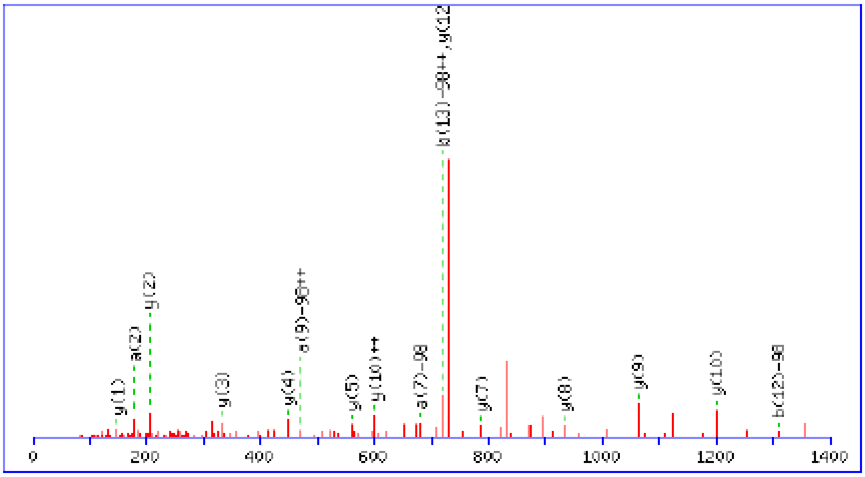

Monoisotopic mass of neutral peptide Mr(calc): 1738.7968
Fixed modifications: Carbamidomethyl (C)

Variable modifications:
T5 : Phospho (ST), with neutral losses 97.9769(shown in table), 0.0000
Ions Score: 51 **Expect:** 0.018
Matches (Bold Red): 17/168 fragment ions using 27 most intense peaks

#	a	a++	b	b++	Seq.	y	y++	y*	y*++	#
1	30.0338	15.5206	58.0287	29.5180	G					15
2	**177.1022**	89.0548	205.0972	103.0522	F	1584.8057	792.9065	1567.7791	784.3932	14
3	274.1550	137.5811	302.1499	151.5786	P	1437.7372	**719.3723**	1420.7107	710.8590	13
4	331.1765	166.0919	359.1714	180.0893	G	1340.6845	670.8459	1323.6579	662.3326	12
5	414.2136	207.6104	442.2085	221.6079	T	1283.6630	642.3351	1266.6365	633.8219	11
6	551.2725	276.1399	579.2674	290.1373	H	**1200.6259**	**600.8166**	1183.5994	592.3033	10
7	**680.3151**	340.6612	708.3100	354.6586	E	**1063.5670**	532.2871	1046.5405	523.7739	9
8	827.3835	414.1954	855.3784	428.1928	F	**934.5244**	467.7658	917.4979	459.2526	8
9	940.4676	**470.7374**	968.4625	484.7349	L	**787.4560**	394.2316	770.4294	385.7184	7
10	1053.5516	527.2794	1081.5465	541.2769	L	674.3719	337.6896	657.3454	329.1763	6
11	1166.6357	583.8215	1194.6306	597.8189	L	**561.2879**	281.1476	544.2613	272.6343	5
12	1281.6626	641.3350	**1309.6575**	655.3324	D	**448.2038**	224.6055	431.1773	216.0923	4
13	1410.7052	705.8562	1438.7001	**719.8537**	E	**333.1769**	167.0921	316.1503	158.5788	3
14	1467.7267	734.3670	1495.7216	748.3644	G	**204.1343**	102.5708	187.1077	94.0575	2
15					K	**147.1128**	74.0600	130.0863	65.5468	1

MS/MS Fragmentation of **LLSSGELYDIVGIPTSK**
Found in **gi|15229349**, ribose 5-phosphate isomerase-related [Arabidopsis thaliana]

Match to Query 119: 1870.963788 from(936.489170,2+) intensity(11798.0000)
Title: 126: Sum of 4 scans in range 1638 (rt=39.9752, f=2, i=148) to 1641 (rt=40.0771, f=2, i=151)

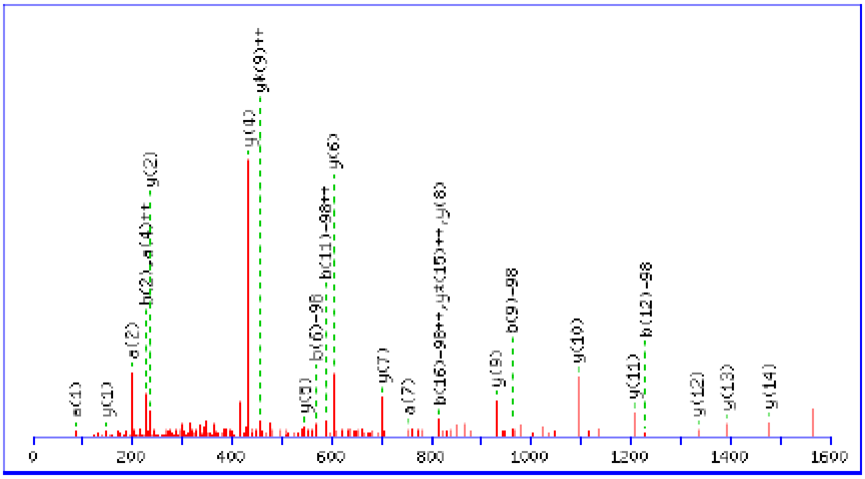

Monoisotopic mass of neutral peptide Mr(calc): 1870.9829
Fixed modifications: Carbamidomethyl (C)
Variable modifications:

S3 : Phospho (ST), with neutral losses 97.9769(shown in table), 0.0000
Ions Score: 86 **Expect:** 7.6e-06
Matches (Bold Red): 25/192 fragment ions using 40 most intense peaks

#	a	a++	b	b++	Seq.	y	y++	y*	y*++	#
1	**86.0964**	43.5519	114.0913	57.5493	L					17
2	**199.1805**	100.0939	**227.1754**	114.0913	L	1660.8792	830.9432	1643.8527	822.4300	16
3	268.2019	134.6046	296.1969	148.6021	S	1547.7952	774.4012	1530.7686	765.8879	15
4	355.2340	178.1206	383.2289	192.1181	S	**1478.7737**	739.8905	1461.7472	731.3772	14
5	412.2554	206.6314	440.2504	220.6288	G	**1391.7417**	696.3745	1374.7151	687.8612	13
6	541.2980	271.1527	**569.2929**	285.1501	E	**1334.7202**	667.8637	1317.6937	659.3505	12
7	654.3821	327.6947	682.3770	341.6921	L	**1205.6776**	603.3424	1188.6511	594.8292	11
8	817.4454	409.2263	845.4403	423.2238	Y	**1092.5936**	546.8004	1075.5670	538.2871	10
9	932.4724	466.7398	**960.4673**	480.7373	D	**929.5302**	465.2688	912.5037	**456.7555**	9
10	1045.5564	523.2819	1073.5513	537.2793	I	**814.5033**	407.7553	797.4767	399.2420	8
11	1144.6248	572.8161	1172.6198	**586.8135**	V	**701.4192**	351.2132	684.3927	342.7000	7
12	1201.6463	601.3268	**1229.6412**	615.3242	G	**602.3508**	301.6790	585.3243	293.1658	6
13	1314.7304	657.8688	1342.7253	671.8663	I	**545.3293**	273.1683	528.3028	264.6550	5
14	1411.7831	706.3952	1439.7781	720.3927	P	**432.2453**	216.6263	415.2187	208.1130	4
15	1512.8308	756.9190	1540.8257	770.9165	T	335.1925	168.0999	318.1660	159.5866	3
16	1599.8628	800.4351	1627.8578	**814.4325**	S	**234.1448**	117.5761	217.1183	109.0628	2
17					K	**147.1128**	74.0600	130.0863	65.5468	1

MS/MS Fragmentation of SLGIPLVGLDTHPR
Found in gi|15229349, ribose 5-phosphate isomerase-related [Arabidopsis thaliana]

Match to Query 104: 1553.835868 from(777.925210,2+) intensity(6694.0000)
Title: 112: Sum of 2 scans in range 1556 (rt=37.5266, f=2, i=127) to 1559 (rt=37.6494, f=2, i=128)

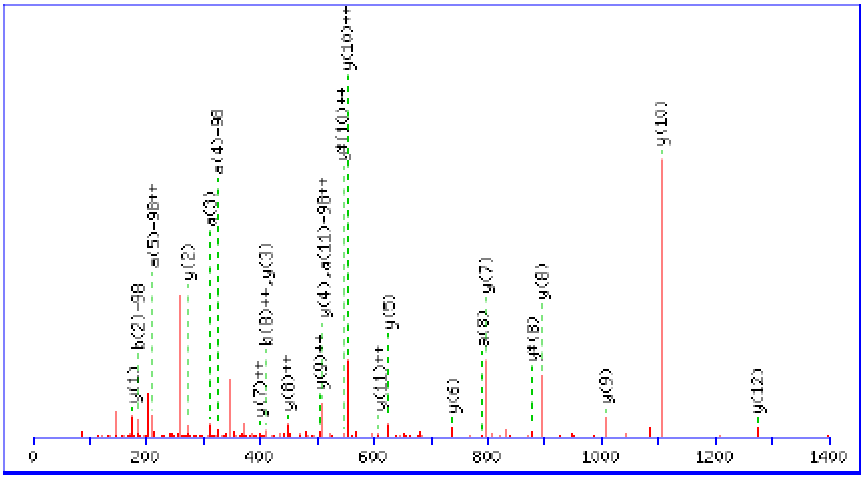

Monoisotopic mass of neutral peptide Mr(calc): 1553.7967
Fixed modifications: Carbamidomethyl (C)
Variable modifications:

S1 : Phospho (ST), with neutral losses 0.0000(shown in table), 97.9769
Ions Score: 61 **Expect:** 0.0021
Matches (Bold Red): 25/156 fragment ions using 49 most intense peaks

#	a	a^{++}	b	b^{++}	Seq.	y	y^{++}	y^{*}	y^{*++}	#
1	140.0107	70.5090	168.0056	84.5065	S					14
2	253.0948	127.0510	281.0897	141.0485	L	1387.8056	694.4064	1370.7791	685.8932	13
3	**310.1162**	155.5618	338.1112	169.5592	G	**1274.7215**	637.8644	1257.6950	629.3511	12
4	423.2003	212.1038	451.1952	226.1012	I	1217.7001	**609.3537**	1200.6735	600.8404	11
5	520.2531	260.6302	548.2480	274.6276	P	**1104.6160**	**552.8116**	1087.5895	**544.2984**	10
6	633.3371	317.1722	661.3320	331.1697	L	**1007.5633**	**504.2853**	990.5367	495.7720	9
7	732.4055	366.7064	760.4005	380.7039	V	**894.4792**	**447.7432**	**877.4526**	439.2300	8
8	**789.4270**	395.2171	817.4219	**409.2146**	G	**795.4108**	**398.2090**	778.3842	389.6958	7
9	902.5111	451.7592	930.5060	465.7566	L	**738.3893**	369.6983	721.3628	361.1850	6
10	1017.5380	509.2726	1045.5329	523.2701	D	**625.3053**	313.1563	608.2787	304.6430	5
11	1118.5857	559.7965	1146.5806	573.7939	T	**510.2783**	255.6428	493.2518	247.1295	4
12	1255.6446	628.3259	1283.6395	642.3234	H	**409.2306**	205.1190	392.2041	196.6057	3
13	1352.6974	676.8523	1380.6923	690.8498	P	**272.1717**	136.5895	255.1452	128.0762	2
14					R	**175.1190**	88.0631	158.0924	79.5498	1

63c

MS/MS Fragmentation of LQDLFKEFGCESK
Found in gi|15229349, ribose 5-phosphate isomerase-related [Arabidopsis thaliana]

Match to Query 110: 1679.760028 from(840.887290,2+) intensity(6820.0000)
Title: 101: Sum of 2 scans in range 1507 (rt=35.9358, f=2, i=112) to 1509 (rt=36.0177, f=2, i=113)

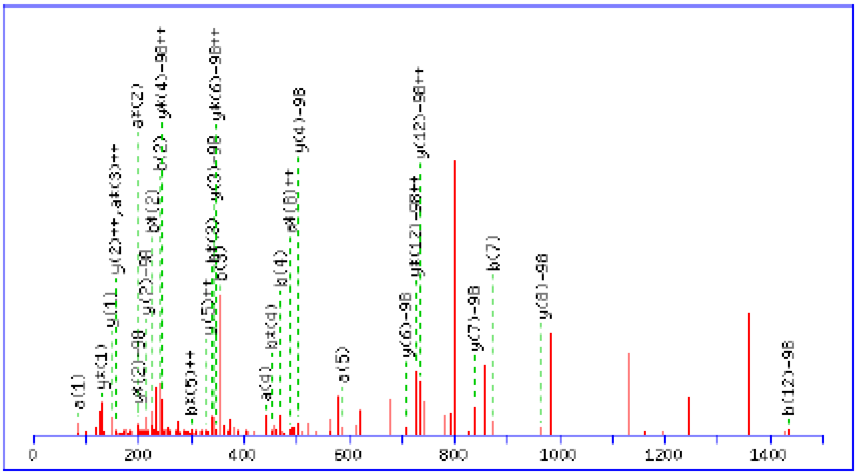

Monoisotopic mass of neutral peptide Mr(calc): 1679.7266
Fixed modifications: Carbamidomethyl (C)
Variable modifications:

S12 : Phospho (ST), with neutral losses 97.9769(shown in table), 0.0000
Ions Score: 27 **Expect:** 5.2
Matches (Bold Red): 30/192 fragment ions using 66 most intense peaks

#	a	a++	a*	a*++	b	b++	b*	b*++	Seq.	y	y++	y*	y*++	#
1	**86.0964**	43.5519			114.0913	57.5493			L					13
2	214.1550	107.5811	**197.1285**	99.0679	**242.1499**	121.5786	**225.1234**	113.0653	Q	1469.6729	**735.3401**	1452.6464	**726.8268**	12
3	329.1819	165.0946	312.1554	**156.5813**	**357.1769**	179.0921	**340.1503**	170.5788	D	1341.6144	671.3108	1324.5878	662.7975	11
4	**442.2660**	221.6366	425.2395	213.1234	**470.2609**	235.6341	**453.2344**	227.1208	L	1226.5874	613.7973	1209.5609	605.2841	10
5	**589.3344**	295.1709	572.3079	286.6576	617.3293	309.1683	600.3028	**300.6550**	F	1113.5034	557.2553	1096.4768	548.7420	9
6	717.4294	359.2183	700.4028	350.7051	745.4243	373.2158	728.3978	364.7025	K	**966.4349**	483.7211	949.4084	475.2078	8
7	846.4720	423.7396	829.4454	415.2264	**874.4669**	437.7371	857.4403	429.2238	E	**838.3400**	419.6736	821.3134	411.1603	7
8	993.5404	497.2738	976.5138	**488.7606**	1021.5353	511.2713	1004.5088	502.7580	F	**709.2974**	355.1523	692.2708	**346.6391**	6
9	1050.5619	525.7846	1033.5353	517.2713	1078.5568	539.7820	1061.5302	531.2688	G	562.2290	281.6181	545.2024	273.1048	5
10	1210.5925	605.7999	1193.5660	597.2866	1238.5874	619.7973	1221.5609	611.2841	C	**505.2075**	253.1074	488.1810	**244.5941**	4
11	1339.6351	670.3212	1322.6086	661.8079	1367.6300	684.3186	1350.6035	675.8054	E	**345.1769**	173.0921	328.1503	164.5788	3
12	1408.6566	704.8319	1391.6300	696.3186	**1436.6515**	718.8294	1419.6249	710.3161	S	**216.1343**	108.5708	**199.1077**	100.0575	2
13									K	**147.1128**	74.0600	**130.0863**	65.5468	1

MS/MS Fragmentation of AILNESSEFVGDKVAYALAQGLK
Found in gi|414550, cytosolic triose phosphate isomerase

Match to Query 152: 2502.321072 from(835.114300,3+) intensity(9789.0000)
Title: 145: Sum of 4 scans in range 1680 (rt=41.6594, f=2, i=175) to 1683 (rt=41.7626, f=2, i=178)

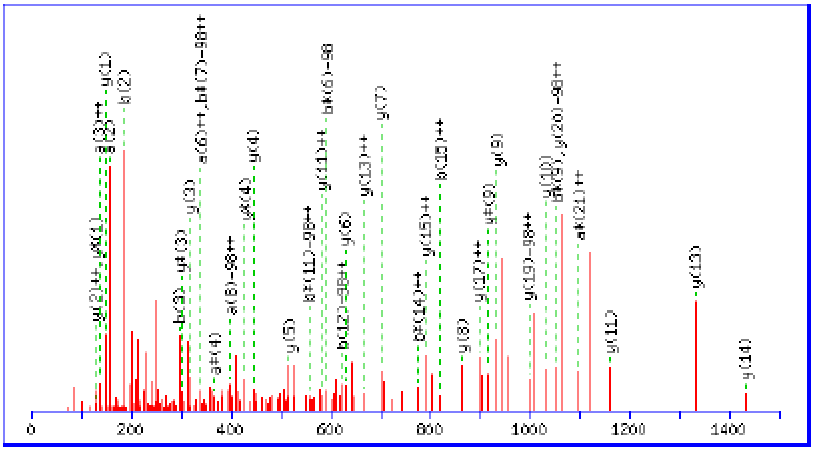

Monoisotopic mass of neutral peptide Mr(calc): 2502.2407
Fixed modifications: Carbamidomethyl (C)
Variable modifications:
S6 : Phospho (ST), with neutral losses 97.9769(shown in table), 0.0000
Ions Score: 41 **Expect:** 0.22
Matches (Bold Red): 38/408 fragment ions using 80 most intense peaks

#	a	a^{++}	a^{*}	a^{*++}	b	b^{++}	b^{*}	b^{*++}	Seq.	y	y^{++}	y^{*}	y^{*++}	#
1	44.0495	22.5284			72.0444	36.5258			A					23
2	**157.1335**	79.0704			**185.1285**	93.0679			I	2334.2340	1167.6206	2317.2074	1159.1074	22
3	270.2176	**135.6124**			**298.2125**	149.6099			L	2221.1499	1111.0786	2204.1234	1102.5653	21
4	384.2605	192.6339	**367.2340**	184.1206	412.2554	206.6314	395.2289	198.1181	N	2108.0659	**1054.5366**	2091.0393	1046.0233	20
5	513.3031	257.1552	496.2766	248.6419	541.2980	271.1527	524.2715	262.6394	E	1994.0229	**997.5151**	1976.9964	989.0018	19
6	582.3246	291.6659	565.2980	283.1527	610.3195	305.6634	**593.2929**	297.1501	S	1864.9803	932.9938	1847.9538	924.4805	18
7	669.3566	335.1819	652.3301	326.6687	697.3515	349.1794	680.3250	**340.6661**	S	1795.9589	**898.4831**	1778.9323	889.9698	17
8	798.3992	**399.7032**	781.3727	391.1900	826.3941	413.7007	809.3676	405.1874	E	1708.9269	854.9671	1691.9003	846.4538	16
9	945.4676	473.2374	928.4411	464.7242	973.4625	487.2349	956.4360	478.7216	F	1579.8843	**790.4458**	1562.8577	781.9325	15
10	1044.5360	522.7717	1027.5095	514.2584	1072.5309	536.7691	1055.5044	528.2558	V	**1432.8158**	716.9116	1415.7893	708.3983	14
11	1101.5575	551.2824	1084.5309	542.7691	1129.5524	565.2798	1112.5259	**556.7666**	G	**1333.7474**	**667.3774**	1316.7209	658.8641	13
12	1216.5844	608.7959	1199.5579	600.2826	1244.5794	**622.7933**	1227.5528	614.2800	D	1276.7260	638.8666	1259.6994	630.3533	12
13	1344.6794	672.8433	1327.6528	664.3301	1372.6743	686.8408	1355.6478	678.3275	K	**1161.6990**	**581.3531**	1144.6725	572.8399	11
14	1443.7478	722.3775	1426.7213	713.8643	1471.7427	736.3750	1454.7162	727.8617	V	**1033.6041**	517.3057	1016.5775	508.7924	10
15	1514.7849	757.8961	1497.7584	749.3828	1542.7798	771.8936	1525.7533	763.3803	A	**934.5356**	467.7715	**917.5091**	459.2582	9
16	1677.8483	839.4278	1660.8217	830.9145	1705.8432	853.4252	1688.8166	844.9119	Y	**863.4985**	432.2529	846.4720	423.7396	8
17	1748.8854	874.9463	1731.8588	866.4330	1776.8803	888.9438	1759.8537	880.4305	A	**700.4352**	350.7212	683.4087	342.2080	7
18	1861.9694	931.4884	1844.9429	922.9751	1889.9643	945.4858	1872.9378	936.9725	L	**629.3981**	315.2027	612.3715	306.6894	6
19	1933.0065	967.0069	1915.9800	958.4936	1961.0015	981.0044	1943.9749	972.4911	A	**516.3140**	258.6607	499.2875	250.1474	5
20	2061.0651	1031.0362	2044.0386	1022.5229	2089.0600	1045.0337	2072.0335	1036.5204	Q	**445.2769**	223.1421	**428.2504**	214.6288	4
21	2118.0866	1059.5469	2101.0600	1051.0337	2146.0815	1073.5444	2129.0550	1065.0311	G	**317.2183**	159.1128	**300.1918**	150.5995	3
22	2231.1707	1116.0890	2214.1441	1107.5757	2259.1656	1130.0864	2242.1390	1121.5731	L	260.1969	**130.6021**	243.1703	122.0888	2
23									K	**147.1128**	74.0600	**130.0863**	65.5468	1

MS/MS Fragmentation of VASPAQAQEVHDELRK
Found in gi|414550, cytosolic triose phosphate isomerase

Match to Query 131: 1856.922762 from(619.981530,3+) intensity(9856.0000)
Title: 41: Sum of 2 scans in range 1202 (rt=25.7284, f=3, i=40) to 1205 (rt=25.8514, f=3, i=41)

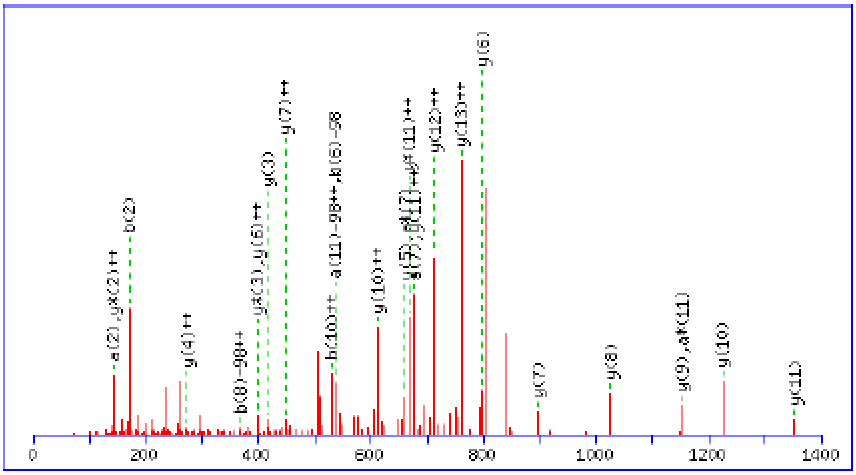

Monoisotopic mass of neutral peptide Mr(calc): 1856.8782
Fixed modifications: Carbamidomethyl (C)
Variable modifications:

S3 : Phospho (ST), with neutral losses 97.9769(shown in table), 0.0000
Ions Score: 58 **Expect:** 0.0054
Matches (**Bold Red**): 27/260 fragment ions using 33 most intense peaks

#	a	a++	a*	a*++	b	b++	b*	b*++	Seq.	y	y++	y*	y*++	#
1	72.0808	36.5440			100.0757	50.5415			V					16
2	**143.1179**	72.0626			**171.1128**	86.0600			A	1660.8402	830.9237	1643.8136	822.4104	15
3	212.1393	106.5733			240.1343	120.5708			S	1589.8030	795.4052	1572.7765	786.8919	14
4	309.1921	155.0997			337.1870	169.0971			P	1520.7816	**760.8944**	1503.7550	752.3812	13
5	380.2292	190.6182			408.2241	204.6157			A	1423.7288	**712.3680**	1406.7023	703.8548	12
6	508.2878	254.6475	491.2613	246.1343	**536.2827**	268.6450	519.2562	260.1317	Q	**1352.6917**	**676.8495**	1335.6652	**668.3362**	11
7	579.3249	290.1661	562.2984	281.6528	607.3198	304.1636	590.2933	295.6503	A	**1224.6331**	**612.8202**	1207.6066	604.3069	10
8	707.3835	354.1954	690.3569	345.6821	735.3784	**368.1928**	718.3519	359.6796	Q	**1153.5960**	577.3016	1136.5695	568.7884	9
9	836.4261	418.7167	819.3995	410.2034	864.4210	432.7141	847.3945	424.2009	E	**1025.5374**	513.2724	1008.5109	504.7591	8
10	935.4945	468.2509	918.4680	459.7376	963.4894	482.2483	946.4629	473.7351	V	**896.4948**	**448.7511**	879.4683	440.2378	7
11	1072.5534	**536.7803**	1055.5269	528.2671	1100.5483	550.7778	1083.5218	542.2645	H	**797.4264**	**399.2169**	780.3999	390.7036	6
12	1187.5804	594.2938	1170.5538	585.7805	1215.5753	608.2913	1198.5487	599.7780	D	**660.3675**	330.6874	643.3410	322.1741	5
13	1316.6229	658.8151	1299.5964	650.3018	1344.6179	672.8126	1327.5913	664.2993	E	545.3406	**273.1739**	528.3140	264.6606	4
14	1429.7070	715.3571	1412.6805	706.8439	1457.7019	729.3546	1440.6754	720.8413	L	**416.2980**	208.6526	**399.2714**	200.1394	3
15	1585.8081	793.4077	1568.7816	784.8944	1613.8030	807.4052	1596.7765	798.8919	R	303.2139	152.1106	286.1874	**143.5973**	2
16									K	147.1128	74.0600	130.0863	65.5468	1

MS/MS Fragmentation of EHGNSPGYYDGR
Found in gi|13926229, F1O19.10/F1O19.10 [Arabidopsis thaliana]

Match to Query 99: 1430.577208 from(716.295880,2+) intensity(25092.0000)
Title: 14: Sum of 7 scans in range 1196 (rt=23.3959, f=2, i=33) to 1205 (rt=23.7597, f=4, i=20)

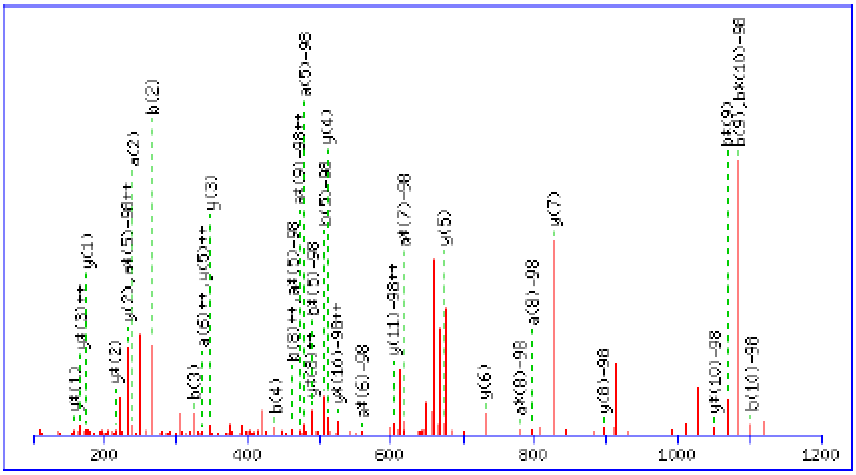

Monoisotopic mass of neutral peptide Mr(calc): 1430.5252
Fixed modifications: Carbamidomethyl (C)
Variable modifications:

S5 : Phospho (ST), with neutral losses 97.9769(shown in table), 0.0000
Ions Score: 37 **Expect:** 0.45
Matches (Bold Red): 36/192 fragment ions using 67 most intense peaks

#	a	a^{++}	a*	a^{*++}	b	b^{++}	b*	b^{*++}	Seq.	y	y^{++}	y*	y^{*++}	#
1	102.0550	51.5311			130.0499	65.5286			E					12
2	**239.1139**	120.0606			**267.1088**	134.0580			H	1204.5130	**602.7601**	1187.4865	594.2469	11
3	296.1353	148.5713			**324.1302**	162.5688			G	1067.4541	534.2307	**1050.4275**	**525.7174**	10
4	410.1783	205.5928	393.1517	197.0795	**438.1732**	219.5902	421.1466	211.0769	N	1010.4326	505.7200	993.4061	497.2067	9
5	**479.1997**	240.1035	**462.1732**	**231.5902**	**507.1946**	254.1010	**490.1681**	245.5877	S	**896.3897**	448.6985	879.3632	440.1852	8
6	576.2525	288.6299	**559.2259**	280.1166	604.2474	302.6273	587.2208	294.1141	P	**827.3682**	414.1878	810.3417	405.6745	7
7	633.2739	317.1406	**616.2474**	308.6273	661.2689	331.1381	644.2423	322.6248	G	**730.3155**	365.6614	713.2889	357.1481	6
8	**796.3373**	398.6723	**779.3107**	390.1590	824.3322	412.6697	807.3056	404.1565	Y	**673.2940**	**337.1506**	656.2675	328.6374	5
9	959.4006	480.2039	942.3741	**471.6907**	987.3955	494.2014	970.3690	485.6881	Y	**510.2307**	255.6190	493.2041	247.1057	4
10	1074.4275	537.7174	1057.4010	529.2041	**1102.4225**	551.7149	**1085.3959**	543.2016	D	**347.1674**	174.0873	330.1408	**165.5740**	3
11	1131.4490	566.2281	1114.4225	557.7149	1159.4439	580.2256	1142.4174	571.7123	G	**232.1404**	116.5738	**215.1139**	108.0606	2
12									R	**175.1190**	88.0631	**158.0924**	79.5498	1

65b

MS/MS Fragmentation of **KFETLSYLPDLTDSELAK**
Found in gi|13926229, F1O19.10/F1O19.10 [Arabidopsis thaliana]

Match to Query 178: 2149.108048 from(1075.561300,2+) intensity(6746.0000)

Title: 130: Scan 1643 (rt=38.8713, f=3, i=113)

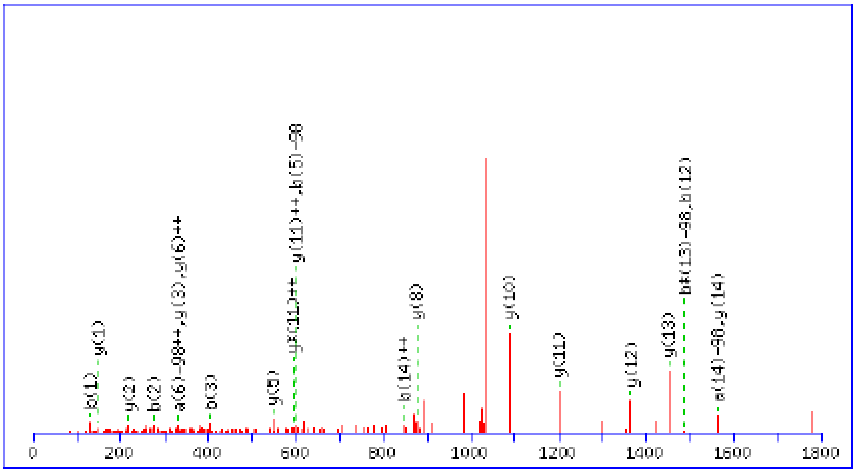


```
Monoisotopic mass of neutral peptide Mr(calc): 2149.0231
Fixed modifications: Carbamidomethyl (C)
Variable modifications:
T4      : Phospho (ST), with neutral losses 97.9769(shown in table), 0.0000
Ions Score: 56   Expect: 0.0067
```

Matches (**Bold Red**): 22/328 fragment ions using 34 most intense peaks

#	a	a++	a*	a*++	b	b++	b*	b*++	Seq.	y	y++	y*	y*++	#
1	101.1073	51.0573	84.0808	42.5440	**129.1022**	65.0548	112.0757	56.5415	K					18
2	248.1757	124.5915	231.1492	116.0782	**276.1707**	138.5890	259.1441	130.0757	F	1923.9586	962.4829	1906.9320	953.9697	17
3	377.2183	189.1128	360.1918	180.5995	**405.2132**	203.1103	388.1867	194.5970	E	1776.8902	888.9487	1759.8636	880.4355	16
4	460.2554	230.6314	443.2289	222.1181	488.2504	244.6288	471.2238	236.1155	T	1647.8476	824.4274	1630.8210	815.9142	15
5	573.3395	287.1734	556.3130	278.6601	**601.3344**	301.1708	584.3079	292.6576	L	**1564.8105**	782.9089	1547.7839	774.3956	14
6	660.3715	**330.6894**	643.3450	322.1761	688.3664	344.6869	671.3399	336.1736	S	**1451.7264**	726.3668	1434.6999	717.8536	13
7	823.4349	412.2211	806.4083	403.7078	851.4298	426.2185	834.4032	417.7053	Y	**1364.6944**	682.8508	1347.6678	674.3376	12
8	936.5189	468.7631	919.4924	460.2498	964.5138	482.7606	947.4873	474.2473	L	**1201.6311**	**601.3192**	1184.6045	**592.8059**	11
9	1033.5717	517.2895	1016.5451	508.7762	1061.5666	531.2869	1044.5401	522.7737	P	**1088.5470**	544.7771	1071.5204	536.2639	10
10	1148.5986	574.8030	1131.5721	566.2897	1176.5935	588.8004	1159.5670	580.2871	D	991.4942	496.2508	974.4677	487.7375	9
11	1261.6827	631.3450	1244.6561	622.8317	1289.6776	645.3424	1272.6511	636.8292	L	**876.4673**	438.7373	859.4407	430.2240	8
12	1362.7304	681.8688	1345.7038	673.3556	1390.7253	695.8663	1373.6987	687.3530	T	763.3832	382.1953	746.3567	373.6820	7
13	1477.7573	739.3823	1460.7308	730.8690	1505.7522	753.3798	**1488.7257**	744.8665	D	662.3355	**331.6714**	645.3090	323.1581	6
14	**1564.7893**	782.8983	1547.7628	774.3850	1592.7843	796.8958	1575.7577	788.3825	S	**547.3086**	274.1579	530.2821	265.6447	5
15	1693.8319	847.4196	1676.8054	838.9063	1721.8269	861.4171	1704.8003	852.9038	E	460.2766	230.6419	443.2500	222.1287	4
16	1806.9160	903.9616	1789.8895	895.4484	1834.9109	917.9591	1817.8844	909.4458	L	**331.2340**	166.1206	314.2074	157.6074	3
17	1877.9531	939.4802	1860.9266	930.9669	1905.9480	953.4777	1888.9215	944.9644	A	**218.1499**	109.5786	201.1234	101.0653	2
18									K	**147.1128**	74.0600	130.0863	65.5468	1

MS/MS Fragmentation of EHGNTPGYYDGR
Found in gi|16194, ribulose bisphosphate carboxylase [Arabidopsis thaliana]

Match to Query 90: 1444.535708 from(723.275130,2+) intensity(3005.0000)
Title: 19: Sum of 3 scans in range 1114 (rt=22.4968, f=2, i=47) to 1120 (rt=22.7429, f=2, i=49)

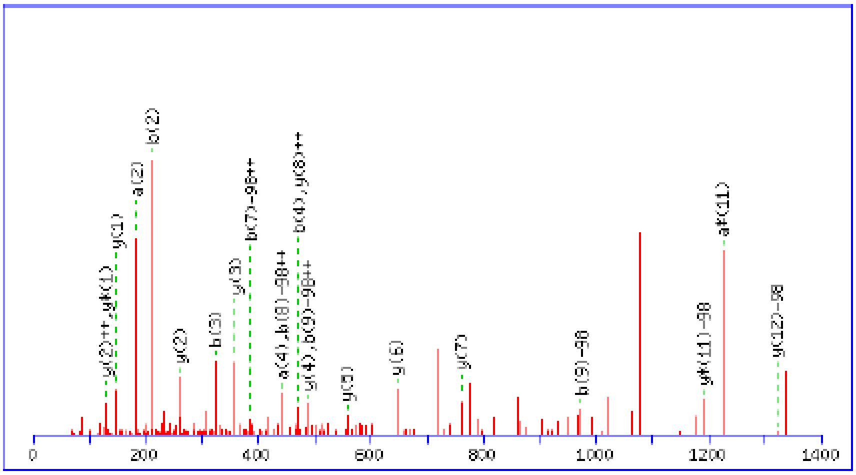

Monoisotopic mass of neutral peptide Mr(calc): 1627.7681
Fixed modifications: Carbamidomethyl (C)
Variable modifications:

T7 : Phospho (ST), with neutral losses 97.9769(shown in table), 0.0000
Ions Score: 37 **Expect:** 0.69
Matches (Bold Red)**:** 22/180 fragment ions using 37 most intense peaks

#	a	a++	a*	a*++	b	b++	b*	b*++	Seq.	y	y++	y*	y*++	#
1	86.0964	43.5519			114.0913	57.5493			L					14
2	**183.1492**	92.0782			**211.1441**	106.0757			P	1417.7144	709.3608	1400.6879	700.8476	13
3	296.2333	148.6203			**324.2282**	162.6177			L	**1320.6616**	660.8345	1303.6351	652.3212	12
4	**443.3017**	222.1545			**471.2966**	236.1519			F	1207.5776	604.2924	**1190.5510**	595.7792	11
5	500.3231	250.6652			528.3180	264.6627			G	1060.5092	530.7582	1043.4826	522.2449	10
6	660.3538	330.6805			688.3487	344.6780			C	1003.4877	502.2475	986.4612	493.7342	9
7	743.3909	372.1991			771.3858	**386.1965**			T	843.4571	422.2322	826.4305	413.7189	8
8	858.4178	429.7126			886.4127	**443.7100**			D	**760.4199**	380.7136	743.3934	372.2003	7
9	945.4499	473.2286			**973.4448**	**487.2260**			S	**645.3930**	323.2001	628.3665	314.6869	6
10	1016.4870	508.7471			1044.4819	522.7446			A	**558.3610**	279.6841	541.3344	271.1709	5
11	1144.5456	572.7764	1127.5190	564.2631	1172.5405	586.7739	1155.5139	578.2606	Q	**487.3239**	244.1656	470.2973	235.6523	4
12	1243.6140	622.3106	1226.5874	613.7973	1271.6089	636.3081	1254.5823	627.7948	V	**359.2653**	180.1363	342.2387	171.6230	3
13	1356.6980	678.8527	1339.6715	670.3394	1384.6929	692.8501	1367.6664	684.3368	L	**260.1969**	**130.6021**	243.1703	122.0888	2
14									K	**147.1128**	74.0600	**130.0863**	65.5468	1

66b

MS/MS Fragmentation of KFETLSYLPDLSDVELAK
Found in gi|16194, ribulose bisphosphate carboxylase [Arabidopsis thaliana]

Match to Query 111: 2147.131848 from(1074.573200,2+) intensity(6845.0000)
Title: 95: Sum of 2 scans in range 1748 (rt=40.0075, f=2, i=185) to 1751 (rt=40.135, f=2, i=186)

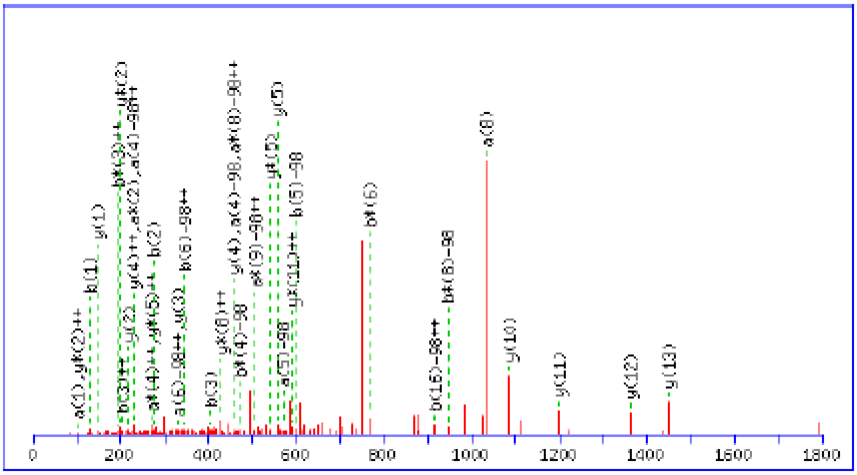

Monoisotopic mass of neutral peptide Mr(calc): 2147.0439
Fixed modifications: Carbamidomethyl (C)
Variable modifications:

T4 : Phospho (ST), with neutral losses 97.9769(shown in table), 0.0000
Ions Score: 26 **Expect:** 8.1
Matches (Bold Red): 37/328 fragment ions using 80 most intense peaks

#	a	a++	a*	a*++	b	b++	b*	b*++	Seq.	y	y++	y*	y*++	#
1	**101.1073**	51.0573	84.0808	42.5440	**129.1022**	65.0548	112.0757	56.5415	K					18
2	248.1757	124.5915	**231.1492**	116.0782	**276.1707**	138.5890	259.1441	130.0757	F	1921.9793	961.4933	1904.9528	952.9800	17
3	377.2183	189.1128	360.1918	180.5995	**405.2132**	**203.1103**	388.1867	**194.5970**	E	1774.9109	887.9591	1757.8844	879.4458	16
4	**460.2554**	**230.6314**	443.2289	222.1181	488.2504	244.6288	**471.2238**	236.1155	T	1645.8683	823.4378	1628.8418	814.9245	15
5	**573.3395**	287.1734	556.3130	278.6601	**601.3344**	301.1708	584.3079	292.6576	L	1562.8312	781.9192	1545.8047	773.4060	14
6	660.3715	**330.6894**	643.3450	322.1761	688.3664	**344.6869**	671.3399	336.1736	S	**1449.7472**	725.3772	1432.7206	716.8639	13
7	823.4349	412.2211	806.4083	403.7078	851.4298	426.2185	834.4032	417.7053	Y	**1362.7151**	681.8612	1345.6886	673.3479	12
8	936.5189	468.7631	919.4924	**460.2498**	964.5138	482.7606	**947.4873**	474.2473	L	**1199.6518**	600.3295	1182.6252	**591.8163**	11
9	1033.5717	517.2895	1016.5451	**508.7762**	1061.5666	531.2869	1044.5401	522.7737	P	**1086.5677**	543.7875	1069.5412	535.2742	10
10	1148.5986	574.8030	1131.5721	566.2897	1176.5935	588.8004	1159.5670	580.2871	D	989.5150	495.2611	972.4884	486.7478	9
11	1261.6827	631.3450	1244.6561	622.8317	1289.6776	645.3424	1272.6511	636.8292	L	874.4880	437.7477	857.4615	**429.2344**	8
12	1348.7147	674.8610	1331.6882	666.3477	1376.7096	688.8585	1359.6831	680.3452	S	761.4040	381.2056	744.3774	372.6923	7
13	1463.7417	732.3745	1446.7151	723.8612	1491.7366	746.3719	1474.7100	737.8587	D	674.3719	337.6896	657.3454	329.1763	6
14	1562.8101	781.9087	1545.7835	773.3954	1590.8050	795.9061	1573.7784	787.3929	V	**559.3450**	280.1761	**542.3184**	**271.6629**	5
15	1691.8527	846.4300	1674.8261	837.9167	1719.8476	860.4274	1702.8210	851.9142	E	**460.2766**	**230.6419**	443.2500	222.1287	4
16	1804.9367	902.9720	1787.9102	894.4587	1832.9317	**916.9695**	1815.9051	908.4562	L	**331.2340**	166.1206	314.2074	157.6074	3
17	1875.9739	938.4906	1858.9473	929.9773	1903.9688	952.4880	1886.9422	943.9747	A	**218.1499**	109.5786	**201.1234**	**101.0653**	2
18									K	**147.1128**	74.0600	130.0863	65.5468	1

MS/MS Fragmentation of FETLSYLPDLSDVELAK
Found in gi|16194, ribulose bisphosphate carboxylase [Arabidopsis thaliana]

Match to Query 125: 2018.958248 from(1010.486400,2+) intensity(5987.0000)
Title: 140: Sum of 4 scans in range 1671 (rt=42.1263, f=2, i=201) to 1674 (rt=42.2289, f=2, i=204)

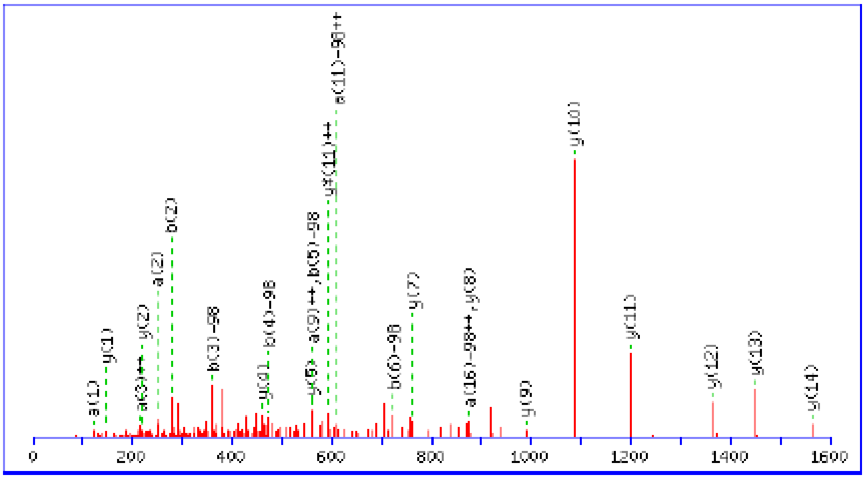

Monoisotopic mass of neutral peptide Mr(calc): 2018.9489
Fixed modifications: Carbamidomethyl (C)
Variable modifications:

T3 : Phospho (ST), with neutral losses 97.9769(shown in table), 0.0000
Ions Score: 70 **Expect:** 0.00024
Matches (Bold Red): 24/192 fragment ions using 36 most intense peaks

#	a	a++	b	b++	Seq.	y	y++	y*	y*++	#
1	**120.0808**	60.5440	148.0757	74.5415	F					17
2	**249.1234**	125.0653	**277.1183**	139.0628	E	1774.9109	887.9591	1757.8844	879.4458	16
3	332.1605	166.5839	**360.1554**	180.5813	T	1645.8683	823.4378	1628.8418	814.9245	15
4	445.2445	223.1259	**473.2395**	237.1234	L	**1562.8312**	781.9192	1545.8047	773.4060	14
5	532.2766	266.6419	**560.2715**	280.6394	S	**1449.7472**	725.3772	1432.7206	716.8639	13
6	695.3399	348.1736	**723.3348**	362.1710	Y	**1362.7151**	681.8612	1345.6886	673.3479	12
7	808.4240	404.7156	836.4189	418.7131	L	**1199.6518**	600.3295	1182.6252	**591.8163**	11
8	905.4767	453.2420	933.4716	467.2395	P	**1086.5677**	543.7875	1069.5412	535.2742	10
9	1020.5037	510.7555	1048.4986	524.7529	D	**989.5150**	495.2611	972.4884	486.7478	9
10	1133.5877	567.2975	1161.5826	581.2950	L	**874.4880**	437.7477	857.4615	429.2344	8
11	1220.6198	**610.8135**	1248.6147	624.8110	S	**761.4040**	381.2056	744.3774	372.6923	7
12	1335.6467	668.3270	1363.6416	682.3244	D	674.3719	337.6896	657.3454	329.1763	6
13	1434.7151	717.8612	1462.7100	731.8587	V	**559.3450**	280.1761	542.3184	271.6629	5
14	1563.7577	782.3825	1591.7526	796.3800	E	**460.2766**	230.6419	443.2500	222.1287	4
15	1676.8418	838.9245	1704.8367	852.9220	L	331.2340	166.1206	314.2074	157.6074	3
16	1747.8789	**874.4431**	1775.8738	888.4405	A	**218.1499**	109.5786	201.1234	101.0653	2
17					K	**147.1128**	74.0600	130.0863	65.5468	1

MS/MS Fragmentation of **LPLFGCTDSAQVLK**
Found in **gi|16194**, ribulose bisphosphate carboxylase [Arabidopsis thaliana]

Match to Query 70: 1627.830468 from(814.922510,2+) intensity(15707.0000)
Title: 75: Sum of 6 scans in range 1729 (rt=38.62, f=2, i=186) to 1737 (rt=38.9435, f=4, i=47)

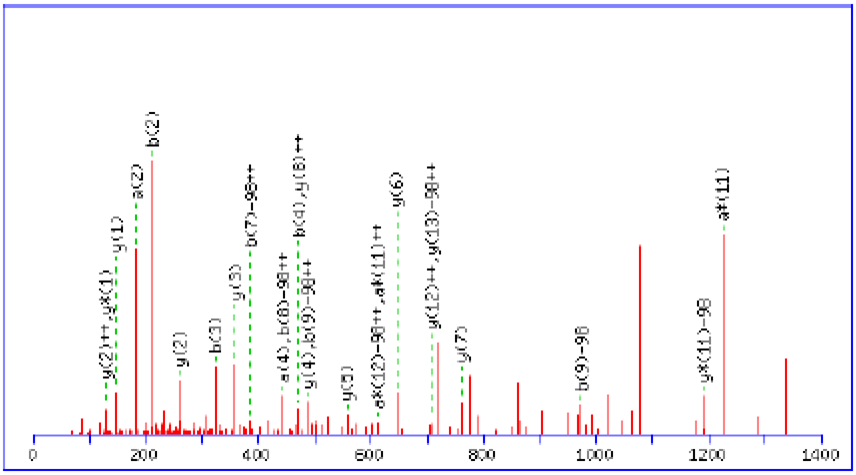

Monoisotopic mass of neutral peptide Mr(calc): 1627.7681
Fixed modifications: Carbamidomethyl (C)
Variable modifications:

T7 : Phospho (ST), with neutral losses 97.9769(shown in table), 0.0000
Ions Score: 30 **Expect:** 3.3
Matches (**Bold Red**): 25/180 fragment ions using 36 most intense peaks

#	a	a++	a*	a*++	b	b++	b*	b*++	Seq.	y	y++	y*	y*++	#
1	86.0964	43.5519			114.0913	57.5493			L					14
2	**183.1492**	92.0782			**211.1441**	106.0757			P	1417.7144	**709.3608**	1400.6879	700.8476	13
3	296.2333	148.6203			**324.2282**	162.6177			L	1320.6616	660.8345	1303.6351	652.3212	12
4	**443.3017**	222.1545			**471.2966**	236.1519			F	1207.5776	604.2924	**1190.5510**	595.7792	11
5	500.3231	250.6652			528.3180	264.6627			G	1060.5092	530.7582	1043.4826	522.2449	10
6	660.3538	330.6805			688.3487	344.6780			C	1003.4877	502.2475	986.4612	493.7342	9
7	743.3909	372.1991			771.3858	**386.1965**			T	843.4571	422.2322	826.4305	413.7189	8
8	858.4178	429.7126			886.4127	**443.7100**			D	**760.4199**	380.7136	743.3934	372.2003	7
9	945.4499	473.2286			**973.4448**	**487.2260**			S	**645.3930**	323.2001	628.3665	314.6869	6
10	1016.4870	508.7471			1044.4819	522.7446			A	**558.3610**	279.6841	541.3344	271.1709	5
11	1144.5456	572.7764	1127.5190	564.2631	1172.5405	586.7739	1155.5139	578.2606	Q	**487.3239**	244.1656	470.2973	235.6523	4
12	1243.6140	622.3106	1226.5874	**613.7973**	1271.6089	636.3081	1254.5823	627.7948	V	**359.2653**	180.1363	342.2387	171.6230	3
13	1356.6980	678.8527	1339.6715	670.3394	1384.6929	692.8501	1367.6664	684.3368	L	**260.1969**	**130.6021**	243.1703	122.0888	2
14									K	**147.1128**	74.0600	**130.0863**	65.5468	1

MS/MS Fragmentation of VKPSSPAELEALMGPK
Found in gi|15223217, glycine cleavage system H protein, mitochondrial, putative [Arabidopsis thaliana]

Match to Query 107: 1732.928308 from(867.471430,2+) intensity(12872.0000)
Title: 81: Sum of 7 scans in range 1489 (rt=34.4271, f=2, i=136) to 1498 (rt=34.7892, f=4, i=76)

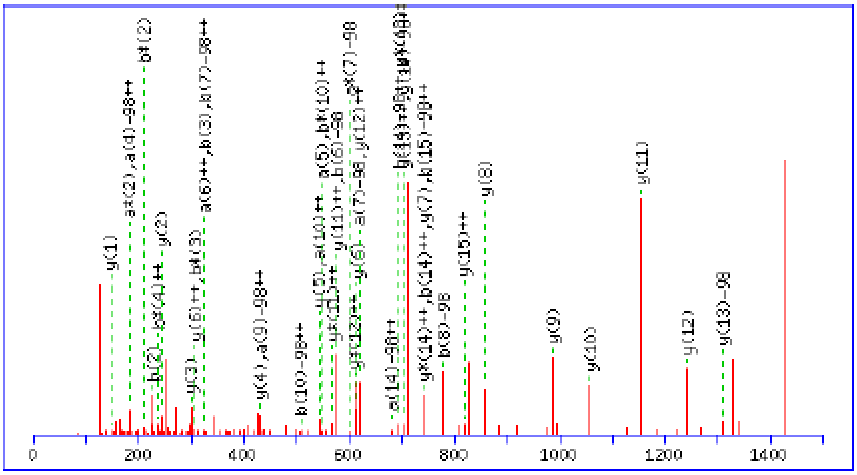

Monoisotopic mass of neutral peptide Mr(calc): 1732.8470
Fixed modifications: Carbamidomethyl (C)
Variable modifications:

S4 : Phospho (ST), with neutral losses 97.9769(shown in table), 0.0000
Ions Score: 74 **Expect:** 9.9e-05
Matches (Bold Red): 46/284 fragment ions using 65 most intense peaks

#	a	a++	a*	a*++	b	b++	b*	b*++	Seq.	y	y++	y*	y*++	#
1	72.0808	36.5440			100.0757	50.5415			V					16
2	200.1757	100.5915	**183.1492**	92.0782	**228.1707**	114.5890	**211.1441**	106.0757	K	1536.8090	768.9082	1519.7825	760.3949	15
3	297.2285	149.1179	280.2020	140.6046	**325.2234**	163.1153	**308.1969**	154.6021	P	1408.7141	**704.8607**	1391.6875	**696.3474**	14
4	366.2500	**183.6286**	349.2234	175.1153	394.2449	197.6261	377.2183	189.1128	S	**1311.6613**	656.3343	1294.6348	647.8210	13
5	453.2820	227.1446	436.2554	218.6314	481.2769	241.1421	464.2504	232.6288	S	**1242.6399**	**621.8236**	1225.6133	**613.3103**	12
6	550.3347	275.6710	533.3082	267.1577	**578.3297**	289.6685	561.3031	281.1552	P	**1155.6078**	**578.3075**	1138.5813	**569.7943**	11
7	**621.3719**	311.1896	**604.3453**	302.6763	649.3668	**325.1870**	632.3402	316.6738	A	**1058.5551**	529.7812	1041.5285	521.2679	10
8	750.4145	375.7109	733.3879	367.1976	**778.4094**	389.7083	761.3828	381.1950	E	**987.5179**	494.2626	970.4914	485.7493	9
9	863.4985	**432.2529**	846.4720	423.7396	891.4934	446.2504	874.4669	437.7371	L	**858.4754**	429.7413	841.4488	421.2280	8
10	992.5411	496.7742	975.5146	488.2609	1020.5360	**510.7717**	1003.5095	502.2584	E	**745.3913**	373.1993	728.3647	364.6860	7
11	1063.5782	532.2928	1046.5517	523.7795	1091.5731	546.2902	1074.5466	537.7769	A	**616.3487**	**308.6780**	599.3221	300.1647	6
12	1176.6623	588.8348	1159.6357	580.3215	1204.6572	602.8322	1187.6307	594.3190	L	**545.3116**	273.1594	528.2850	264.6462	5
13	1307.7028	654.3550	1290.6762	645.8418	1335.6977	668.3525	1318.6711	659.8392	M	**432.2275**	216.6174	415.2010	208.1041	4
14	1364.7242	**682.8658**	1347.6977	674.3525	1392.7192	**696.8632**	1375.6926	688.3499	G	**301.1870**	151.0972	284.1605	142.5839	3
15	1461.7770	731.3921	1444.7505	722.8789	1489.7719	**745.3896**	1472.7454	736.8763	P	**244.1656**	122.5864	227.1390	114.0731	2
16									K	**147.1128**	74.0600	130.0863	65.5468	1

MS/MS Fragmentation of HEAAGDGTTTASILAR
Found in gi|84468442, putative rubisco subunit binding-protein alpha subunit [Trifolium pratense]

Match to Query 95: 1649.729108 from(825.871830,2+) intensity(11158.0000)

Title: 40: Scan 1403 (rt=29.0474, f=2, i=73)

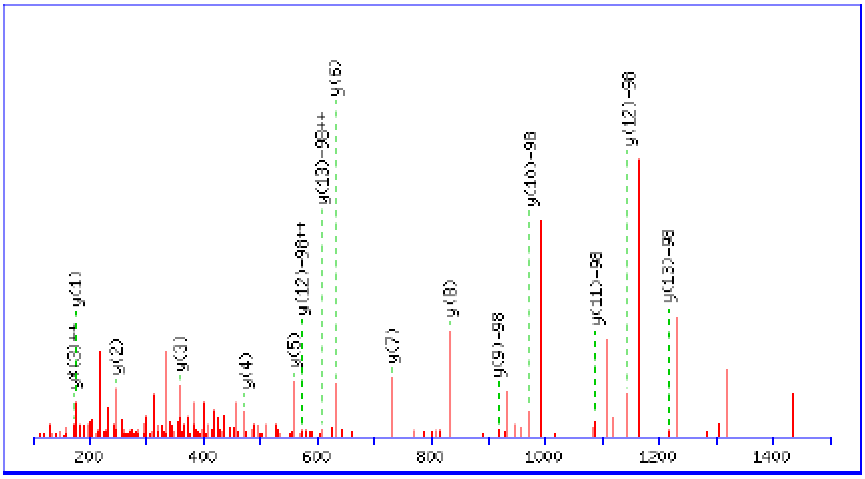


```
Monoisotopic mass of neutral peptide Mr(calc): 1649.7410
Fixed modifications: Carbamidomethyl (C)
Variable modifications:
T8      : Phospho (ST), with neutral losses 97.9769(shown in table), 0.0000
```

```
Ions Score: 50   Expect: 0.026
Matches (Bold Red): 16/180 fragment ions using 61 most intense peaks
```

#	a	a^{++}	b	b^{++}	Seq.	y	y^{++}	y*	y^{*++}	#
1	110.0713	55.5393	138.0662	69.5367	H					16
2	239.1139	120.0606	267.1088	134.0580	E	1415.7125	708.3599	1398.6859	699.8466	15
3	310.1510	155.5791	338.1459	169.5766	A	1286.6699	643.8386	1269.6434	635.3253	14
4	381.1881	191.0977	409.1830	205.0951	A	**1215.6328**	**608.3200**	1198.6062	599.8068	13
5	438.2096	219.6084	466.2045	233.6059	G	**1144.5957**	**572.8015**	1127.5691	564.2882	12
6	553.2365	277.1219	581.2314	291.1193	D	**1087.5742**	544.2907	1070.5477	535.7775	11
7	610.2580	305.6326	638.2529	319.6301	G	**972.5473**	486.7773	955.5207	478.2640	10
8	693.2951	347.1512	721.2900	361.1486	T	**915.5258**	458.2665	898.4993	449.7533	9
9	794.3428	397.6750	822.3377	411.6725	T	**832.4887**	416.7480	815.4621	408.2347	8
10	895.3904	448.1989	923.3853	462.1963	T	**731.4410**	366.2241	714.4145	357.7109	7
11	966.4275	483.7174	994.4225	497.7149	A	**630.3933**	315.7003	613.3668	307.1870	6
12	1053.4596	527.2334	1081.4545	541.2309	S	**559.3562**	280.1817	542.3297	271.6685	5
13	1166.5436	583.7755	1194.5386	597.7729	I	**472.3242**	236.6657	455.2976	228.1525	4
14	1279.6277	640.3175	1307.6226	654.3149	L	**359.2401**	180.1237	342.2136	**171.6104**	3
15	1350.6648	675.8360	1378.6597	689.8335	A	**246.1561**	123.5817	229.1295	115.0684	2
16					R	**175.1190**	88.0631	158.0924	79.5498	1

MS/MS Fragmentation of QLDASGKPDSFTGK
Found in gi|13926291, AT5g66570/K1F13_25 [Arabidopsis thaliana]

Match to Query 74: 1529.760768 from(765.887660,2+) intensity(3207.0000)
Title: 28: Sum of 6 scans in range 1207 (rt=25.1187, f=2, i=69) to 1214 (rt=25.4057, f=3, i=32)

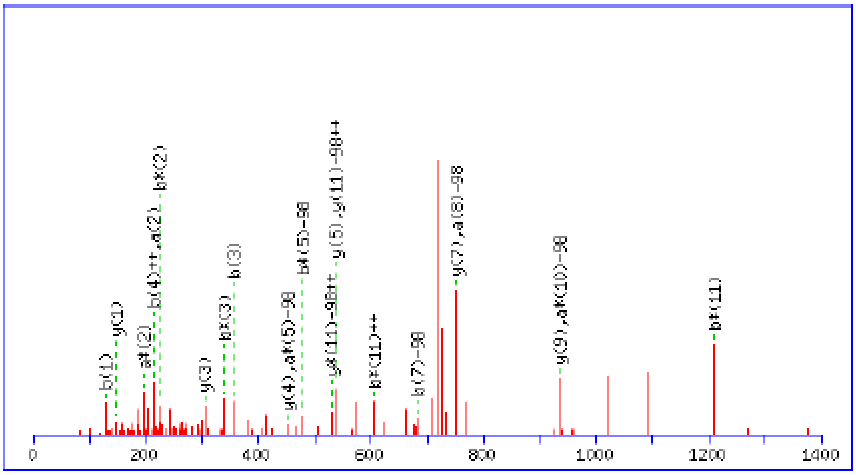

Monoisotopic mass of neutral peptide Mr(calc): 1529.6763
Fixed modifications: Carbamidomethyl (C)
Variable modifications:

S5 : Phospho (ST), with neutral losses 97.9769(shown in table), 0.0000
Ions Score: 29 **Expect:** 4
Matches (Bold Red): 22/244 fragment ions using 33 most intense peaks

#	a	a++	a*	a*++	b	b++	b*	b*++	Seq.	y	y++	y*	y*++	#
1	101.0709	51.0391	84.0444	42.5258	**129.0659**	65.0366	112.0393	56.5233	Q					14
2	**214.1550**	107.5811	**197.1285**	99.0679	242.1499	121.5786	**225.1234**	113.0653	L	1304.6481	652.8277	1287.6216	644.3144	13
3	329.1819	165.0946	312.1554	156.5813	**357.1769**	179.0921	**340.1503**	170.5788	D	1191.5640	596.2857	1174.5375	587.7724	12
4	400.2191	200.6132	383.1925	192.0999	428.2140	**214.6106**	411.1874	206.0974	A	1076.5371	**538.7722**	1059.5105	**530.2589**	11
5	469.2405	235.1239	**452.2140**	226.6106	497.2354	249.1214	**480.2089**	240.6081	S	1005.5000	503.2536	988.4734	494.7404	10
6	526.2620	263.6346	509.2354	255.1214	554.2569	277.6321	537.2303	269.1188	G	**936.4785**	468.7429	919.4520	460.2296	9
7	654.3569	327.6821	637.3304	319.1688	**682.3519**	341.6796	665.3253	333.1663	K	879.4571	440.2322	862.4305	431.7189	8
8	**751.4097**	376.2085	734.3832	367.6952	779.4046	390.2059	762.3781	381.6927	P	**751.3621**	376.1847	734.3355	367.6714	7
9	866.4367	433.7220	849.4101	425.2087	894.4316	447.7194	877.4050	439.2061	D	654.3093	327.6583	637.2828	319.1450	6
10	953.4687	477.2380	**936.4421**	468.7247	981.4636	491.2354	964.4370	482.7222	S	**539.2824**	270.1448	522.2558	261.6316	5
11	1100.5371	550.7722	1083.5105	542.2589	1128.5320	564.7696	1111.5055	556.2564	F	**452.2504**	226.6288	435.2238	218.1155	4
12	1201.5848	601.2960	1184.5582	592.7827	1229.5797	615.2935	1212.5531	606.7802	T	**305.1819**	153.0946	288.1554	144.5813	3
13	1258.6062	629.8068	1241.5797	621.2935	1286.6012	643.8042	1269.5746	635.2909	G	204.1343	102.5708	187.1077	94.0575	2
14									K	**147.1128**	74.0600	130.0863	65.5468	1

MS/MS Fragmentation of SMNEDVDLSFKK
Found in gi|18416540, Clp amino terminal domain-containing protein [Arabidopsis thaliana]

Match to Query 76: 1491.683748 from(746.849150,2+) intensity(2400.0000)
Title: 54: Sum of 3 scans in range 1358 (rt=30.1087, f=2, i=104) to 1364 (rt=30.3547, f=2, i=106)

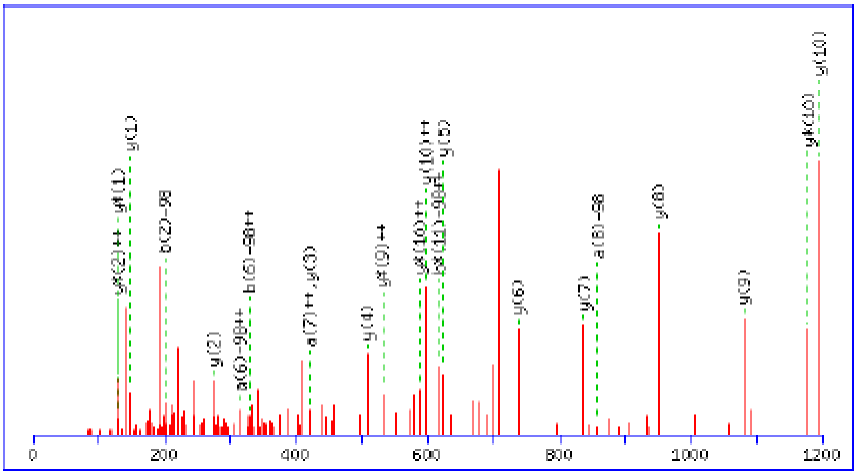

Monoisotopic mass of neutral peptide Mr(calc): 1491.6316
Fixed modifications: Carbamidomethyl (C)
Variable modifications:

S1 : Phospho (ST), with neutral losses 97.9769(shown in table), 0.0000
Ions Score: 43 **Expect:** 0.12
Matches (Bold Red): 22/204 fragment ions using 51 most intense peaks

#	a	a++	a*	a*++	b	b++	b*	b*++	Seq.	y	y++	y*	y*++	#
1	42.0338	21.5205			70.0287	35.5180			S					12
2	173.0743	87.0408			**201.0692**	101.0382			M	1325.6406	663.3239	1308.6140	654.8107	11
3	287.1172	144.0623	270.0907	135.5490	315.1121	158.0597	298.0856	149.5464	N	**1194.6001**	**597.8037**	**1177.5735**	**589.2904**	10
4	416.1598	208.5835	399.1333	200.0703	444.1547	222.5810	427.1282	214.0677	E	**1080.5572**	540.7822	1063.5306	**532.2689**	9
5	531.1868	266.0970	514.1602	257.5837	559.1817	280.0945	542.1551	271.5812	D	**951.5146**	476.2609	934.4880	467.7477	8
6	630.2552	**315.6312**	613.2286	307.1180	658.2501	**329.6287**	641.2235	321.1154	V	**836.4876**	418.7475	819.4611	410.2342	7
7	745.2821	373.1447	728.2556	364.6314	773.2770	387.1422	756.2505	378.6289	D	**737.4192**	369.2132	720.3927	360.7000	6
8	**858.3662**	429.6867	841.3396	421.1735	886.3611	443.6842	869.3346	435.1709	L	**622.3923**	311.6998	605.3657	303.1865	5
9	945.3982	473.2027	928.3717	464.6895	973.3931	487.2002	956.3666	478.6869	S	**509.3082**	255.1577	492.2817	246.6445	4
10	1092.4666	546.7370	1075.4401	538.2237	1120.4615	560.7344	1103.4350	552.2211	F	**422.2762**	211.6417	405.2496	203.1285	3
11	1220.5616	610.7844	1203.5350	602.2712	1248.5565	624.7819	1231.5300	**616.2686**	K	**275.2078**	138.1075	258.1812	**129.5942**	2
12									K	**147.1128**	74.0600	**130.0863**	65.5468	1

MS/MS Fragmentation of VPSSGLMPASDVLIR
Found in gi|18390982, ATP-dependent Clp protease proteolytic subunit, putative [Arabidopsis thaliana]

Match to Query 75: 1636.876368 from(819.445460,2+) intensity(926.0000)
Title: 64: Sum of 3 scans in range 1448 (rt=33.1796, f=2, i=149) to 1454 (rt=33.4255, f=2, i=151)

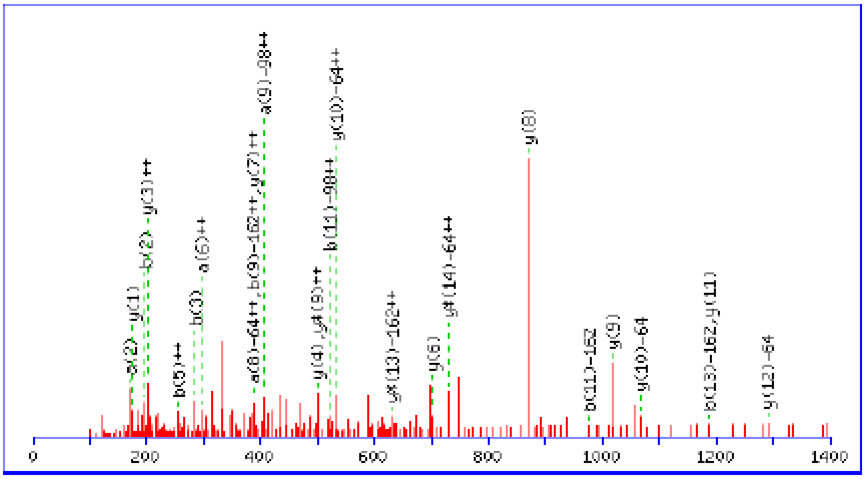

Monoisotopic mass of neutral peptide Mr(calc): 1636.7896
Fixed modifications: Carbamidomethyl (C)
Variable modifications:

S4 : Phospho (ST), with neutral losses 97.9769(shown in table), 0.0000
M7 : Oxidation (M), with neutral losses 63.9983(shown in table), 0.0000
Ions Score: 15 **Expect:** 85
Matches (Bold Red): 25/268 fragment ions using 53 most intense peaks

#	a	a++	b	b++	Seq.	y	y++	y*	y*++	#
1	72.0808	36.5440	100.0757	50.5415	V					15
2	**169.1335**	85.0704	**197.1285**	99.0679	P	1376.7532	688.8803	1359.7267	680.3670	14
3	256.1656	128.5864	**284.1605**	142.5839	S	1279.7005	640.3539	1262.6739	**631.8406**	13
4	325.1870	163.0971	353.1819	177.0946	S	1192.6684	596.8379	1175.6419	588.3246	12
5	382.2085	191.6079	410.2034	205.6053	G	1123.6470	562.3271	1106.6204	553.8139	11
6	495.2926	248.1499	523.2875	262.1474	L	**1066.6255**	**533.8164**	1049.5990	525.3031	10
7	578.3297	289.6685	606.3246	303.6659	M	953.5415	477.2744	936.5149	468.7611	9
8	675.3824	338.1949	703.3773	352.1923	P	**870.5043**	435.7558	853.4778	427.2425	8
9	746.4195	373.7134	774.4145	**387.7109**	A	773.4516	**387.2294**	756.4250	378.7162	7
10	833.4516	417.2294	861.4465	431.2269	S	**702.4145**	351.7109	685.3879	343.1976	6
11	948.4785	474.7429	**976.4734**	488.7404	D	615.3824	308.1949	598.3559	299.6816	5
12	1047.5469	524.2771	1075.5418	538.2746	V	**500.3555**	250.6814	483.3289	242.1681	4
13	1160.6310	580.8191	**1188.6259**	594.8166	L	401.2871	**201.1472**	384.2605	192.6339	3
14	1273.7151	637.3612	1301.7100	651.3586	I	288.2030	144.6051	271.1765	136.0919	2
15					R	**175.1190**	88.0631	158.0924	79.5498	1

MS/MS Fragmentation of **LLDQSSVSHLFPVTK**
Found in **gi|15224993**, PAA2 (20S PROTEASOME SUBUNIT PAA2); peptidase [Arabidopsis thaliana]

Match to Query 88: 1749.917888 from(875.966220,2+) intensity(10718.0000)
Title: 95: Sum of 3 scans in range 1660 (rt=38.5009, f=2, i=168) to 1662 (rt=38.5691, f=2, i=170)

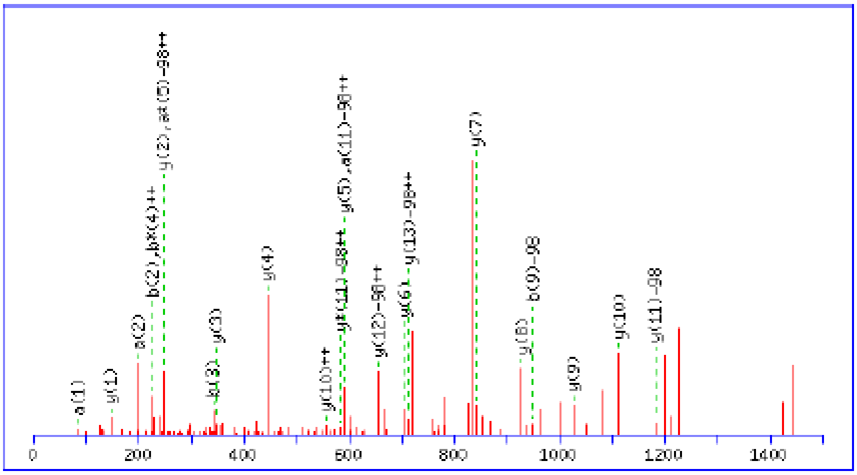

Monoisotopic mass of neutral peptide Mr(calc): 1749.8703
Fixed modifications: Carbamidomethyl (C)
Variable modifications:

S5 : Phospho (ST), with neutral losses 97.9769(shown in table), 0.0000
Ions Score: 57 **Expect:** 0.0056
Matches (Bold Red): 23/252 fragment ions using 48 most intense peaks

#	a	a++	a*	a*++	b	b++	b*	b*++	Seq.	y	y++	y*	y*++	#
1	**86.0964**	43.5519			114.0913	57.5493			L					15
2	**199.1805**	100.0939			**227.1754**	114.0913			L	1539.8166	770.4119	1522.7900	761.8986	14
3	314.2074	157.6074			**342.2023**	171.6048			D	1426.7325	**713.8699**	1409.7059	705.3566	13
4	442.2660	221.6366	425.2395	213.1234	470.2609	235.6341	453.2344	**227.1208**	Q	1311.7056	**656.3564**	1294.6790	647.8431	12
5	511.2875	256.1474	494.2609	**247.6341**	539.2824	270.1448	522.2558	261.6316	S	**1183.6470**	592.3271	1166.6204	**583.8139**	11
6	598.3195	299.6634	581.2929	291.1501	626.3144	313.6608	609.2879	305.1476	S	**1114.6255**	**557.8164**	1097.5990	549.3031	10
7	697.3879	349.1976	680.3614	340.6843	725.3828	363.1950	708.3563	354.6818	V	**1027.5935**	514.3004	1010.5669	505.7871	9
8	784.4199	392.7136	767.3934	384.2003	812.4149	406.7111	795.3883	398.1978	S	**928.5251**	464.7662	911.4985	456.2529	8
9	921.4788	461.2431	904.4523	452.7298	**949.4738**	475.2405	932.4472	466.7272	H	**841.4931**	421.2502	824.4665	412.7369	7
10	1034.5629	517.7851	1017.5364	509.2718	1062.5578	531.7826	1045.5313	523.2693	L	**704.4341**	352.7207	687.4076	344.2074	6
11	1181.6313	**591.3193**	1164.6048	582.8060	1209.6262	605.3168	1192.5997	596.8035	F	**591.3501**	296.1787	574.3235	287.6654	5
12	1278.6841	639.8457	1261.6575	631.3324	1306.6790	653.8431	1289.6525	645.3299	P	**444.2817**	222.6445	427.2551	214.1312	4
13	1377.7525	689.3799	1360.7260	680.8666	1405.7474	703.3773	1388.7209	694.8641	V	**347.2289**	174.1181	330.2023	165.6048	3
14	1478.8002	739.9037	1461.7736	731.3905	1506.7951	753.9012	1489.7685	745.3879	T	**248.1605**	124.5839	231.1339	116.0706	2
15									K	**147.1128**	74.0600	130.0863	65.5468	1

MS/MS Fragmentation of ATSAGMKEQEAVNFLEK
Found in gi|15224993, PAA2 (20S PROTEASOME SUBUNIT PAA2); peptidase [Arabidopsis thaliana]

Match to Query 93: 1931.926808 from(966.970680,2+) intensity(4904.0000)
Title: 71: Sum of 3 scans in range 1559 (rt=35.1413, f=2, i=129) to 1563 (rt=35.3055, f=2, i=131)

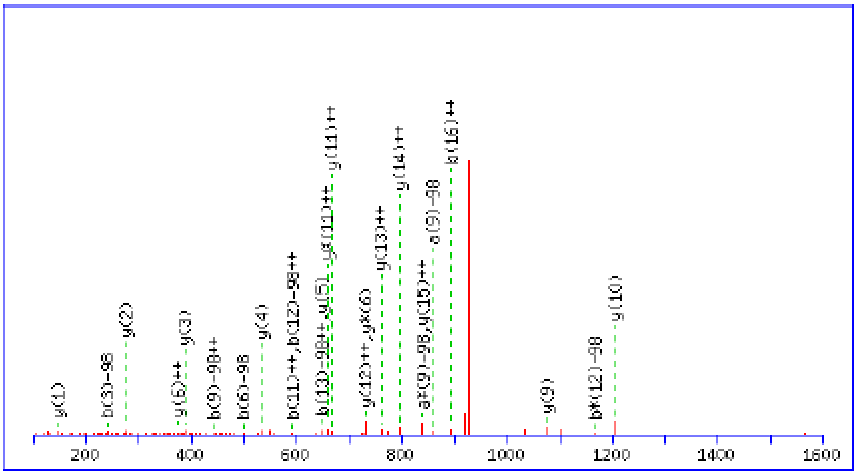

Monoisotopic mass of neutral peptide Mr(calc): 1931.8699
Fixed modifications: Carbamidomethyl (C)
Variable modifications:

T2 : Phospho (ST), with neutral losses 97.9769(shown in table), 0.0000
Ions Score: 54 **Expect:** 0.011
Matches (Bold Red): 25/272 fragment ions using 33 most intense peaks

#	a	a++	a*	a*++	b	b++	b*	b*++	Seq.	y	y++	y*	y*++	#
1	44.0495	22.5284			72.0444	36.5258			A					17
2	127.0866	64.0469			155.0815	78.0444			T	1763.8633	882.4353	1746.8367	873.9220	16
3	214.1186	107.5629			**242.1135**	121.5604			S	1680.8261	**840.9167**	1663.7996	832.4034	15
4	285.1557	143.0815			313.1506	157.0790			A	1593.7941	**797.4007**	1576.7676	788.8874	14
5	342.1772	171.5922			370.1721	185.5897			G	1522.7570	**761.8821**	1505.7305	753.3689	13
6	473.2177	237.1125			**501.2126**	251.1099			M	1465.7355	**733.3714**	1448.7090	724.8581	12
7	601.3126	301.1600	584.2861	292.6467	629.3076	315.1574	612.2810	306.6441	K	1334.6951	**667.8512**	1317.6685	**659.3379**	11
8	730.3552	365.6813	713.3287	357.1680	758.3501	379.6787	741.3236	371.1654	E	**1206.6001**	603.8037	1189.5735	595.2904	10
9	**858.4138**	429.7105	**841.3873**	421.1973	886.4087	**443.7080**	869.3822	435.1947	Q	**1077.5575**	539.2824	1060.5310	530.7691	9
10	987.4564	494.2318	970.4299	485.7186	1015.4513	508.2293	998.4248	499.7160	E	949.4989	475.2531	932.4724	466.7398	8
11	1058.4935	529.7504	1041.4670	521.2371	1086.4884	543.7479	1069.4619	535.2346	A	820.4563	410.7318	803.4298	402.2185	7
12	1157.5619	579.2846	1140.5354	570.7713	1185.5568	**593.2821**	**1168.5303**	584.7688	V	749.4192	**375.2132**	**732.3927**	366.7000	6
13	1271.6049	636.3061	1254.5783	627.7928	1299.5998	**650.3035**	1282.5732	641.7902	N	**650.3508**	325.6790	633.3243	317.1658	5
14	1418.6733	709.8403	1401.6467	701.3270	1446.6682	723.8377	1429.6416	715.3245	F	**536.3079**	268.6576	519.2813	260.1443	4
15	1531.7573	766.3823	1514.7308	757.8690	1559.7522	780.3798	1542.7257	771.8665	L	**389.2395**	195.1234	372.2129	186.6101	3
16	1660.7999	830.9036	1643.7734	822.3903	1688.7948	844.9011	1671.7683	836.3878	E	**276.1554**	138.5813	259.1288	130.0681	2
17									K	**147.1128**	74.0600	130.0863	65.5468	1

MS/MS Fragmentation of LLDQSSVTHLFPITK
Found in gi|2511588, multicatalytic endopeptidase complex, proteasome component, alpha subunit [Arabidopsis thaliana]

Match to Query 92: 1777.947468 from(889.981010,2+) intensity(6890.0000)
Title: 97: Sum of 4 scans in range 1694 (rt=39.2783, f=2, i=175) to 1697 (rt=39.3809, f=2, i=178)

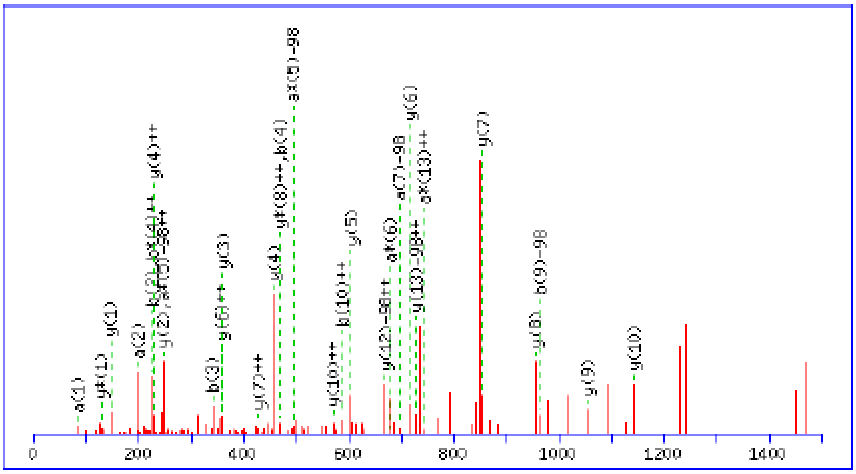

Monoisotopic mass of neutral peptide Mr(calc): 1777.9016
Fixed modifications: Carbamidomethyl (C)
Variable modifications:

S5 : Phospho (ST), with neutral losses 97.9769(shown in table), 0.0000
Ions Score: 47 **Expect:** 0.054
Matches (Bold Red): 31/252 fragment ions using 60 most intense peaks

#	a	a++	a*	a*++	b	b++	b*	b*++	Seq.	y	y++	y*	y*++	#
1	86.0964	43.5519			114.0913	57.5493			L					15
2	199.1805	100.0939			227.1754	114.0913			L	1567.8479	784.4276	1550.8213	775.9143	14
3	314.2074	157.6074			342.2023	171.6048			D	1454.7638	727.8855	1437.7373	719.3723	13
4	442.2660	221.6366	425.2395	213.1234	470.2609	235.6341	453.2344	227.1208	Q	1339.7369	670.3721	1322.7103	661.8588	12
5	511.2875	256.1474	494.2609	247.6341	539.2824	270.1448	522.2558	261.6316	S	1211.6783	606.3428	1194.6517	597.8295	11
6	598.3195	299.6634	581.2929	291.1501	626.3144	313.6608	609.2879	305.1476	S	1142.6568	571.8320	1125.6303	563.3188	10
7	697.3879	349.1976	680.3614	340.6843	725.3828	363.1950	708.3563	354.6818	V	1055.6248	528.3160	1038.5982	519.8028	9
8	798.4356	399.7214	781.4090	391.2082	826.4305	413.7189	809.4040	405.2056	T	956.5564	478.7818	939.5298	470.2686	8
9	935.4945	468.2509	918.4679	459.7376	963.4894	482.2483	946.4629	473.7351	H	855.5087	428.2580	838.4822	419.7447	7
10	1048.5786	524.7929	1031.5520	516.2796	1076.5735	538.7904	1059.5469	530.2771	L	718.4498	359.7285	701.4232	351.2153	6
11	1195.6470	598.3271	1178.6204	589.8139	1223.6419	612.3246	1206.6153	603.8113	F	605.3657	303.1865	588.3392	294.6732	5
12	1292.6997	646.8535	1275.6732	638.3402	1320.6947	660.8510	1303.6681	652.3377	P	458.2973	229.6523	441.2708	221.1390	4
13	1405.7838	703.3955	1388.7573	694.8823	1433.7787	717.3930	1416.7522	708.8797	I	361.2445	181.1259	344.2180	172.6126	3
14	1506.8315	753.9194	1489.8049	745.4061	1534.8264	767.9168	1517.7999	759.4036	T	248.1605	124.5839	231.1339	116.0706	2
15									K	147.1128	74.0600	130.0863	65.5468	1

MS/MS Fragmentation of **KLPSILVDEASVQK**
Found in **gi|15219317**, 20S proteasome alpha subunit B, putative [Arabidopsis thaliana]

Match to Query 81: 1605.753288 from(803.883920,2+) intensity(9310.0000)

Title: 69: Scan 1509 (rt=34.1016, f=2, i=94)

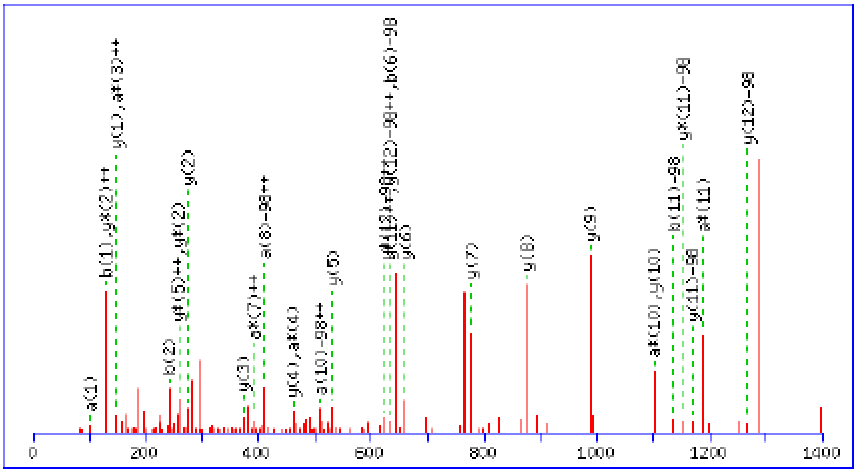


```
Monoisotopic mass of neutral peptide Mr(calc): 1605.8379
Fixed modifications: Carbamidomethyl (C)
Variable modifications:
S4      : Phospho (ST), with neutral losses 97.9769(shown in table), 0.0000
Ions Score: 62   Expect: 0.0015
```

Matches (**Bold Red**): 31/248 fragment ions using 54 most intense peaks

#	a	a^{++}	a^{*}	a^{*++}	b	b^{++}	b^{*}	b^{*++}	Seq.	y	y^{++}	y^{*}	y^{*++}	#
1	**101.1073**	51.0573	84.0808	42.5440	**129.1022**	65.0548	112.0757	56.5415	K					14
2	214.1914	107.5993	197.1648	99.0861	**242.1863**	121.5968	225.1598	113.0835	L	1380.7733	690.8903	1363.7468	682.3770	13
3	311.2442	156.1257	294.2176	**147.6124**	339.2391	170.1232	322.2125	161.6099	P	**1267.6892**	**634.3483**	1250.6627	**625.8350**	12
4	380.2656	190.6364	363.2391	182.1232	408.2605	204.6339	391.2340	196.1206	S	**1170.6365**	585.8219	**1153.6099**	577.3086	11
5	493.3497	247.1785	476.3231	238.6652	521.3446	261.1759	504.3180	252.6627	I	**1101.6150**	551.3111	1084.5885	542.7979	10
6	606.4337	303.7205	589.4072	295.2072	**634.4287**	317.7180	617.4021	309.2047	L	**988.5310**	494.7691	971.5044	486.2558	9
7	705.5022	353.2547	688.4756	344.7414	733.4971	367.2522	716.4705	358.7389	V	**875.4469**	438.2271	858.4203	429.7138	8
8	820.5291	**410.7682**	803.5025	402.2549	848.5240	424.7656	831.4975	416.2524	D	**776.3785**	388.6929	759.3519	380.1796	7
9	949.5717	475.2895	932.5451	466.7762	977.5666	489.2869	960.5401	480.7737	E	**661.3515**	331.1794	644.3250	322.6661	6
10	1020.6088	**510.8080**	1003.5823	502.2948	1048.6037	524.8055	1031.5772	516.2922	A	**532.3089**	266.6581	515.2824	**258.1448**	5
11	1107.6408	554.3241	1090.6143	545.8108	**1135.6357**	568.3215	1118.6092	559.8082	S	**461.2718**	231.1396	444.2453	222.6263	4
12	1206.7092	603.8583	1189.6827	595.3450	1234.7042	617.8557	1217.6776	609.3424	V	**374.2398**	187.6235	357.2132	179.1103	3
13	1334.7678	667.8875	1317.7413	659.3743	1362.7627	681.8850	1345.7362	673.3717	Q	**275.1714**	138.0893	**258.1448**	**129.5761**	2
14									K	**147.1128**	74.0600	130.0863	65.5468	1

MS/MS Fragmentation of LYKEPIPVTQLVR
Found in gi|15219317, 20S proteasome alpha subunit B, putative [Arabidopsis thaliana]

Match to Query 86: 1634.796688 from(818.405620,2+) intensity(13424.0000)
Title: 76: Sum of 3 scans in range 1534 (rt=34.9465, f=4, i=82) to 1536 (rt=35.0145, f=4, i=84)

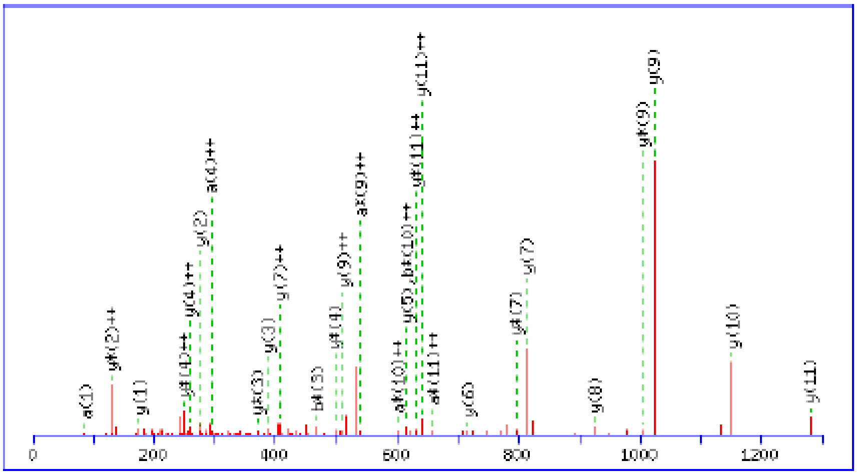

Monoisotopic mass of neutral peptide Mr(calc): 1634.8797
Fixed modifications: Carbamidomethyl (C)
Variable modifications:

Y2 : Phospho (Y)
Ions Score: 42 **Expect:** 0.17
Matches (**Bold Red**): 28/136 fragment ions using 63 most intense peaks

#	a	a++	a*	a*++	b	b++	b*	b*++	Seq.	y	y++	y*	y*++	#
1	**86.0964**	43.5519			114.0913	57.5493			L					13
2	329.1261	165.0667			357.1210	179.0641			Y	1522.8029	761.9051	1505.7764	753.3918	12
3	457.2210	229.1142	440.1945	220.6009	485.2160	243.1116	**468.1894**	234.5983	K	**1279.7732**	**640.3903**	1262.7467	**631.8770**	11
4	586.2636	**293.6355**	569.2371	285.1222	614.2585	307.6329	597.2320	299.1196	E	**1151.6783**	576.3428	1134.6517	567.8295	10
5	683.3164	342.1618	666.2898	333.6486	711.3113	356.1593	694.2848	347.6460	P	**1022.6357**	**511.8215**	**1005.6091**	503.3082	9
6	796.4005	398.7039	779.3739	390.1906	824.3954	412.7013	807.3688	404.1881	I	**925.5829**	463.2951	908.5564	454.7818	8
7	893.4532	447.2303	876.4267	438.7170	921.4481	461.2277	904.4216	452.7144	P	**812.4989**	**406.7531**	**795.4723**	398.2398	7
8	992.5216	496.7645	975.4951	488.2512	1020.5166	510.7619	1003.4900	502.2486	V	**715.4461**	358.2267	698.4196	349.7134	6
9	1093.5693	547.2883	1076.5428	**538.7750**	1121.5642	561.2858	1104.5377	552.7725	T	**616.3777**	308.6925	599.3511	300.1792	5
10	1221.6279	611.3176	1204.6013	**602.8043**	1249.6228	625.3150	1232.5963	**616.8018**	Q	515.3300	**258.1686**	**498.3035**	**249.6554**	4
11	1334.7120	667.8596	1317.6854	**659.3463**	1362.7069	681.8571	1345.6803	673.3438	L	**387.2714**	194.1394	**370.2449**	185.6261	3
12	1433.7804	717.3938	1416.7538	708.8806	1461.7753	731.3913	1444.7487	722.8780	V	**274.1874**	137.5973	257.1608	**129.0840**	2
13									R	**175.1190**	88.0631	158.0924	79.5498	1

MS/MS Fragmentation of RYTEDMELDDAIHTAILTLK
Found in gi|15219317, 20S proteasome alpha subunit B, putative [Arabidopsis thaliana]

Match to Query 123: 2443.020672 from(815.347500,3+) intensity(13570.0000)
Title: 110: Sum of 3 scans in range 1761 (rt=41.5868, f=2, i=178) to 1763 (rt=41.6548, f=2, i=180)

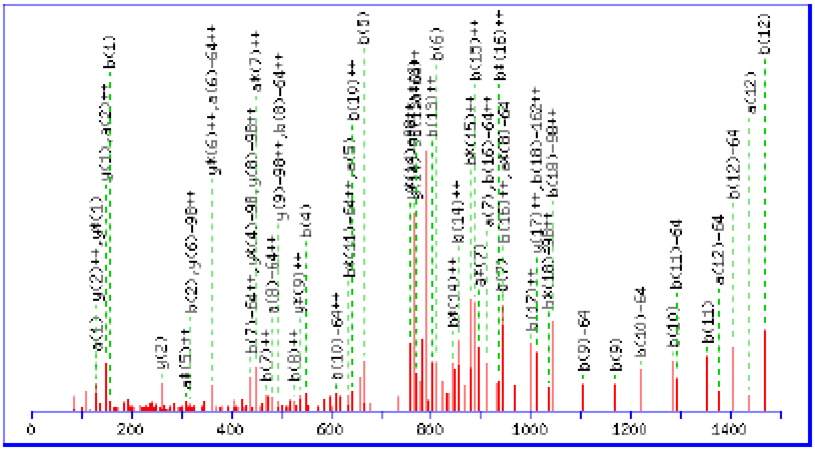

Monoisotopic mass of neutral peptide Mr(calc): 2443.1342
Fixed modifications: Carbamidomethyl (C)
Variable modifications:
M6 : Oxidation (M), with neutral losses 0.0000(shown in table), 63.9983
T18 : Phospho (ST), with neutral losses 97.9769(shown in table), 0.0000
Ions Score: 60 **Expect:** 0.0027

Matches (Bold Red): 60/480 fragment ions using 68 most intense peaks

#	a	a++	a*	a*++	b	b++	b*	b*++	Seq.	y	y++	y*	y*++	#
1	**129.1135**	65.0604	112.0869	56.5471	**157.1084**	79.0578	140.0818	70.5446	R					20
2	292.1768	**146.5920**	275.1503	138.0788	**320.1717**	160.5895	303.1452	152.0762	Y	2190.0635	1095.5354	2173.0369	1087.0221	19
3	393.2245	197.1159	376.1979	188.6026	421.2194	211.1133	404.1928	202.6001	T	2027.0001	1014.0037	2009.9736	1005.4904	18
4	522.2671	261.6372	505.2405	253.1239	**550.2620**	275.6346	533.2354	267.1214	E	1925.9525	963.4799	1908.9259	954.9666	17
5	**637.2940**	319.1506	620.2675	**310.6374**	**665.2889**	333.1481	648.2624	324.6348	D	1796.9099	898.9586	1779.8833	890.4453	16
6	784.3294	392.6683	**767.3029**	384.1551	**812.3243**	406.6658	795.2978	398.1525	M	1681.8829	841.4451	1664.8564	832.9318	15
7	**913.3720**	457.1896	**896.3455**	**448.6764**	**941.3669**	**471.1871**	924.3404	462.6738	E	1534.8475	**767.9274**	1517.8210	**759.4141**	14
8	1026.4561	513.7317	1009.4295	505.2184	1054.4510	**527.7291**	1037.4244	519.2159	L	1405.8049	703.4061	1388.7784	694.8928	13
9	1141.4830	571.2451	1124.4565	562.7319	**1169.4779**	585.2426	1152.4514	576.7293	D	1292.7209	646.8641	1275.6943	638.3508	12
10	1256.5100	628.7586	1239.4834	620.2453	**1284.5049**	**642.7561**	1267.4783	634.2428	D	1177.6939	589.3506	1160.6674	580.8373	11
11	1327.5471	664.2772	1310.5205	655.7639	**1355.5420**	678.2746	1338.5154	669.7614	A	1062.6670	531.8371	1045.6404	523.3239	10
12	**1440.6311**	720.8192	1423.6046	712.3059	**1468.6261**	734.8167	1451.5995	726.3034	I	991.6299	**496.3186**	974.6033	487.8053	9
13	1577.6901	789.3487	1560.6635	780.8354	1605.6850	**803.3461**	1588.6584	794.8328	H	878.5458	**439.7765**	861.5193	431.2633	8
14	1678.7377	839.8725	1661.7112	831.3592	1706.7326	**853.8700**	1689.7061	**845.3567**	T	741.4869	371.2471	724.4603	362.7338	7
15	1749.7748	875.3911	1732.7483	866.8778	1777.7698	**889.3885**	1760.7432	**880.8752**	A	640.4392	**320.7232**	623.4127	312.2100	6
16	1862.8589	931.9331	1845.8324	923.4198	1890.8538	**945.9306**	1873.8273	**937.4173**	I	569.4021	285.2047	552.3756	276.6914	5
17	1975.9430	988.4751	1958.9164	979.9619	2003.9379	**1002.4726**	1986.9113	993.9593	L	456.3180	228.6627	**439.2915**	220.1494	4
18	2058.9801	1029.9937	2041.9535	1021.4804	2086.9750	**1043.9911**	2069.9484	**1035.4779**	T	343.2340	172.1206	326.2074	163.6074	3
19	2172.0641	1086.5357	2155.0376	1078.0224	2200.0591	1100.5332	2183.0325	1092.0199	L	**260.1969**	**130.6021**	243.1703	122.0888	2
20									K	**147.1128**	74.0600	**130.0863**	65.5468	1

MS/MS Fragmentation of YTSIKPLGDR
Found in gi|15242045, CPN20 (CHAPERONIN 20); calmodulin binding [Arabidopsis thaliana]

Match to Query 36: 1228.533368 from(615.273960,2+) intensity(2238.0000)
Title: 18: Sum of 4 scans in range 1448 (rt=29.0355, f=2, i=65) to 1451 (rt=29.1381, f=2, i=68)

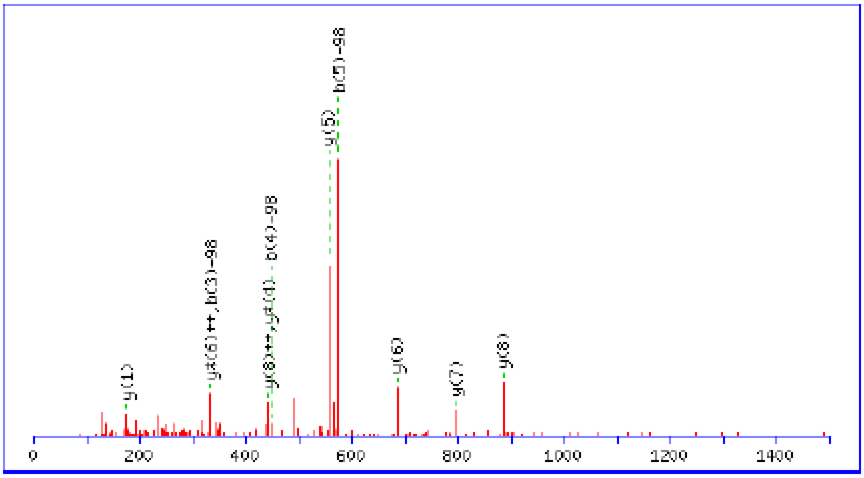

Monoisotopic mass of neutral peptide Mr(calc): 1228.5853
Fixed modifications: Carbamidomethyl (C)
Variable modifications:

T2 : Phospho (ST), with neutral losses 97.9769(shown in table), 0.0000
Ions Score: 22 **Expect:** 14
Matches (Bold Red): 11/148 fragment ions using 27 most intense peaks

#	a	a++	a*	a*++	b	b++	b*	b*++	Seq.	y	y++	y*	y*++	#
1	136.0757	68.5415			164.0706	82.5389			Y					10
2	219.1128	110.0600			247.1077	124.0575			T	968.5523	484.7798	951.5258	476.2665	9
3	306.1448	153.5761			**334.1397**	167.5735			S	**885.5152**	**443.2613**	868.4887	434.7480	8
4	419.2289	210.1181			**447.2238**	224.1155			I	**798.4832**	399.7452	781.4567	391.2320	7
5	547.3239	274.1656	530.2973	265.6523	**575.3188**	288.1630	558.2922	279.6497	K	**685.3992**	343.2032	668.3726	**334.6899**	6
6	644.3766	322.6919	627.3501	314.1787	672.3715	336.6894	655.3450	328.1761	P	**557.3042**	279.1557	540.2776	270.6425	5
7	757.4607	379.2340	740.4341	370.7207	785.4556	393.2314	768.4290	384.7182	L	460.2514	230.6293	**443.2249**	222.1161	4
8	814.4821	407.7447	797.4556	399.2314	842.4771	421.7422	825.4505	413.2289	G	347.1674	174.0873	330.1408	165.5740	3
9	929.5091	465.2582	912.4825	456.7449	957.5040	479.2556	940.4775	470.7424	D	290.1459	145.5766	273.1193	137.0633	2
10									R	**175.1190**	88.0631	158.0924	79.5498	1

MS/MS Fragmentation of YTSIKPLGDR
Found in gi|15242045, CPN20 (CHAPERONIN 20); calmodulin binding [Arabidopsis thaliana]

Match to Query 39: 1228.602528 from(615.308540,2+) intensity(13730.0000)
Title: 39: Sum of 2 scans in range 1261 (rt=26.8403, f=2, i=67) to 1262 (rt=26.8745, f=2, i=68)

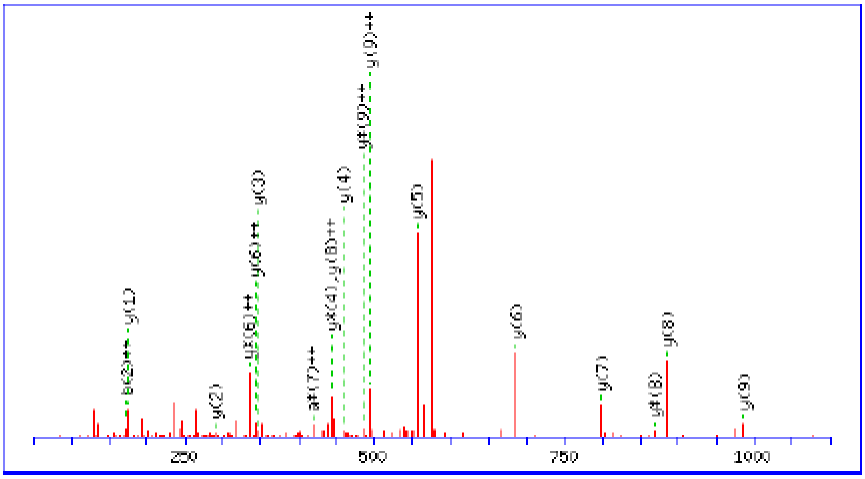

Monoisotopic mass of neutral peptide Mr(calc): 1228.5853
Fixed modifications: Carbamidomethyl (C)
Variable modifications:

Y1 : Phospho (Y)
Ions Score: 42 **Expect:** 0.15
Matches (**Bold Red**): 18/92 fragment ions using 48 most intense peaks

#	a	a++	a*	a*++	b	b++	b*	b*++	Seq.	y	y++	y*	y*++	#
1	216.0420	108.5246			244.0369	122.5221			Y					10
2	317.0897	159.0485			345.0846	**173.0459**			T	**986.5629**	**493.7851**	969.5364	**485.2718**	9
3	404.1217	202.5645			432.1166	216.5620			S	**885.5152**	**443.2613**	**868.4887**	434.7480	8
4	517.2058	259.1065			545.2007	273.1040			I	**798.4832**	399.7452	781.4567	391.2320	7
5	645.3007	323.1540	628.2742	314.6407	673.2957	337.1515	656.2691	328.6382	K	**685.3992**	**343.2032**	668.3726	**334.6899**	6
6	742.3535	371.6804	725.3270	363.1671	770.3484	385.6779	753.3219	377.1646	P	**557.3042**	279.1557	540.2776	270.6425	5
7	855.4376	428.2224	838.4110	**419.7092**	883.4325	442.2199	866.4059	433.7066	L	**460.2514**	230.6293	**443.2249**	222.1161	4
8	912.4590	456.7332	895.4325	448.2199	940.4540	470.7306	923.4274	462.2173	G	**347.1674**	174.0873	330.1408	165.5740	3
9	1027.4860	514.2466	1010.4594	505.7334	1055.4809	528.2441	1038.4543	519.7308	D	**290.1459**	145.5766	273.1193	137.0633	2
10									R	**175.1190**	88.0631	158.0924	79.5498	1

MS/MS Fragmentation of YTSIKPLGDR
Found in gi|15242045, CPN20 (CHAPERONIN 20); calmodulin binding [Arabidopsis thaliana]

Match to Query 40: 1228.613988 from(615.314270,2+) intensity(21510.0000)
Title: 34: Sum of 4 scans in range 1237 (rt=25.9879, f=2, i=62) to 1242 (rt=26.1926, f=4, i=25)

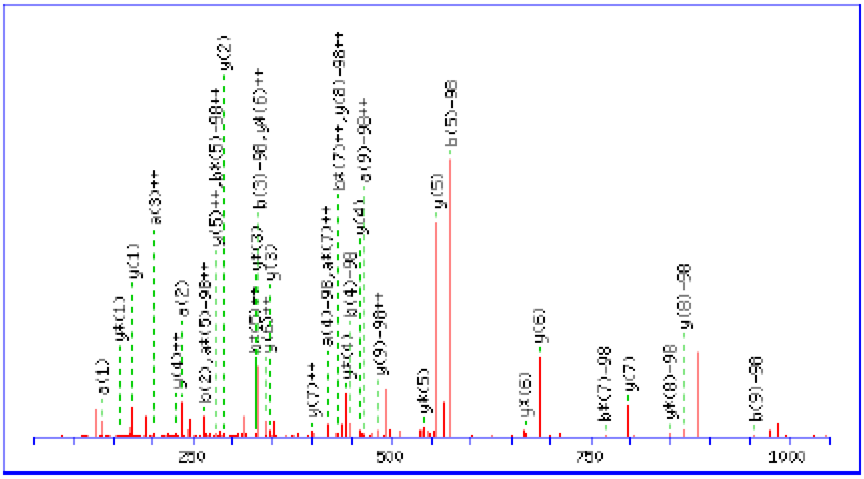

Monoisotopic mass of neutral peptide Mr(calc): 1228.5853
Fixed modifications: Carbamidomethyl (C)
Variable modifications:

S3 : Phospho (ST), with neutral losses 97.9769(shown in table), 0.0000
Ions Score: 37 **Expect:** 0.43
Matches (**Bold Red**): 37/148 fragment ions using 70 most intense peaks

#	a	a^{++}	a^{*}	a^{*++}	b	b^{++}	b^{*}	b^{*++}	Seq.	y	y^{++}	y^{*}	y^{*++}	#
1	**136.0757**	68.5415			164.0706	82.5389			Y					10
2	**237.1234**	119.0653			**265.1183**	133.0628			T	968.5523	**484.7798**	951.5258	476.2665	9
3	306.1448	153.5761			**334.1397**	167.5735			S	**867.5047**	**434.2560**	**850.4781**	425.7427	8
4	**419.2289**	210.1181			**447.2238**	224.1155			I	**798.4832**	**399.7452**	781.4567	391.2320	7
5	547.3239	274.1656	530.2973	**265.6523**	**575.3188**	288.1630	558.2922	**279.6497**	K	**685.3992**	**343.2032**	**668.3726**	**334.6899**	6
6	644.3766	322.6919	627.3501	314.1787	672.3715	336.6894	655.3450	328.1761	P	**557.3042**	**279.1557**	**540.2776**	270.6425	5
7	757.4607	379.2340	740.4341	370.7207	785.4556	393.2314	**768.4290**	384.7182	L	**460.2514**	**230.6293**	**443.2249**	222.1161	4
8	814.4821	407.7447	797.4556	399.2314	842.4771	421.7422	825.4505	413.2289	G	**347.1674**	174.0873	**330.1408**	165.5740	3
9	929.5091	**465.2582**	912.4825	456.7449	**957.5040**	479.2556	940.4775	470.7424	D	**290.1459**	145.5766	273.1193	137.0633	2
10									R	**175.1190**	88.0631	**158.0924**	79.5498	1

76d

MS/MS Fragmentation of TLGGILLPSTAQSKPQGGEVVAVGEGR
Found in gi|15242045, CPN20 (CHAPERONIN 20); calmodulin binding [Arabidopsis thaliana]

Match to Query 95: 2700.271962 from(901.097930,3+) intensity(7548.0000)
Title: 73: Sum of 3 scans in range 1679 (rt=37.6872, f=3, i=88) to 1685 (rt=37.9332, f=3, i=90)

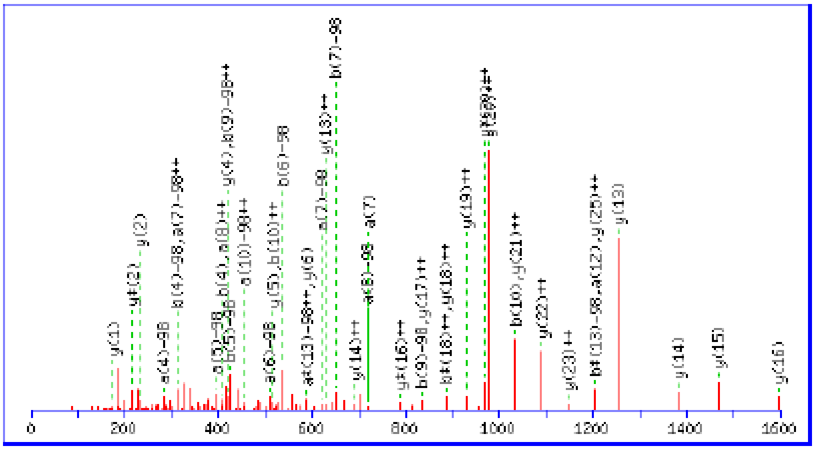


```
Monoisotopic mass of neutral peptide Mr(calc): 2700.3848
Fixed modifications: Carbamidomethyl (C)
Variable modifications:
T1      : Phospho (ST), with neutral losses 97.9769(shown in table), 0.0000
Ions Score: 56  Expect: 0.0061
Matches (Bold Red): 44/432 fragment ions using 64 most intense peaks
```

#	a	a++	a*	a*++	b	b++	b*	b*++	Seq.	y	y++	y*	y*++	#
1	56.0495	28.5284			84.0444	42.5258			T					27
2	169.1335	85.0704			197.1284	99.0679			L	2520.3780	1260.6927	2503.3515	1252.1794	26
3	226.1550	113.5811			254.1499	127.5786			G	2407.2940	**1204.1506**	2390.2674	1195.6373	25
4	**283.1765**	142.0919			**311.1714**	156.0893			G	2350.2725	1175.6399	2333.2460	1167.1266	24
5	**396.2605**	198.6339			**424.2554**	212.6314			I	2293.2510	**1147.1292**	2276.2245	1138.6159	23
6	**509.3446**	255.1759			**537.3395**	269.1734			L	2180.1670	**1090.5871**	2163.1404	1082.0739	22
7	**622.4287**	**311.7180**			**650.4236**	325.7154			L	2067.0829	**1034.0451**	2050.0564	1025.5318	21
8	**719.4814**	360.2443			747.4763	374.2418			P	1953.9988	**977.5031**	1936.9723	**968.9898**	20
9	806.5134	403.7604			**834.5084**	**417.7578**			S	1856.9461	**928.9767**	1839.9195	920.4634	19
10	907.5611	**454.2842**			935.5560	468.2817			T	1769.9141	**885.4607**	1752.8875	876.9474	18
11	978.5982	489.8028			1006.5932	503.8002			A	1668.8664	**834.9368**	1651.8398	826.4236	17
12	1106.6568	553.8320	1089.6303	545.3188	1134.6517	567.8295	1117.6252	559.3162	Q	**1597.8293**	799.4183	1580.8027	**790.9050**	16
13	1193.6888	597.3481	1176.6623	**588.8348**	1221.6838	611.3455	**1204.6572**	602.8322	S	**1469.7707**	735.3890	1452.7441	726.8757	15
14	1321.7838	661.3955	1304.7573	652.8823	1349.7787	675.3930	1332.7522	666.8797	K	**1382.7387**	**691.8730**	1365.7121	683.3597	14
15	1418.8366	709.9219	1401.8100	701.4086	1446.8315	723.9194	1429.8049	715.4061	P	**1254.6437**	**627.8255**	1237.6171	619.3122	13
16	1546.8951	773.9512	1529.8686	765.4379	1574.8901	787.9487	1557.8635	779.4354	Q	1157.5909	579.2991	1140.5644	570.7858	12
17	1603.9166	802.4619	1586.8901	793.9487	1631.9115	816.4594	1614.8850	807.9461	G	1029.5324	515.2698	1012.5058	506.7565	11
18	1660.9381	830.9727	1643.9115	822.4594	1688.9330	844.9701	1671.9064	836.4569	G	972.5109	486.7591	955.4843	478.2458	10
19	1789.9807	895.4940	1772.9541	886.9807	1817.9756	909.4914	1800.9490	900.9782	E	915.4894	458.2483	898.4629	449.7351	9
20	1889.0491	945.0282	1872.0225	936.5149	1917.0440	959.0256	1900.0174	950.5124	V	786.4468	393.7271	769.4203	385.2138	8
21	1988.1175	994.5624	1971.0909	986.0491	2016.1124	1008.5598	1999.0859	1000.0466	V	687.3784	344.1928	670.3519	335.6796	7
22	2059.1546	1030.0809	2042.1281	1021.5677	2087.1495	1044.0784	2070.1230	1035.5651	A	**588.3100**	294.6586	571.2835	286.1454	6
23	2158.2230	1079.6152	2141.1965	1071.1019	2186.2179	1093.6126	2169.1914	1085.0993	V	**517.2729**	259.1401	500.2463	250.6268	5
24	2215.2445	1108.1259	2198.2179	1099.6126	2243.2394	1122.1233	2226.2129	1113.6101	G	**418.2045**	209.6059	401.1779	201.0926	4
25	2344.2871	1172.6472	2327.2605	1164.1339	2372.2820	1186.6446	2355.2554	1178.1314	E	361.1830	181.0951	344.1565	172.5819	3
26	2401.3085	1201.1579	2384.2820	1192.6446	2429.3035	1215.1554	2412.2769	1206.6421	G	**232.1404**	116.5738	**215.1139**	108.0606	2
27									R	**175.1190**	88.0631	158.0924	79.5498	1

MS/MS Fragmentation of ITAIKDIIPILEK
Found in gi|15226314, CPN60A (chloroplast / 60 kDa chaperonin alpha subunit); ATP binding / protein binding / unfolded protein binding [Arabidopsis thaliana]

Match to Query 83: 1545.846008 from(773.930280,2+) intensity(4407.0000)
Title: 114: Sum of 4 scans in range 1770 (rt=40.5593, f=2, i=182) to 1773 (rt=40.6686, f=2, i=185)

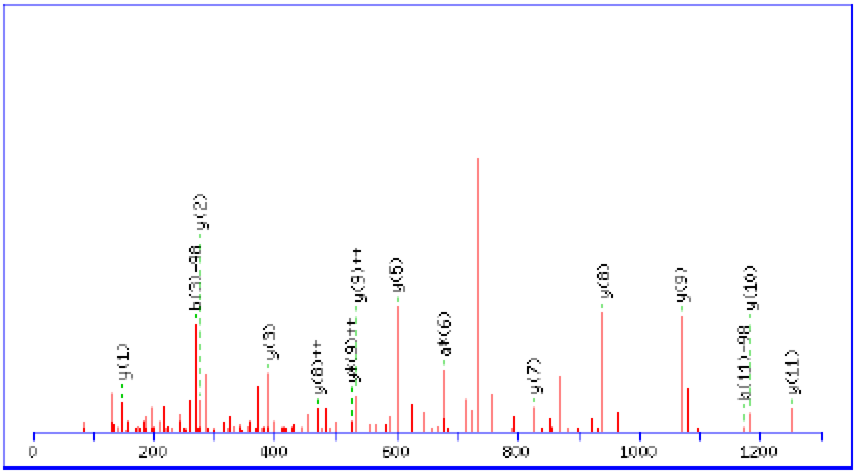

Monoisotopic mass of neutral peptide Mr(calc): 1545.8782
Fixed modifications: Carbamidomethyl (C)

Variable modifications:
T2 : Phospho (ST), with neutral losses 97.9769(shown in table), 0.0000
Ions Score: 64 **Expect:** 0.001
Matches (Bold Red): 15/208 fragment ions using 25 most intense peaks

#	a	a++	a*	a*++	b	b++	b*	b*++	Seq.	y	y++	y*	y*++	#
1	86.0964	43.5519			114.0913	57.5493			I					13
2	169.1335	85.0704			197.1284	99.0679			T	1335.8246	668.4159	1318.7981	659.9027	12
3	240.1706	120.5890			**268.1656**	134.5864			A	**1252.7875**	626.8974	1235.7610	618.3841	11
4	353.2547	177.1310			381.2496	191.1285			I	**1181.7504**	591.3788	1164.7238	582.8656	10
5	481.3497	241.1785	464.3231	232.6652	509.3446	255.1759	492.3180	246.6627	K	**1068.6663**	**534.8368**	1051.6398	**526.3235**	9
6	596.3766	298.6919	579.3501	290.1787	624.3715	312.6894	607.3450	304.1761	D	**940.5714**	**470.7893**	923.5448	462.2760	8
7	709.4607	355.2340	692.4341	346.7207	737.4556	369.2314	720.4290	360.7182	I	**825.5444**	413.2758	808.5179	404.7626	7
8	822.5447	411.7760	805.5182	403.2627	850.5397	425.7735	833.5131	417.2602	I	712.4604	356.7338	695.4338	348.2205	6
9	919.5975	460.3024	902.5710	451.7891	947.5924	474.2998	930.5659	465.7866	P	**599.3763**	300.1918	582.3497	291.6785	5
10	1032.6816	516.8444	1015.6550	508.3311	1060.6765	530.8419	1043.6499	522.3286	I	502.3235	251.6654	485.2970	243.1521	4
11	1145.7656	573.3865	1128.7391	564.8732	**1173.7606**	587.3839	1156.7340	578.8706	L	**389.2395**	195.1234	372.2129	186.6101	3
12	1274.8082	637.9078	1257.7817	629.3945	1302.8031	651.9052	1285.7766	643.3919	E	**276.1554**	138.5813	259.1288	130.0681	2
13									K	**147.1128**	74.0600	130.0863	65.5468	1

80a

MS/MS Fragmentation of VFDQEISSGQLLVVPQGFSVMK
Found in gi|166678, 12S storage protein CRB

Match to Query 104: 2503.270648 from(1252.642600,2+) intensity(4386.0000)
Title: 99: Sum of 4 scans in range 1832 (rt=41.6418, f=2, i=220) to 1835 (rt=41.751, f=2, i=223)

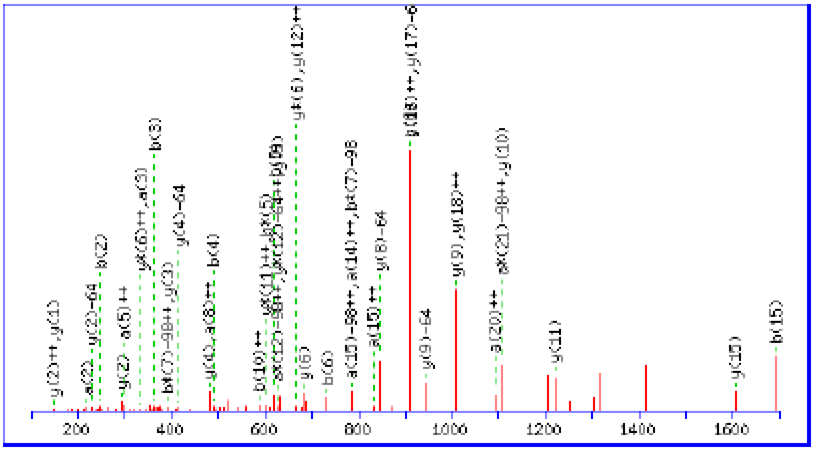

Monoisotopic mass of neutral peptide Mr(calc): 2503.2070
Fixed modifications: Carbamidomethyl (C)
Variable modifications:
S7 : Phospho (ST), with neutral losses 97.9769(shown in table), 0.0000
M21 : Oxidation (M), with neutral losses 0.0000(shown in table), 63.9983
Ions Score: 55 **Expect:** 0.0095

Matches (Bold Red): 44/504 fragment ions using 53 most intense peaks

#	a	a++	a*	a*++	b	b++	b*	b*++	Seq.	y	y++	y*	y*++	#
1	72.0808	36.5440			100.0757	50.5415			V					22
2	219.1492	110.0782			247.1441	124.0757			F	2307.1689	1154.0881	2290.1424	1145.5748	21
3	334.1761	167.5917			362.1710	181.5892			D	2160.1005	1080.5539	2143.0740	1072.0406	20
4	462.2347	231.6210	445.2082	223.1077	490.2296	245.6185	473.2031	237.1052	Q	2045.0736	1023.0404	2028.0470	1014.5272	19
5	591.2773	296.1423	574.2508	287.6290	619.2722	310.1397	602.2457	301.6265	E	1917.0150	959.0111	1899.9885	950.4979	18
6	704.3614	352.6843	687.3348	344.1710	732.3563	366.6818	715.3297	358.1685	I	1787.9724	894.4898	1770.9459	885.9766	17
7	773.3828	387.1950	756.3563	378.6818	801.3777	401.1925	784.3512	392.6792	S	1674.8884	837.9478	1657.8618	829.4345	16
8	860.4149	430.7111	843.3883	422.1978	888.4098	444.7085	871.3832	436.1952	S	1605.8669	803.4371	1588.8403	794.9238	15
9	917.4363	459.2218	900.4098	450.7085	945.4312	473.2193	928.4047	464.7060	G	1518.8349	759.9211	1501.8083	751.4078	14
10	1045.4949	523.2511	1028.4683	514.7378	1073.4898	537.2485	1056.4633	528.7353	Q	1461.8134	731.4103	1444.7869	722.8971	13
11	1158.5790	579.7931	1141.5524	571.2798	1186.5739	593.7906	1169.5473	585.2773	L	1333.7548	667.3811	1316.7283	658.8678	12
12	1271.6630	636.3351	1254.6365	627.8219	1299.6579	650.3326	1282.6314	641.8193	L	1220.6708	610.8390	1203.6442	602.3257	11
13	1370.7314	685.8694	1353.7049	677.3561	1398.7264	699.8668	1381.6998	691.3535	V	1107.5867	554.2970	1090.5601	545.7837	10
14	1469.7998	735.4036	1452.7733	726.8903	1497.7948	749.4010	1480.7682	740.8877	V	1008.5183	504.7628	991.4917	496.2495	9
15	1566.8526	783.9299	1549.8261	775.4167	1594.8475	797.9274	1577.8210	789.4141	P	909.4499	455.2286	892.4233	446.7153	8
16	1694.9112	847.9592	1677.8846	839.4460	1722.9061	861.9567	1705.8796	853.4434	Q	812.3971	406.7022	795.3706	398.1889	7
17	1751.9327	876.4700	1734.9061	867.9567	1779.9276	890.4674	1762.9010	881.9541	G	684.3385	342.6729	667.3120	334.1596	6
18	1899.0011	950.0042	1881.9745	941.4909	1926.9960	964.0016	1909.9694	955.4884	F	627.3171	314.1622	610.2905	305.6489	5
19	1986.0331	993.5202	1969.0065	985.0069	2014.0280	1007.5176	1997.0015	999.0044	S	480.2486	240.6280	463.2221	232.1147	4
20	2085.1015	1043.0544	2068.0750	1034.5411	2113.0964	1057.0519	2096.0699	1048.5386	V	393.2166	197.1119	376.1901	188.5987	3
21	2232.1369	1116.5721	2215.1104	1108.0588	2260.1318	1130.5696	2243.1053	1122.0563	M	294.1482	147.5777	277.1217	139.0645	2
22									K	147.1128	74.0600	130.0863	65.5468	1

80b

MS/MS Fragmentation of GLPLEVITNGYQISPEEAKR
Found in gi|166678, 12S storage protein CRB

Match to Query 98: 2293.076848 from(1147.545700,2+) intensity(10327.0000)
Title: 90: Sum of 4 scans in range 1589 (rt=36.8425, f=2, i=202) to 1592 (rt=36.9451, f=2, i=205)

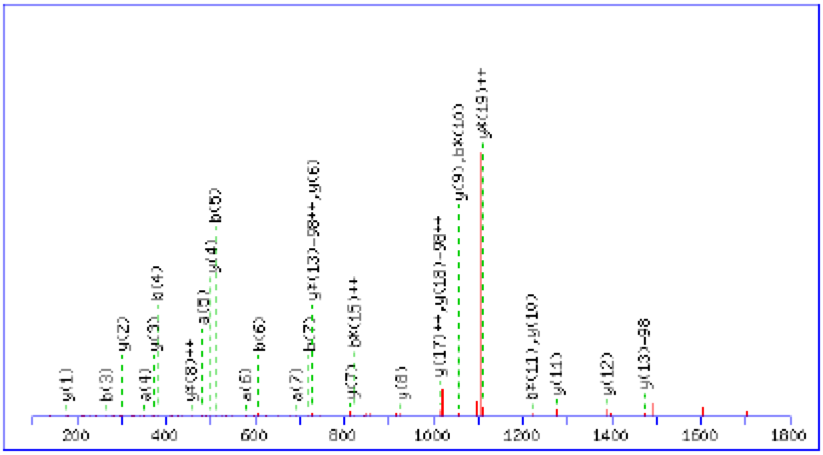

Monoisotopic mass of neutral peptide Mr(calc): 2293.1355
Fixed modifications: Carbamidomethyl (C)
Variable modifications:
T8 : Phospho (ST), with neutral losses 97.9769(shown in table), 0.0000
Ions Score: 62 **Expect:** 0.0016

Matches (**Bold Red**): 29/316 fragment ions using 45 most intense peaks

#	a	a++	a*	a*++	b	b++	b*	b*++	Seq.	y	y++	y*	y*++	#
1	30.0338	15.5206			58.0287	29.5180			G					20
2	143.1179	72.0626			171.1128	86.0600			L	2139.1444	1070.0759	2122.1179	1061.5626	19
3	240.1707	120.5890			**268.1656**	134.5864			P	2026.0604	**1013.5338**	2009.0338	1005.0206	18
4	**353.2547**	177.1310			**381.2496**	191.1285			L	1929.0076	965.0074	1911.9811	956.4942	17
5	**482.2973**	241.6523			**510.2922**	255.6498			E	1815.9235	908.4654	1798.8970	899.9521	16
6	**581.3657**	291.1865			**609.3606**	305.1840			V	1686.8810	843.9441	1669.8544	835.4308	15
7	**694.4498**	347.7285			**722.4447**	361.7260			I	1587.8125	794.4099	1570.7860	785.8966	14
8	777.4869	389.2471			805.4818	403.2445			T	**1474.7285**	737.8679	1457.7019	**729.3546**	13
9	891.5298	446.2685	874.5033	437.7553	919.5247	460.2660	902.4982	451.7527	N	**1391.6914**	696.3493	1374.6648	687.8360	12
10	948.5513	474.7793	931.5247	466.2660	976.5462	488.7767	959.5197	480.2635	G	**1277.6484**	639.3279	1260.6219	630.8146	11
11	1111.6146	556.3109	1094.5881	547.7977	1139.6095	570.3084	1122.5830	561.7951	Y	**1220.6270**	610.8171	1203.6004	602.3039	10
12	1239.6732	620.3402	1222.6466	611.8270	1267.6681	634.3377	1250.6416	625.8244	Q	**1057.5636**	529.2855	1040.5371	520.7722	9
13	1352.7573	676.8823	1335.7307	668.3690	1380.7522	690.8797	1363.7256	682.3664	I	**929.5051**	465.2562	912.4785	**456.7429**	8
14	1439.7893	720.3983	1422.7627	711.8850	1467.7842	734.3957	1450.7577	725.8825	S	**816.4210**	408.7141	799.3945	400.2009	7
15	1536.8420	768.9247	1519.8155	760.4114	1564.8370	782.9221	1547.8104	774.4088	P	**729.3890**	365.1981	712.3624	356.6849	6
16	1665.8846	833.4460	1648.8581	824.9327	1693.8796	847.4434	1676.8530	838.9301	E	632.3362	316.6717	615.3097	308.1585	5
17	1794.9272	897.9673	1777.9007	889.4540	1822.9222	911.9647	1805.8956	903.4514	E	**503.2936**	252.1504	486.2671	243.6372	4
18	1865.9643	933.4858	1848.9378	924.9725	1893.9593	947.4833	1876.9327	938.9700	A	**374.2510**	187.6292	357.2245	179.1159	3
19	1994.0593	997.5333	1977.0328	989.0200	2022.0542	1011.5308	2005.0277	1003.0175	K	**303.2139**	152.1106	286.1874	143.5973	2
20									R	**175.1190**	88.0631	158.0924	79.5498	1

MS/MS Fragmentation of TNENAQVNTLAGR
Found in gi|166678, 12S storage protein CRB

Match to Query 58: 1466.703028 from(734.358790,2+) intensity(1910.0000)
Title: 24: Sum of 4 scans in range 1404 (rt=28.0616, f=4, i=5) to 1411 (rt=28.3381, f=4, i=8)

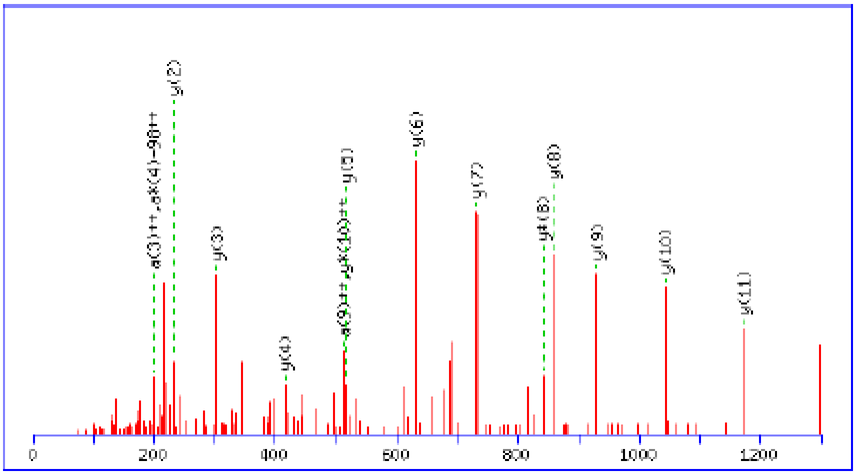

Monoisotopic mass of neutral peptide Mr(calc): 1466.6515
Fixed modifications: Carbamidomethyl (C)
Variable modifications:

T1 : Phospho (ST), with neutral losses 97.9769(shown in table), 0.0000
Ions Score: 71 **Expect:** 0.00027
Matches (**Bold Red**): 15/232 fragment ions using 27 most intense peaks

#	a	a++	a*	a*++	b	b++	b*	b*++	Seq.	y	y++	y*	y*++	#
1	56.0495	28.5284			84.0444	42.5258			T					13
2	170.0924	85.5498	153.0658	77.0366	198.0873	99.5473	181.0608	91.0340	N	1286.6448	643.8260	1269.6182	635.3127	12
3	299.1350	150.0711	282.1084	141.5579	327.1299	164.0686	310.1034	155.5553	E	**1172.6018**	586.8046	1155.5753	578.2913	11
4	413.1779	207.0926	396.1514	**198.5793**	441.1728	221.0901	424.1463	212.5768	N	**1043.5592**	522.2833	1026.5327	**513.7700**	10
5	484.2150	242.6112	467.1885	234.0979	512.2099	256.6086	495.1834	248.0953	A	**929.5163**	465.2618	912.4898	456.7485	9
6	612.2736	306.6404	595.2471	298.1272	640.2685	320.6379	623.2420	312.1246	Q	**858.4792**	429.7432	**841.4526**	421.2300	8
7	711.3420	356.1746	694.3155	347.6614	739.3369	370.1721	722.3104	361.6588	V	**730.4206**	365.7139	713.3941	357.2007	7
8	825.3849	413.1961	808.3584	404.6828	853.3799	427.1936	836.3533	418.6803	N	**631.3522**	316.1797	614.3257	307.6665	6
9	926.4326	463.7200	909.4061	455.2067	954.4275	477.7174	937.4010	469.2041	T	**517.3093**	259.1583	500.2827	250.6450	5
10	1039.5167	520.2620	1022.4901	511.7487	1067.5116	534.2594	1050.4851	525.7462	L	**416.2616**	208.6344	399.2350	200.1212	4
11	1110.5538	555.7805	1093.5273	547.2673	1138.5487	569.7780	1121.5222	561.2647	A	**303.1775**	152.0924	286.1510	143.5791	3
12	1167.5753	584.2913	1150.5487	575.7780	1195.5702	598.2887	1178.5436	589.7755	G	**232.1404**	116.5738	215.1139	108.0606	2
13									R	175.1190	88.0631	158.0924	79.5498	1

MS/MS Fragmentation of VPELVAKTELENIAK
Found in gi|9758672, unnamed protein product [Arabidopsis thaliana]

Match to Query 122: 1732.817168 from(867.415860,2+) intensity(15089.0000)

Title: 103: Scan 1444 (rt=34.768, f=3, i=101)

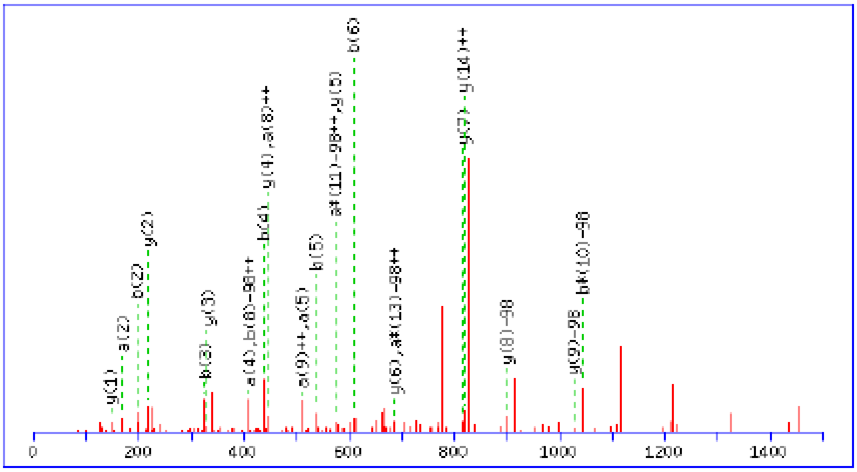


```
Monoisotopic mass of neutral peptide Mr(calc): 1732.9012
Fixed modifications: Carbamidomethyl (C)
Variable modifications:
T8      : Phospho (ST), with neutral losses 97.9769(shown in table), 0.0000
Ions Score: 48   Expect: 0.035
```

Matches (Bold Red): 24/228 fragment ions using 41 most intense peaks

#	a	a++	a*	a*++	b	b++	b*	b*++	Seq.	y	y++	y*	y*++	#
1	72.0808	36.5440			100.0757	50.5415			V					15
2	**169.1335**	85.0704			**197.1285**	99.0679			P	1536.8632	768.9352	1519.8366	760.4220	14
3	298.1761	149.5917			**326.1710**	163.5892			E	1439.8104	720.4088	1422.7839	711.8956	13
4	**411.2602**	206.1337			**439.2551**	220.1312			L	1310.7678	655.8875	1293.7413	647.3743	12
5	**510.3286**	255.6679			**538.3235**	269.6654			V	1197.6838	599.3455	1180.6572	590.8322	11
6	581.3657	291.1865			**609.3606**	305.1840			A	1098.6153	549.8113	1081.5888	541.2980	10
7	709.4607	355.2340	692.4341	346.7207	737.4556	369.2314	720.4291	360.7182	K	**1027.5782**	514.2928	1010.5517	505.7795	9
8	792.4978	396.7525	775.4712	388.2393	820.4927	**410.7500**	803.4662	402.2367	T	**899.4833**	450.2453	882.4567	441.7320	8
9	921.5404	461.2738	904.5138	452.7606	949.5353	475.2713	932.5088	466.7580	E	**816.4462**	408.7267	799.4196	400.2134	7
10	1034.6245	517.8159	1017.5979	509.3026	1062.6194	531.8133	**1045.5928**	523.3000	L	**687.4036**	344.2054	670.3770	335.6921	6
11	1163.6670	582.3372	1146.6405	**573.8239**	1191.6620	596.3346	1174.6354	587.8213	E	**574.3195**	287.6634	557.2930	279.1501	5
12	1277.7100	639.3586	1260.6834	630.8453	1305.7049	653.3561	1288.6783	644.8428	N	**445.2769**	223.1421	428.2504	214.6288	4
13	1390.7940	695.9007	1373.7675	**687.3874**	1418.7889	709.8981	1401.7624	701.3848	I	**331.2340**	166.1206	314.2074	157.6074	3
14	1461.8311	731.4192	1444.8046	722.9059	1489.8261	745.4167	1472.7995	736.9034	A	**218.1499**	109.5786	201.1234	101.0653	2
15									K	**147.1128**	74.0600	130.0863	65.5468	1
